# Supplementary material for: Healthy dietary patterns and the risk of individual chronic diseases in community-dwelling adults
Source: Nat Commun. 2023 Oct 23;14:6704. doi: 10.1038/s41467-023-42523-9 (PMC10593819; doi:10.1038/s41467-023-42523-9)
Supplement: Supplementary file 1 — Supplementary Information [file 41467_2023_42523_MOESM1_ESM.pdf]

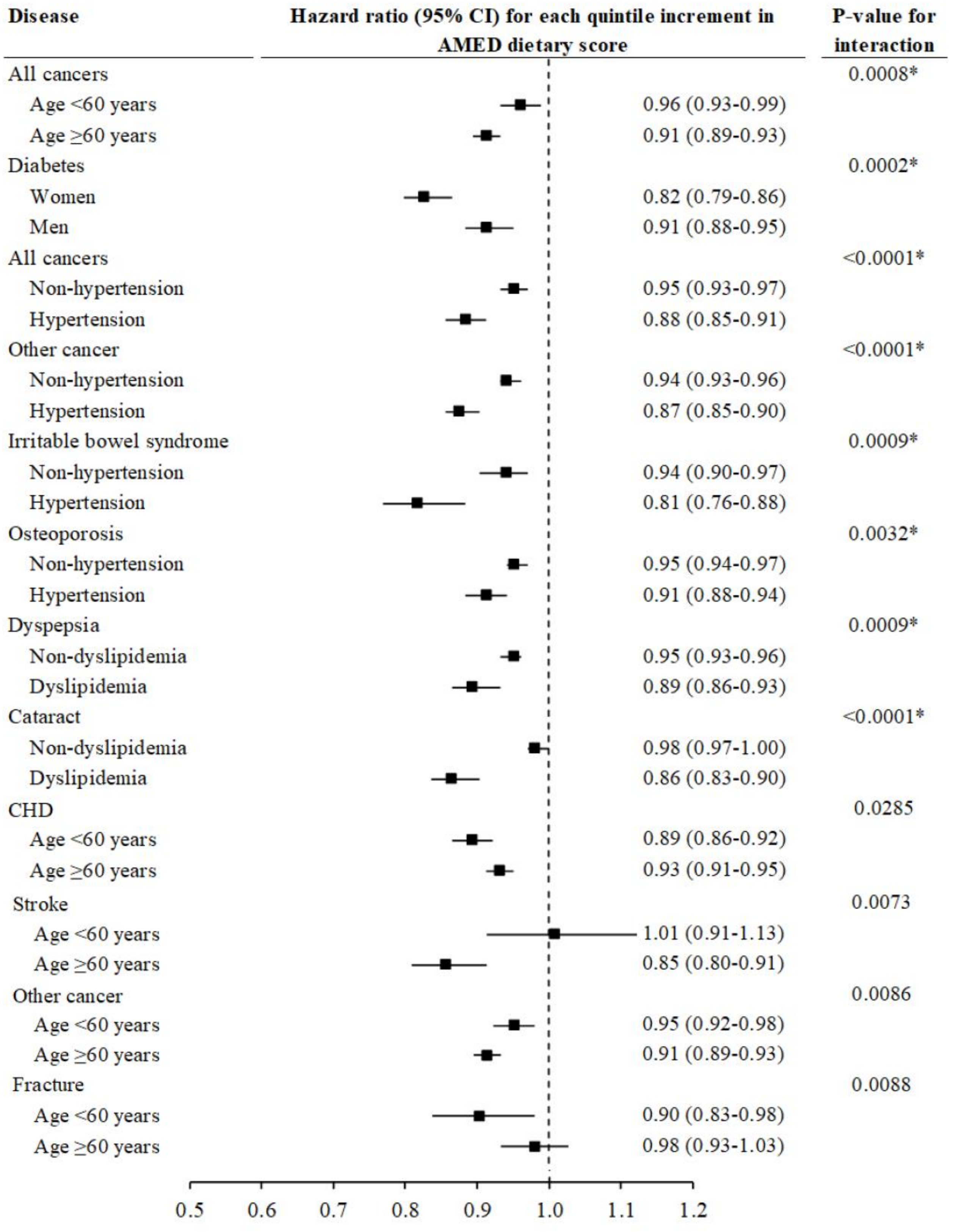

Disease
Hazard ratio (95% CI) for each quintile increment in AMED dietary score
P-value for interaction
All cancers
0.0008*
Age <60 years
0.96 (0.93-0.99)
Age ≥60 years
0.91 (0.89-0.93)
Diabetes
0.0002*
Women
0.82 (0.79-0.86)
Men
0.91 (0.88-0.95)
All cancers
<0.0001*
Non-hypertension
0.95 (0.93-0.97)
Hypertension
0.88 (0.85-0.91)
Other cancer
<0.0001*
Non-hypertension
0.94 (0.93-0.96)
Hypertension
0.87 (0.85-0.90)
Irritable bowel syndrome
0.0009*
Non-hypertension
0.94 (0.90-0.97)
Hypertension
0.81 (0.76-0.88)
Osteoporosis
0.0032*
Non-hypertension
0.95 (0.94-0.97)
Hypertension
0.91 (0.88-0.94)
Dyspepsia
0.0009*
Non-dyslipidemia
0.95 (0.93-0.96)
Dyslipidemia
0.89 (0.86-0.93)
Cataract
<0.0001*
Non-dyslipidemia
0.98 (0.97-1.00)
Dyslipidemia
0.86 (0.83-0.90)
CHD
0.0285
Age <60 years
0.89 (0.86-0.92)
Age ≥60 years
0.93 (0.91-0.95)
Stroke
0.0073
Age <60 years
1.01 (0.91-1.13)
Age ≥60 years
0.85 (0.80-0.91)
Other cancer
0.0086
Age <60 years
0.95 (0.92-0.98)
Age ≥60 years
0.91 (0.89-0.93)
Fracture
0.0088
Age <60 years
0.90 (0.83-0.98)
Age ≥60 years
0.98 (0.93-1.03)
0.5
0.6
0.7
0.8
0.9
1.0
1.1
1.2

Continued

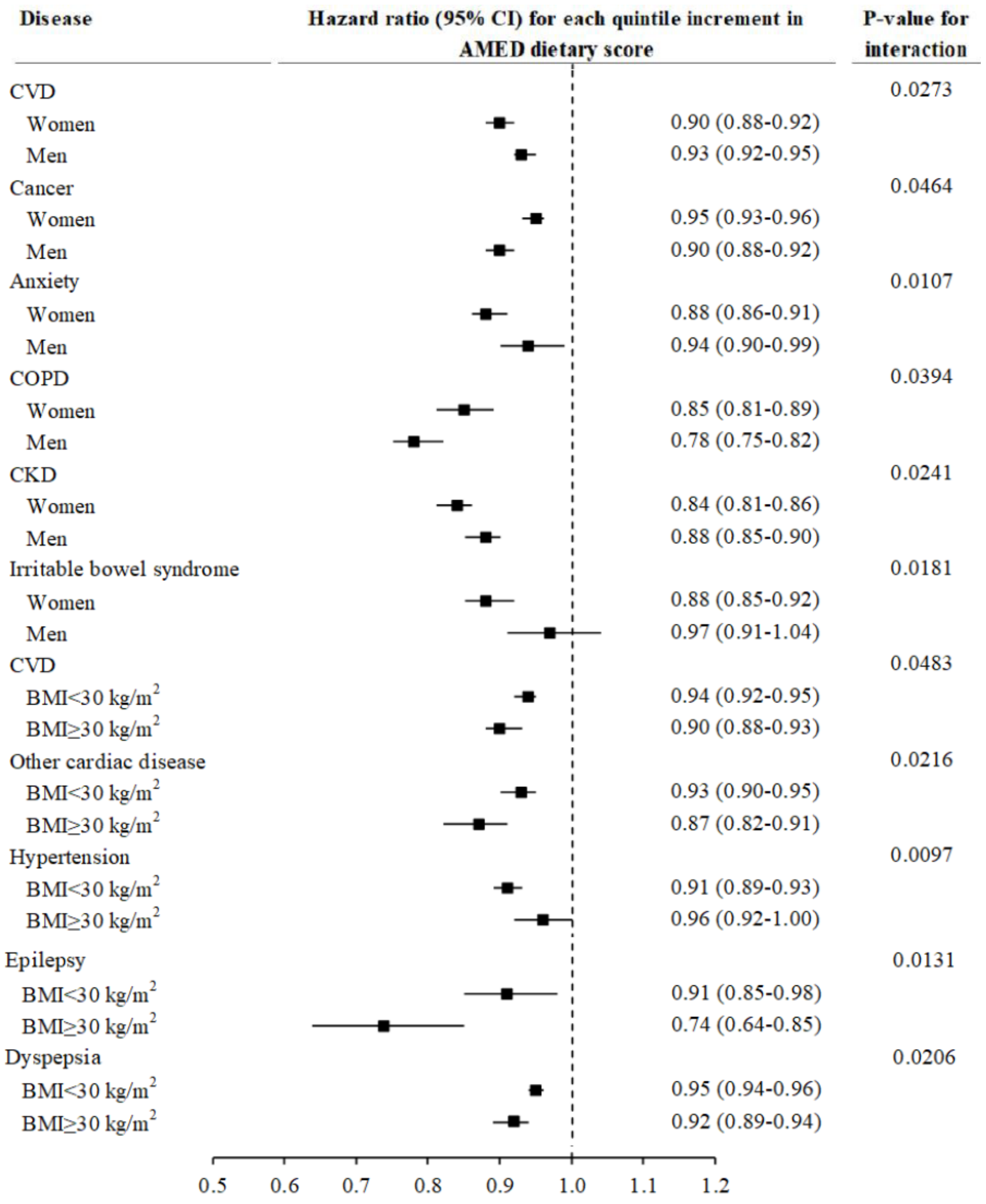
Disease
Hazard ratio (95% CI) for each quintile increment in AMED dietary score
P-value for interaction
CVD
0.0273
Women
0.90 (0.88-0.92)
Men
0.93 (0.92-0.95)
Cancer
0.0464
Women
0.95 (0.93-0.96)
Men
0.90 (0.88-0.92)
Anxiety
0.0107
Women
0.88 (0.86-0.91)
Men
0.94 (0.90-0.99)
COPD
0.0394
Women
0.85 (0.81-0.89)
Men
0.78 (0.75-0.82)
CKD
0.0241
Women
0.84 (0.81-0.86)
Men
0.88 (0.85-0.90)
Irritable bowel syndrome
0.0181
Women
0.88 (0.85-0.92)
Men
0.97 (0.91-1.04)
CVD
0.0483
BMI<30 kg/m2
0.94 (0.92-0.95)
BMI≥30 kg/m2
0.90 (0.88-0.93)
Other cardiac disease
0.0216
BMI<30 kg/m2
0.93 (0.90-0.95)
BMI≥30 kg/m2
0.87 (0.82-0.91)
Hypertension
0.0097
BMI<30 kg/m2
0.91 (0.89-0.93)
BMI≥30 kg/m2
0.96 (0.92-1.00)
Epilepsy
0.0131
BMI<30 kg/m2
0.91 (0.85-0.98)
BMI≥30 kg/m2
0.74 (0.64-0.85)
Dyspepsia
0.0206
BMI<30 kg/m2
0.95 (0.94-0.96)
BMI≥30 kg/m2
0.92 (0.89-0.94)
0.5
0.6
0.7
0.8
0.9
1.0
1.1
1.2

Continued

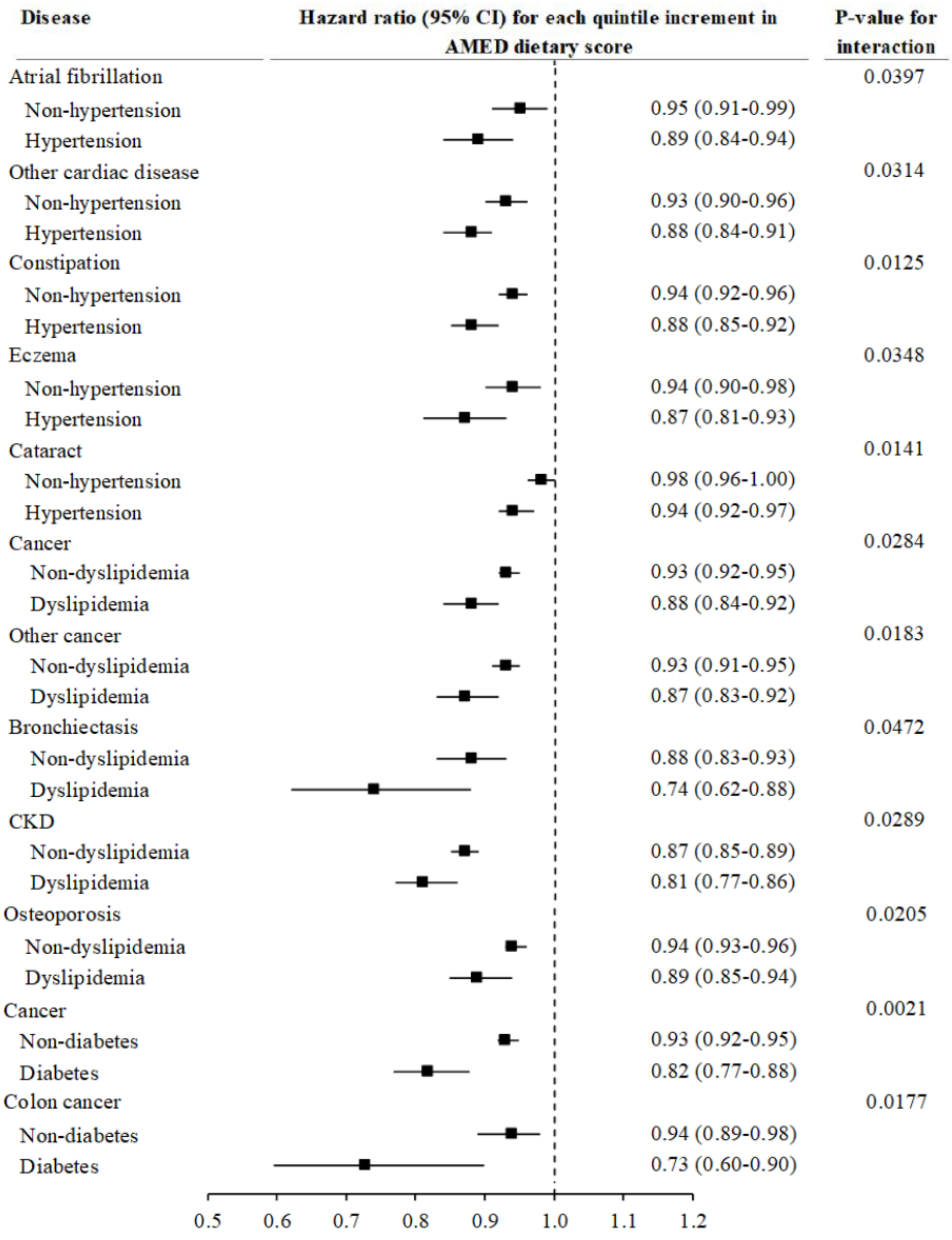

Disease
Hazard ratio (95% CI) for each quintile increment in AMED dietary score
P-value for interaction
Atrial fibrillation
0.0397
Non-hypertension
0.95 (0.91-0.99)
Hypertension
0.89 (0.84-0.94)
Other cardiac disease
0.0314
Non-hypertension
0.93 (0.90-0.96)
Hypertension
0.88 (0.84-0.91)
Constipation
0.0125
Non-hypertension
0.94 (0.92-0.96)
Hypertension
0.88 (0.85-0.92)
Eczema
0.0348
Non-hypertension
0.94 (0.90-0.98)
Hypertension
0.87 (0.81-0.93)
Cataract
0.0141
Non-hypertension
0.98 (0.96-1.00)
Hypertension
0.94 (0.92-0.97)
Cancer
0.0284
Non-dyslipidemia
0.93 (0.92-0.95)
Dyslipidemia
0.88 (0.84-0.92)
Other cancer
0.0183
Non-dyslipidemia
0.93 (0.91-0.95)
Dyslipidemia
0.87 (0.83-0.92)
Bronchiectasis
0.0472
Non-dyslipidemia
0.88 (0.83-0.93)
Dyslipidemia
0.74 (0.62-0.88)
CKD
0.0289
Non-dyslipidemia
0.87 (0.85-0.89)
Dyslipidemia
0.81 (0.77-0.86)
Osteoporosis
0.0205
Non-dyslipidemia
0.94 (0.93-0.96)
Dyslipidemia
0.89 (0.85-0.94)
Cancer
0.0021
Non-diabetes
0.93 (0.92-0.95)
Diabetes
0.82 (0.77-0.88)
Colon cancer
0.0177
Non-diabetes
0.94 (0.89-0.98)
Diabetes
0.73 (0.60-0.90)
0.5
0.6
0.7
0.8
0.9
1.0
1.1
1.2

Continued

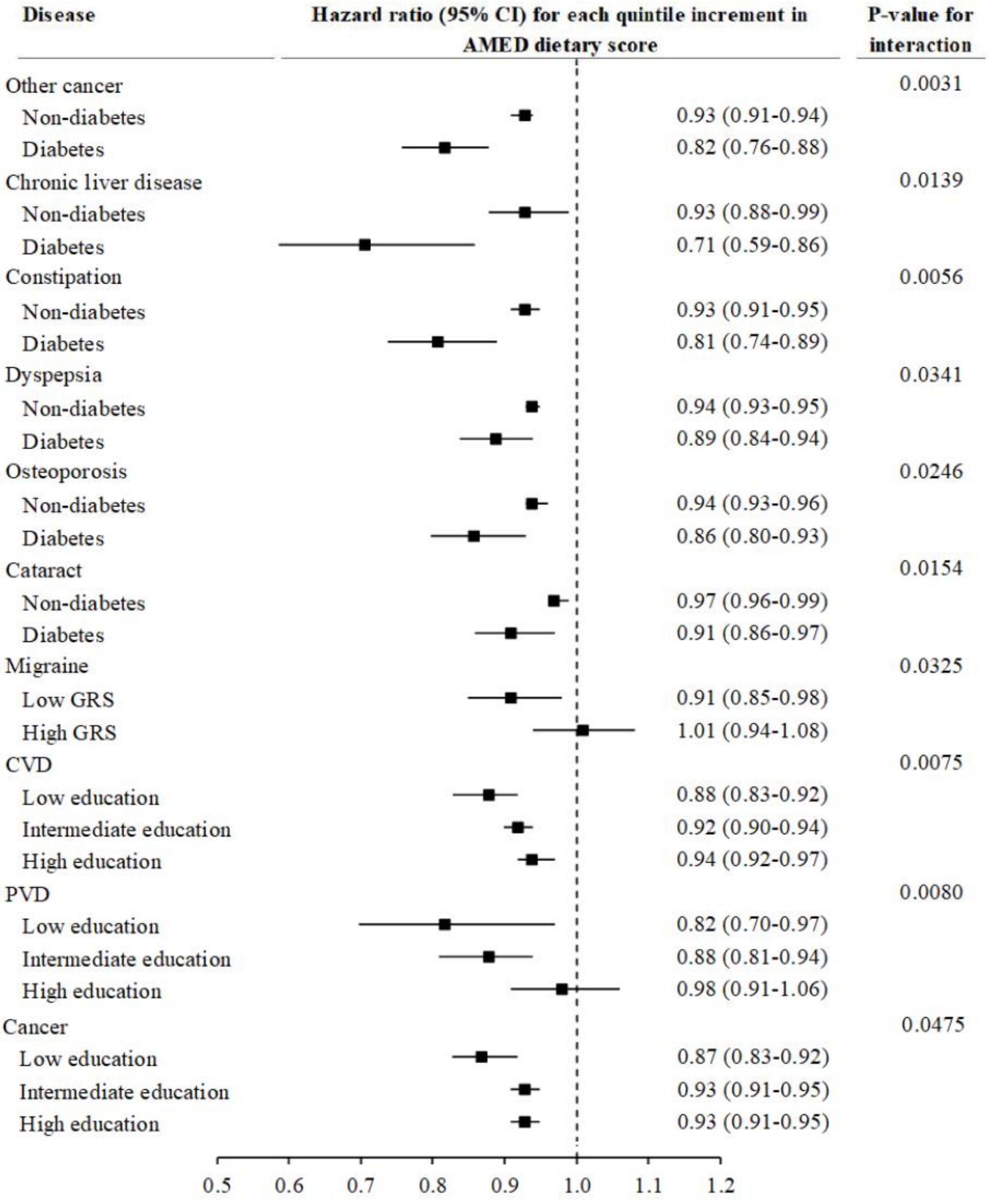
Disease
Hazard ratio (95% CI) for each quintile increment in AMED dietary score
P-value for interaction
Other cancer
0.0031
Non-diabetes
0.93 (0.91-0.94)
Diabetes
0.82 (0.76-0.88)
Chronic liver disease
0.0139
Non-diabetes
0.93 (0.88-0.99)
Diabetes
0.71 (0.59-0.86)
Constipation
0.0056
Non-diabetes
0.93 (0.91-0.95)
Diabetes
0.81 (0.74-0.89)
Dyspepsia
0.0341
Non-diabetes
0.94 (0.93-0.95)
Diabetes
0.89 (0.84-0.94)
Osteoporosis
0.0246
Non-diabetes
0.94 (0.93-0.96)
Diabetes
0.86 (0.80-0.93)
Cataract
0.0154
Non-diabetes
0.97 (0.96-0.99)
Diabetes
0.91 (0.86-0.97)
Migraine
0.0325
Low GRS
0.91 (0.85-0.98)
High GRS
1.01 (0.94-1.08)
CVD
0.0075
Low education
0.88 (0.83-0.92)
Intermediate education
0.92 (0.90-0.94)
High education
0.94 (0.92-0.97)
PVD
0.0080
Low education
0.82 (0.70-0.97)
Intermediate education
0.88 (0.81-0.94)
High education
0.98 (0.91-1.06)
Cancer
0.0475
Low education
0.87 (0.83-0.92)
Intermediate education
0.93 (0.91-0.95)
High education
0.93 (0.91-0.95)
0.5
0.6
0.7
0.8
0.9
1.0
1.1
1.2

Continued

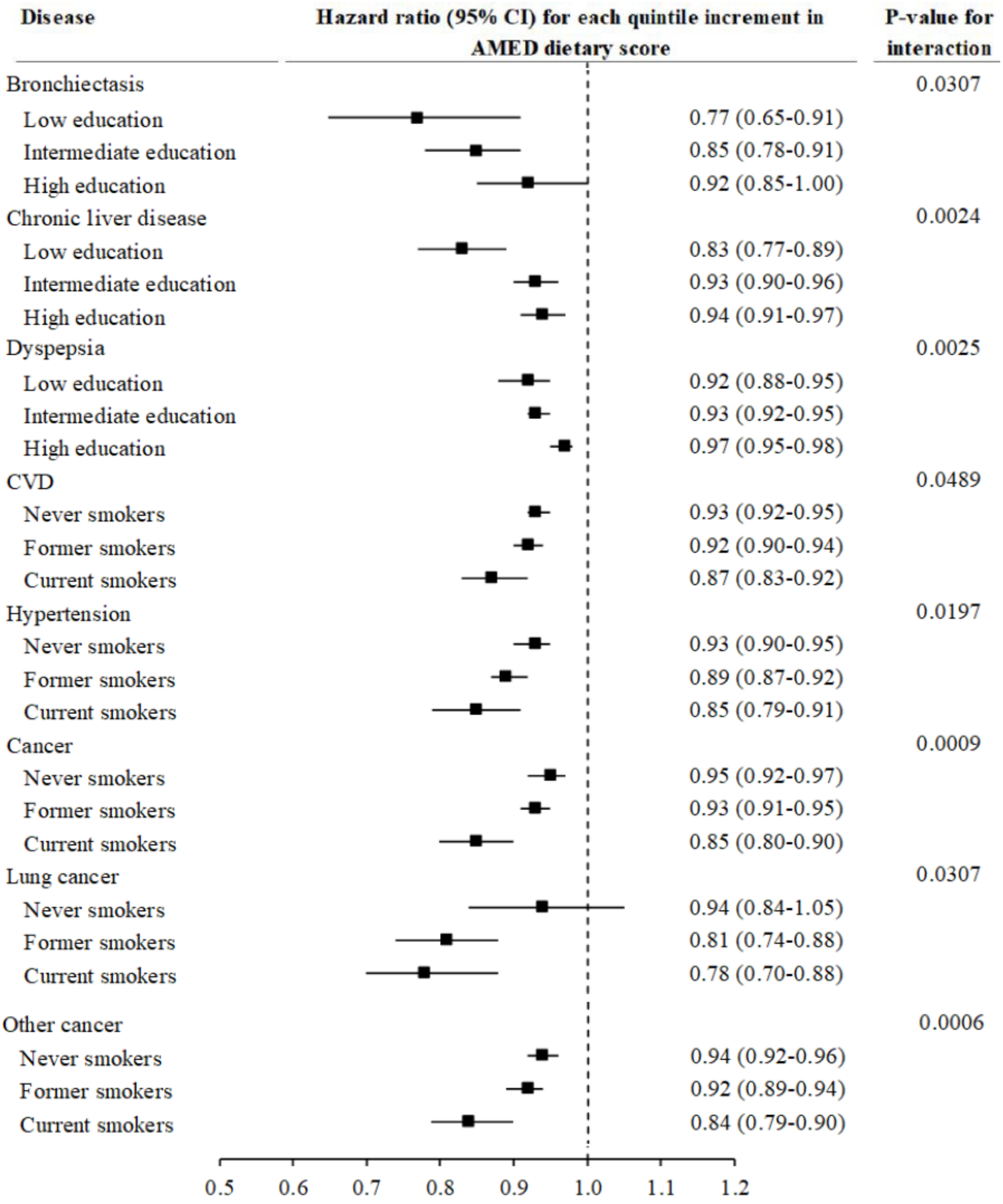
Disease
Hazard ratio (95% CI) for each quintile increment in AMED dietary score
P-value for interaction
Bronchiectasis
0.0307
Low education
0.77 (0.65-0.91)
Intermediate education
0.85 (0.78-0.91)
High education
0.92 (0.85-1.00)
Chronic liver disease
0.0024
Low education
0.83 (0.77-0.89)
Intermediate education
0.93 (0.90-0.96)
High education
0.94 (0.91-0.97)
Dyspepsia
0.0025
Low education
0.92 (0.88-0.95)
Intermediate education
0.93 (0.92-0.95)
High education
0.97 (0.95-0.98)
CVD
0.0489
Never smokers
0.93 (0.92-0.95)
Former smokers
0.92 (0.90-0.94)
Current smokers
0.87 (0.83-0.92)
Hypertension
0.0197
Never smokers
0.93 (0.90-0.95)
Former smokers
0.89 (0.87-0.92)
Current smokers
0.85 (0.79-0.91)
Cancer
0.0009
Never smokers
0.95 (0.92-0.97)
Former smokers
0.93 (0.91-0.95)
Current smokers
0.85 (0.80-0.90)
Lung cancer
0.0307
Never smokers
0.94 (0.84-1.05)
Former smokers
0.81 (0.74-0.88)
Current smokers
0.78 (0.70-0.88)
Other cancer
0.0006
Never smokers
0.94 (0.92-0.96)
Former smokers
0.92 (0.89-0.94)
Current smokers
0.84 (0.79-0.90)
0.5
0.6
0.7
0.8
0.9
1.0
1.1
1.2

Continued

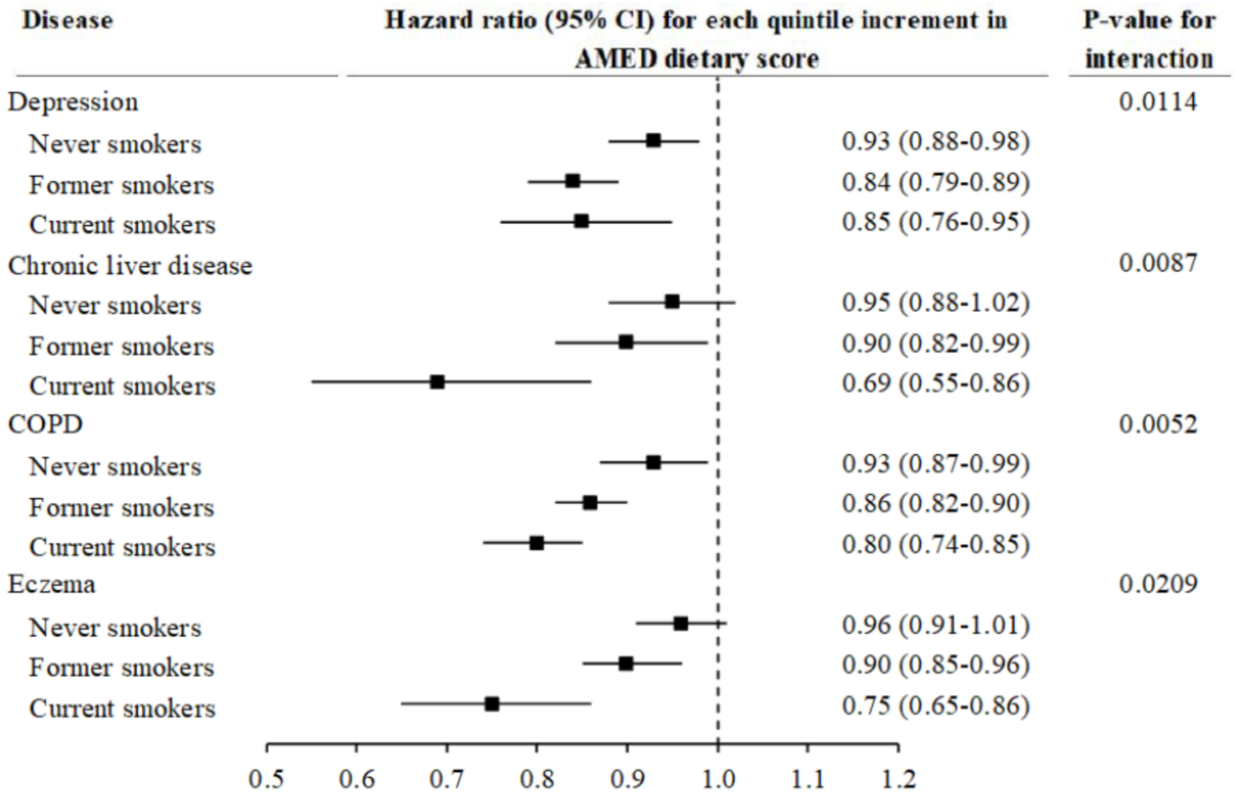
Disease
Hazard ratio (95% CI) for each quintile increment in AMED dietary score
P-value for interaction
Depression
0.0114
Never smokers
0.93 (0.88-0.98)
Former smokers
0.84 (0.79-0.89)
Current smokers
0.85 (0.76-0.95)
Chronic liver disease
0.0087
Never smokers
0.95 (0.88-1.02)
Former smokers
0.90 (0.82-0.99)
Current smokers
0.69 (0.55-0.86)
COPD
0.0052
Never smokers
0.93 (0.87-0.99)
Former smokers
0.86 (0.82-0.90)
Current smokers
0.80 (0.74-0.85)
Eczema
0.0209
Never smokers
0.96 (0.91-1.01)
Former smokers
0.90 (0.85-0.96)
Current smokers
0.75 (0.65-0.86)
0.5
0.6
0.7
0.8
0.9
1.0
1.1
1.2

Figure S1. The association between the Alternate Mediterranean Diet Index and the risk of individual chronic diseases moderated by important factors

BMI, body mass index; CHD, coronary heart disease; CKD, chronic kidney disease; COPD, chronic obstructive pulmonary disease; CVD, cardiovascular disease; GRS, genetic risk score; PVD, peripheral artery disease.

Cardiovascular disease includes coronary heart disease, heart failure, atrial fibrillation, other cardiac disease, stroke, and peripheral vascular disease. All cancers encompass any type of cancer except for non-melanoma skin cancer. Cox proportional regression models were used to test whether the association between the Alternate Mediterranean Diet Index and the risk of 48 individual chronic diseases was moderated by age, sex, obesity, hypertension, diabetes, dyslipidemia, GRS for longevity, and education. Horizontal lines indicate the range of the 95% confidence interval. The vertical dash lines represent the hazard ratio of 1. Only the results with significant interaction (P-value <0.05) are shown in this figure.
* Indicates significant interaction while controlling for false discovery rate (FDR), with two-sided statistical tests.

Disease	Hazard ratio (95% CI) for each quintile increment in AEDII dietary score	P-value for interaction
Diabetes		<0.0001*
Age <60 years	0.74 (0.70-0.77)	
Age ≥60 years	0.86 (0.83-0.89)	
Chronic kidney disease		0.0002*
Age <60 years	0.80 (0.76-0.84)	
Age ≥60 years	0.88 (0.86-0.90)	
CVD		0.0263
Age <60 years	0.92 (0.89-0.95)	
Age ≥60 years	0.96 (0.94-0.97)	
Other cardiac disease		0.0348
Age <60 years	0.91 (0.86-0.95)	
Age ≥60 years	0.96 (0.94-0.99)	
Other cancer		0.0440
Age <60 years	0.94 (0.92-0.97)	
Age ≥60 years	0.98 (0.96-0.996)	
Anxiety		0.0050
Age <60 years	0.88 (0.85-0.92)	
Age ≥60 years	0.95 (0.92-0.98)	
Alcohol use disorder		0.0108
Age <60 years	1.02 (0.96-1.09)	
Age ≥60 years	1.15 (1.08-1.23)	
Cataract		0.0422
Age <60 years	0.93 (0.90-0.96)	
Age ≥60 years	0.97 (0.96-0.99)	
CVD		0.0027
Women	0.92 (0.90-0.94)	
Men	0.97 (0.95-0.99)	
Atrial fibrillation		0.0036
Women	0.90 (0.85-0.95)	
Men	1.00 (0.96-1.05)	
Diabetes		0.0183
Women	0.78 (0.75-0.82)	
Men	0.84 (0.81-0.87)	
Chronic kidney disease		0.0048
Women	0.83 (0.81-0.86)	
Men	0.89 (0.86-0.92)	
Inflammatory bowel disease		0.0490
Women	0.96 (0.89-1.03)	
Men	0.85 (0.78-0.92)	
Thyroid disorder		0.0310
Women	0.89 (0.86-0.92)	
Men	0.96 (0.91-1.01)	
Psychoactive substance abuse		0.0306
BMI<30 kg/m^2	0.98 (0.89-1.08)	
BMI≥30 kg/m^2	1.21 (1.03-1.42)	
CHD		0.0289
Non-hypertension	0.95 (0.93-0.98)	
Hypertension	0.92 (0.89-0.95)	
Stomach cancer		0.0381
Non-hypertension	0.87 (0.76-0.99)	
Hypertension	1.11 (0.92-1.34)	
Dementia		0.0437
Non-hypertension	1.01 (0.95-1.09)	
Hypertension	0.90 (0.82-0.99)	
Epilepsy		0.0318
Non-dislipidemia	0.97 (0.91-1.04)	
Dislipidemia	0.77 (0.64-0.94)	
Eczema		0.0387
Non-dislipidemia	0.94 (0.90-0.98)	
Dislipidemia	1.06 (0.95-1.18)	
Non-melanoma skin cancer		0.0197
Non-diabetes	1.00 (0.98-1.02)	
Diabetes	1.13 (1.02-1.26)	
Pernicious anaemia		0.0149
Non-diabetes	0.80 (0.72-0.90)	
Diabetes	1.28 (0.93-1.76)	
Hypertension		0.0346
Low GRS	0.91 (0.88-0.93)	
High GRS	0.94 (0.92-0.97)	
Alcohol use disorder		0.0417
Low GRS	1.04 (0.98-1.11)	
High GRS	1.14 (1.07-1.22)	
Chronic liver disease		0.0370
Low education	0.80 (0.65-0.98)	
Intermediate education	0.91 (0.84-0.99)	
High education	0.93 (0.86-1.01)	
Psychoactive substance abuse		0.0147
Never smokers	1.09 (0.94-1.27)	
Former smokers	1.01 (0.89-1.15)	
Current smokers	0.83 (0.71-0.98)	

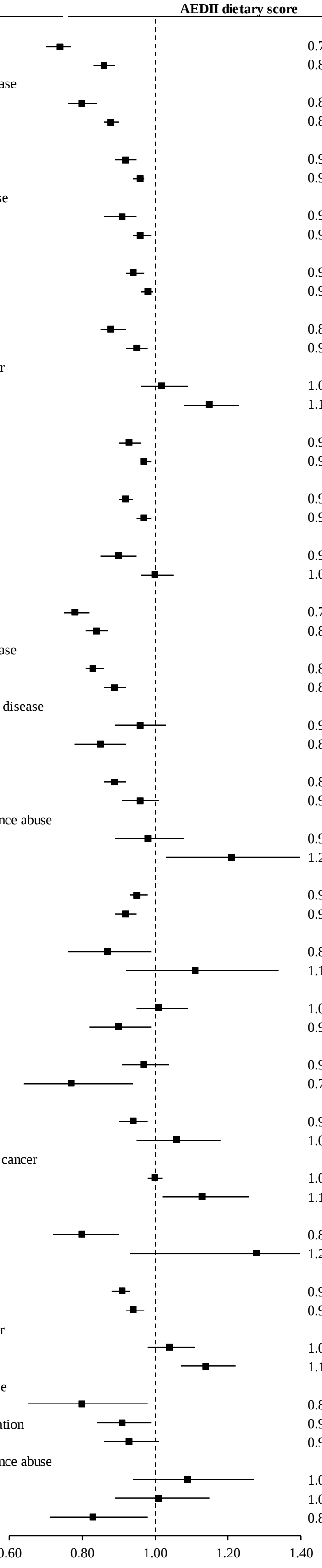

Figure S2. The association between the Anti-Empirical Dietary Inflammatory Index and the risk of individual chronic diseases moderated by important factors

BMI, body mass index; CHD, coronary heart disease; CVD, cardiovascular disease; GRS, genetic risk score.

Cardiovascular disease includes coronary heart disease, heart failure, atrial fibrillation, other cardiac disease, stroke, and peripheral vascular disease. Cox proportional regression models were used to test whether the association between the Anti-Empirical Dietary Inflammatory Index and the risk of 48 individual chronic diseases was moderated by age, sex, obesity, hypertension, diabetes, dyslipidemia, GRS for longevity, and education. Horizontal lines indicate the range of the 95% confidence interval. The vertical dash lines represent the hazard ratio of 1. Only the results with significant interaction (P-value <0.05) are shown in this figure.

*Indicates significant interaction while controlling for false discovery rate (FDR), with two-sided statistical tests.

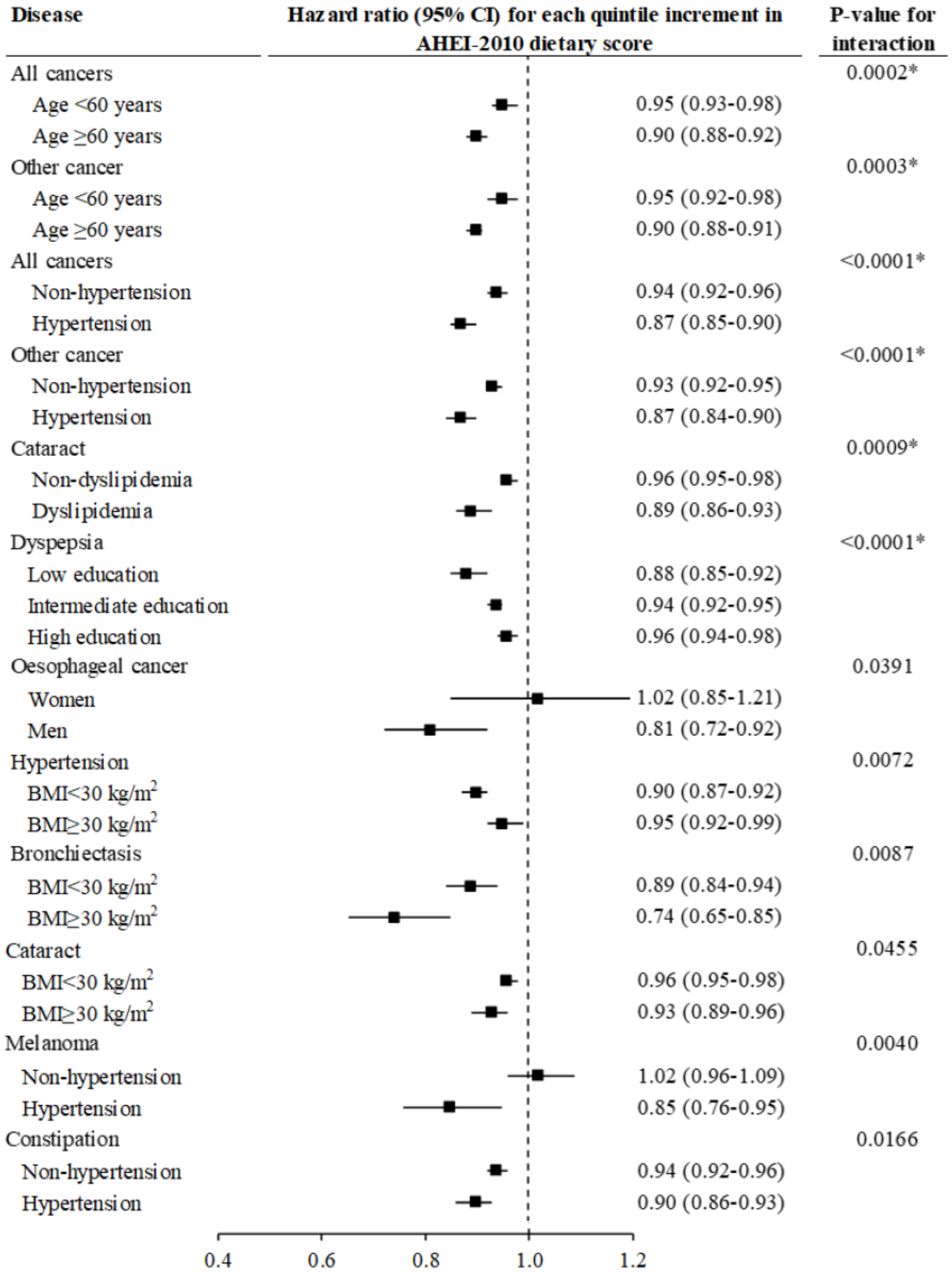
Disease
Hazard ratio (95% CI) for each quintile increment in AHEI-2010 dietary score
P-value for interaction
All cancers
0.0002*
Age <60 years
0.95 (0.93-0.98)
Age ≥60 years
0.90 (0.88-0.92)
Other cancer
0.0003*
Age <60 years
0.95 (0.92-0.98)
Age ≥60 years
0.90 (0.88-0.91)
All cancers
<0.0001*
Non-hypertension
0.94 (0.92-0.96)
Hypertension
0.87 (0.85-0.90)
Other cancer
<0.0001*
Non-hypertension
0.93 (0.92-0.95)
Hypertension
0.87 (0.84-0.90)
Cataract
0.0009*
Non-dyslipidemia
0.96 (0.95-0.98)
Dyslipidemia
0.89 (0.86-0.93)
Dyspepsia
<0.0001*
Low education
0.88 (0.85-0.92)
Intermediate education
0.94 (0.92-0.95)
High education
0.96 (0.94-0.98)
Oesophageal cancer
0.0391
Women
1.02 (0.85-1.21)
Men
0.81 (0.72-0.92)
Hypertension
0.0072
BMI<30 kg/m2
0.90 (0.87-0.92)
BMI≥30 kg/m2
0.95 (0.92-0.99)
Bronchiectasis
0.0087
BMI<30 kg/m2
0.89 (0.84-0.94)
BMI≥30 kg/m2
0.74 (0.65-0.85)
Cataract
0.0455
BMI<30 kg/m2
0.96 (0.95-0.98)
BMI≥30 kg/m2
0.93 (0.89-0.96)
Melanoma
0.0040
Non-hypertension
1.02 (0.96-1.09)
Hypertension
0.85 (0.76-0.95)
Constipation
0.0166
Non-hypertension
0.94 (0.92-0.96)
Hypertension
0.90 (0.86-0.93)
0.4
0.6
0.8
1.0
1.2

Continued

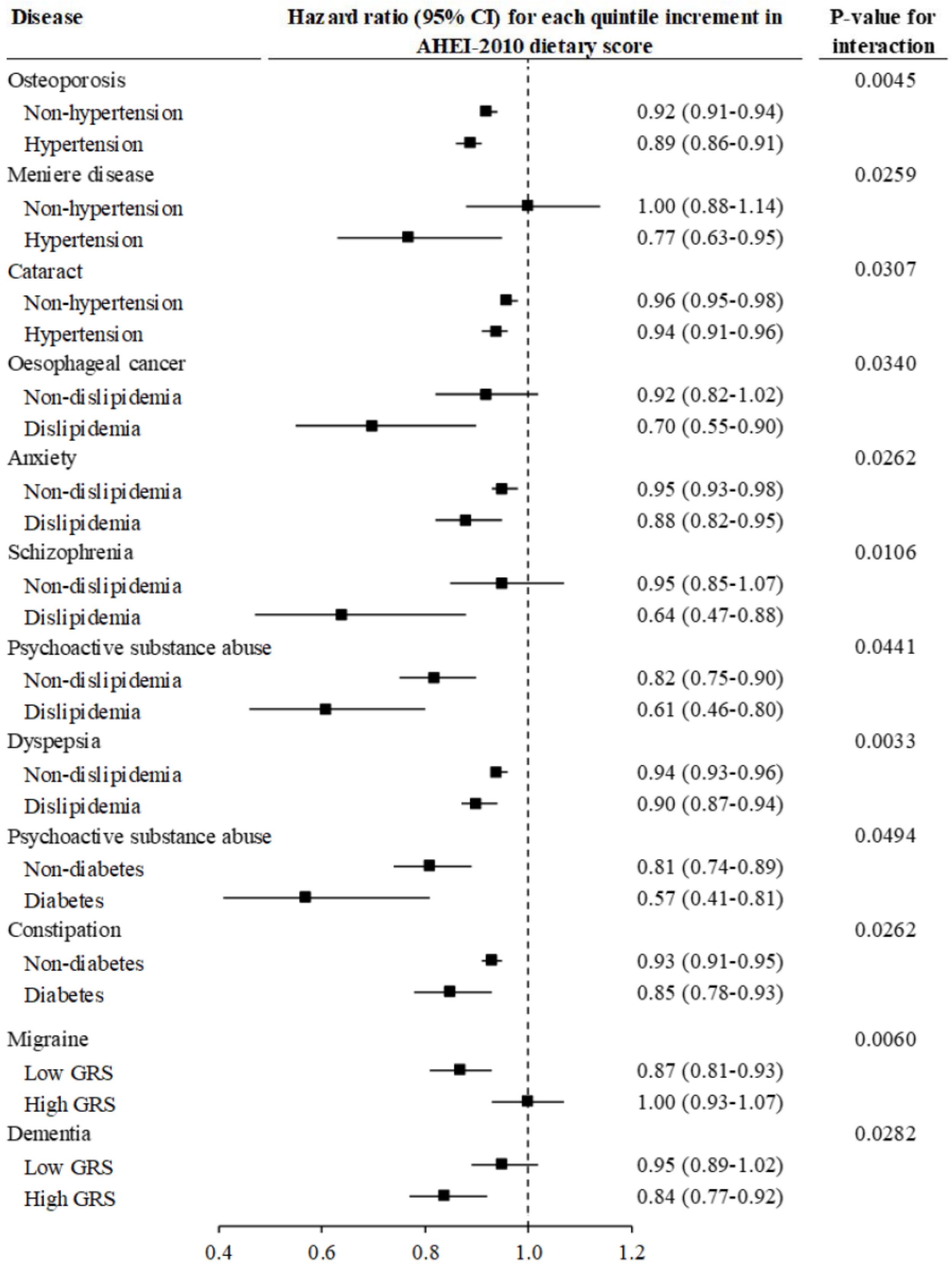
Disease
Hazard ratio (95% CI) for each quintile increment in AHEI-2010 dietary score
P-value for interaction
Osteoporosis
0.0045
Non-hypertension
0.92 (0.91-0.94)
Hypertension
0.89 (0.86-0.91)
Meniere disease
0.0259
Non-hypertension
1.00 (0.88-1.14)
Hypertension
0.77 (0.63-0.95)
Cataract
0.0307
Non-hypertension
0.96 (0.95-0.98)
Hypertension
0.94 (0.91-0.96)
Oesophageal cancer
0.0340
Non-dislipidemia
0.92 (0.82-1.02)
Dislipidemia
0.70 (0.55-0.90)
Anxiety
0.0262
Non-dislipidemia
0.95 (0.93-0.98)
Dislipidemia
0.88 (0.82-0.95)
Schizophrenia
0.0106
Non-dislipidemia
0.95 (0.85-1.07)
Dislipidemia
0.64 (0.47-0.88)
Psychoactive substance abuse
0.0441
Non-dislipidemia
0.82 (0.75-0.90)
Dislipidemia
0.61 (0.46-0.80)
Dyspepsia
0.0033
Non-dislipidemia
0.94 (0.93-0.96)
Dislipidemia
0.90 (0.87-0.94)
Psychoactive substance abuse
0.0494
Non-diabetes
0.81 (0.74-0.89)
Diabetes
0.57 (0.41-0.81)
Constipation
0.0262
Non-diabetes
0.93 (0.91-0.95)
Diabetes
0.85 (0.78-0.93)
Migraine
0.0060
Low GRS
0.87 (0.81-0.93)
High GRS
1.00 (0.93-1.07)
Dementia
0.0282
Low GRS
0.95 (0.89-1.02)
High GRS
0.84 (0.77-0.92)
0.4
0.6
0.8
1.0
1.2

Continued

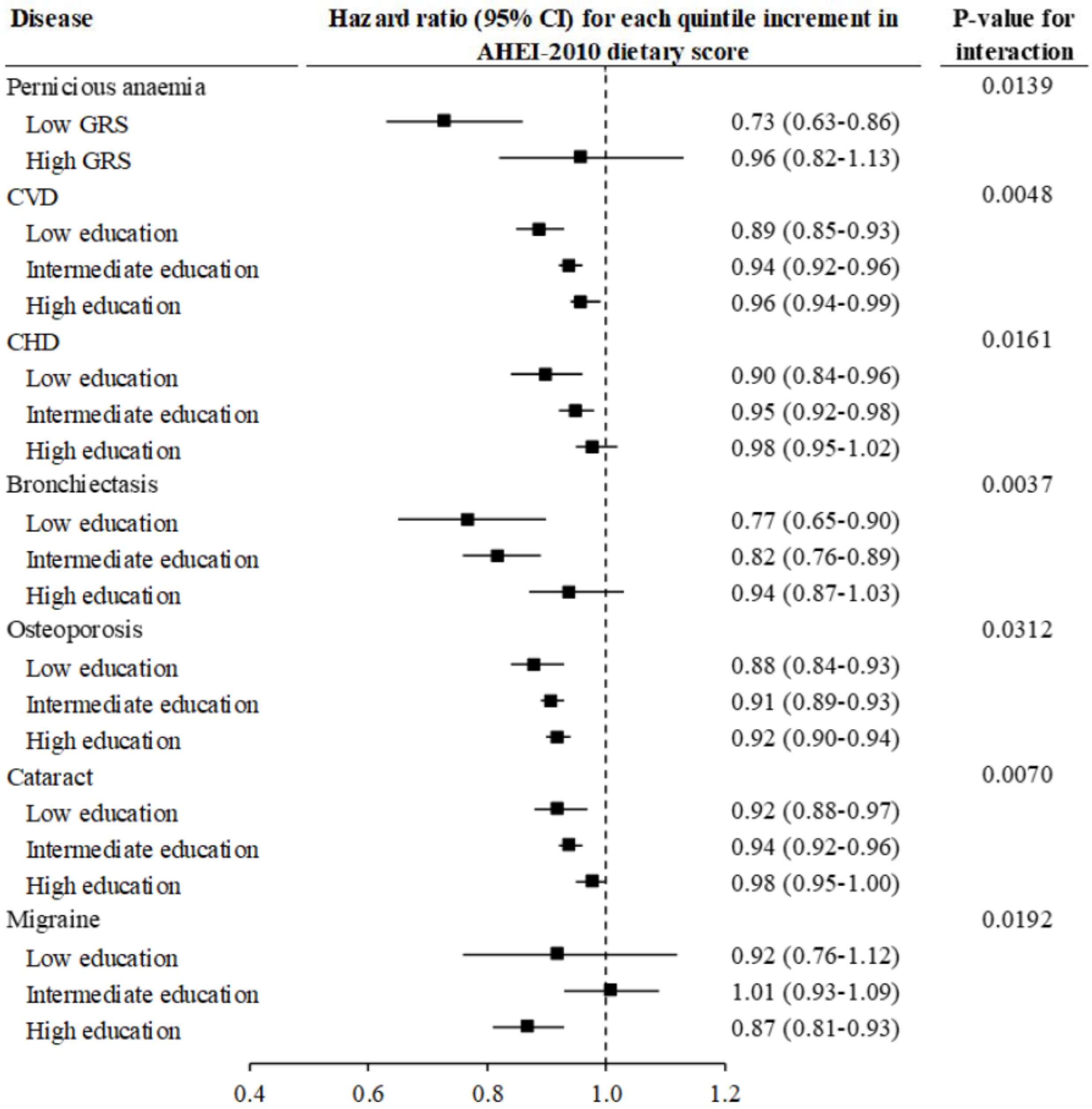
Disease
Hazard ratio (95% CI) for each quintile increment in AHEI-2010 dietary score
P-value for interaction
Pernicious anaemia
0.0139
Low GRS
0.73 (0.63-0.86)
High GRS
0.96 (0.82-1.13)
CVD
0.0048
Low education
0.89 (0.85-0.93)
Intermediate education
0.94 (0.92-0.96)
High education
0.96 (0.94-0.99)
CHD
0.0161
Low education
0.90 (0.84-0.96)
Intermediate education
0.95 (0.92-0.98)
High education
0.98 (0.95-1.02)
Bronchiectasis
0.0037
Low education
0.77 (0.65-0.90)
Intermediate education
0.82 (0.76-0.89)
High education
0.94 (0.87-1.03)
Osteoporosis
0.0312
Low education
0.88 (0.84-0.93)
Intermediate education
0.91 (0.89-0.93)
High education
0.92 (0.90-0.94)
Cataract
0.0070
Low education
0.92 (0.88-0.97)
Intermediate education
0.94 (0.92-0.96)
High education
0.98 (0.95-1.00)
Migraine
0.0192
Low education
0.92 (0.76-1.12)
Intermediate education
1.01 (0.93-1.09)
High education
0.87 (0.81-0.93)
0.4
0.6
0.8
1.0
1.2

Figure S3. The association between the Alternate Healthy Eating Index-2010 and the risk of individual chronic diseases moderated by important factors

BMI, body mass index; CHD, coronary heart disease; CVD, cardiovascular disease; GRS, genetic risk score.

Cardiovascular disease includes coronary heart disease, heart failure, atrial fibrillation, other cardiac disease, stroke, and peripheral vascular disease. All cancers encompass any type of cancer except for non-melanoma skin cancer. Cox proportional regression models were used to test whether the association between the Alternate Healthy Eating Index-2010 and the risk of 48 individual chronic diseases was moderated by age, sex, obesity, hypertension, diabetes, dyslipidemia, GRS for longevity, and education. Horizontal lines indicate the range of the 95% confidence interval. The vertical dash lines represent the hazard ratio of 1. Only the results with significant interaction (P-value <0.05) are shown in this figure.
*Indicates significant interaction while controlling for false discovery rate (FDR), with two-sided statistical tests.

Disease	Hazard ratio (95% CI) for each quintile increment in HPDI dietary score	P-value for interaction
Hypertension		0.0009*
Age <60 years	0.87 (0.84-0.90)	
Age ≥60 years	0.93 (0.90-0.95)	
CVD		0.0115
Age <60 years	0.93 (0.91-0.96)	
Age ≥60 years	0.96 (0.94-0.97)	
Atrial fibrillation		0.0039
Age <60 years	0.87 (0.80-0.94)	
Age ≥60 years	0.97 (0.94-1.01)	
Other cardiac disease		0.0472
Age <60 years	0.92 (0.88-0.97)	
Age ≥60 years	0.96 (0.93-0.98)	
Hypertension		0.0009
Age <60 years	0.87 (0.84-0.90)	
Age ≥60 years	0.93 (0.90-0.95)	
Diabetes		0.0038
Age <60 years	0.83 (0.79-0.86)	
Age ≥60 years	0.90 (0.87-0.93)	
CVD		0.0136
Women	0.93 (0.91-0.95)	
Men	0.97 (0.95-0.98)	
Heart failure		0.0280
Women	0.88 (0.83-0.93)	
Men	0.96 (0.92-1.01)	
Atrial fibrillation		0.0043
Women	0.90 (0.85-0.95)	
Men	0.99 (0.95-1.03)	
Hypertension		0.0263
Women	0.89 (0.86-0.91)	
Men	0.92 (0.90-0.95)	
Diabetes		0.0286
Women	0.84 (0.81-0.88)	
Men	0.89 (0.86-0.92)	
Anxiety		0.0470
Women	0.91 (0.89-0.94)	
Men	0.96 (0.92-1.01)	

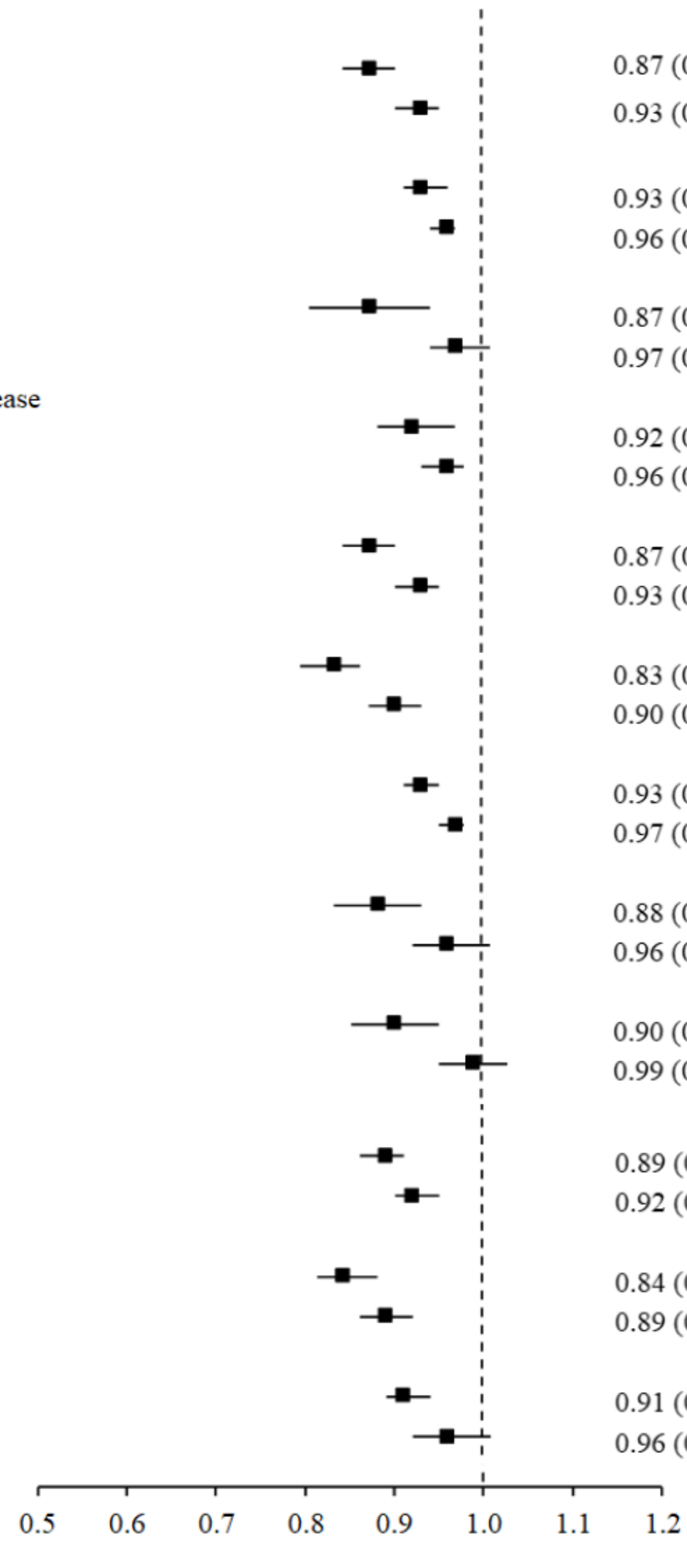

Continued

Disease	Hazard ratio (95% CI) for each quintile increment in HPDI dietary score	P-value for interaction
CKD		0.0449
Women	0.86 (0.83-0.88)	
Men	0.89 (0.87-0.92)	
Diverticular disease		0.0277
Women	0.90 (0.89-0.92)	
Men	0.93 (0.91-0.95)	
Atrial fibrillation		0.0219
BMI<30 kg/m^2	1.00 (0.96-1.04)	
BMI≥30 kg/m^2	0.91 (0.85-0.96)	
Other cardiac disease		0.0222
BMI<30 kg/m^2	0.97 (0.94-1.00)	
BMI≥30 kg/m^2	0.91 (0.86-0.95)	
Irritable bowel syndrome		0.0303
BMI<30 kg/m^2	0.86 (0.81-0.91)	
BMI≥30 kg/m^2	0.98 (0.88-1.09)	
Cataract		0.0048
BMI<30 kg/m^2	0.98 (0.96-1.00)	
BMI≥30 kg/m^2	0.92 (0.89-0.96)	
Hypertension		0.0010
Non-hypertension	0.91 (0.88-0.93)	
Hypertension	0.99 (0.95-1.03)	
Cancer		0.0162
Non-hypertension	0.96 (0.94-0.98)	
Hypertension	0.92 (0.89-0.95)	
Melanoma		0.0050
Non-hypertension	1.07 (1.00-1.14)	
Hypertension	0.89 (0.80-0.99)	
Other cancer		0.0164
Non-hypertension	0.96 (0.94-0.98)	
Hypertension	0.92 (0.89-0.94)	
Meniere disease		0.0465
Non-hypertension	0.94 (0.83-1.07)	
Hypertension	0.74 (0.60-0.91)	
Anxiety		0.0455
Non-dyslipidemia	0.94 (0.91-0.96)	
Dyslipidemia	0.87 (0.81-0.94)	

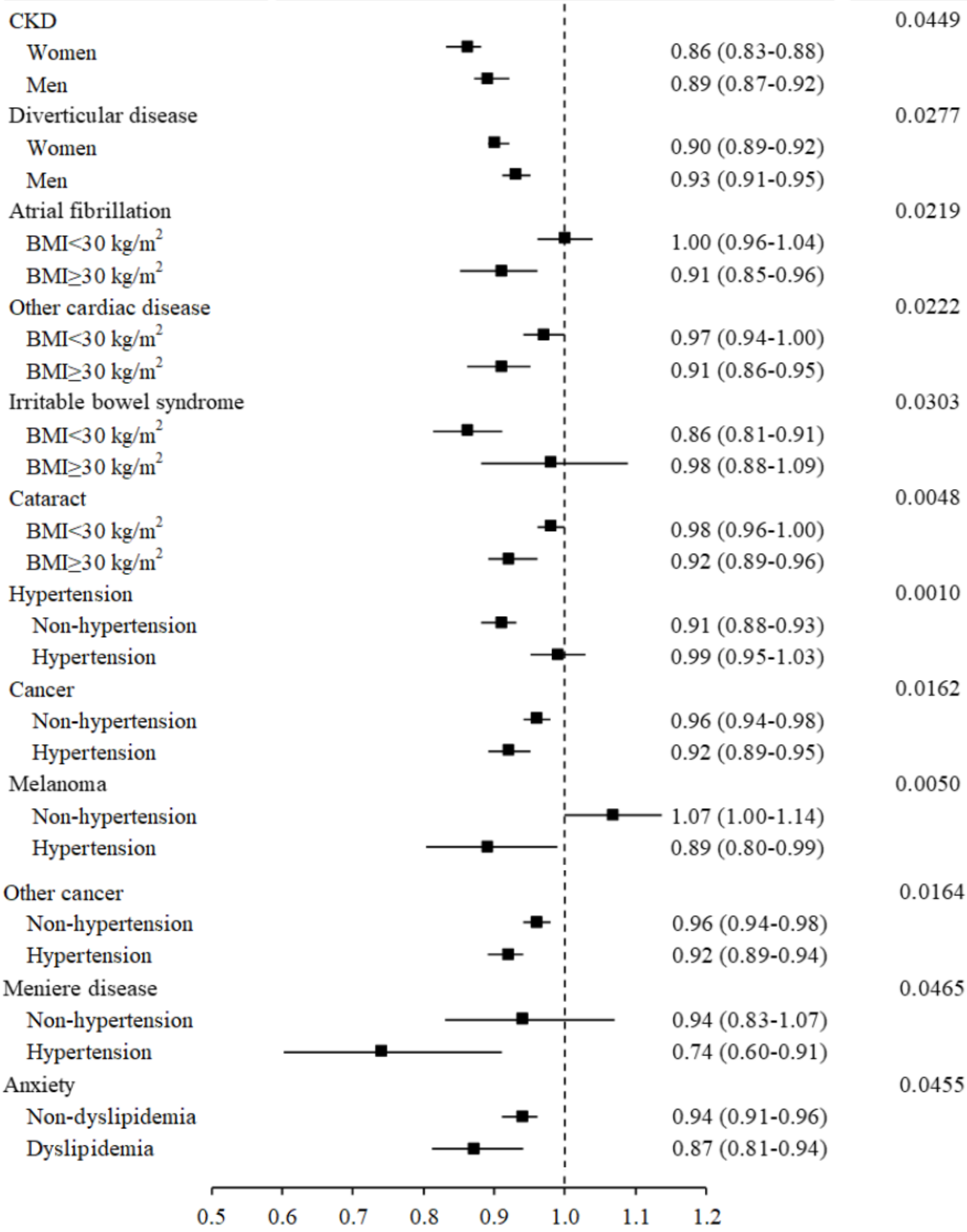

Continued

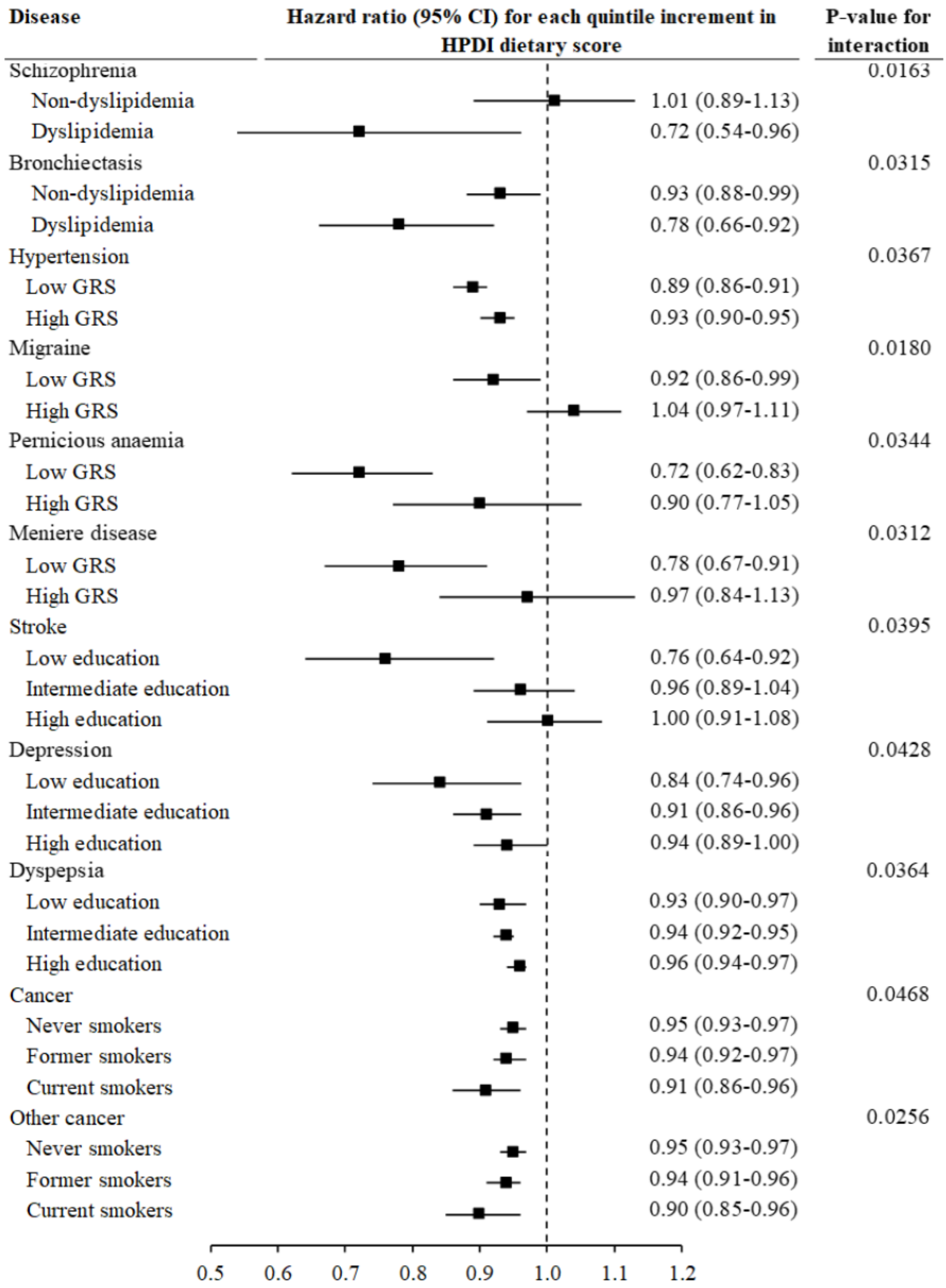
Disease
Hazard ratio (95% CI) for each quintile increment in HPDI dietary score
P-value for interaction
Schizophrenia 0.0163
Non-dyslipidemia 1.01 (0.89-1.13)
Dyslipidemia 0.72 (0.54-0.96)
Bronchiectasis 0.0315
Non-dyslipidemia 0.93 (0.88-0.99)
Dyslipidemia 0.78 (0.66-0.92)
Hypertension 0.0367
Low GRS 0.89 (0.86-0.91)
High GRS 0.93 (0.90-0.95)
Migraine 0.0180
Low GRS 0.92 (0.86-0.99)
High GRS 1.04 (0.97-1.11)
Pernicious anaemia 0.0344
Low GRS 0.72 (0.62-0.83)
High GRS 0.90 (0.77-1.05)
Meniere disease 0.0312
Low GRS 0.78 (0.67-0.91)
High GRS 0.97 (0.84-1.13)
Stroke 0.0395
Low education 0.76 (0.64-0.92)
Intermediate education 0.96 (0.89-1.04)
High education 1.00 (0.91-1.08)
Depression 0.0428
Low education 0.84 (0.74-0.96)
Intermediate education 0.91 (0.86-0.96)
High education 0.94 (0.89-1.00)
Dyspepsia 0.0364
Low education 0.93 (0.90-0.97)
Intermediate education 0.94 (0.92-0.95)
High education 0.96 (0.94-0.97)
Cancer 0.0468
Never smokers 0.95 (0.93-0.97)
Former smokers 0.94 (0.92-0.97)
Current smokers 0.91 (0.86-0.96)
Other cancer 0.0256
Never smokers 0.95 (0.93-0.97)
Former smokers 0.94 (0.91-0.96)
Current smokers 0.90 (0.85-0.96)
0.5 0.6 0.7 0.8 0.9 1.0 1.1 1.2

Figure S4. The association between the Healthful Plant-based Diet Index and the risk of individual chronic diseases moderated by important factors

BMI, body mass index; CKD, chronic kidney disease; COPD, chronic obstructive pulmonary disease; CVD, cardiovascular disease; GRS, genetic risk score.

Cardiovascular disease includes coronary heart disease, heart failure, atrial fibrillation, other cardiac disease, stroke, and peripheral vascular disease. All cancers encompass any type of cancer except for non-melanoma skin cancer. Cox proportional regression models were used to test whether the association between the Healthful Plant-based Diet Index and the risk of 48 individual chronic diseases was moderated by age, sex, obesity, hypertension, diabetes, dyslipidemia, GRS for longevity, and education. Horizontal lines indicate the range of the 95% confidence interval. The vertical dash lines represent the hazard ratio of 1. Only the results with significant interaction (P-value <0.05) are shown in this figure.

*Indicates significant interaction while controlling for false discovery rate (FDR), with two-sided statistical tests.

Cardiometabolic disorder	Events/ participants	Incidence	Hazard ratio (95% CI), Model 1	Hazard ratio (95% CI), Model 2
Cardiovascular disease	5092/107913	5.41		
AMED			0.94 (0.92-0.96)*	0.97 (0.95-0.99)*
AEDII			0.95 (0.93-0.97)*	0.97 (0.95-0.99)*
AHEI-2010			0.96 (0.94-0.98)*	0.98 (0.96-0.996)
HPDI			0.97 (0.95-0.99)*	0.99 (0.97-1.01)
Coronary heart disease	2981/111742	3.07		
AMED			0.96 (0.93-0.98)*	0.99 (0.96-1.01)
AEDII			0.93 (0.90-0.95)*	0.95 (0.92-0.97)*
AHEI-2010			0.99 (0.96-1.01)	1.01 (0.98-1.03)
HPDI			0.96 (0.94-0.99)*	0.99 (0.96-1.01)
Atrial fibrillation	432/112745	0.44		
AMED			0.94 (0.88-1.01)	0.97 (0.90-1.04)
AEDII			0.98 (0.92-1.05)	1.01 (0.94-1.08)
AHEI-2010			0.93 (0.87-0.99)*	0.95 (0.89-1.02)
HPDI			0.96 (0.90-1.03)	0.99 (0.92-1.06)
Heart failure	1065/114497	1.07		
AMED			0.89 (0.86-0.93)*	0.94 (0.90-0.98)*
AEDII			0.92 (0.88-0.96)*	0.96 (0.92-0.99)*
AHEI-2010			0.95 (0.91-0.99)*	0.98 (0.94-1.02)
HPDI			0.94 (0.90-0.98)*	0.97 (0.93-1.02)
Other cardiac problem	2627/113478	2.66		
AMED			0.93 (0.90-0.95)*	0.95 (0.92-0.98)*
AEDII			0.95 (0.93-0.98)*	0.97 (0.95-0.998)
AHEI-2010			0.94 (0.92-0.97)*	0.96 (0.93-0.99)*
HPDI			0.97 (0.94-0.99)*	0.99 (0.96-1.02)
Stroke	187/114314	0.19		
AMED			0.96 (0.87-1.07)	0.98 (0.89-1.09)
AEDII			0.93 (0.84-1.03)	0.95 (0.86-1.06)
AHEI-2010			0.93 (0.84-1.04)	0.94 (0.84-1.04)
HPDI			0.99 (0.89-1.09)	1.00 (0.90-1.12)
Peripheral vascular disease	605/114595	0.61		
AMED			0.92 (0.87-0.97)*	0.94 (0.88-0.99)*
AEDII			0.96 (0.91-1.02)	0.97 (0.92-1.03)
AHEI-2010			0.95 (0.89-0.99)*	0.96 (0.90-1.01)
HPDI			0.95 (0.90-1.01)	0.97 (0.91-1.03)
Hypertension	982/85292	1.31		
AMED			0.91 (0.87-0.95)*	0.95 (0.91-0.99)*
AEDII			0.97 (0.93-1.01)	1.01 (0.96-1.05)
AHEI-2010			0.90 (0.86-0.94)*	0.92 (0.88-0.97)*
HPDI			0.91 (0.87-0.95)*	0.94 (0.90-0.98)*
Diabetes	1584/110289	1.65		
AMED			0.89 (0.85-0.92)*	0.96 (0.93-0.99)*
AEDII			0.83 (0.80-0.86)*	0.90 (0.87-0.93)*
AHEI-2010			0.93 (0.89-0.96)*	0.97 (0.94-1.01)
HPDI			0.86 (0.83-0.89)*	0.92 (0.89-0.96)*

0.8 0.9 1.0 1.1 1.2 0.8 0.9 1.0 1.1 1.2

Figure S5. The association between dietary scores and the risk of cardiometabolic disorders among individuals by excluding those developed the corresponding disease in the first four years of follow-up

AEDII, Anti-Empirical Dietary Inflammatory Index; AHEI-2010, Alternate Healthy Eating Index-2010; AMED, Alternate Mediterranean Diet score; CI, confidence interval; HPDI, Healthful Plant-based Diet Index.

The incidence refers to the number of event cases per 1000 person-years. Cardiovascular disease includes coronary heart disease, heart failure, atrial fibrillation, other cardiac disease, stroke, and peripheral vascular disease. Cox proportional hazard regression models were used to examine associations of each of the four dietary scores with the risk of individual cardiometabolic disorders adjusted for potential confounding variables. Model 1 was adjusted for age, sex, and total energy intake; Model 2 was adjusted for Model 1 plus ethnicity, education, income, BMI, smoking, sleep, physical activity, and GRS for longevity. Dietary scores were analyzed as continuous variables (each quintile increment). The vertical dash lines represent the hazard ratio of 1. Squares represent the hazard ratios (black color for AMED, orange color for AEDII, blue color for AHEI-2010, and green color for HPDI). Horizontal lines indicate the range of the 95% confidence interval.

*Indicates a significant association through two-sided statistical tests while controlling for FDR.

Cancer	Events/ participants	Incidence	Hazard ratio (95% CI), Model 1	Hazard ratio (95% CI), Model 2
All cancers	3641/104981	3.96		
AMED			0.92 (0.90-0.95)*	0.95 (0.92-0.97)*
AEDII			0.99 (0.97-1.01)	1.00 (0.97-1.02)
AHEI-2010			0.95 (0.92-0.97)*	0.96 (0.94-0.98)*
HPDI			0.96 (0.94-0.98)*	0.97 (0.95-1.003)
Non-melanoma skin cancer	2258/112137	2.32		
AMED			1.00 (0.97-1.03)	1.00 (0.97-1.03)
AEDII			1.03 (1.01-1.06)	1.03 (1.001-1.06)
AHEI-2010			0.97 (0.94-1.01)	0.97 (0.94-1.001)
HPDI			0.99 (0.96-1.02)	0.99 (0.96-1.02)
Melanoma	295/113859	0.30		
AMED			1.01 (0.93-1.09)	1.01 (0.93-1.10)
AEDII			0.97 (0.90-1.06)	0.97 (0.89-1.05)
AHEI-2010			1.01 (0.93-1.10)	1.02 (0.94-1.11)
HPDI			1.00 (0.92-1.09)	1.00 (0.92-1.08)
Lung cancer	367/114823	0.37		
AMED			0.82 (0.76-0.89)*	0.88 (0.82-0.95)*
AEDII			1.00 (0.93-1.08)	0.99 (0.92-1.06)
AHEI-2010			0.85 (0.79-0.92)*	0.90 (0.84-0.97)*
HPDI			0.93 (0.87-1.01)	0.97 (0.90-1.04)
Stomach cancer	78/115031	0.08		
AMED			0.90 (0.77-1.06)	0.94 (0.80-1.10)
AEDII			0.87 (0.74-1.02)	0.86 (0.73-1.02)
AHEI-2010			0.90 (0.76-1.05)	0.89 (0.76-1.05)
HPDI			0.95 (0.81-1.12)	0.94 (0.80-1.10)
Oesophageal cancer	104/115011	0.10		
AMED			0.80 (0.70-0.92)*	0.86 (0.75-0.99)*
AEDII			1.00 (0.88-1.15)	1.05 (0.91-1.21)
AHEI-2010			0.88 (0.76-1.01)	0.92 (0.80-1.06)
HPDI			1.00 (0.87-1.15)	1.06 (0.92-1.22)
Colon cancer	472/114307	0.47		
AMED			0.95 (0.89-1.01)	0.97 (0.91-1.03)
AEDII			1.02 (0.95-1.08)	1.02 (0.96-1.09)
AHEI-2010			0.92 (0.86-0.98)*	0.94 (0.88-1.01)
HPDI			0.94 (0.88-1.01)	0.96 (0.90-1.02)
Prostate cancer	1308/51543	2.93		
AMED			1.00 (0.88-1.13)	1.01 (0.89-1.15)
AEDII			0.94 (0.83-1.06)	0.96 (0.84-1.08)
AHEI-2010			1.01 (0.89-1.14)	1.01 (0.89-1.14)
HPDI			0.89 (0.79-1.01)	0.91 (0.80-1.03)
Ovarian cancer	129/67630	0.22		
AMED			0.96 (0.92-1.00)	0.97 (0.93-1.02)
AEDII			0.98 (0.94-1.02)	0.99 (0.94-1.03)
AHEI-2010			0.93 (0.89-0.98)*	0.94 (0.90-0.99)*
HPDI			0.98 (0.94-1.03)	1.00 (0.95-1.04)
Breast cancer	1044/63711	1.88		
AMED			1.03 (0.99-1.07)	1.02 (0.98-1.06)
AEDII			1.03 (0.99-1.07)	1.02 (0.98-1.06)
AHEI-2010			0.99 (0.95-1.03)	0.98 (0.95-1.02)
HPDI			1.01 (0.97-1.05)	1.00 (0.96-1.04)
Other cancers	3178/106857	3.40		
AMED			0.92 (0.90-0.95)*	0.94 (0.92-0.97)*
AEDII			0.99 (0.96-1.01)	1.00 (0.97-1.02)
AHEI-2010			0.94 (0.92-0.97)*	0.96 (0.93-0.98)*
HPDI			0.95 (0.93-0.97)*	0.97 (0.94-0.99)*

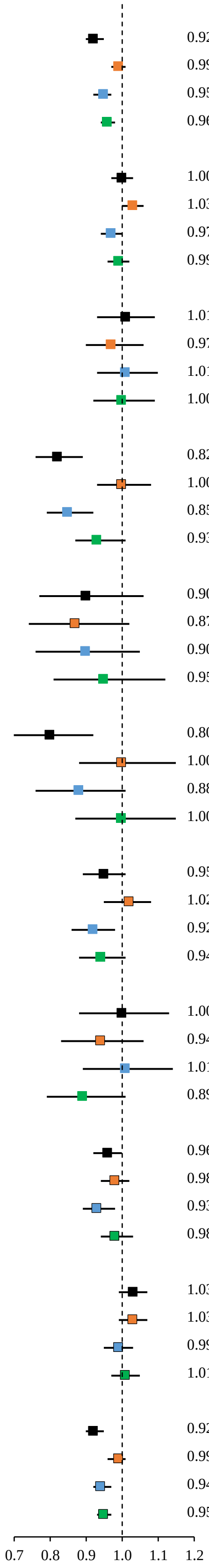

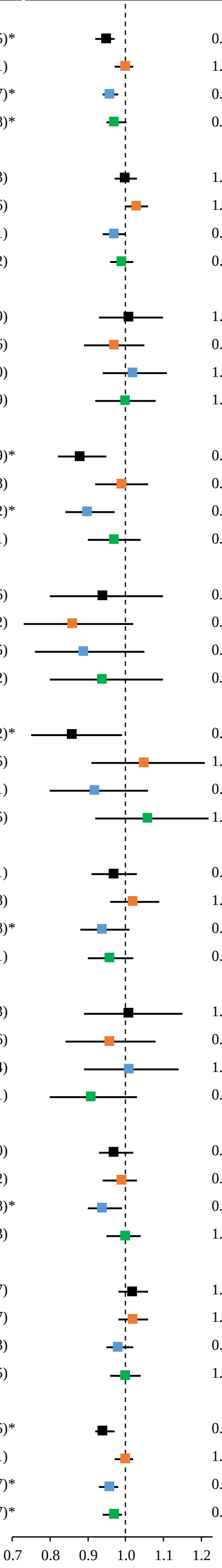

Figure S6. The association between dietary scores and the risk of all cancers and types of cancers among individuals by excluding those developed the corresponding disease in the first four years of follow-up

AEDII, Anti-Empirical Dietary Inflammatory Index; AHEI-2010, Alternate Healthy Eating Index-2010; AMED, Alternate Mediterranean Diet score; CI, confidence interval; HPDI, Healthful Plant-based Diet Index.

All cancers encompass any type of cancer except for non-melanoma skin cancer. The incidence refers to the number of event cases per 1000 person-years. Cox proportional hazard regression models were used to examine associations of each of the four dietary scores with the risk of individual cancers. Model 1 was adjusted for age, sex, and total energy intake; Model 2 was adjusted for Model 1 plus ethnicity, education, income, BMI, smoking, sleep, physical activity, and GRS for longevity (pack-years, age stopping smoking, and number of cigarettes currently smoked daily were further adjusted for lung cancer). Dietary scores were analyzed as continuous variables (each quintile increment). The analysis for ovarian cancer and breast cancer was conducted among women only while the analysis for prostate cancer was conducted among men only. The vertical dash lines represent the hazard ratio of 1. Squares represent the hazard ratios (black color for AMED, orange color for AEDII, blue color for AHEI-2010, and green color for HPDI). Horizontal lines indicate the range of the 95% confidence interval.

*Indicates a significant association through two-sided statistical tests while controlling for FDR.

Neurological/psychological disorder	Events/ participants	Incidence	Hazard ratio (95% CI), Model 1	Hazard ratio (95% CI), Model 2
Dementia	581/115001	0.58		
AMED			0.93 (0.87-0.98)*	0.93 (0.88-0.99)*
AEDII			0.96 (0.90-1.01)	0.96 (0.91-1.02)
AHEI-2010			0.93 (0.88-0.99)*	0.93 (0.88-0.99)*
HPDI			0.97 (0.91-1.03)	0.97 (0.92-1.03)
Parkinson's disease	291/114892	0.29		
AMED			0.92 (0.85-0.99)*	0.92 (0.85-0.99)*
AEDII			0.98 (0.90-1.06)	0.98 (0.90-1.07)
AHEI-2010			0.95 (0.87-1.03)	0.94 (0.87-1.02)
HPDI			0.98 (0.90-1.07)	0.98 (0.90-1.07)
Depression	282/108415	0.30		
AMED			0.89 (0.82-0.97)*	0.92 (0.85-1.01)
AEDII			0.93 (0.86-1.01)	0.96 (0.88-1.04)
AHEI-2010			0.99 (0.91-1.07)	1.00 (0.92-1.09)
HPDI			0.93 (0.86-1.01)	0.96 (0.88-1.05)
Anxiety	2060/112007	2.12		
AMED			0.90 (0.87-0.93)*	0.92 (0.89-0.95)*
AEDII			0.92 (0.89-0.95)*	0.94 (0.91-0.97)*
AHEI-2010			0.98 (0.95-1.01)	0.99 (0.96-1.02)
HPDI			0.95 (0.92-0.98)*	0.98 (0.94-1.01)
Schizophrenia	67/114748	0.07		
AMED			0.95 (0.81-1.13)	1.04 (0.88-1.24)
AEDII			0.89 (0.75-1.06)	0.93 (0.78-1.10)
AHEI-2010			0.91 (0.76-1.08)	0.95 (0.80-1.13)
HPDI			1.05 (0.88-1.25)	1.11 (0.93-1.33)
Multiple sclerosis	51/114647	0.05		
AMED			0.87 (0.71-1.06)	0.90 (0.74-1.10)
AEDII			0.98 (0.80-1.19)	0.98 (0.80-1.19)
AHEI-2010			0.86 (0.71-1.05)	0.89 (0.72-1.08)
HPDI			1.06 (0.87-1.30)	1.09 (0.89-1.34)
Alcohol use disorder	501/114485	0.50		
AMED			0.79 (0.74-0.85)*	0.84 (0.78-0.89)*
AEDII			1.17 (1.09-1.24)*	1.17 (1.10-1.25)*
AHEI-2010			0.72 (0.67-0.77)*	0.75 (0.70-0.80)*
HPDI			0.91 (0.86-0.97)*	0.94 (0.88-1.001)
Psychoactive substance abuse	95/114947	0.10		
AMED			0.82 (0.71-0.95)*	0.88 (0.76-1.02)
AEDII			1.18 (1.02-1.37)*	1.20 (1.04-1.39)*
AHEI-2010			0.76 (0.65-0.88)*	0.79 (0.68-0.92)*
HPDI			1.03 (0.89-1.20)	1.07 (0.93-1.24)
Migraine	433/111019	0.45		
AMED			0.98 (0.92-1.05)	0.99 (0.93-1.06)
AEDII			0.96 (0.89-1.02)	0.96 (0.89-1.02)
AHEI-2010			0.98 (0.91-1.05)	0.98 (0.92-1.05)
HPDI			0.97 (0.91-1.04)	0.98 (0.91-1.05)
Epilepsy	234/114177	0.24		
AMED			0.93 (0.85-1.02)	0.94 (0.86-1.03)
AEDII			0.99 (0.90-1.08)	0.98 (0.90-1.08)
AHEI-2010			0.94 (0.86-1.03)	0.96 (0.87-1.05)
HPDI			0.95 (0.86-1.04)	0.95 (0.87-1.05)

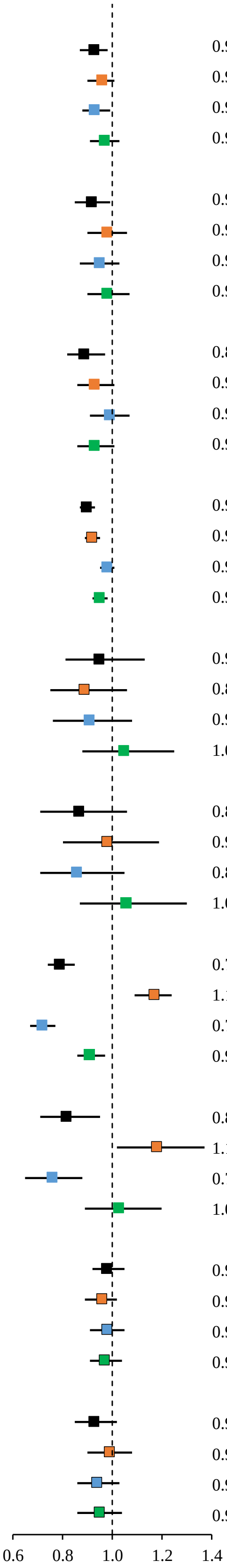

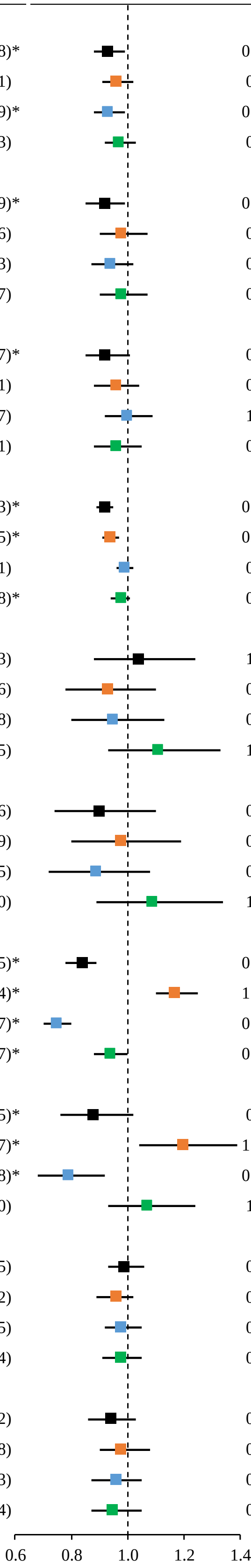

Figure S7. The association between dietary scores and the risk of neurological and psychological disorders among individuals by excluding those developed the corresponding disease in the first four years of follow-up

AEDII, Anti-Empirical Dietary Inflammatory Index; AHEI-2010, Alternate Healthy Eating Index-2010; AMED, Alternate Mediterranean Diet score; CI, confidence interval; HPDI, Healthful Plant-based Diet Index.

The incidence refers to the number of event cases per 1000 person-years. Cox proportional hazard regression models were used to examine associations of each of the four dietary scores with the risk of individual neurological/psychological disorders. Model 1 was adjusted for age, sex, and total energy intake; Model 2 was adjusted for Model 1 plus ethnicity, education, income, BMI, smoking, sleep, physical activity, and GRS for longevity. Dietary scores were analyzed as continuous variables (each quintile increment). The vertical dash lines represent the hazard ratio of 1. Squares represent the hazard ratios (black color for AMED, orange color for AEDII, blue color for AHEI-2010, and green color for HPDI). Horizontal lines indicate the range of the 95% confidence interval.

*Indicates a significant association through two-sided statistical tests while controlling for FDR.

Digestive disorder	Events/ participants	Incidence	Hazard ratio (95% CI), Model 1	Hazard ratio (95% CI), Model 2
Dyspepsia	5622/98701	6.54		
AMED			0.94 (0.93-0.96)*	0.96 (0.94-0.98)*
AEDII			0.93 (0.91-0.94)*	0.94 (0.92-0.96)*
AHEI-2010			0.96 (0.95-0.98)*	0.97 (0.95-0.99)*
HPDI			0.94 (0.92-0.96)*	0.96 (0.94-0.98)*
Treated constipation	2238/112743	2.28		
AMED			0.94 (0.92-0.97)*	0.96 (0.93-0.99)*
AEDII			0.96 (0.93-0.98)*	0.96 (0.94-0.99)*
AHEI-2010			0.96 (0.93-0.99)*	0.97 (0.94-0.99)*
HPDI			0.96 (0.94-0.99)*	0.98 (0.95-1.01)
Diverticular disease	4898/108109	5.21		
AMED			0.93 (0.91-0.95)*	0.96 (0.94-0.97)*
AEDII			0.96 (0.94-0.98)*	0.97 (0.95-0.99)*
AHEI-2010			0.93 (0.92-0.95)*	0.95 (0.93-0.97)*
HPDI			0.93 (0.91-0.95)*	0.95 (0.93-0.97)*
Inflammatory bowel disease	284/113835	0.29		
AMED			0.95 (0.87-1.03)	0.96 (0.88-1.05)
AEDII			0.94 (0.87-1.02)	0.95 (0.87-1.03)
AHEI-2010			0.96 (0.88-1.04)	0.96 (0.88-1.04)
HPDI			0.97 (0.89-1.05)	0.98 (0.90-1.07)
Irritable bowel syndrome	757/111770	0.78		
AMED			0.90 (0.86-0.95)*	0.92 (0.88-0.97)*
AEDII			0.92 (0.87-0.97)*	0.93 (0.88-0.98)*
AHEI-2010			0.94 (0.89-0.99)*	0.96 (0.91-1.004)
HPDI			0.91 (0.86-0.96)*	0.93 (0.88-0.98)*
Chronic liver disease	163/114465	0.16		
AMED			0.81 (0.72-0.90)*	0.88 (0.78-0.98)*
AEDII			0.82 (0.73-0.92)*	0.87 (0.77-0.97)*
AHEI-2010			0.89 (0.79-0.99)*	0.93 (0.84-1.04)
HPDI			0.84 (0.75-0.93)*	0.90 (0.80-0.99)*

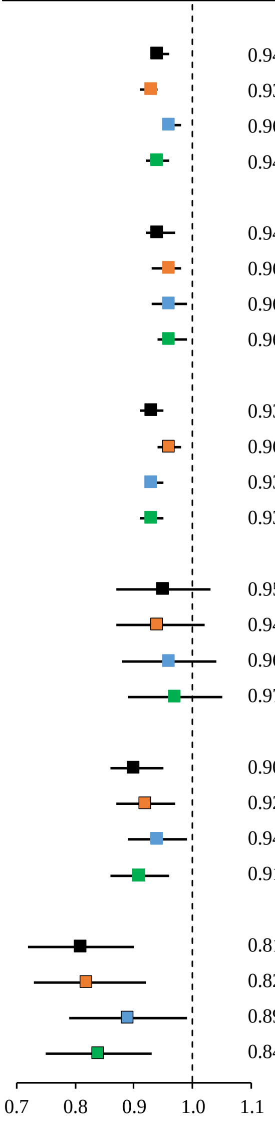

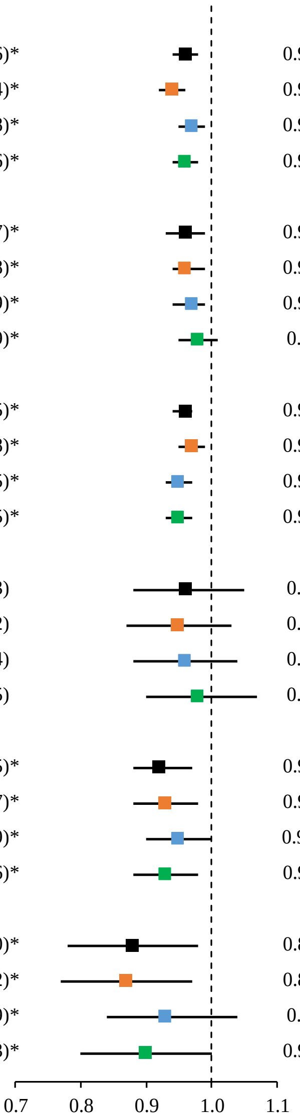

Figure S8. The association between dietary scores and the risk of digestive disorders among individuals by excluding those developed the corresponding disease in the first four years of follow-up

AEDII, Anti-Empirical Dietary Inflammatory Index; AHEI-2010, Alternate Healthy Eating Index-2010; AMED, Alternate Mediterranean Diet score; CI, confidence interval; HPDI, Healthful Plant-based Diet Index.

The incidence refers to the number of event cases per 1000 person-years. Cox proportional hazard regression models were used to examine associations of each of the four dietary scores with the risk of individual digestive disorders. Model 1 was adjusted for age, sex, and total energy intake; Model 2 was adjusted for Model 1 plus ethnicity, education, income, BMI, smoking, sleep, physical activity, and GRS for longevity. Dietary scores were analyzed as continuous variables (each quintile increment). The vertical dash lines represent the hazard ratio of 1. Squares represent the hazard ratios (black color for AMED, orange color for AEDII, blue color for AHEI-2010, and green color for HPDI). Horizontal lines indicate the range of the 95% confidence interval.

*Indicates a significant association through two-sided statistical tests while controlling for FDR.

Disease	Events/ participants	Incidence	Hazard ratio (95% CI), Model 1	Hazard ratio (95% CI), Model 2
COPD	1224/113299	1.24		
AMED			0.83 (0.80-0.86)*	0.89 (0.85-0.93)*
AEDII			0.93 (0.89-0.97)*	0.92 (0.89-0.96)*
AHEI-2010			0.86 (0.82-0.89)*	0.90 (0.87-0.94)*
HPDI			0.89 (0.85-0.92)*	0.92 (0.88-0.96)*
Asthma	975/99613	1.12		
AMED			0.94 (0.90-0.98)*	0.97 (0.93-1.01)
AEDII			0.96 (0.91-1.00)	0.98 (0.94-1.03)
AHEI-2010			0.95 (0.91-1.00)	0.96 (0.92-1.01)
HPDI			0.96 (0.92-1.01)	0.99 (0.94-1.03)
Bronchiectasis	461/114621	0.46		
AMED			0.86 (0.81-0.92)*	0.86 (0.80-0.92)*
AEDII			1.01 (0.95-1.08)	0.99 (0.93-1.06)
AHEI-2010			0.87 (0.81-0.93)*	0.87 (0.81-0.92)*
HPDI			0.93 (0.87-0.99)*	0.92 (0.86-0.99)*
Osteoporosis	3732/102961	4.17		
AMED			0.95 (0.93-0.97)*	0.98 (0.96-1.01)
AEDII			0.97 (0.95-0.99)*	1.00 (0.98-1.02)
AHEI-2010			0.93 (0.91-0.95)*	0.95 (0.93-0.97)*
HPDI			0.96 (0.94-0.99)*	0.99 (0.97-1.02)
Fracture	517/114303	0.52		
AMED			0.98 (0.92-1.04)	0.97 (0.91-1.03)
AEDII			1.04 (0.97-1.10)	1.02 (0.95-1.08)
AHEI-2010			0.99 (0.93-1.05)	0.99 (0.93-1.05)
HPDI			1.00 (0.94-1.07)	0.99 (0.93-1.06)
Chronic kidney disease	2946/113427	2.98		
AMED			0.87 (0.84-0.89)*	0.91 (0.89-0.93)*
AEDII			0.86 (0.84-0.89)*	0.90 (0.88-0.93)*
AHEI-2010			0.92 (0.90-0.95)*	0.95 (0.92-0.97)*
HPDI			0.88 (0.86-0.90)*	0.92 (0.89-0.94)*
Thyroid disorders	1394/107546	1.49		
AMED			0.93 (0.90-0.97)*	0.96 (0.93-1.003)
AEDII			0.91 (0.88-0.95)*	0.94 (0.90-0.97)*
AHEI-2010			1.00 (0.97-1.04)	1.02 (0.98-1.06)
HPDI			0.96 (0.92-1.00)	0.99 (0.95-1.03)
Psoriasis/eczema	822/110128	0.86		
AMED			0.91 (0.87-0.96)*	0.93 (0.88-0.98)*
AEDII			0.98 (0.93-1.02)	0.98 (0.94-1.03)
AHEI-2010			0.92 (0.88-0.97)*	0.93 (0.89-0.98)*
HPDI			0.96 (0.91-1.01)	0.97 (0.92-1.02)
Meniere’s disease	57/114741	0.06		
AMED			1.11 (0.92-1.34)	1.16 (0.96-1.40)
AEDII			0.98 (0.81-1.18)	1.01 (0.84-1.22)
AHEI-2010			1.06 (0.88-1.28)	1.08 (0.90-1.31)
HPDI			0.88 (0.73-1.06)	0.90 (0.75-1.09)
Prostate disorders	2145/48142	5.15		
AMED			0.99 (0.96-1.02)	0.99 (0.96-1.02)
AEDII			0.97 (0.94-1.00)	0.98 (0.95-1.01)
AHEI-2010			0.99 (0.96-1.02)	0.99 (0.96-1.02)
HPDI			0.97 (0.94-1.00)	0.97 (0.94-1.001)
Glaucoma	1137/113357	1.15		
AMED			0.99 (0.95-1.03)	0.98 (0.94-1.03)
AEDII			0.97 (0.93-1.01)	0.97 (0.93-1.01)
AHEI-2010			1.03 (0.99-1.08)	1.03 (0.98-1.07)
HPDI			1.02 (0.97-1.06)	1.01 (0.97-1.06)
Cataract	5176/109307	5.44		
AMED			0.97 (0.95-0.99)*	0.98 (0.96-0.998)
AEDII			0.98 (0.96-1.00)	0.99 (0.97-1.01)
AHEI-2010			0.98 (0.96-1.00)	0.98 (0.96-1.002)
HPDI			0.97 (0.95-0.99)*	0.98 (0.96-1.002)
AMD	923/114471	0.93		
AMED			0.98 (0.94-1.03)	0.99 (0.95-1.04)
AEDII			1.01 (0.96-1.06)	1.01 (0.97-1.06)
AHEI-2010			0.99 (0.94-1.04)	0.99 (0.95-1.04)
HPDI			1.01 (0.96-1.06)	1.01 (0.97-1.06)
Pernicious anaemia	85/114781	0.09		
AMED			0.77 (0.66-0.90)*	0.80 (0.69-0.94)*
AEDII			0.96 (0.82-1.12)	0.99 (0.85-1.15)
AHEI-2010			0.83 (0.71-0.97)*	0.85 (0.73-0.99)*
HPDI			0.80 (0.68-0.93)*	0.82 (0.70-0.96)*

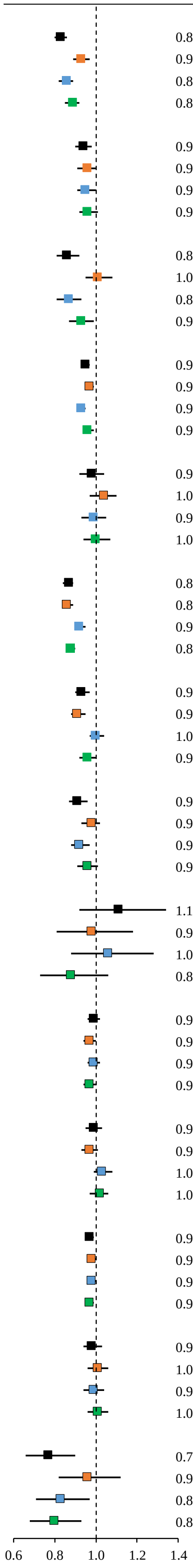

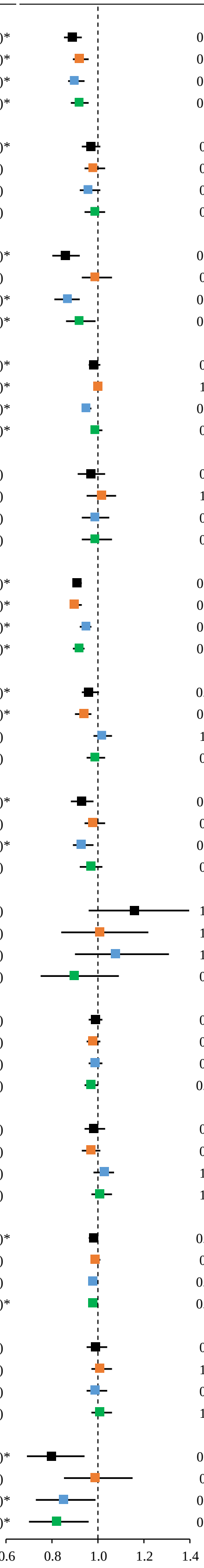

Figure S9. The association between dietary scores and the risk of other chronic diseases among individuals by excluding those developed the corresponding disease in the first four years of follow-up

AEDII, Anti-Empirical Dietary Inflammatory Index; AHEI-2010, Alternate Healthy Eating Index-2010; AMED, Alternate Mediterranean Diet score; AMD, age related macular degeneration; CI, confidence interval; COPD, chronic obstructive pulmonary disease; HPDI, Healthful Plant-based Diet Index.

The incidence refers to the number of event cases per 1000 person-years. Cox proportional hazard regression models were used to examine associations of each of the four dietary scores with the risk of individual other chronic diseases. Model 1 was adjusted for age, sex, and total energy intake; Model 2 was adjusted for Model 1 plus ethnicity, education, income, BMI, smoking, sleep, physical activity, and GRS for longevity. Dietary scores were analyzed as continuous variables (each quintile increment). The analysis for prostate disorders was conducted among men only. The vertical dash lines represent the hazard ratio of 1. Squares represent the hazard ratios (black color for AMED, orange color for AEDII, blue color for AHEI-2010, and green color for HPDI). Horizontal lines indicate the range of the 95% confidence interval.

*Indicates a significant association through two-sided statistical tests while controlling for FDR.

Cardiometabolic disorder	Events/ participants	Incidence	Hazard ratio (95% CI), Model 1	Hazard ratio (95% CI), Model 2
Cardiovascular disease	5354/69420	8.96		
AMED			0.91 (0.89-0.92)*	0.93 (0.91-0.95)*
AEDII			0.95 (0.93-0.97)*	0.97 (0.95-0.99)*
AHEI-2010			0.94 (0.92-0.95)*	0.95 (0.93-0.97)*
HPDI			0.95 (0.93-0.97)*	0.97 (0.95-0.99)*
Coronary heart disease	2850/70578	4.70		
AMED			0.91 (0.88-0.93)*	0.94 (0.91-0.96)*
AEDII			0.93 (0.90-0.95)*	0.95 (0.93-0.98)*
AHEI-2010			0.94 (0.92-0.97)*	0.96 (0.94-0.99)*
HPDI			0.94 (0.92-0.97)*	0.97 (0.94-0.99)*
Atrial fibrillation	1095/70916	1.80		
AMED			0.92 (0.88-0.96)*	0.95 (0.91-0.99)*
AEDII			0.96 (0.92-1.00)	0.99 (0.95-1.03)
AHEI-2010			0.94 (0.90-0.98)*	0.96 (0.92-1.01)
HPDI			0.98 (0.94-1.02)	1.01 (0.96-1.05)
Heart failure	884/71419	1.44		
AMED			0.85 (0.81-0.89)*	0.89 (0.85-0.94)*
AEDII			0.91 (0.86-0.95)*	0.95 (0.90-0.99)*
AHEI-2010			0.93 (0.88-0.97)*	0.96 (0.93-0.99)*
HPDI			0.94 (0.90-0.99)*	0.98 (0.94-1.03)
Other cardiac problem	2175/71099	3.56		
AMED			0.90 (0.87-0.93)*	0.92 (0.89-0.95)*
AEDII			0.95 (0.92-0.98)*	0.96 (0.94-0.98)*
AHEI-2010			0.93 (0.90-0.96)*	0.94 (0.92-0.97)*
HPDI			0.95 (0.92-0.98)*	0.97 (0.95-1.01)
Stroke	442/71337	0.72		
AMED			0.86 (0.81-0.92)*	0.88 (0.82-0.94)*
AEDII			0.98 (0.91-1.05)	0.99 (0.92-1.06)
AHEI-2010			0.89 (0.84-0.96)*	0.90 (0.84-0.97)*
HPDI			0.95 (0.88-1.01)	0.96 (0.90-1.03)
Peripheral vascular disease	523/71359	0.85		
AMED			0.89 (0.84-0.95)*	0.91 (0.86-0.97)*
AEDII			0.96 (0.90-1.02)	0.96 (0.90-1.02)
AHEI-2010			0.94 (0.88-1.00)	0.95 (0.90-1.01)
HPDI			0.96 (0.90-1.02)	0.97 (0.91-1.04)
Hypertension	2333/55185	4.90		
AMED			0.89 (0.86-0.91)*	0.93 (0.90-0.96)*
AEDII			0.92 (0.89-0.95)*	0.96 (0.93-0.98)*
AHEI-2010			0.90 (0.87-0.92)*	0.92 (0.90-0.95)*
HPDI			0.90 (0.87-0.93)*	0.94 (0.91-0.97)*
Diabetes	1474/69147	2.48		
AMED			0.87 (0.83-0.90)*	0.95 (0.91-0.98)*
AEDII			0.81 (0.78-0.84)*	0.88 (0.85-0.91)*
AHEI-2010			0.93 (0.90-0.96)*	0.98 (0.95-1.02)
HPDI			0.85 (0.82-0.88)*	0.92 (0.89-0.95)*

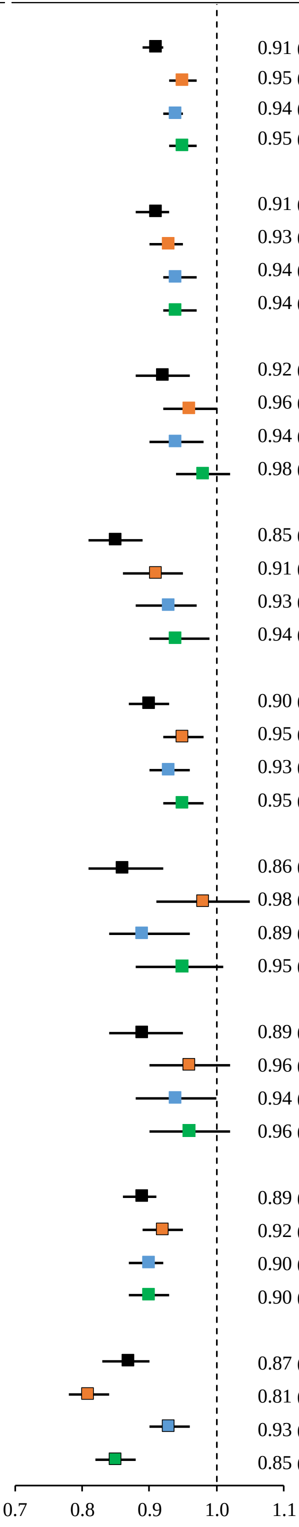

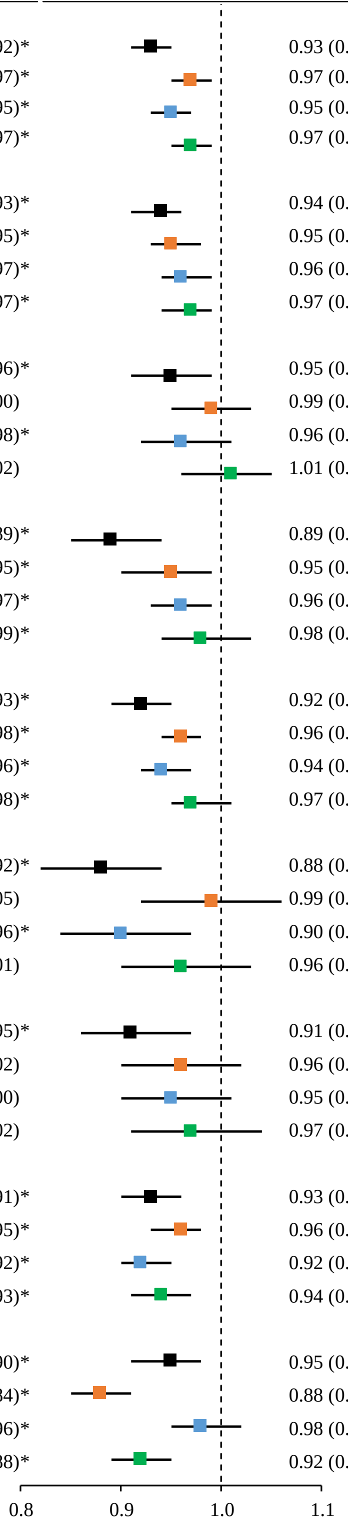

Figure S10. The association between dietary scores and the risk of cardiometabolic disorders among individuals with three or more dietary assessments

AEDII, Anti-Empirical Dietary Inflammatory Index; AHEI-2010, Alternate Healthy Eating Index-2010; AMED, Alternate Mediterranean Diet score; CI, confidence interval; HPDI, Healthful Plant-based Diet Index.

The incidence refers to the number of event cases per 1000 person-years. Cardiovascular disease includes coronary heart disease, heart failure, atrial fibrillation, other cardiac disease, stroke, and peripheral vascular disease. Cox proportional hazard regression models were used to examine associations of each of the four dietary scores with the risk of individual cardiometabolic disorders adjusted for potential confounding variables. Model 1 was adjusted for age, sex, and total energy intake; Model 2 was adjusted for Model 1 plus ethnicity, education, income, BMI, smoking, sleep, physical activity, and GRS for longevity. Dietary scores were analyzed as continuous variables (each quintile increment). The vertical dash lines represent the hazard ratio of 1. Squares represent the hazard ratios (black color for AMED, orange color for AEDII, blue color for AHEI-2010, and green color for HPDI). Horizontal lines indicate the range of the 95% confidence interval.

*Indicates a significant association through two-sided statistical tests while controlling for FDR.

Cancer	Events/ participants	Incidence	Hazard ratio (95% CI), Model 1	Hazard ratio (95% CI), Model 2
All cancers	3791/66671	6.60		
AMED			0.91 (0.89-0.93)*	0.93 (0.91-0.95)*
AEDII			0.99 (0.97-1.01)	1.00 (0.97-1.02)
AHEI-2010			0.93 (0.91-0.95)*	0.95 (0.92-0.97)*
HPDI			0.94 (0.92-0.96)*	0.95 (0.93-0.97)*
Non-melanoma skin cancer	2219/70475	3.66		
AMED			0.99 (0.96-1.02)	0.98 (0.95-1.01)
AEDII			1.01 (0.98-1.04)	1.01 (0.98-1.04)
AHEI-2010			0.97 (0.94-1.00)	0.96 (0.93-0.99)*
HPDI			0.97 (0.94-1.00)	0.96 (0.94-0.99)*
Melanoma	295/70835	0.48		
AMED			0.99 (0.91-1.07)	0.98 (0.91-1.07)
AEDII			1.01 (0.93-1.10)	1.01 (0.93-1.10)
AHEI-2010			0.99 (0.91-1.08)	0.99 (0.91-1.08)
HPDI			0.95 (0.88-1.04)	0.95 (0.87-1.03)
Lung cancer	375/71488	0.61		
AMED			0.79 (0.73-0.85)*	0.89 (0.83-0.96)*
AEDII			1.04 (0.97-1.12)	1.03 (0.96-1.10)
AHEI-2010			0.87 (0.81-0.93)*	0.95 (0.88-1.01)
HPDI			0.94 (0.87-1.01)	0.99 (0.92-1.06)
Stomach cancer	98/71498	0.16		
AMED			0.89 (0.78-1.03)	0.91 (0.79-1.06)
AEDII			0.95 (0.82-1.09)	0.96 (0.83-1.11)
AHEI-2010			0.97 (0.84-1.11)	0.99 (0.86-1.14)
HPDI			0.96 (0.83-1.11)	0.98 (0.85-1.13)
Oesophageal cancer	120/71506	0.20		
AMED			0.85 (0.74-0.96)*	0.90 (0.79-1.02)
AEDII			0.96 (0.84-1.09)	1.00 (0.88-1.14)
AHEI-2010			0.96 (0.85-1.10)	1.01 (0.89-1.15)
HPDI			0.99 (0.87-1.12)	1.04 (0.92-1.19)
Colon cancer	503/71206	0.82		
AMED			0.93 (0.87-0.99)*	0.95 (0.90-1.02)
AEDII			1.01 (0.95-1.07)	1.02 (0.96-1.09)
AHEI-2010			0.92 (0.86-0.98)*	0.97 (0.92-1.02)
HPDI			0.89 (0.83-0.94)*	0.91 (0.85-0.97)*
Prostate cancer	2080/52315	4.60		
AMED			1.01 (0.98-1.04)	1.00 (0.97-1.03)
AEDII			1.01 (0.98-1.05)	1.01 (0.98-1.04)
AHEI-2010			0.98 (0.95-1.01)	0.98 (0.94-1.01)
HPDI			1.01 (0.98-1.04)	1.00 (0.97-1.03)
Ovarian cancer	137/42271	0.38		
AMED			0.90 (0.80-1.01)	0.92 (0.81-1.04)
AEDII			0.88 (0.78-1.00)	0.91 (0.81-1.03)
AHEI-2010			0.95 (0.84-1.07)	0.95 (0.85-1.08)
HPDI			0.84 (0.74-0.94)*	0.86 (0.76-0.97)*
Breast cancer	1170/40294	3.36		
AMED			0.97 (0.93-1.01)	0.98 (0.94-1.02)
AEDII			0.99 (0.95-1.03)	0.99 (0.95-1.03)
AHEI-2010			0.96 (0.92-1.00)	0.97 (0.93-1.01)
HPDI			0.99 (0.95-1.03)	1.00 (0.96-1.05)
Other cancers	3242/67588	5.57		
AMED			0.90 (0.88-0.93)*	0.92 (0.90-0.95)*
AEDII			0.99 (0.96-1.01)	1.00 (0.97-1.02)
AHEI-2010			0.92 (0.90-0.95)*	0.94 (0.91-0.96)*
HPDI			0.93 (0.91-0.96)*	0.95 (0.93-0.97)*

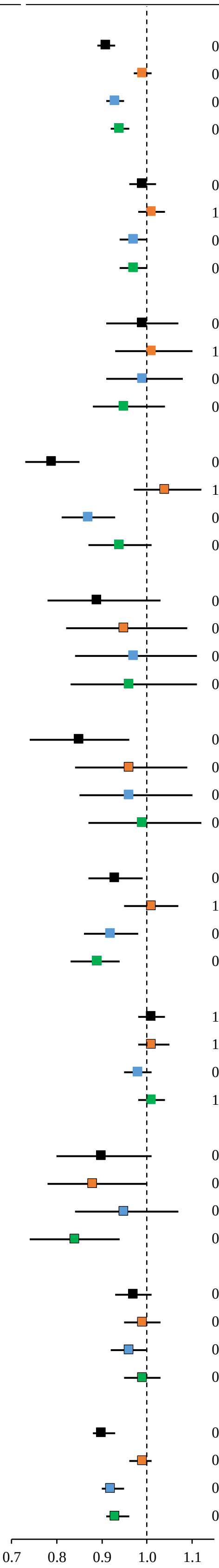

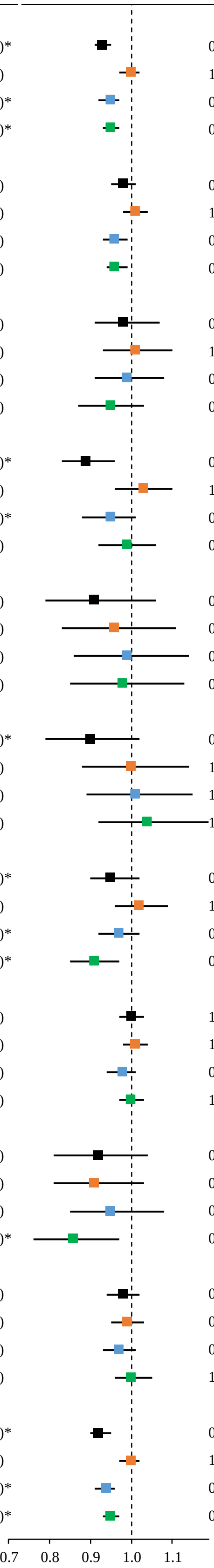

Figure S11. The association between dietary scores and the risk of all cancers and types of cancers among individuals with three or more dietary assessments

AEDII, Anti-Empirical Dietary Inflammatory Index; AHEI-2010, Alternate Healthy Eating Index-2010; AMED, Alternate Mediterranean Diet score; CI, confidence interval; HPDI, Healthful Plant-based Diet Index.

The incidence refers to the number of event cases per 1000 person-years. All cancers encompass any type of cancer except for non-melanoma skin cancer. Cox proportional hazard regression models were used to examine associations of each of the four dietary scores with the risk of individual cancers. Model 1 was adjusted for age, sex, and total energy intake; Model 2 was adjusted for Model 1 plus ethnicity, education, income, BMI, smoking, sleep, physical activity, and GRS for longevity (pack-years, age stopping smoking, and number of cigarettes currently smoked daily were further adjusted for lung cancer). Dietary scores were analyzed as continuous variables (each quintile increment). The analysis for ovarian cancer and breast cancer was conducted among women only while the analysis for prostate cancer was conducted among men only. The vertical dash lines represent the hazard ratio of 1. Squares represent the hazard ratios (black color for AMED, orange color for AEDII, blue color for AHEI-2010, and green color for HPDI). Horizontal lines indicate the range of the 95% confidence interval.

*Indicates a significant association through two-sided statistical tests while controlling for FDR.

Neurological/psychological disorder	Events/ participants	Incidence	Hazard ratio (95% CI), Model 1	Hazard ratio (95% CI), Model 2
Dementia	401/71505	0.65		
AMED			0.93 (0.87-1.00)	0.95 (0.88-1.02)
AEDII			0.97 (0.91-1.04)	0.98 (0.92-1.06)
AHEI-2010			0.94 (0.88-1.01)	0.95 (0.88-1.02)
HPDI			0.97 (0.90-1.04)	0.98 (0.91-1.05)
Parkinson's disease	234/71436	0.38		
AMED			0.87 (0.80-0.96)*	0.87 (0.80-0.96)*
AEDII			0.98 (0.90-1.08)	0.99 (0.90-1.09)
AHEI-2010			0.92 (0.84-1.01)	0.92 (0.83-1.01)
HPDI			0.98 (0.89-1.07)	0.99 (0.90-1.08)
Depression	541/67909	0.93		
AMED			0.88 (0.83-0.94)*	0.92 (0.87-0.98)*
AEDII			0.90 (0.85-0.96)*	0.93 (0.87-0.99)*
AHEI-2010			0.95 (0.89-1.01)	0.96 (0.91-1.02)
HPDI			0.95 (0.89-1.01)	0.98 (0.92-1.04)
Anxiety	1727/70143	2.86		
AMED			0.89 (0.86-0.92)*	0.91 (0.88-0.95)*
AEDII			0.92 (0.89-0.95)*	0.94 (0.91-0.97)*
AHEI-2010			0.96 (0.92-0.99)*	0.97 (0.94-0.99)*
HPDI			0.94 (0.91-0.97)*	0.97 (0.94-0.99)*
Schizophrenia	71/71316	0.12		
AMED			0.94 (0.79-1.10)	1.03 (0.87-1.22)
AEDII			0.97 (0.82-1.14)	1.02 (0.86-1.21)
AHEI-2010			0.89 (0.75-1.05)	0.94 (0.79-1.11)
HPDI			1.02 (0.86-1.21)	1.11 (0.93-1.31)
Multiple sclerosis	61/71244	0.10		
AMED			0.93 (0.77-1.11)	0.94 (0.78-1.12)
AEDII			0.88 (0.74-1.06)	0.87 (0.72-1.04)
AHEI-2010			0.87 (0.73-1.05)	0.88 (0.73-1.06)
HPDI			1.00 (0.84-1.20)	1.01 (0.84-1.22)
Alcohol use disorder	452/71271	0.74		
AMED			0.77 (0.72-0.82)*	0.81 (0.76-0.87)*
AEDII			1.19 (1.12-1.28)*	1.21 (1.13-1.29)*
AHEI-2010			0.69 (0.64-0.74)*	0.72 (0.67-0.78)*
HPDI			0.90 (0.84-0.96)*	0.93 (0.87-0.99)*
Psychoactive substance abuse	82/71448	0.13		
AMED			0.81 (0.69-0.94)*	0.87 (0.74-1.01)
AEDII			1.15 (0.98-1.34)	1.16 (0.99-1.36)
AHEI-2010			0.71 (0.60-0.84)*	0.75 (0.63-0.88)*
HPDI			0.98 (0.84-1.15)	1.03 (0.88-1.20)
Migraine	394/69154	0.66		
AMED			0.98 (0.92-1.06)	0.99 (0.92-1.07)
AEDII			0.98 (0.91-1.05)	0.98 (0.91-1.05)
AHEI-2010			0.97 (0.90-1.04)	0.97 (0.90-1.04)
HPDI			0.96 (0.90-1.03)	0.96 (0.90-1.04)
Epilepsy	204/71021	0.33		
AMED			0.88 (0.80-0.97)*	0.89 (0.81-0.98)*
AEDII			0.91 (0.82-1.00)	0.99 (0.92-1.06)
AHEI-2010			0.92 (0.83-1.02)	0.93 (0.85-1.03)
HPDI			0.91 (0.82-1.00)	0.92 (0.83-1.01)

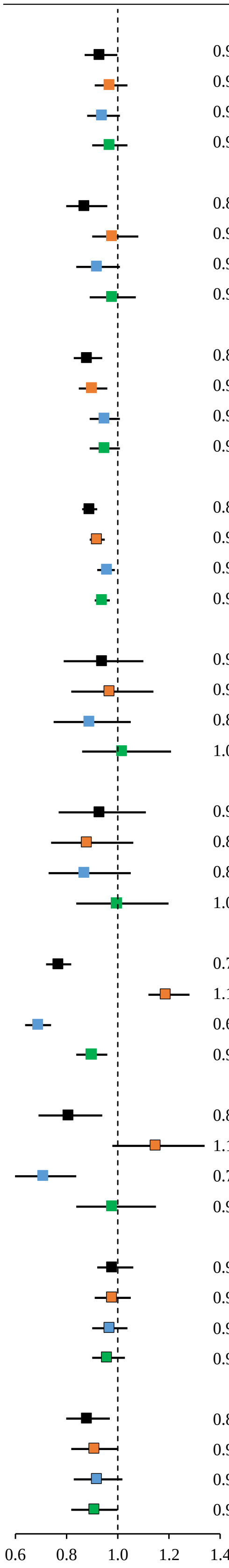

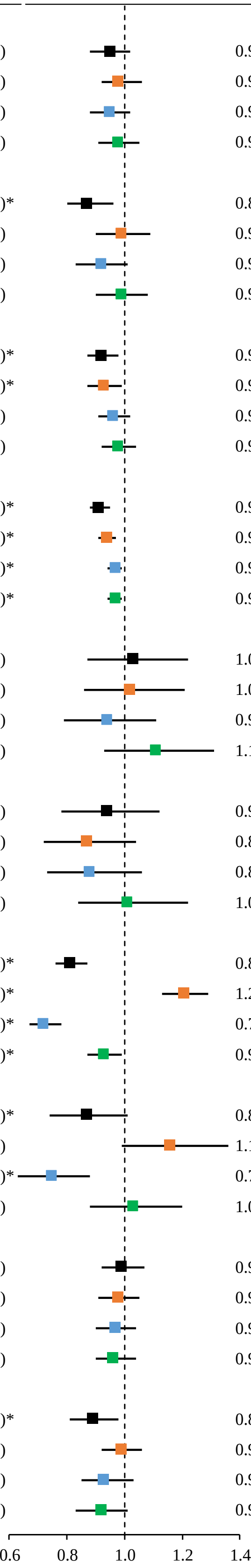

Figure S12. The association between dietary scores and the risk of neurological and psychological disorders among individuals with three or more dietary assessments

AEDII, Anti-Empirical Dietary Inflammatory Index; AHEI-2010, Alternate Healthy Eating Index-2010; AMED, Alternate Mediterranean Diet score; CI, confidence interval; HPDI, Healthful Plant-based Diet Index.

The incidence refers to the number of event cases per 1000 person-years. Cox proportional hazard regression models were used to examine associations of each of the four dietary scores with the risk of individual neurological/psychological disorders. Model 1 was adjusted for age, sex, and total energy intake; Model 2 was adjusted for Model 1 plus ethnicity, education, income, BMI, smoking, sleep, physical activity, and GRS for longevity. Dietary scores were analyzed as continuous variables (each quintile increment). The vertical dash lines represent the hazard ratio of 1. Squares represent the hazard ratios (black color for AMED, orange color for AEDII, blue color for AHEI-2010, and green color for HPDI). Horizontal lines indicate the range of the 95% confidence interval.

*Indicates a significant association through two-sided statistical tests while controlling for FDR.

Digestive disorder	Events/ participants	Incidence	Hazard ratio (95% CI), Model 1	Hazard ratio (95% CI), Model 2
Dyspepsia	5931/64022	10.77		
AMED			0.93 (0.91-0.95)*	0.95 (0.93-0.97)*
AEDII			0.93 (0.92-0.95)*	0.95 (0.93-0.97)*
AHEI-2010			0.94 (0.93-0.96)*	0.95 (0.94-0.97)*
HPDI			0.94 (0.92-0.95)*	0.95 (0.94-0.97)*
Treated constipation	2041/70752	3.36		
AMED			0.91 (0.88-0.94)*	0.92 (0.89-0.95)*
AEDII			0.92 (0.90-0.95)*	0.94 (0.91-0.97)*
AHEI-2010			0.94 (0.91-0.97)*	0.95 (0.92-0.98)*
HPDI			0.95 (0.92-0.98)*	0.96 (0.93-0.99)*
Diverticular disease	4940/69030	8.32		
AMED			0.91 (0.89-0.93)*	0.94 (0.92-0.96)*
AEDII			0.95 (0.94-0.97)*	0.97 (0.95-0.99)*
AHEI-2010			0.92 (0.90-0.93)*	0.93 (0.91-0.95)*
HPDI			0.92 (0.90-0.94)*	0.94 (0.92-0.96)*
Inflammatory bowel disease	268/70809	0.44		
AMED			0.96 (0.88-1.04)	0.99 (0.91-1.08)
AEDII			0.95 (0.87-1.04)	0.96 (0.88-1.05)
AHEI-2010			0.90 (0.82-0.98)*	0.96 (0.90-1.02)
HPDI			0.92 (0.85-1.01)	0.94 (0.86-1.03)
Irritable bowel syndrome	658/69692	1.10		
AMED			0.90 (0.85-0.95)*	0.93 (0.88-0.98)*
AEDII			0.89 (0.85-0.94)*	0.91 (0.86-0.96)*
AHEI-2010			0.92 (0.87-0.97)*	0.93 (0.88-0.99)*
HPDI			0.89 (0.84-0.94)*	0.91 (0.86-0.97)*
Chronic liver disease	123/71154	0.20		
AMED			0.83 (0.73-0.94)*	0.82 (0.75-0.90)*
AEDII			0.86 (0.76-0.98)*	0.84 (0.77-0.92)*
AHEI-2010			0.84 (0.74-0.95)*	0.86 (0.78-0.95)*
HPDI			0.80 (0.71-0.92)*	0.86 (0.79-0.94)*

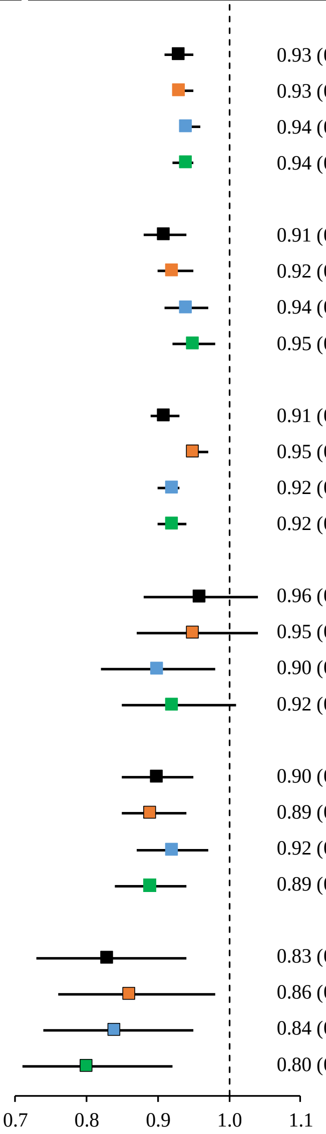

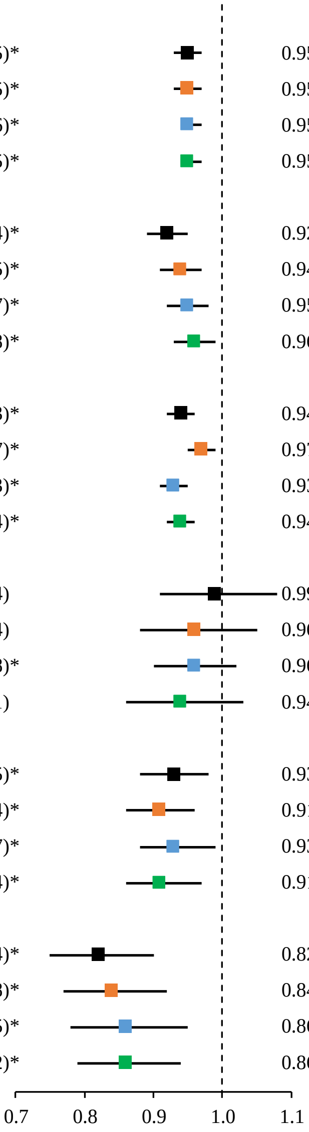

Figure S13. The association between dietary scores and the risk of digestive disorders among individuals with three or more dietary assessments

AEDII, Anti-Empirical Dietary Inflammatory Index; AHEI-2010, Alternate Healthy Eating Index-2010; AMED, Alternate Mediterranean Diet score; CI, confidence interval; HPDI, Healthful Plant-based Diet Index.

The incidence refers to the number of event cases per 1000 person-years. Cox proportional hazard regression models were used to examine associations of each of the four dietary scores with the risk of individual digestive disorders. Model 1 was adjusted for age, sex, and total energy intake; Model 2 was adjusted for Model 1 plus ethnicity, education, income, BMI, smoking, sleep, physical activity, and GRS for longevity. Dietary scores were analyzed as continuous variables (each quintile increment). The vertical dash lines represent the hazard ratio of 1. Squares represent the hazard ratios (black color for AMED, orange color for AEDII, blue color for AHEI-2010, and green color for HPDI). Horizontal lines indicate the range of the 95% confidence interval.

*Indicates a significant association through two-sided statistical tests while controlling for FDR.

Disease	Events/ participants	Incidence	Hazard ratio (95% CI), Model 1	Hazard ratio (95% CI), Model 2
COPD	1073/70725	1.76		
AMED			0.81 (0.77-0.84)*	0.87 (0.83-0.91)*
AEDII			0.93 (0.89-0.97)*	0.92 (0.88-0.96)*
AHEI-2010			0.85 (0.82-0.89)*	0.90 (0.86-0.94)*
HPDI			0.88 (0.84-0.92)*	0.91 (0.87-0.95)*
Asthma	1013/63086	1.87		
AMED			0.92 (0.88-0.96)*	0.95 (0.91-0.99)*
AEDII			0.96 (0.92-1.00)	1.00 (0.95-1.04)
AHEI-2010			0.92 (0.88-0.96)*	0.93 (0.89-0.97)*
HPDI			0.94 (0.90-0.99)*	0.97 (0.93-1.02)
Bronchiectasis	380/71309	0.62		
AMED			0.90 (0.84-0.97)*	0.90 (0.84-0.97)*
AEDII			1.00 (0.93-1.08)	1.00 (0.93-1.07)
AHEI-2010			0.89 (0.83-0.96)*	0.88 (0.82-0.95)*
HPDI			0.96 (0.90-1.04)	0.96 (0.89-1.03)
Osteoporosis	3689/65525	6.54		
AMED			0.93 (0.91-0.95)*	0.97 (0.94-0.99)*
AEDII			0.95 (0.93-0.97)*	0.98 (0.96-1.01)
AHEI-2010			0.92 (0.90-0.95)*	0.94 (0.92-0.97)*
HPDI			0.96 (0.94-0.98)*	1.00 (0.97-1.02)
Fracture	454/71149	0.74		
AMED			1.03 (0.96-1.10)	1.01 (0.95-1.08)
AEDII			1.06 (0.99-1.13)	1.04 (0.97-1.11)
AHEI-2010			1.02 (0.95-1.09)	1.00 (0.94-1.07)
HPDI			1.04 (0.97-1.11)	1.02 (0.95-1.09)
Chronic kidney dise	2491/71159	4.07		
AMED			0.84 (0.81-0.86)*	0.88 (0.86-0.91)*
AEDII			0.86 (0.83-0.88)*	0.90 (0.87-0.93)*
AHEI-2010			0.90 (0.88-0.93)*	0.93 (0.90-0.96)*
HPDI			0.86 (0.84-0.89)*	0.91 (0.88-0.93)*
Thyroid disorders	1299/67265	2.25		
AMED			0.94 (0.91-0.98)*	0.98 (0.94-1.02)
AEDII			0.90 (0.86-0.93)*	0.91 (0.88-0.95)*
AHEI-2010			1.00 (0.96-1.04)	1.02 (0.98-1.06)
HPDI			0.96 (0.92-1.00)	0.99 (0.95-1.03)
Psoriasis/eczema	705/68579	1.20		
AMED			0.91 (0.86-0.95)*	0.92 (0.87-0.97)*
AEDII			0.96 (0.91-1.01)	0.97 (0.92-1.03)
AHEI-2010			0.90 (0.86-0.95)*	0.91 (0.87-0.96)*
HPDI			0.96 (0.91-1.01)	0.97 (0.92-1.02)
Meniere's disease	58/71299	0.09		
AMED			1.01 (0.84-1.22)	1.04 (0.86-1.25)
AEDII			0.98 (0.82-1.18)	1.01 (0.84-1.22)
AHEI-2010			0.96 (0.80-1.15)	0.97 (0.81-1.17)
HPDI			0.86 (0.71-1.03)	0.88 (0.73-1.06)
Prostate disorders	3310/49307	7.76		
AMED			0.97 (0.94-0.99)*	0.97 (0.94-0.99)*
AEDII			0.97 (0.95-0.99)*	0.97 (0.95-0.995)*
AHEI-2010			0.97 (0.95-0.99)*	0.97 (0.95-0.995)*
HPDI			0.96 (0.94-0.99)*	0.96 (0.94-0.99)*
Glaucoma	948/70631	1.56		
AMED			0.98 (0.94-1.03)	0.97 (0.92-1.02)
AEDII			0.99 (0.94-1.03)	0.98 (0.94-1.03)
AHEI-2010			1.03 (0.98-1.08)	1.02 (0.97-1.07)
HPDI			1.03 (0.99-1.08)	1.03 (0.98-1.08)
Cataract	4574/69253	7.68		
AMED			0.97 (0.95-0.99)*	0.97 (0.95-0.99)*
AEDII			0.98 (0.96-1.00)	0.99 (0.96-1.01)
AHEI-2010			0.98 (0.96-1.00)	0.99 (0.97-1.01)
HPDI			0.98 (0.96-1.01)	0.99 (0.97-1.01)
AMD	767/71330	1.25		
AMED			0.96 (0.91-1.01)	0.96 (0.92-1.01)
AEDII			0.99 (0.94-1.04)	1.00 (0.95-1.05)
AHEI-2010			0.99 (0.94-1.04)	0.98 (0.93-1.04)
HPDI			1.00 (0.95-1.05)	1.00 (0.95-1.05)
Pernicious anaemia	91/71327	0.15		
AMED			0.77 (0.67-0.90)*	0.79 (0.70-0.89)*
AEDII			0.86 (0.74-1.00)	0.89 (0.76-1.03)
AHEI-2010			0.86 (0.74-1.00)	0.87 (0.74-1.02)
HPDI			0.78 (0.67-0.90)*	0.79 (0.68-0.92)*

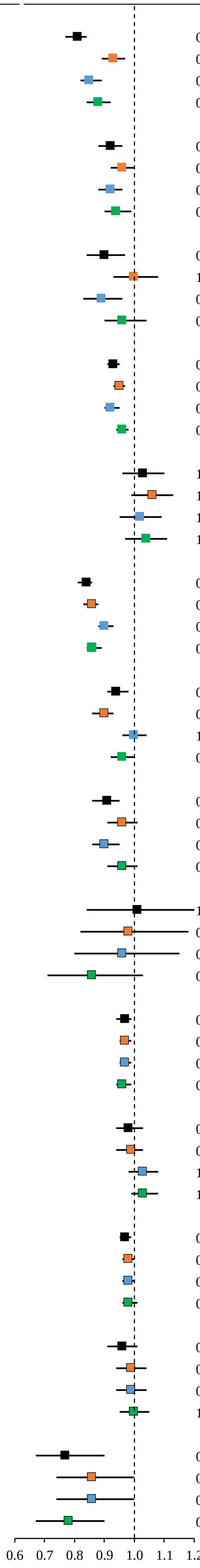

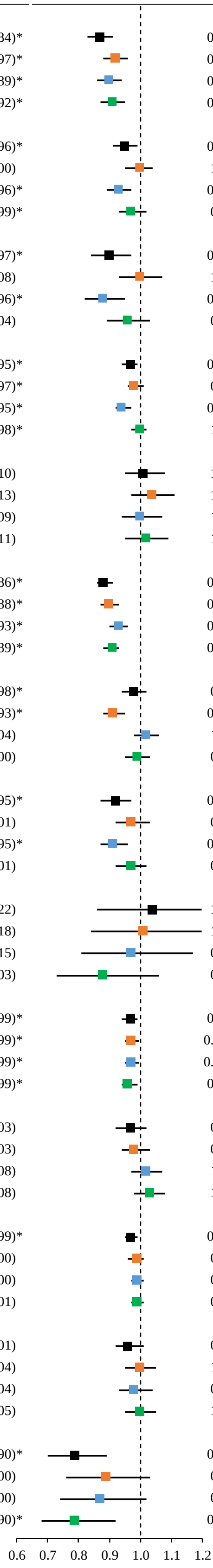

Figure S14. The association between dietary scores and the risk of other chronic diseases among individuals with three or more dietary assessments

AEDII, Anti-Empirical Dietary Inflammatory Index; AHEI-2010, Alternate Healthy Eating Index-2010; AMED, Alternate Mediterranean Diet score; AMD, age related macular degeneration; CI, confidence interval; COPD, chronic obstructive pulmonary disease; HPDI, Healthful Plant-based Diet Index.

The incidence refers to the number of event cases per 1000 person-years. Cox proportional hazard regression models were used to examine associations of each of the four dietary scores with the risk of individual other chronic diseases. Model 1 was adjusted for age, sex, and total energy intake; Model 2 was adjusted for Model 1 plus ethnicity, education, income, BMI, smoking, sleep, physical activity, and GRS for longevity. Dietary scores were analyzed as continuous variables (each quintile increment). The analysis for prostate disorders was conducted among men only. The vertical dash lines represent the hazard ratio of 1. Squares represent the hazard ratios (black color for AMED, orange color for AEDII, blue color for AHEI-2010, and green color for HPDI). Horizontal lines indicate the range of the 95% confidence interval.

*Indicates a significant association through two-sided statistical tests while controlling for FDR.

Table S1. The association between the Alternate Mediterranean Diet Index and the incidence of individual chronic diseases

	Alternate Mediterranean Diet Index					P-value
	Quintile 1	Quintile 2	Quintile 3	Quintile 4	Quintile 5	for trend*
Cardiovascular disease[†]						
Events	1934	1735	1856	1535	1865	
Person-years	179474	174641	202785	183701	231642	
HR (95% CI), Model 1	Reference	0.90 (0.84-0.96)	0.81 (0.76-0.86)	0.74 (0.70-0.80)	0.71 (0.66-0.75)	<0.0001
HR (95% CI), Model 2	Reference	0.92 (0.87-0.99)	0.85 (0.79-0.90)	0.80 (0.75-0.86)	0.79 (0.74-0.84)	<0.0001
Coronary heart disease						
Events	1081	953	983	839	964	
Person-years	182900	177386	205885	186333	235142	
HR (95% CI), Model 1	Reference	0.90 (0.83-0.98)	0.79 (0.73-0.87)	0.77 (0.70-0.84)	0.70 (0.64-0.77)	<0.0001
HR (95% CI), Model 2	Reference	0.93 (0.86-1.02)	0.84 (0.77-0.91)	0.84 (0.76-0.92)	0.80 (0.73-0.87)	<0.0001
Heart failure						
Events	353	313	312	276	264	
Person-years	185511	179575	208224	188405	237489	
HR (95% CI), Model 1	Reference	0.89 (0.76-1.03)	0.75 (0.64-0.87)	0.73 (0.63-0.86)	0.55 (0.46-0.64)	<0.0001
HR (95% CI), Model 2	Reference	0.94 (0.80-1.09)	0.81 (0.70-0.95)	0.83 (0.71-0.98)	0.67 (0.56-0.79)	<0.0001
Atrial fibrillation						
Events	397	370	357	327	380	
Person-years	184263	178338	206738	187117	235985	
HR (95% CI), Model 1	Reference	0.94 (0.81-1.08)	0.77 (0.66-0.88)	0.79 (0.68-0.91)	0.71 (0.62-0.82)	<0.0001
HR (95% CI), Model 2	Reference	0.97 (0.84-1.12)	0.81 (0.70-0.93)	0.85 (0.74-0.99)	0.82 (0.71-0.95)	0.0066
Other cardiac disease						
Events	795	718	749	586	738	
Person-years	184656	178669	207371	187654	236523	
HR (95% CI), Model 1	Reference	0.91 (0.82-1.01)	0.80 (0.72-0.88)	0.69 (0.62-0.77)	0.68 (0.62-0.76)	<0.0001
HR (95% CI), Model 2	Reference	0.94 (0.85-1.04)	0.85 (0.76-0.93)	0.75 (0.67-0.84)	0.77 (0.69-0.85)	<0.0001
Stroke						
Events	166	135	161	129	128	
Person-years	185253	179349	208137	188232	237353	
HR (95% CI), Model 1	Reference	0.81 (0.65-1.02)	0.82 (0.66-1.02)	0.72 (0.57-0.91)	0.56 (0.44-0.70)	<0.0001
HR (95% CI), Model 2	Reference	0.84 (0.67-1.05)	0.85 (0.68-1.06)	0.76 (0.61-0.97)	0.60 (0.47-0.76)	0.0010
Peripheral vascular disease						
Events	194	174	184	138	180	
Person-years	185270	179427	208177	188277	237277	
HR (95% CI), Model 1	Reference	0.90 (0.74-1.11)	0.81 (0.66-0.99)	0.67 (0.54-0.83)	0.68 (0.55-0.84)	0.0004
HR (95% CI), Model 2	Reference	0.94 (0.76-1.15)	0.85 (0.70-1.05)	0.72 (0.57-0.89)	0.75 (0.60-0.92)	0.0115
Hypertension						
Events	875	718	829	659	801	
Person-years	140746	136975	160561	147647	189005	
HR (95% CI), Model 1	Reference	0.82 (0.74-0.91)	0.79 (0.72-0.87)	0.68 (0.61-0.75)	0.63 (0.57-0.69)	<0.0001
HR (95% CI), Model 2	Reference	0.86 (0.78-0.95)	0.86 (0.78-0.94)	0.77 (0.70-0.85)	0.77 (0.69-0.85)	<0.0001

Diabetes						
Events	575	530	503	396	447	
Person-years	178251	172903	201465	182669	231597	
HR (95% CI), Model 1	Reference	0.95 (0.84-1.07)	0.77 (0.68-0.87)	0.68 (0.59-0.77)	0.60 (0.53-0.68)	<0.0001
HR (95% CI), Model 2	Reference	1.07 (0.95-1.21)	0.91 (0.80-1.02)	0.87 (0.77-0.99)	0.89 (0.78-1.01)	0.0047
All cancers‡						
Events	1308	1166	1251	1040	1284	
Person-years	173558	168083	195731	176159	222541	
HR (95% CI), Model 1	Reference	0.89 (0.82-0.96)	0.79 (0.73-0.86)	0.72 (0.67-0.79)	0.69 (0.64-0.75)	<0.0001
HR (95% CI), Model 2	Reference	0.91 (0.84-0.99)	0.83 (0.76-0.89)	0.77 (0.71-0.84)	0.76 (0.70-0.82)	<0.0001
Non-melanoma skin cancer						
Events	674	628	724	659	874	
Person-years	183425	177283	205619	185858	234092	
HR (95% CI), Model 1	Reference	0.94 (0.84-1.05)	0.91 (0.82-1.02)	0.92 (0.83-1.03)	0.96 (0.86-1.06)	0.49
HR (95% CI), Model 2	Reference	0.92 (0.83-1.03)	0.89 (0.80-0.99)	0.90 (0.80-1.00)	0.92 (0.82-1.02)	0.25
Melanoma						
Events	87	89	99	84	126	
Person-years	184493	178156	206726	186569	235490	
HR (95% CI), Model 1	Reference	1.04 (0.77-1.40)	0.98 (0.73-1.31)	0.92 (0.68-1.24)	1.07 (0.81-1.41)	0.85
HR (95% CI), Model 2	Reference	1.04 (0.77-1.39)	0.97 (0.73-1.30)	0.91 (0.67-1.24)	1.07 (0.81-1.42)	0.84
Lung cancer						
Events	176	131	101	90	111	
Person-years	185751	179747	208583	188563	237723	
HR (95% CI), Model 1	Reference	0.73 (0.58-0.91)	0.46 (0.36-0.59)	0.45 (0.35-0.58)	0.42 (0.33-0.53)	<0.0001
HR (95% CI), Model 2	Reference	0.80 (0.64-1.00)	0.53 (0.42-0.68)	0.53 (0.41-0.69)	0.53 (0.42-0.68)	<0.0001
Stomach cancer						
Events	32	34	28	24	27	
Person-years	185774	179753	208573	188611	237718	
HR (95% CI), Model 1	Reference	1.09 (0.67-1.77)	0.78 (0.47-1.29)	0.75 (0.44-1.29)	0.68 (0.40-1.14)	0.31
HR (95% CI), Model 2	Reference	1.14 (0.71-1.86)	0.83 (0.50-1.38)	0.83 (0.49-1.43)	0.80 (0.47-1.35)	0.59
Oesophageal cancer						
Events	54	47	29	30	34	
Person-years	185772	179754	208567	188618	237786	
HR (95% CI), Model 1	Reference	0.88 (0.60-1.31)	0.46 (0.29-0.73)	0.54 (0.34-0.84)	0.48 (0.31-0.74)	0.0004
HR (95% CI), Model 2	Reference	0.96 (0.65-1.42)	0.52 (0.33-0.82)	0.64 (0.41-1.00)	0.62 (0.40-0.97)	0.0159
Colon cancer						
Events	163	149	149	155	165	
Person-years	184901	179007	207830	187890	236939	
HR (95% CI), Model 1	Reference	0.92 (0.73-1.15)	0.77 (0.62-0.97)	0.89 (0.71-1.11)	0.73 (0.59-0.91)	0.0379
HR (95% CI), Model 2	Reference	0.94 (0.75-1.18)	0.80 (0.64-1.01)	0.94 (0.75-1.18)	0.81 (0.64-1.01)	0.19
Ovarian cancer§						
Events	42	33	50	47	49	
Person-years	94169	98811	121726	117613	157791	
HR (95% CI), Model 1	Reference	0.72 (0.46-1.14)	0.86 (0.57-1.30)	0.82 (0.54-1.24)	0.61 (0.40-0.93)	0.19

HR (95% CI), Model 2	Reference	0.74 (0.47-1.16)	0.89 (0.59-1.35)	0.85 (0.56-1.30)	0.66 (0.43-1.00)	0.32
Breast cancer§						
Events	346	316	383	375	486	
Person-years	90043	94302	116620	112496	150030	
HR (95% CI), Model 1	Reference	0.86 (0.74-1.00)	0.84 (0.72-0.97)	0.84 (0.73-0.98)	0.81 (0.70-0.93)	0.0367
HR (95% CI), Model 2	Reference	0.88 (0.75-1.02)	0.86 (0.75-1.00)	0.88 (0.76-1.02)	0.86 (0.74-0.99)	0.23
Prostate cancer¶						
Events	406	413	444	392	425	
Person-years	101367	88578	95657	78618	87976	
HR (95% CI), Model 1	Reference	1.11 (0.96-1.27)	1.07 (0.93-1.22)	1.13 (0.99-1.30)	1.06 (0.92-1.22)	0.46
HR (95% CI), Model 2	Reference	1.08 (0.95-1.24)	1.04 (0.91-1.19)	1.10 (0.95-1.26)	1.01 (0.87-1.16)	0.58
Other cancers						
Events	1149	976	1086	898	1080	
Person-years	175658	170433	198260	178715	225408	
HR (95% CI), Model 1	Reference	0.85 (0.78-0.92)	0.79 (0.73-0.86)	0.72 (0.65-0.78)	0.67 (0.61-0.72)	<0.0001
HR (95% CI), Model 2	Reference	0.87 (0.79-0.94)	0.82 (0.75-0.89)	0.76 (0.69-0.83)	0.73 (0.67-0.79)	<0.0001
Depression						
Events	244	184	185	178	204	
Person-years	174476	170054	197781	179290	227066	
HR (95% CI), Model 1	Reference	0.75 (0.62-0.91)	0.64 (0.53-0.77)	0.66 (0.54-0.80)	0.58 (0.48-0.70)	<0.0001
HR (95% CI), Model 2	Reference	0.80 (0.66-0.96)	0.69 (0.57-0.84)	0.75 (0.62-0.92)	0.70 (0.58-0.85)	0.0008
Anxiety						
Events	665	543	603	488	622	
Person-years	181770	176250	204367	185197	233488	
HR (95% CI), Model 1	Reference	0.80 (0.72-0.90)	0.75 (0.67-0.83)	0.64 (0.57-0.72)	0.63 (0.56-0.70)	<0.0001
HR (95% CI), Model 2	Reference	0.83 (0.74-0.93)	0.79 (0.71-0.88)	0.70 (0.62-0.79)	0.71 (0.63-0.79)	<0.0001
Schizophrenia						
Events	24	25	19	21	26	
Person-years	185265	179267	207936	188024	237271	
HR (95% CI), Model 1	Reference	1.05 (0.60-1.83)	0.68 (0.37-1.24)	0.81 (0.45-1.46)	0.77 (0.44-1.35)	0.56
HR (95% CI), Model 2	Reference	1.19 (0.68-2.10)	0.83 (0.45-1.52)	1.06 (0.58-1.92)	1.12 (0.63-1.99)	0.80
Alcohol use disorder						
Events	232	165	145	122	91	
Person-years	184872	179074	207866	188129	237508	
HR (95% CI), Model 1	Reference	0.75 (0.61-0.91)	0.58 (0.47-0.71)	0.55 (0.44-0.69)	0.33 (0.26-0.43)	<0.0001
HR (95% CI), Model 2	Reference	0.79 (0.64-0.96)	0.63 (0.51-0.77)	0.62 (0.50-0.78)	0.39 (0.30-0.50)	<0.0001
Psychoactive substance abuse						
Events	48	33	27	17	20	
Person-years	185529	179448	208403	188477	237673	
HR (95% CI), Model 1	Reference	0.74 (0.47-1.15)	0.54 (0.33-0.86)	0.39 (0.22-0.68)	0.38 (0.22-0.64)	0.0004
HR (95% CI), Model 2	Reference	0.81 (0.52-1.27)	0.61 (0.38-0.99)	0.47 (0.27-0.82)	0.48 (0.28-0.82)	0.0177
Epilepsy						
Events	95	65	74	69	63	
Person-years	184231	178569	206904	187535	236381	

HR (95% CI), Model 1	Reference	0.70 (0.51-0.95)	0.68 (0.50-0.92)	0.69 (0.51-0.95)	0.50 (0.36-0.69)	0.0009
HR (95% CI), Model 2	Reference	0.71 (0.52-0.98)	0.70 (0.51-0.94)	0.73 (0.53-1.00)	0.53 (0.38-0.74)	0.0049
Migraine						
Events	129	116	121	109	169	
Person-years	179707	173982	201481	182248	229349	
HR (95% CI), Model 1	Reference	0.91 (0.71-1.17)	0.81 (0.63-1.03)	0.79 (0.61-1.02)	0.96 (0.76-1.21)	0.24
HR (95% CI), Model 2	Reference	0.91 (0.71-1.17)	0.81 (0.63-1.04)	0.80 (0.62-1.04)	0.98 (0.77-1.24)	0.24
Dementia						
Events	144	132	130	108	159	
Person-years	185757	179703	208556	188577	237760	
HR (95% CI), Model 1	Reference	0.89 (0.70-1.13)	0.72 (0.57-0.92)	0.65 (0.51-0.84)	0.74 (0.59-0.93)	0.0045
HR (95% CI), Model 2	Reference	0.91 (0.72-1.15)	0.74 (0.59-0.95)	0.68 (0.53-0.87)	0.77 (0.61-0.97)	0.0152
Parkinson's disease						
Events	80	74	85	78	72	
Person-years	185636	179634	208288	188407	237582	
HR (95% CI), Model 1	Reference	0.93 (0.68-1.28)	0.91 (0.67-1.23)	0.93 (0.68-1.27)	0.67 (0.49-0.93)	0.15
HR (95% CI), Model 2	Reference	0.92 (0.67-1.27)	0.89 (0.66-1.21)	0.91 (0.66-1.25)	0.65 (0.47-0.90)	0.10
Multiple sclerosis						
Events	22	16	24	15	17	
Person-years	185090	179203	207783	187908	236904	
HR (95% CI), Model 1	Reference	0.75 (0.39-1.43)	0.97 (0.54-1.73)	0.66 (0.34-1.28)	0.59 (0.31-1.13)	0.41
HR (95% CI), Model 2	Reference	0.78 (0.41-1.48)	1.02 (0.57-1.84)	0.72 (0.37-1.40)	0.66 (0.35-1.28)	0.58
Bronchiectasis						
Events	147	126	130	107	138	
Person-years	185399	179273	207999	188116	237123	
HR (95% CI), Model 1	Reference	0.83 (0.65-1.05)	0.70 (0.55-0.88)	0.61 (0.48-0.78)	0.59 (0.47-0.75)	<0.0001
HR (95% CI), Model 2	Reference	0.85 (0.67-1.07)	0.72 (0.57-0.92)	0.63 (0.49-0.82)	0.61 (0.48-0.78)	0.0003
Asthma						
Events	366	283	347	307	353	
Person-years	162356	157725	183538	166101	211731	
HR (95% CI), Model 1	Reference	0.77 (0.66-0.90)	0.79 (0.69-0.92)	0.76 (0.66-0.89)	0.67 (0.58-0.78)	<0.0001
HR (95% CI), Model 2	Reference	0.80 (0.68-0.94)	0.84 (0.73-0.98)	0.84 (0.72-0.98)	0.77 (0.66-0.90)	0.0108
COPD						
Events	472	362	348	273	299	
Person-years	183104	177747	206370	186843	235767	
HR (95% CI), Model 1	Reference	0.76 (0.66-0.87)	0.61 (0.53-0.70)	0.52 (0.45-0.61)	0.44 (0.38-0.51)	<0.0001
HR (95% CI), Model 2	Reference	0.84 (0.73-0.97)	0.72 (0.63-0.83)	0.65 (0.56-0.76)	0.61 (0.52-0.70)	<0.0001
CKD						
Events	1006	803	830	670	749	
Person-years	184657	178865	207683	187803	236838	
HR (95% CI), Model 1	Reference	0.79 (0.72-0.87)	0.68 (0.62-0.75)	0.60 (0.55-0.67)	0.52 (0.48-0.58)	<0.0001
HR (95% CI), Model 2	Reference	0.83 (0.76-0.91)	0.74 (0.68-0.81)	0.68 (0.62-0.75)	0.63 (0.58-0.70)	<0.0001
Chronic liver disease						
Events	65	52	51	41	27	
Person-years	184607	178945	207812	187506	236753	

HR (95% CI), Model 1	Reference	0.83 (0.58-1.20)	0.70 (0.49-1.02)	0.64 (0.43-0.94)	0.34 (0.21-0.53)	0.0001
HR (95% CI), Model 2	Reference	0.91 (0.63-1.31)	0.82 (0.56-1.18)	0.79 (0.53-1.17)	0.46 (0.29-0.74)	0.0231
Irritable bowel syndrome						
Events	269	186	201	205	239	
Person-years	180794	174930	203270	183676	231855	
HR (95% CI), Model 1	Reference	0.68 (0.56-0.82)	0.61 (0.51-0.73)	0.66 (0.55-0.80)	0.58 (0.49-0.70)	<0.0001
HR (95% CI), Model 2	Reference	0.70 (0.58-0.85)	0.65 (0.54-0.78)	0.73 (0.60-0.87)	0.67 (0.56-0.80)	<0.0001
Inflammatory bowel disease						
Events	90	84	99	78	97	
Person-years	183284	178055	206771	186922	235926	
HR (95% CI), Model 1	Reference	0.97 (0.72-1.30)	0.98 (0.74-1.31)	0.86 (0.64-1.17)	0.85 (0.64-1.14)	0.74
HR (95% CI), Model 2	Reference	1.00 (0.75-1.35)	1.04 (0.78-1.38)	0.93 (0.68-1.27)	0.94 (0.70-1.27)	0.95
Treated constipation						
Events	748	601	683	555	747	
Person-years	183504	177750	206238	186878	235367	
HR (95% CI), Model 1	Reference	0.80 (0.72-0.89)	0.76 (0.69-0.84)	0.67 (0.60-0.75)	0.70 (0.63-0.78)	<0.0001
HR (95% CI), Model 2	Reference	0.81 (0.73-0.90)	0.78 (0.70-0.86)	0.69 (0.62-0.77)	0.73 (0.66-0.81)	<0.0001
Dyspepsia						
Events	1967	1851	2049	1796	2152	
Person-years	161654	160170	186776	169731	216999	
HR (95% CI), Model 1	Reference	0.92 (0.86-0.98)	0.86 (0.80-0.91)	0.81 (0.76-0.86)	0.74 (0.70-0.79)	<0.0001
HR (95% CI), Model 2	Reference	0.94 (0.89-1.01)	0.89 (0.83-0.94)	0.86 (0.81-0.92)	0.81 (0.76-0.86)	<0.0001
Diverticular disease						
Events	1652	1512	1733	1397	1708	
Person-years	178360	173725	201076	182796	231244	
HR (95% CI), Model 1	Reference	0.91 (0.85-0.98)	0.88 (0.82-0.94)	0.77 (0.72-0.83)	0.73 (0.68-0.78)	<0.0001
HR (95% CI), Model 2	Reference	0.94 (0.88-1.01)	0.93 (0.87-0.99)	0.83 (0.77-0.90)	0.82 (0.77-0.88)	<0.0001
Pernicious anaemia						
Events	42	29	24	20	21	
Person-years	185284	179260	208111	188195	237266	
HR (95% CI), Model 1	Reference	0.68 (0.42-1.09)	0.46 (0.28-0.77)	0.41 (0.24-0.71)	0.33 (0.19-0.56)	<0.0001
HR (95% CI), Model 2	Reference	0.71 (0.44-1.14)	0.50 (0.30-0.83)	0.46 (0.27-0.79)	0.38 (0.22-0.66)	0.0024
Fracture						
Events	141	136	142	149	191	
Person-years	184817	178762	207617	187792	236670	
HR (95% CI), Model 1	Reference	0.94 (0.74-1.19)	0.80 (0.64-1.02)	0.90 (0.72-1.14)	0.87 (0.70-1.09)	0.45
HR (95% CI), Model 2	Reference	0.94 (0.74-1.18)	0.79 (0.63-1.00)	0.88 (0.70-1.11)	0.83 (0.66-1.04)	0.30
Osteoporosis						
Events	1220	1107	1272	1152	1364	
Person-years	169269	165208	190803	172687	218473	
HR (95% CI), Model 1	Reference	0.89 (0.82-0.96)	0.85 (0.78-0.92)	0.83 (0.76-0.90)	0.74 (0.69-0.80)	<0.0001
HR (95% CI), Model 2	Reference	0.92 (0.85-1.00)	0.91 (0.84-0.98)	0.92 (0.85-1.00)	0.88 (0.81-0.95)	0.0308
Meniere's disease						
Events	20	16	19	22	24	
Person-years	185327	179344	207816	188129	237036	

HR (95% CI), Model 1	Reference	0.82 (0.42-1.58)	0.83 (0.44-1.56)	1.06 (0.58-1.96)	0.92 (0.50-1.68)	0.91
HR (95% CI), Model 2	Reference	0.84 (0.43-1.62)	0.87 (0.46-1.63)	1.14 (0.61-2.10)	1.02 (0.55-1.89)	0.88
Eczema						
Events	259	217	226	197	233	
Person-years	177847	172311	200365	181060	228585	
HR (95% CI), Model 1	Reference	0.85 (0.71-1.02)	0.75 (0.63-0.90)	0.72 (0.60-0.87)	0.66 (0.56-0.80)	0.0001
HR (95% CI), Model 2	Reference	0.86 (0.72-1.04)	0.77 (0.65-0.93)	0.76 (0.63-0.91)	0.72 (0.60-0.86)	0.0034
Glaucoma						
Events	266	287	327	282	381	
Person-years	183795	177555	206177	186420	234904	
HR (95% CI), Model 1	Reference	1.08 (0.91-1.27)	1.02 (0.87-1.21)	0.97 (0.82-1.15)	1.01 (0.86-1.19)	0.78
HR (95% CI), Model 2	Reference	1.07 (0.90-1.26)	1.01 (0.86-1.19)	0.95 (0.80-1.12)	0.97 (0.83-1.14)	0.69
Cataract						
Events	1386	1306	1490	1482	1822	
Person-years	180498	174625	202287	183047	230150	
HR (95% CI), Model 1	Reference	0.91 (0.85-0.98)	0.85 (0.79-0.91)	0.91 (0.84-0.98)	0.85 (0.79-0.91)	<0.0001
HR (95% CI), Model 2	Reference	0.92 (0.86-1.00)	0.87 (0.80-0.93)	0.93 (0.87-1.00)	0.88 (0.82-0.94)	0.0010
AMD						
Events	222	247	256	243	310	
Person-years	185448	179263	208165	188033	237161	
HR (95% CI), Model 1	Reference	1.08 (0.90-1.30)	0.91 (0.76-1.09)	0.92 (0.77-1.11)	0.89 (0.74-1.05)	0.15
HR (95% CI), Model 2	Reference	1.10 (0.92-1.32)	0.93 (0.78-1.12)	0.96 (0.80-1.15)	0.93 (0.78-1.11)	0.31
Thyroid disorders						
Events	426	364	437	396	484	
Person-years	174786	169636	196180	177193	223434	
HR (95% CI), Model 1	Reference	0.84 (0.73-0.96)	0.84 (0.73-0.96)	0.81 (0.71-0.93)	0.75 (0.66-0.86)	0.0010
HR (95% CI), Model 2	Reference	0.86 (0.75-0.99)	0.88 (0.77-1.01)	0.88 (0.76-1.01)	0.86 (0.75-0.98)	0.16
Prostate disorders¶						
Events	757	635	687	582	649	
Person-years	95699	83753	90280	73983	82675	
HR (95% CI), Model 1	Reference	0.90 (0.81-1.00)	0.87 (0.78-0.96)	0.89 (0.80-0.99)	0.85 (0.77-0.95)	0.0253
HR (95% CI), Model 2	Reference	0.90 (0.81-1.00)	0.87 (0.79-0.97)	0.89 (0.80-1.00)	0.86 (0.77-0.96)	0.0463

AMD, age related macular degeneration; CI, confidence interval; CKD, chronic kidney disease; COPD, chronic obstructive pulmonary disease; HR, hazard ratio.

*Cox proportional hazard regression models were used to examine associations of the Alternate Mediterranean Diet Index (quintiles) with the incidence of individual chronic diseases. The statistical tests were two-sided. Model 1 was adjusted for age and sex; Model 2 was adjusted for Model 1 plus ethnicity, education, income, smoking, alcohol consumption, sleep, physical activity, and GRS for longevity; Model 3 was adjusted for Model 2 and total energy intake.

†Cardiovascular disease includes coronary heart disease, heart failure, atrial fibrillation, other cardiac disease, stroke, and peripheral vascular disease.

‡All cancers encompass any type of cancer except for non-melanoma skin cancer.

§These analyses were conducted among women only.

¶These analyses were conducted among men only.

Table S2. The association between the Anti-Empirical Dietary Inflammatory Index and the incidence of individual chronic diseases

	Anti-Empirical Dietary Inflammatory Index					P-value
	Quintile 1	Quintile 2	Quintile 3	Quintile 4	Quintile 5	for trend*
Cardiovascular disease†						
Events	1848	1736	1787	1722	1832	
Person-years	186833	189651	189808	190269	190653	
HR (95% CI), Model 1	Reference	0.86 (0.80-0.91)	0.84 (0.78-0.89)	0.77 (0.72-0.82)	0.79 (0.74-0.84)	<0.0001
HR (95% CI), Model 2	Reference	0.90 (0.84-0.96)	0.89 (0.83-0.95)	0.84 (0.78-0.89)	0.86 (0.81-0.92)	<0.0001
Coronary heart disease						
Events	1044	959	953	898	966	
Person-years	194104	196233	196788	197158	197389	
HR (95% CI), Model 1	Reference	0.84 (0.77-0.92)	0.79 (0.73-0.87)	0.71 (0.65-0.78)	0.74 (0.68-0.81)	<0.0001
HR (95% CI), Model 2	Reference	0.90 (0.82-0.98)	0.86 (0.79-0.94)	0.79 (0.72-0.87)	0.83 (0.76-0.91)	<0.0001
Heart failure						
Events	323	308	310	290	287	
Person-years	199849	201514	201814	201976	202480	
HR (95% CI), Model 1	Reference	0.86 (0.73-1.00)	0.81 (0.69-0.95)	0.72 (0.61-0.84)	0.68 (0.58-0.80)	<0.0001
HR (95% CI), Model 2	Reference	0.94 (0.81-1.10)	0.91 (0.78-1.07)	0.84 (0.71-0.98)	0.81 (0.69-0.95)	0.0696
Atrial fibrillation						
Events	359	346	349	382	395	
Person-years	197275	199160	199268	199146	199806	
HR (95% CI), Model 1	Reference	0.87 (0.75-1.01)	0.82 (0.71-0.95)	0.85 (0.74-0.98)	0.84 (0.73-0.97)	0.0639
HR (95% CI), Model 2	Reference	0.93 (0.81-1.08)	0.89 (0.77-1.04)	0.95 (0.82-1.11)	0.95 (0.82-1.10)	0.68
Other cardiac disease						
Events	720	694	750	732	690	
Person-years	197356	199433	199456	199690	200659	
HR (95% CI), Model 1	Reference	0.88 (0.79-0.97)	0.90 (0.81-1.00)	0.84 (0.76-0.93)	0.76 (0.68-0.84)	<0.0001
HR (95% CI), Model 2	Reference	0.93 (0.84-1.03)	0.96 (0.87-1.06)	0.91 (0.82-1.01)	0.84 (0.75-0.93)	0.0179
Stroke						
Events	146	138	139	138	158	
Person-years	199885	201598	201696	202074	202395	
HR (95% CI), Model 1	Reference	0.86 (0.68-1.09)	0.82 (0.65-1.04)	0.78 (0.61-0.98)	0.86 (0.69-1.08)	0.28
HR (95% CI), Model 2	Reference	0.89 (0.71-1.13)	0.86 (0.68-1.08)	0.82 (0.65-1.04)	0.91 (0.72-1.15)	0.53
Peripheral vascular disease						
Events	194	153	165	176	182	
Person-years	199999	201870	202008	202356	202715	
HR (95% CI), Model 1	Reference	0.73 (0.59-0.90)	0.76 (0.62-0.93)	0.78 (0.64-0.96)	0.79 (0.64-0.97)	0.0237
HR (95% CI), Model 2	Reference	0.74 (0.60-0.92)	0.77 (0.63-0.95)	0.80 (0.65-0.98)	0.80 (0.65-0.99)	0.0485
Hypertension						
Events	790	759	759	753	821	
Person-years	144563	150133	151007	153200	153548	
HR (95% CI), Model 1	Reference	0.85 (0.77-0.94)	0.80 (0.72-0.89)	0.74 (0.67-0.82)	0.79 (0.72-0.87)	<0.0001
HR (95% CI), Model 2	Reference	0.94 (0.85-1.03)	0.90 (0.82-1.00)	0.87 (0.78-0.96)	0.94 (0.85-1.04)	0.0741

Diabetes						
Events	701	513	453	414	370	
Person-years	188726	193160	194638	196272	197261	
HR (95% CI), Model 1	Reference	0.68 (0.61-0.76)	0.58 (0.51-0.65)	0.51 (0.45-0.57)	0.44 (0.39-0.50)	<0.0001
HR (95% CI), Model 2	Reference	0.84 (0.75-0.94)	0.74 (0.65-0.83)	0.70 (0.62-0.80)	0.64 (0.56-0.72)	<0.0001
All cancers‡						
Events	1118	1169	1232	1275	1255	
Person-years	183586	184369	184542	184284	185076	
HR (95% CI), Model 1	Reference	0.97 (0.89-1.05)	0.98 (0.91-1.07)	0.98 (0.91-1.06)	0.94 (0.87-1.02)	0.68
HR (95% CI), Model 2	Reference	1.00 (0.92-1.08)	1.01 (0.93-1.10)	1.02 (0.94-1.10)	0.97 (0.89-1.06)	0.79
Non-melanoma skin cancer						
Events	612	654	701	805	787	
Person-years	196196	197290	197639	196936	197476	
HR (95% CI), Model 1	Reference	0.98 (0.88-1.09)	1.00 (0.89-1.11)	1.10 (0.99-1.22)	1.04 (0.93-1.16)	0.18
HR (95% CI), Model 2	Reference	0.96 (0.86-1.07)	0.97 (0.87-1.08)	1.06 (0.95-1.18)	1.01 (0.91-1.12)	0.33
Melanoma						
Events	71	110	99	102	103	
Person-years	199394	200612	200691	201006	201134	
HR (95% CI), Model 1	Reference	1.46 (1.09-1.97)	1.28 (0.94-1.73)	1.27 (0.94-1.73)	1.26 (0.93-1.71)	0.18
HR (95% CI), Model 2	Reference	1.44 (1.06-1.94)	1.25 (0.92-1.70)	1.24 (0.91-1.68)	1.23 (0.90-1.67)	0.23
Lung cancer						
Events	115	95	122	138	139	
Person-years	200687	202328	202506	202715	203121	
HR (95% CI), Model 1	Reference	0.75 (0.57-0.98)	0.92 (0.71-1.19)	0.99 (0.77-1.27)	0.97 (0.76-1.25)	0.21
HR (95% CI), Model 2	Reference	0.78 (0.59-1.02)	0.94 (0.72-1.21)	1.00 (0.77-1.28)	0.91 (0.71-1.17)	0.36
Stomach cancer						
Events	30	34	23	35	23	
Person-years	201129	202687	202928	203082	203598	
HR (95% CI), Model 1	Reference	1.05 (0.64-1.72)	0.67 (0.39-1.16)	0.98 (0.60-1.60)	0.62 (0.36-1.07)	0.17
HR (95% CI), Model 2	Reference	1.14 (0.69-1.86)	0.74 (0.43-1.28)	1.10 (0.67-1.80)	0.70 (0.40-1.22)	0.24
Oesophageal cancer						
Events	32	38	40	34	50	
Person-years	201073	202691	202889	203134	203481	
HR (95% CI), Model 1	Reference	1.08 (0.67-1.73)	1.07 (0.67-1.70)	0.86 (0.53-1.40)	1.22 (0.78-1.91)	0.63
HR (95% CI), Model 2	Reference	1.19 (0.74-1.91)	1.21 (0.75-1.93)	1.01 (0.62-1.64)	1.43 (0.91-2.25)	0.48
Colon cancer						
Events	159	137	149	156	180	
Person-years	199602	201543	201603	201800	202169	
HR (95% CI), Model 1	Reference	0.79 (0.63-1.00)	0.82 (0.66-1.03)	0.83 (0.66-1.03)	0.93 (0.75-1.15)	0.22
HR (95% CI), Model 2	Reference	0.82 (0.65-1.04)	0.86 (0.69-1.08)	0.87 (0.70-1.09)	0.97 (0.78-1.21)	0.36
Ovarian cancer§						
Events	51	48	39	46	37	
Person-years	119355	121632	119607	118748	116206	
HR (95% CI), Model 1	Reference	0.89 (0.60-1.33)	0.72 (0.47-1.09)	0.84 (0.56-1.25)	0.67 (0.44-1.03)	0.36

HR (95% CI), Model 2	Reference	0.93 (0.62-1.38)	0.76 (0.50-1.16)	0.91 (0.60-1.36)	0.75 (0.49-1.16)	0.72
Breast cancer[§]						
Events	371	408	392	378	357	
Person-years	113147	114402	112356	111467	109061	
HR (95% CI), Model 1	Reference	1.08 (0.93-1.24)	1.04 (0.90-1.20)	1.01 (0.87-1.16)	0.96 (0.83-1.12)	0.61
HR (95% CI), Model 2	Reference	1.10 (0.95-1.26)	1.07 (0.93-1.23)	1.03 (0.89-1.20)	1.00 (0.86-1.16)	0.63
Prostate cancer[¶]						
Events	339	386	460	430	466	
Person-years	91020	87982	89961	90493	93306	
HR (95% CI), Model 1	Reference	1.10 (0.95-1.27)	1.21 (1.05-1.39)	1.08 (0.94-1.25)	1.11 (0.96-1.28)	0.12
HR (95% CI), Model 2	Reference	1.07 (0.93-1.24)	1.17 (1.02-1.35)	1.04 (0.90-1.20)	1.07 (0.93-1.23)	0.24
Other cancers						
Events	970	1000	1072	1073	1074	
Person-years	186565	187613	187923	187849	188676	
HR (95% CI), Model 1	Reference	0.96 (0.88-1.05)	0.99 (0.90-1.08)	0.95 (0.87-1.04)	0.93 (0.85-1.02)	0.49
HR (95% CI), Model 2	Reference	0.99 (0.90-1.08)	1.02 (0.94-1.12)	0.99 (0.91-1.08)	0.97 (0.88-1.06)	0.78
Depression						
Events	242	217	167	203	166	
Person-years	187863	190426	192127	192180	193075	
HR (95% CI), Model 1	Reference	0.89 (0.74-1.06)	0.68 (0.56-0.83)	0.83 (0.68-1.00)	0.68 (0.56-0.83)	0.0002
HR (95% CI), Model 2	Reference	0.96 (0.80-1.16)	0.74 (0.61-0.91)	0.93 (0.77-1.12)	0.77 (0.63-0.95)	0.0102
Anxiety						
Events	699	575	551	583	513	
Person-years	194044	196717	197311	197589	198659	
HR (95% CI), Model 1	Reference	0.79 (0.71-0.89)	0.75 (0.67-0.84)	0.79 (0.70-0.88)	0.69 (0.61-0.77)	<0.0001
HR (95% CI), Model 2	Reference	0.83 (0.75-0.93)	0.80 (0.71-0.89)	0.85 (0.76-0.95)	0.75 (0.67-0.84)	<0.0001
Schizophrenia						
Events	21	28	24	18	24	
Person-years	200500	202104	202506	202736	203097	
HR (95% CI), Model 1	Reference	1.34 (0.76-2.37)	1.14 (0.63-2.05)	0.85 (0.45-1.59)	1.12 (0.62-2.01)	0.63
HR (95% CI), Model 2	Reference	1.56 (0.88-2.75)	1.37 (0.76-2.48)	1.08 (0.57-2.05)	1.48 (0.81-2.72)	0.49
Alcohol use disorder						
Events	100	127	143	165	220	
Person-years	200127	201683	201905	202129	202317	
HR (95% CI), Model 1	Reference	1.26 (0.97-1.63)	1.38 (1.07-1.79)	1.57 (1.23-2.02)	2.05 (1.62-2.61)	<0.0001
HR (95% CI), Model 2	Reference	1.30 (1.00-1.70)	1.42 (1.10-1.84)	1.62 (1.26-2.09)	2.05 (1.61-2.61)	<0.0001
Psychoactive substance abuse						
Events	20	26	34	33	32	
Person-years	200957	202440	202722	203034	203466	
HR (95% CI), Model 1	Reference	1.32 (0.74-2.36)	1.72 (0.99-3.00)	1.68 (0.96-2.94)	1.62 (0.92-2.84)	0.29
HR (95% CI), Model 2	Reference	1.45 (0.81-2.60)	1.92 (1.10-3.36)	1.89 (1.08-3.33)	1.80 (1.01-3.19)	0.14
Epilepsy						
Events	76	66	66	80	78	
Person-years	199204	201090	201470	201703	202274	

HR (95% CI), Model 1	Reference	0.82 (0.59-1.14)	0.79 (0.57-1.11)	0.94 (0.68-1.29)	0.90 (0.66-1.24)	0.63
HR (95% CI), Model 2	Reference	0.83 (0.60-1.16)	0.82 (0.59-1.14)	0.97 (0.70-1.33)	0.93 (0.67-1.28)	0.69
Migraine						
Events	143	117	131	150	103	
Person-years	191840	194757	196398	196839	197851	
HR (95% CI), Model 1	Reference	0.80 (0.62-1.02)	0.89 (0.70-1.13)	1.02 (0.81-1.29)	0.71 (0.55-0.91)	0.0198
HR (95% CI), Model 2	Reference	0.81 (0.63-1.04)	0.90 (0.71-1.14)	1.03 (0.82-1.30)	0.71 (0.54-0.92)	0.0207
Dementia						
Events	123	131	135	140	144	
Person-years	200957	202488	202728	202936	203319	
HR (95% CI), Model 1	Reference	0.93 (0.73-1.19)	0.89 (0.70-1.14)	0.86 (0.68-1.10)	0.85 (0.67-1.09)	0.72
HR (95% CI), Model 2	Reference	0.95 (0.74-1.21)	0.91 (0.71-1.16)	0.89 (0.69-1.14)	0.88 (0.69-1.13)	0.85
Parkinson’s disease						
Events	64	78	83	73	91	
Person-years	200699	202339	202561	203007	203247	
HR (95% CI), Model 1	Reference	1.06 (0.76-1.48)	1.06 (0.76-1.46)	0.87 (0.63-1.22)	1.05 (0.76-1.45)	0.73
HR (95% CI), Model 2	Reference	1.06 (0.76-1.47)	1.05 (0.75-1.46)	0.87 (0.62-1.22)	1.05 (0.76-1.46)	0.71
Multiple sclerosis						
Events	27	18	17	19	13	
Person-years	200478	201794	202474	202431	202895	
HR (95% CI), Model 1	Reference	0.67 (0.37-1.22)	0.64 (0.35-1.18)	0.73 (0.41-1.32)	0.51 (0.26-0.99)	0.32
HR (95% CI), Model 2	Reference	0.68 (0.37-1.24)	0.65 (0.35-1.20)	0.74 (0.40-1.34)	0.50 (0.25-0.99)	0.33
Bronchiectasis						
Events	123	124	119	156	126	
Person-years	200239	201926	202100	202264	202731	
HR (95% CI), Model 1	Reference	0.90 (0.70-1.16)	0.83 (0.64-1.06)	1.03 (0.81-1.31)	0.81 (0.63-1.04)	0.19
HR (95% CI), Model 2	Reference	0.91 (0.71-1.17)	0.83 (0.64-1.07)	1.03 (0.81-1.31)	0.81 (0.63-1.04)	0.18
Asthma						
Events	337	325	347	349	298	
Person-years	171449	174064	175110	176889	177283	
HR (95% CI), Model 1	Reference	0.93 (0.79-1.08)	0.97 (0.83-1.12)	0.95 (0.82-1.10)	0.80 (0.68-0.94)	0.0603
HR (95% CI), Model 2	Reference	1.00 (0.86-1.17)	1.07 (0.92-1.24)	1.08 (0.93-1.26)	0.92 (0.79-1.08)	0.30
COPD						
Events	379	361	345	309	360	
Person-years	197312	199294	199736	200265	200106	
HR (95% CI), Model 1	Reference	0.86 (0.74-0.99)	0.78 (0.67-0.90)	0.66 (0.57-0.77)	0.75 (0.65-0.87)	<0.0001
HR (95% CI), Model 2	Reference	0.91 (0.79-1.06)	0.82 (0.71-0.95)	0.70 (0.60-0.82)	0.75 (0.65-0.87)	<0.0001
CKD						
Events	986	812	790	757	713	
Person-years	196633	198873	199715	199923	200682	
HR (95% CI), Model 1	Reference	0.74 (0.67-0.81)	0.67 (0.61-0.74)	0.61 (0.56-0.67)	0.56 (0.51-0.61)	<0.0001
HR (95% CI), Model 2	Reference	0.81 (0.74-0.89)	0.77 (0.70-0.84)	0.72 (0.65-0.79)	0.67 (0.61-0.74)	<0.0001
Chronic liver disease						
Events	64	56	38	46	32	
Person-years	199927	201719	201866	202205	202618	

HR (95% CI), Model 1	Reference	0.83 (0.58-1.20)	0.55 (0.37-0.83)	0.66 (0.45-0.96)	0.45 (0.29-0.69)	0.0012
HR (95% CI), Model 2	Reference	0.95 (0.66-1.36)	0.64 (0.43-0.96)	0.79 (0.54-1.17)	0.55 (0.35-0.85)	0.0314
Irritable bowel syndrome						
Events	259	241	224	210	166	
Person-years	193652	196297	197214	198070	198671	
HR (95% CI), Model 1	Reference	0.90 (0.75-1.07)	0.83 (0.69-0.99)	0.77 (0.64-0.92)	0.61 (0.50-0.74)	<0.0001
HR (95% CI), Model 2	Reference	0.94 (0.79-1.12)	0.87 (0.73-1.04)	0.82 (0.68-0.99)	0.65 (0.54-0.80)	0.0006
Inflammatory bowel disease						
Events	97	91	96	88	76	
Person-years	198310	200248	200848	201415	201833	
HR (95% CI), Model 1	Reference	0.92 (0.69-1.23)	0.97 (0.73-1.28)	0.88 (0.66-1.17)	0.75 (0.56-1.02)	0.40865
HR (95% CI), Model 2	Reference	0.96 (0.72-1.28)	1.01 (0.76-1.35)	0.93 (0.69-1.25)	0.79 (0.58-1.07)	0.53324
Treated constipation						
Events	686	699	646	664	639	
Person-years	196546	197739	198510	198591	199575	
HR (95% CI), Model 1	Reference	0.95 (0.86-1.06)	0.85 (0.76-0.94)	0.84 (0.76-0.94)	0.79 (0.71-0.88)	<0.0001
HR (95% CI), Model 2	Reference	0.98 (0.88-1.09)	0.87 (0.78-0.97)	0.88 (0.79-0.98)	0.83 (0.74-0.92)	0.0022
Dyspepsia						
Events	2094	2038	1903	1962	1818	
Person-years	167220	171736	173649	176081	176843	
HR (95% CI), Model 1	Reference	0.91 (0.85-0.96)	0.82 (0.77-0.87)	0.81 (0.76-0.86)	0.74 (0.69-0.79)	<0.0001
HR (95% CI), Model 2	Reference	0.95 (0.89-1.01)	0.86 (0.81-0.92)	0.87 (0.82-0.93)	0.80 (0.75-0.85)	<0.0001
Diverticular disease						
Events	1613	1668	1573	1596	1552	
Person-years	187128	189159	190264	190303	191041	
HR (95% CI), Model 1	Reference	0.97 (0.91-1.04)	0.88 (0.82-0.94)	0.87 (0.81-0.93)	0.82 (0.77-0.88)	<0.0001
HR (95% CI), Model 2	Reference	1.01 (0.94-1.08)	0.92 (0.86-0.99)	0.92 (0.85-0.98)	0.87 (0.81-0.94)	<0.0001
Pernicious anaemia						
Events	37	24	28	21	26	
Person-years	200237	202245	202498	202893	203335	
HR (95% CI), Model 1	Reference	0.62 (0.37-1.03)	0.71 (0.43-1.16)	0.52 (0.30-0.89)	0.63 (0.38-1.05)	0.13
HR (95% CI), Model 2	Reference	0.67 (0.40-1.12)	0.77 (0.47-1.27)	0.59 (0.34-1.01)	0.73 (0.43-1.22)	0.34
Fracture						
Events	137	142	147	146	187	
Person-years	199728	201368	201526	201878	202025	
HR (95% CI), Model 1	Reference	0.94 (0.74-1.19)	0.94 (0.74-1.18)	0.89 (0.70-1.12)	1.12 (0.90-1.40)	0.26
HR (95% CI), Model 2	Reference	0.90 (0.71-1.14)	0.88 (0.70-1.12)	0.82 (0.65-1.04)	1.02 (0.81-1.27)	0.29
Osteoporosis						
Events	1187	1240	1228	1261	1199	
Person-years	178374	181130	181072	180895	181934	
HR (95% CI), Model 1	Reference	0.96 (0.88-1.04)	0.91 (0.84-0.99)	0.91 (0.84-0.98)	0.84 (0.78-0.91)	0.0005
HR (95% CI), Model 2	Reference	1.04 (0.96-1.12)	1.02 (0.94-1.10)	1.04 (0.96-1.13)	0.99 (0.91-1.07)	0.63
Meniere’s disease						
Events	17	23	24	21	16	

Person-years	200507	202171	202510	202645	203075	
HR (95% CI), Model 1	Reference	1.29 (0.69-2.42)	1.33 (0.71-2.47)	1.14 (0.60-2.17)	0.86 (0.43-1.71)	0.65
HR (95% CI), Model 2	Reference	1.37 (0.73-2.57)	1.43 (0.76-2.68)	1.27 (0.66-2.42)	0.99 (0.49-1.99)	0.68
Eczema						
Events	205	247	231	229	220	
Person-years	191932	194023	193673	194669	195035	
HR (95% CI), Model 1	Reference	1.14 (0.95-1.37)	1.04 (0.86-1.26)	1.01 (0.83-1.22)	0.95 (0.79-1.15)	0.36
HR (95% CI), Model 2	Reference	1.19 (0.99-1.43)	1.09 (0.90-1.32)	1.06 (0.87-1.28)	1.00 (0.82-1.21)	0.29
Glaucoma						
Events	275	305	326	315	322	
Person-years	198363	199600	199659	199726	199802	
HR (95% CI), Model 1	Reference	1.01 (0.86-1.19)	1.02 (0.87-1.20)	0.94 (0.80-1.11)	0.94 (0.80-1.10)	0.71
HR (95% CI), Model 2	Reference	1.00 (0.85-1.18)	1.01 (0.86-1.19)	0.93 (0.79-1.10)	0.92 (0.78-1.09)	0.68
Cataract						
Events	1328	1466	1521	1579	1592	
Person-years	190741	191767	191558	191364	191468	
HR (95% CI), Model 1	Reference	0.98 (0.91-1.05)	0.96 (0.89-1.03)	0.94 (0.87-1.01)	0.92 (0.85-0.99)	0.17
HR (95% CI), Model 2	Reference	1.00 (0.93-1.08)	0.99 (0.92-1.07)	0.98 (0.91-1.06)	0.97 (0.90-1.04)	0.84
AMD						
Events	218	230	281	270	279	
Person-years	199986	201698	201608	201932	202191	
HR (95% CI), Model 1	Reference	0.93 (0.77-1.12)	1.08 (0.90-1.29)	0.97 (0.81-1.16)	0.98 (0.82-1.17)	0.55
HR (95% CI), Model 2	Reference	0.95 (0.79-1.15)	1.11 (0.93-1.33)	1.01 (0.84-1.21)	1.03 (0.86-1.23)	0.52
Thyroid disorders						
Events	469	436	452	411	339	
Person-years	186182	188418	189506	190376	191472	
HR (95% CI), Model 1	Reference	0.87 (0.77-1.00)	0.89 (0.78-1.01)	0.79 (0.69-0.90)	0.65 (0.56-0.75)	<0.0001
HR (95% CI), Model 2	Reference	0.92 (0.81-1.05)	0.95 (0.83-1.08)	0.86 (0.75-0.98)	0.71 (0.62-0.82)	<0.0001
Prostate disorders¶						
Events	597	644	710	696	663	
Person-years	85125	82111	83562	84017	87117	
HR (95% CI), Model 1	Reference	1.03 (0.92-1.15)	1.06 (0.95-1.18)	0.99 (0.88-1.10)	0.88 (0.79-0.99)	0.0105
HR (95% CI), Model 2	Reference	1.04 (0.93-1.16)	1.07 (0.96-1.19)	0.99 (0.89-1.11)	0.89 (0.79-1.00)	0.0135

AMD, age related macular degeneration; CI, confidence interval; CKD, chronic kidney disease; COPD, chronic obstructive pulmonary disease; HR, hazard ratio.

*Cox proportional hazard regression models were used to examine associations of the Anti-Empirical Dietary Inflammatory Index (quintiles) with the incidence of individual chronic diseases. The statistical tests were two-sided. Model 1 was adjusted for age and sex; Model 2 was adjusted for Model 1 plus ethnicity, education, income, smoking, alcohol consumption, sleep, physical activity, and GRS for longevity; Model 3 was adjusted for Model 2 and total energy intake.

†All cancers encompass any type of cancer except for non-melanoma skin cancer.

‡These analyses were conducted among women only.

¶These analyses were conducted among men only.

Table S3. The association between the Alternate Healthy Eating Index-2010 and the incidence of individual chronic diseases

	Alternate Healthy Eating Index-2010					P-value
	Quintile 1	Quintile 2	Quintile 3	Quintile 4	Quintile 5	for trend*
Cardiovascular disease†						
Events	1975	1833	1792	1661	1664	
Person-years	185979	188764	189463	191239	191769	
HR (95% CI), Model 1	Reference	0.89 (0.84-0.95)	0.85 (0.80-0.91)	0.78 (0.73-0.84)	0.79 (0.73-0.84)	<0.0001
HR (95% CI), Model 2	Reference	0.92 (0.87-0.98)	0.89 (0.84-0.95)	0.83 (0.78-0.89)	0.84 (0.79-0.90)	<0.0001
Coronary heart disease						
Events	1079	995	969	867	910	
Person-years	193741	195960	196214	197460	198296	
HR (95% CI), Model 1	Reference	0.91 (0.83-0.99)	0.87 (0.80-0.95)	0.79 (0.72-0.86)	0.84 (0.76-0.91)	<0.0001
HR (95% CI), Model 2	Reference	0.95 (0.87-1.03)	0.93 (0.85-1.01)	0.85 (0.78-0.93)	0.91 (0.83-1.00)	0.0116
Heart failure						
Events	350	303	297	292	276	
Person-years	199551	201424	201444	202248	202966	
HR (95% CI), Model 1	Reference	0.83 (0.71-0.97)	0.79 (0.68-0.92)	0.78 (0.66-0.91)	0.73 (0.62-0.85)	0.0010
HR (95% CI), Model 2	Reference	0.88 (0.76-1.03)	0.87 (0.74-1.01)	0.86 (0.73-1.01)	0.82 (0.70-0.97)	0.16
Atrial fibrillation						
Events	411	395	360	327	338	
Person-years	196643	198694	199093	199827	200397	
HR (95% CI), Model 1	Reference	0.93 (0.81-1.06)	0.82 (0.71-0.95)	0.75 (0.65-0.87)	0.78 (0.67-0.90)	0.0003
HR (95% CI), Model 2	Reference	0.96 (0.84-1.11)	0.87 (0.76-1.01)	0.80 (0.69-0.93)	0.85 (0.73-0.98)	0.0204
Other cardiac disease						
Events	828	751	691	648	668	
Person-years	197059	198933	199286	200478	200839	
HR (95% CI), Model 1	Reference	0.87 (0.79-0.96)	0.78 (0.70-0.86)	0.72 (0.65-0.80)	0.74 (0.67-0.82)	<0.0001
HR (95% CI), Model 2	Reference	0.91 (0.82-1.00)	0.82 (0.74-0.91)	0.78 (0.70-0.86)	0.80 (0.72-0.89)	<0.0001
Stroke						
Events	164	152	166	129	108	
Person-years	199593	201177	201529	202315	203033	
HR (95% CI), Model 1	Reference	0.89 (0.71-1.11)	0.94 (0.76-1.17)	0.73 (0.58-0.91)	0.60 (0.47-0.77)	0.0002
HR (95% CI), Model 2	Reference	0.92 (0.73-1.14)	0.98 (0.79-1.21)	0.76 (0.60-0.96)	0.63 (0.49-0.81)	0.0009
Peripheral vascular disease						
Events	200	191	149	172	158	
Person-years	199831	201553	201931	202589	203044	
HR (95% CI), Model 1	Reference	0.92 (0.75-1.12)	0.70 (0.56-0.86)	0.80 (0.65-0.98)	0.73 (0.59-0.90)	0.0029
HR (95% CI), Model 2	Reference	0.95 (0.78-1.16)	0.72 (0.58-0.89)	0.84 (0.68-1.03)	0.75 (0.61-0.93)	0.0092
Hypertension						
Events	923	805	771	680	703	
Person-years	146130	149622	150963	152234	153502	
HR (95% CI), Model 1	Reference	0.83 (0.75-0.91)	0.77 (0.70-0.84)	0.67 (0.61-0.74)	0.68 (0.62-0.75)	<0.0001

HR (95% CI), Model 2	Reference	0.88 (0.80-0.97)	0.83 (0.75-0.91)	0.74 (0.67-0.82)	0.77 (0.70-0.85)	<0.0001
Diabetes						
Events	599	491	460	476	425	
Person-years	192079	194081	194613	194307	194977	
HR (95% CI), Model 1	Reference	0.81 (0.72-0.91)	0.75 (0.67-0.85)	0.79 (0.70-0.89)	0.71 (0.63-0.80)	<0.0001
HR (95% CI), Model 2	Reference	0.91 (0.81-1.02)	0.90 (0.79-1.01)	0.96 (0.85-1.08)	0.88 (0.78-1.00)	0.25
All cancers‡						
Events	1351	1199	1207	1165	1127	
Person-years	180774	184901	184419	185453	186311	
HR (95% CI), Model 1	Reference	0.83 (0.77-0.89)	0.81 (0.75-0.88)	0.77 (0.72-0.84)	0.73 (0.68-0.80)	<0.0001
HR (95% CI), Model 2	Reference	0.86 (0.79-0.93)	0.85 (0.79-0.92)	0.82 (0.76-0.89)	0.79 (0.73-0.86)	<0.0001
Non-melanoma skin cancer						
Events	725	720	716	718	680	
Person-years	195365	197061	196938	197728	198444	
HR (95% CI), Model 1	Reference	0.95 (0.86-1.05)	0.92 (0.83-1.02)	0.92 (0.83-1.02)	0.86 (0.77-0.96)	0.0821
HR (95% CI), Model 2	Reference	0.92 (0.83-1.03)	0.89 (0.80-0.98)	0.88 (0.80-0.98)	0.81 (0.73-0.91)	0.0049
Melanoma						
Events	105	80	98	96	106	
Person-years	198830	200547	200443	201177	201840	
HR (95% CI), Model 1	Reference	0.73 (0.55-0.98)	0.88 (0.67-1.16)	0.86 (0.65-1.13)	0.93 (0.71-1.23)	0.31
HR (95% CI), Model 2	Reference	0.73 (0.54-0.97)	0.88 (0.67-1.16)	0.86 (0.65-1.14)	0.95 (0.72-1.25)	0.26
Lung cancer						
Events	171	114	114	110	100	
Person-years	200269	202205	202288	203001	203593	
HR (95% CI), Model 1	Reference	0.62 (0.49-0.78)	0.59 (0.47-0.75)	0.56 (0.44-0.71)	0.49 (0.38-0.63)	<0.0001
HR (95% CI), Model 2	Reference	0.69 (0.54-0.87)	0.68 (0.53-0.86)	0.67 (0.52-0.85)	0.60 (0.47-0.78)	0.0003
Stomach cancer						
Events	30	34	27	29	25	
Person-years	200961	202536	202660	203301	203965	
HR (95% CI), Model 1	Reference	1.13 (0.69-1.85)	0.89 (0.53-1.50)	0.97 (0.58-1.62)	0.85 (0.50-1.45)	0.84
HR (95% CI), Model 2	Reference	1.19 (0.72-1.94)	0.96 (0.57-1.63)	1.06 (0.63-1.78)	0.95 (0.55-1.63)	0.91
Oesophageal cancer						
Events	51	38	40	31	34	
Person-years	200803	202548	202616	203339	203962	
HR (95% CI), Model 1	Reference	0.73 (0.48-1.10)	0.75 (0.50-1.14)	0.59 (0.37-0.92)	0.64 (0.42-1.00)	0.15
HR (95% CI), Model 2	Reference	0.79 (0.52-1.21)	0.84 (0.55-1.28)	0.67 (0.43-1.06)	0.77 (0.49-1.20)	0.51
Colon cancer						
Events	153	164	177	158	129	
Person-years	199352	201355	201219	201962	202831	
HR (95% CI), Model 1	Reference	1.02 (0.82-1.28)	1.08 (0.87-1.34)	0.96 (0.76-1.20)	0.77 (0.61-0.97)	0.0480
HR (95% CI), Model 2	Reference	1.06 (0.85-1.32)	1.13 (0.91-1.41)	1.01 (0.81-1.26)	0.83 (0.65-1.05)	0.0993
Ovarian cancer§						
Events	41	51	39	38	52	
Person-years	100614	114245	119945	126580	134165	

HR (95% CI), Model 1	Reference	1.04 (0.69-1.57)	0.74 (0.48-1.15)	0.67 (0.43-1.04)	0.83 (0.55-1.26)	0.19
HR (95% CI), Model 2	Reference	1.06 (0.70-1.60)	0.75 (0.48-1.17)	0.68 (0.43-1.06)	0.85 (0.56-1.29)	0.21
Breast cancer[§]						
Events	371	363	395	362	415	
Person-years	94298	107806	113092	119416	125822	
HR (95% CI), Model 1	Reference	0.84 (0.73-0.97)	0.86 (0.75-1.00)	0.74 (0.64-0.86)	0.80 (0.70-0.92)	0.0015
HR (95% CI), Model 2	Reference	0.85 (0.74-0.99)	0.88 (0.77-1.02)	0.77 (0.66-0.89)	0.83 (0.72-0.96)	0.0081
Prostate cancer[¶]						
Events	446	446	472	383	334	
Person-years	108177	94742	89378	83710	76755	
HR (95% CI), Model 1	Reference	1.07 (0.93-1.22)	1.16 (1.02-1.32)	0.99 (0.86-1.14)	0.93 (0.81-1.07)	0.0234
HR (95% CI), Model 2	Reference	1.05 (0.92-1.19)	1.13 (0.99-1.28)	0.97 (0.84-1.11)	0.90 (0.78-1.04)	0.0266
Other cancers						
Events	1166	1060	1016	987	960	
Person-years	184373	187973	187956	188854	189471	
HR (95% CI), Model 1	Reference	0.85 (0.78-0.93)	0.80 (0.73-0.87)	0.76 (0.70-0.83)	0.73 (0.67-0.80)	<0.0001
HR (95% CI), Model 2	Reference	0.88 (0.81-0.96)	0.83 (0.77-0.91)	0.81 (0.74-0.88)	0.78 (0.72-0.85)	<0.0001
Depression						
Events	237	202	172	165	219	
Person-years	188377	190894	191631	192498	192269	
HR (95% CI), Model 1	Reference	0.81 (0.67-0.98)	0.68 (0.56-0.83)	0.64 (0.52-0.78)	0.84 (0.69-1.01)	0.0001
HR (95% CI), Model 2	Reference	0.86 (0.71-1.04)	0.73 (0.60-0.89)	0.69 (0.57-0.85)	0.90 (0.74-1.09)	0.0014
Anxiety						
Events	623	570	554	546	628	
Person-years	194718	196901	197133	197871	197698	
HR (95% CI), Model 1	Reference	0.86 (0.76-0.96)	0.81 (0.72-0.91)	0.78 (0.69-0.87)	0.87 (0.77-0.97)	0.0002
HR (95% CI), Model 2	Reference	0.89 (0.79-1.00)	0.85 (0.76-0.95)	0.82 (0.73-0.92)	0.91 (0.81-1.02)	0.0109
Schizophrenia						
Events	31	23	22	19	20	
Person-years	200459	202047	202111	202854	203472	
HR (95% CI), Model 1	Reference	0.72 (0.42-1.23)	0.68 (0.39-1.17)	0.57 (0.32-1.01)	0.58 (0.33-1.03)	0.27
HR (95% CI), Model 2	Reference	0.80 (0.46-1.37)	0.77 (0.44-1.34)	0.67 (0.37-1.19)	0.70 (0.39-1.24)	0.65
Alcohol use disorder						
Events	292	167	122	112	62	
Person-years	198894	201402	201830	202589	203446	
HR (95% CI), Model 1	Reference	0.58 (0.48-0.71)	0.43 (0.35-0.53)	0.40 (0.32-0.50)	0.22 (0.17-0.30)	<0.0001
HR (95% CI), Model 2	Reference	0.62 (0.51-0.75)	0.47 (0.38-0.58)	0.45 (0.36-0.56)	0.26 (0.20-0.34)	<0.0001
Psychoactive substance abuse						
Events	58	34	17	20	16	
Person-years	200532	202425	202551	203294	203817	
HR (95% CI), Model 1	Reference	0.61 (0.40-0.93)	0.31 (0.18-0.54)	0.37 (0.22-0.63)	0.31 (0.18-0.54)	<0.0001
HR (95% CI), Model 2	Reference	0.67 (0.44-1.03)	0.35 (0.20-0.60)	0.43 (0.26-0.72)	0.36 (0.20-0.63)	<0.0001
Epilepsy						
Events	93	69	78	56	70	

Person-years	199349	201165	200999	201892	202336	
HR (95% CI), Model 1	Reference	0.72 (0.53-0.98)	0.80 (0.59-1.09)	0.57 (0.41-0.80)	0.71 (0.52-0.98)	0.0190
HR (95% CI), Model 2	Reference	0.74 (0.54-1.01)	0.83 (0.61-1.13)	0.60 (0.43-0.84)	0.75 (0.55-1.04)	0.0423
Migraine						
Events	137	119	132	129	127	
Person-years	194886	196072	195558	195500	195668	
HR (95% CI), Model 1	Reference	0.83 (0.65-1.06)	0.91 (0.72-1.16)	0.88 (0.69-1.12)	0.85 (0.66-1.08)	0.59
HR (95% CI), Model 2	Reference	0.84 (0.66-1.07)	0.93 (0.73-1.18)	0.89 (0.70-1.14)	0.86 (0.67-1.09)	0.64
Dementia						
Events	159	122	133	114	145	
Person-years	200569	202463	202485	203224	203686	
HR (95% CI), Model 1	Reference	0.70 (0.55-0.89)	0.72 (0.58-0.91)	0.61 (0.48-0.77)	0.74 (0.59-0.94)	0.0009
HR (95% CI), Model 2	Reference	0.71 (0.56-0.90)	0.74 (0.58-0.93)	0.61 (0.48-0.78)	0.75 (0.59-0.94)	0.0016
Parkinson’s disease						
Events	83	78	69	80	79	
Person-years	200607	202318	202253	202965	203711	
HR (95% CI), Model 1	Reference	0.90 (0.66-1.22)	0.77 (0.56-1.06)	0.90 (0.66-1.22)	0.88 (0.64-1.20)	0.64
HR (95% CI), Model 2	Reference	0.89 (0.65-1.21)	0.76 (0.55-1.05)	0.88 (0.64-1.19)	0.85 (0.62-1.17)	0.57
Multiple sclerosis						
Events	15	34	18	13	14	
Person-years	200096	201869	202069	202794	203244	
HR (95% CI), Model 1	Reference	2.22 (1.21-4.08)	1.17 (0.59-2.33)	0.84 (0.40-1.77)	0.89 (0.43-1.86)	0.0040
HR (95% CI), Model 2	Reference	2.31 (1.25-4.24)	1.23 (0.62-2.46)	0.90 (0.42-1.89)	0.97 (0.46-2.03)	0.0050
Bronchiectasis						
Events	157	134	115	113	129	
Person-years	199976	201808	201833	202449	203193	
HR (95% CI), Model 1	Reference	0.77 (0.61-0.97)	0.63 (0.49-0.80)	0.59 (0.47-0.76)	0.65 (0.51-0.82)	0.0001
HR (95% CI), Model 2	Reference	0.79 (0.63-1.00)	0.65 (0.51-0.82)	0.61 (0.48-0.79)	0.66 (0.52-0.83)	0.0002
Asthma						
Events	386	317	322	325	306	
Person-years	171799	174318	175545	175909	177223	
HR (95% CI), Model 1	Reference	0.78 (0.67-0.91)	0.77 (0.67-0.90)	0.77 (0.66-0.89)	0.71 (0.61-0.82)	0.0001
HR (95% CI), Model 2	Reference	0.82 (0.70-0.95)	0.82 (0.70-0.95)	0.82 (0.70-0.95)	0.75 (0.64-0.88)	0.0038
COPD						
Events	465	362	353	298	276	
Person-years	196806	198859	199420	200516	201113	
HR (95% CI), Model 1	Reference	0.73 (0.64-0.84)	0.69 (0.60-0.79)	0.58 (0.50-0.67)	0.52 (0.45-0.61)	<0.0001
HR (95% CI), Model 2	Reference	0.83 (0.73-0.96)	0.81 (0.71-0.94)	0.71 (0.61-0.82)	0.66 (0.57-0.77)	<0.0001
CKD						
Events	901	887	790	752	728	
Person-years	197000	198486	199168	200487	200685	
HR (95% CI), Model 1	Reference	0.94 (0.85-1.03)	0.80 (0.73-0.88)	0.76 (0.69-0.83)	0.72 (0.65-0.80)	<0.0001
HR (95% CI), Model 2	Reference	0.99 (0.90-1.09)	0.87 (0.79-0.96)	0.83 (0.75-0.91)	0.80 (0.73-0.89)	<0.0001
Chronic liver disease						
Events	62	57	43	39	35	

Person-years	199758	201484	201741	202329	203022	
HR (95% CI), Model 1	Reference	0.92 (0.64-1.32)	0.69 (0.47-1.02)	0.63 (0.42-0.95)	0.57 (0.38-0.87)	0.0289
HR (95% CI), Model 2	Reference	1.00 (0.70-1.44)	0.80 (0.54-1.18)	0.75 (0.50-1.12)	0.71 (0.46-1.08)	0.30
Irritable bowel syndrome						
Events	254	225	190	207	224	
Person-years	195353	196492	197182	197302	197574	
HR (95% CI), Model 1	Reference	0.82 (0.69-0.99)	0.67 (0.56-0.81)	0.71 (0.59-0.86)	0.74 (0.62-0.89)	0.0002
HR (95% CI), Model 2	Reference	0.86 (0.71-1.02)	0.70 (0.58-0.85)	0.75 (0.63-0.91)	0.79 (0.66-0.95)	0.0030
Inflammatory bowel disease						
Events	97	97	87	89	78	
Person-years	198178	200167	200722	201393	202195	
HR (95% CI), Model 1	Reference	1.00 (0.75-1.32)	0.89 (0.67-1.19)	0.91 (0.68-1.22)	0.80 (0.59-1.08)	0.58
HR (95% CI), Model 2	Reference	1.03 (0.78-1.37)	0.93 (0.69-1.24)	0.95 (0.71-1.27)	0.82 (0.61-1.11)	0.64
Treated constipation						
Events	731	681	630	608	684	
Person-years	195725	198309	198284	199153	199490	
HR (95% CI), Model 1	Reference	0.88 (0.79-0.97)	0.79 (0.71-0.88)	0.75 (0.67-0.84)	0.83 (0.75-0.92)	<0.0001
HR (95% CI), Model 2	Reference	0.90 (0.81-0.99)	0.81 (0.73-0.90)	0.77 (0.69-0.86)	0.84 (0.76-0.94)	<0.0001
Dyspepsia						
Events	2110	1983	1867	1922	1933	
Person-years	168531	172416	174562	174771	175249	
HR (95% CI), Model 1	Reference	0.89 (0.83-0.94)	0.81 (0.76-0.86)	0.82 (0.77-0.87)	0.81 (0.76-0.86)	<0.0001
HR (95% CI), Model 2	Reference	0.91 (0.86-0.97)	0.84 (0.79-0.89)	0.86 (0.81-0.92)	0.85 (0.80-0.90)	<0.0001
Diverticular disease						
Events	1764	1647	1602	1524	1465	
Person-years	186274	188682	189878	190955	192106	
HR (95% CI), Model 1	Reference	0.89 (0.83-0.95)	0.84 (0.78-0.90)	0.79 (0.73-0.84)	0.74 (0.69-0.79)	<0.0001
HR (95% CI), Model 2	Reference	0.92 (0.86-0.99)	0.89 (0.83-0.95)	0.85 (0.79-0.91)	0.81 (0.75-0.87)	<0.0001
Pernicious anaemia						
Events	38	25	29	22	22	
Person-years	200397	202187	202289	202947	203388	
HR (95% CI), Model 1	Reference	0.61 (0.37-1.02)	0.69 (0.42-1.12)	0.51 (0.30-0.86)	0.49 (0.29-0.83)	0.0411
HR (95% CI), Model 2	Reference	0.64 (0.38-1.06)	0.72 (0.44-1.18)	0.54 (0.32-0.92)	0.52 (0.30-0.89)	0.0841
Fracture						
Events	154	133	148	149	175	
Person-years	199421	201325	201343	202014	202423	
HR (95% CI), Model 1	Reference	0.79 (0.62-0.99)	0.83 (0.66-1.05)	0.81 (0.65-1.02)	0.92 (0.74-1.14)	0.21622
HR (95% CI), Model 2	Reference	0.78 (0.62-0.98)	0.82 (0.65-1.03)	0.80 (0.64-1.01)	0.89 (0.71-1.11)	0.19869
Osteoporosis						
Events	1316	1245	1215	1158	1181	
Person-years	178732	180860	180821	180929	182064	
HR (95% CI), Model 1	Reference	0.88 (0.81-0.95)	0.82 (0.76-0.89)	0.77 (0.71-0.83)	0.76 (0.70-0.82)	<0.0001
HR (95% CI), Model 2	Reference	0.91 (0.85-0.99)	0.88 (0.81-0.95)	0.83 (0.77-0.90)	0.83 (0.76-0.90)	<0.0001
Meniere’s disease						

Events	26	19	10	22	24	
Person-years	200357	202162	202203	202752	203434	
HR (95% CI), Model 1	Reference	0.71 (0.39-1.28)	0.37 (0.18-0.76)	0.80 (0.45-1.42)	0.86 (0.49-1.51)	0.10
HR (95% CI), Model 2	Reference	0.71 (0.39-1.29)	0.38 (0.18-0.78)	0.82 (0.46-1.46)	0.89 (0.50-1.57)	0.11
Eczema						
Events	284	231	209	195	213	
Person-years	191575	193782	193944	194982	195050	
HR (95% CI), Model 1	Reference	0.78 (0.66-0.93)	0.69 (0.58-0.83)	0.64 (0.53-0.77)	0.69 (0.58-0.83)	<0.0001
HR (95% CI), Model 2	Reference	0.80 (0.67-0.95)	0.72 (0.60-0.87)	0.67 (0.56-0.81)	0.72 (0.60-0.86)	<0.0001
Glaucoma						
Events	274	298	285	338	348	
Person-years	197921	199394	199476	199945	200413	
HR (95% CI), Model 1	Reference	1.03 (0.87-1.21)	0.95 (0.80-1.12)	1.11 (0.95-1.31)	1.12 (0.96-1.32)	0.17
HR (95% CI), Model 2	Reference	1.02 (0.87-1.20)	0.94 (0.79-1.11)	1.09 (0.93-1.29)	1.09 (0.93-1.28)	0.29
Cataract						
Events	1426	1458	1466	1541	1595	
Person-years	190409	191791	191560	191782	191356	
HR (95% CI), Model 1	Reference	0.92 (0.86-0.99)	0.88 (0.82-0.95)	0.90 (0.84-0.97)	0.90 (0.84-0.97)	0.0080
HR (95% CI), Model 2	Reference	0.94 (0.87-1.01)	0.90 (0.84-0.97)	0.92 (0.86-0.99)	0.91 (0.85-0.98)	0.0500
AMD						
Events	243	245	252	259	279	
Person-years	199842	201480	201539	202036	202518	
HR (95% CI), Model 1	Reference	0.91 (0.76-1.09)	0.88 (0.74-1.06)	0.88 (0.74-1.05)	0.90 (0.76-1.08)	0.63
HR (95% CI), Model 2	Reference	0.93 (0.78-1.11)	0.91 (0.76-1.08)	0.91 (0.76-1.08)	0.92 (0.77-1.10)	0.81
Thyroid disorders						
Events	401	365	432	465	444	
Person-years	188945	189977	189348	189131	188552	
HR (95% CI), Model 1	Reference	0.84 (0.73-0.97)	0.97 (0.85-1.11)	1.02 (0.89-1.17)	0.94 (0.82-1.08)	0.0685
HR (95% CI), Model 2	Reference	0.87 (0.76-1.00)	1.02 (0.89-1.17)	1.08 (0.94-1.24)	1.02 (0.89-1.17)	0.0423
Prostate disorders[¶]						
Events	782	711	627	647	543	
Person-years	101029	88425	83710	77718	71051	
HR (95% CI), Model 1	Reference	0.97 (0.87-1.07)	0.86 (0.78-0.96)	0.95 (0.86-1.06)	0.86 (0.77-0.96)	0.0175
HR (95% CI), Model 2	Reference	0.97 (0.88-1.08)	0.87 (0.78-0.97)	0.96 (0.87-1.07)	0.87 (0.78-0.98)	0.0271

AMD, age related macular degeneration; CI, confidence interval; CKD, chronic kidney disease; COPD, chronic obstructive pulmonary disease; HR, hazard ratio.

*Cox proportional hazard regression models were used to examine associations of the Alternate Healthy Eating Index-2010 (quintiles) with the incidence of individual chronic diseases. The statistical tests were two-sided. Model 1 was adjusted for age and sex; Model 2 was adjusted for Model 1 plus ethnicity, education, income, smoking, alcohol consumption, sleep, physical activity, and GRS for longevity; Model 3 was adjusted for Model 2 and total energy intake.

[†]Cardiovascular disease includes coronary heart disease, heart failure, atrial fibrillation, other cardiac disease, stroke, and peripheral vascular disease.

[‡]All cancers encompass any type of cancer except for non-melanoma skin cancer.

[§]These analyses were conducted among women only.

[¶]These analyses were conducted among men only.

Table S4. The association between the Healthful Plant-based Diet Index and the incidence of individual chronic diseases

	Healthful Plant-based Diet Index					P-value
	Quintile 1	Quintile 2	Quintile 3	Quintile 4	Quintile 5	for trend*
Cardiovascular disease†						
Events	2470	663	2252	1980	1560	
Person-years	230757	69265	240120	213020	194051	
HR (95% CI), Model 1	Reference	0.87 (0.80-0.95)	0.86 (0.81-0.91)	0.88 (0.83-0.93)	0.79 (0.74-0.84)	<0.0001
HR (95% CI), Model 2	Reference	0.90 (0.82-0.98)	0.90 (0.85-0.96)	0.93 (0.87-0.99)	0.86 (0.80-0.92)	<0.0001
Coronary heart disease						
Events	1406	366	1232	1018	798	
Person-years	240278	72006	248623	220643	200121	
HR (95% CI), Model 1	Reference	0.86 (0.76-0.96)	0.85 (0.79-0.92)	0.83 (0.76-0.90)	0.75 (0.69-0.82)	<0.0001
HR (95% CI), Model 2	Reference	0.89 (0.79-1.00)	0.90 (0.83-0.97)	0.89 (0.82-0.96)	0.83 (0.76-0.91)	0.0015
Heart failure						
Events	437	127	398	311	245	
Person-years	248028	73946	255500	225690	204469	
HR (95% CI), Model 1	Reference	0.95 (0.78-1.15)	0.87 (0.76-0.99)	0.79 (0.68-0.92)	0.72 (0.62-0.85)	0.0009
HR (95% CI), Model 2	Reference	1.00 (0.82-1.22)	0.95 (0.83-1.09)	0.88 (0.76-1.03)	0.85 (0.72-1.00)	0.28
Atrial fibrillation						
Events	513	133	459	434	292	
Person-years	244230	72982	252387	222868	202187	
HR (95% CI), Model 1	Reference	0.86 (0.71-1.04)	0.87 (0.77-0.99)	0.98 (0.86-1.12)	0.79 (0.68-0.91)	0.0067
HR (95% CI), Model 2	Reference	0.88 (0.73-1.07)	0.93 (0.82-1.05)	1.06 (0.93-1.21)	0.88 (0.75-1.02)	0.0620
Other cardiac disease						
Events	981	288	911	769	637	
Person-years	244750	73086	252674	223556	202529	
HR (95% CI), Model 1	Reference	0.95 (0.84-1.09)	0.87 (0.80-0.96)	0.85 (0.77-0.94)	0.81 (0.73-0.89)	0.0003
HR (95% CI), Model 2	Reference	0.98 (0.86-1.12)	0.92 (0.84-1.01)	0.90 (0.82-1.00)	0.88 (0.79-0.97)	0.0998
Stroke						
Events	203	51	171	180	114	
Person-years	247943	74066	255763	225624	204251	
HR (95% CI), Model 1	Reference	0.81 (0.60-1.11)	0.79 (0.64-0.97)	0.96 (0.78-1.18)	0.70 (0.55-0.88)	0.0126
HR (95% CI), Model 2	Reference	0.83 (0.61-1.13)	0.82 (0.67-1.00)	1.00 (0.82-1.23)	0.74 (0.58-0.94)	0.0376
Peripheral vascular disease						
Events	239	62	211	198	160	
Person-years	248376	74130	255957	226014	204470	
HR (95% CI), Model 1	Reference	0.83 (0.63-1.10)	0.81 (0.67-0.98)	0.86 (0.71-1.04)	0.77 (0.63-0.95)	0.11
HR (95% CI), Model 2	Reference	0.85 (0.64-1.13)	0.83 (0.69-1.00)	0.89 (0.73-1.08)	0.80 (0.65-0.99)	0.24
Hypertension						
Events	1089	304	1020	813	656	
Person-years	178278	54204	189506	170878	159585	
HR (95% CI), Model 1	Reference	0.88 (0.78-1.00)	0.84 (0.77-0.92)	0.75 (0.68-0.82)	0.66 (0.59-0.72)	<0.0001
HR (95% CI), Model 2	Reference	0.92 (0.81-1.05)	0.91 (0.83-0.99)	0.83 (0.75-0.91)	0.76 (0.69-0.84)	<0.0001

Diabetes						
Events	795	210	648	427	371	
Person-years	236759	71291	246109	217844	198054	
HR (95% CI), Model 1	Reference	0.86 (0.74-1.01)	0.78 (0.70-0.87)	0.59 (0.52-0.66)	0.58 (0.51-0.65)	<0.0001
HR (95% CI), Model 2	Reference	0.96 (0.82-1.11)	0.92 (0.83-1.02)	0.73 (0.65-0.83)	0.79 (0.69-0.90)	<0.0001
All cancers‡						
Events	1625	458	1557	1314	1095	
Person-years	226058	67691	233641	206340	188128	
HR (95% CI), Model 1	Reference	0.90 (0.81-1.00)	0.87 (0.81-0.94)	0.82 (0.77-0.89)	0.76 (0.70-0.82)	<0.0001
HR (95% CI), Model 2	Reference	0.92 (0.83-1.02)	0.90 (0.84-0.97)	0.86 (0.80-0.93)	0.81 (0.75-0.88)	<0.0001
Non-melanoma skin cancer						
Events	868	280	937	790	684	
Person-years	242689	72560	249674	220721	199893	
HR (95% CI), Model 1	Reference	1.04 (0.91-1.19)	1.01 (0.92-1.11)	0.98 (0.89-1.08)	0.96 (0.87-1.07)	0.79
HR (95% CI), Model 2	Reference	1.03 (0.90-1.17)	0.98 (0.90-1.08)	0.94 (0.85-1.04)	0.91 (0.82-1.01)	0.32
Melanoma						
Events	121	36	132	94	102	
Person-years	247378	73698	254481	224213	203066	
HR (95% CI), Model 1	Reference	0.97 (0.67-1.41)	1.03 (0.80-1.32)	0.83 (0.63-1.10)	1.02 (0.77-1.34)	0.57
HR (95% CI), Model 2	Reference	0.97 (0.67-1.41)	1.02 (0.80-1.31)	0.83 (0.63-1.09)	1.01 (0.76-1.33)	0.57
Lung cancer						
Events	161	61	148	131	108	
Person-years	249099	74221	256530	226530	204976	
HR (95% CI), Model 1	Reference	1.19 (0.88-1.60)	0.81 (0.65-1.02)	0.79 (0.63-1.01)	0.72 (0.56-0.93)	0.0079
HR (95% CI), Model 2	Reference	1.27 (0.94-1.70)	0.87 (0.69-1.09)	0.87 (0.69-1.11)	0.82 (0.63-1.05)	0.0577
Stomach cancer						
Events	41	8	42	29	25	
Person-years	249633	74476	257082	226906	205328	
HR (95% CI), Model 1	Reference	0.66 (0.31-1.41)	1.05 (0.68-1.61)	0.88 (0.54-1.42)	0.91 (0.54-1.52)	0.78
HR (95% CI), Model 2	Reference	0.69 (0.32-1.48)	1.12 (0.73-1.73)	0.96 (0.59-1.56)	1.03 (0.61-1.73)	0.80
Oesophageal cancer						
Events	59	11	48	41	35	
Person-years	249529	74468	257003	226936	205331	
HR (95% CI), Model 1	Reference	0.61 (0.32-1.17)	0.79 (0.54-1.16)	0.79 (0.53-1.19)	0.80 (0.52-1.23)	0.52
HR (95% CI), Model 2	Reference	0.66 (0.35-1.25)	0.88 (0.60-1.29)	0.91 (0.61-1.37)	0.97 (0.62-1.51)	0.76
Colon cancer						
Events	220	56	210	176	119	
Person-years	247684	73914	255151	225589	204381	
HR (95% CI), Model 1	Reference	0.83 (0.62-1.11)	0.90 (0.75-1.09)	0.87 (0.71-1.06)	0.67 (0.53-0.84)	0.0169
HR (95% CI), Model 2	Reference	0.85 (0.64-1.14)	0.94 (0.77-1.14)	0.91 (0.74-1.12)	0.72 (0.57-0.91)	0.0763
Ovarian cancer§						
Events	51	20	61	50	39	
Person-years	113280	38737	148175	147597	147761	
HR (95% CI), Model 1	Reference	1.12 (0.67-1.88)	0.88 (0.61-1.28)	0.72 (0.49-1.07)	0.57 (0.37-0.87)	0.0428

HR (95% CI), Model 2	Reference	1.16 (0.69-1.95)	0.92 (0.63-1.34)	0.77 (0.52-1.15)	0.63 (0.41-0.96)	0.12
Breast cancer[§]						
Events	381	132	479	480	434	
Person-years	106699	36683	139329	138560	139162	
HR (95% CI), Model 1	Reference	1.00 (0.82-1.22)	0.95 (0.83-1.09)	0.96 (0.84-1.10)	0.87 (0.75-1.00)	0.31
HR (95% CI), Model 2	Reference	1.02 (0.83-1.24)	0.98 (0.85-1.12)	1.00 (0.87-1.15)	0.91 (0.79-1.06)	0.66
Prostate cancer[¶]						
Events	621	163	573	443	281	
Person-years	145725	38474	117898	86702	63963	
HR (95% CI), Model 1	Reference	0.95 (0.80-1.13)	1.07 (0.95-1.19)	1.12 (0.99-1.27)	0.96 (0.83-1.11)	0.15
HR (95% CI), Model 2	Reference	0.94 (0.79-1.12)	1.04 (0.93-1.17)	1.09 (0.97-1.24)	0.93 (0.80-1.07)	0.18
Other cancers						
Events	1411	398	1317	1117	946	
Person-years	230254	69087	237832	210347	191108	
HR (95% CI), Model 1	Reference	0.90 (0.80-1.00)	0.85 (0.78-0.91)	0.80 (0.74-0.87)	0.75 (0.69-0.82)	<0.0001
HR (95% CI), Model 2	Reference	0.92 (0.82-1.02)	0.88 (0.81-0.94)	0.84 (0.77-0.91)	0.80 (0.73-0.87)	<0.0001
Depression						
Events	259	85	250	244	157	
Person-years	234715	70218	242202	214221	194314	
HR (95% CI), Model 1	Reference	1.06 (0.83-1.36)	0.88 (0.74-1.05)	0.94 (0.79-1.12)	0.65 (0.53-0.79)	0.0002
HR (95% CI), Model 2	Reference	1.13 (0.88-1.44)	0.96 (0.80-1.14)	1.04 (0.87-1.25)	0.74 (0.60-0.91)	0.0062
Anxiety						
Events	740	222	755	656	548	
Person-years	242190	72401	249546	220518	199666	
HR (95% CI), Model 1	Reference	0.96 (0.82-1.11)	0.91 (0.82-1.01)	0.86 (0.77-0.96)	0.77 (0.69-0.86)	0.0002
HR (95% CI), Model 2	Reference	0.99 (0.85-1.15)	0.96 (0.87-1.07)	0.92 (0.83-1.03)	0.84 (0.75-0.95)	0.0455
Schizophrenia						
Events	26	8	35	29	17	
Person-years	249085	74304	256375	226368	204812	
HR (95% CI), Model 1	Reference	1.07 (0.48-2.37)	1.38 (0.83-2.31)	1.34 (0.78-2.31)	0.91 (0.48-1.71)	0.51
HR (95% CI), Model 2	Reference	1.19 (0.54-2.64)	1.62 (0.97-2.72)	1.67 (0.96-2.88)	1.22 (0.64-2.31)	0.29
Alcohol use disorder						
Events	233	66	213	141	102	
Person-years	247877	74017	255560	226055	204653	
HR (95% CI), Model 1	Reference	1.00 (0.76-1.31)	0.98 (0.81-1.18)	0.79 (0.63-0.97)	0.68 (0.53-0.87)	0.0065
HR (95% CI), Model 2	Reference	1.02 (0.78-1.34)	1.01 (0.84-1.22)	0.83 (0.67-1.02)	0.73 (0.57-0.93)	0.0333
Psychoactive substance abuse						
Events	40	16	39	26	24	
Person-years	249369	74442	256781	226842	205185	
HR (95% CI), Model 1	Reference	1.41 (0.79-2.52)	1.04 (0.66-1.62)	0.83 (0.50-1.37)	0.89 (0.52-1.50)	0.54
HR (95% CI), Model 2	Reference	1.49 (0.83-2.67)	1.12 (0.71-1.75)	0.91 (0.55-1.52)	1.01 (0.59-1.72)	0.61
Epilepsy						
Events	104	26	96	86	54	
Person-years	247657	73859	254924	225458	203844	

HR (95% CI), Model 1	Reference	0.81 (0.52-1.24)	0.85 (0.64-1.13)	0.85 (0.63-1.14)	0.59 (0.42-0.82)	0.0487
HR (95% CI), Model 2	Reference	0.82 (0.53-1.26)	0.87 (0.66-1.16)	0.88 (0.66-1.18)	0.62 (0.44-0.87)	0.10
Migraine						
Events	155	43	153	159	134	
Person-years	241706	72074	248146	218349	197411	
HR (95% CI), Model 1	Reference	0.88 (0.63-1.23)	0.87 (0.69-1.09)	0.97 (0.77-1.21)	0.85 (0.67-1.08)	0.59
HR (95% CI), Model 2	Reference	0.89 (0.63-1.24)	0.88 (0.70-1.10)	0.98 (0.78-1.23)	0.86 (0.67-1.10)	0.65
Dementia						
Events	164	51	183	155	120	
Person-years	249393	74375	256771	226734	205154	
HR (95% CI), Model 1	Reference	0.97 (0.71-1.33)	0.99 (0.80-1.22)	0.94 (0.75-1.18)	0.83 (0.65-1.06)	0.59
HR (95% CI), Model 2	Reference	0.99 (0.72-1.35)	1.01 (0.82-1.25)	0.97 (0.77-1.21)	0.85 (0.66-1.09)	0.67
Parkinson’s disease						
Events	91	25	103	94	76	
Person-years	249150	74407	256535	226667	205095	
HR (95% CI), Model 1	Reference	0.86 (0.55-1.34)	1.02 (0.77-1.35)	1.06 (0.79-1.42)	0.96 (0.70-1.32)	0.92
HR (95% CI), Model 2	Reference	0.86 (0.55-1.34)	1.01 (0.76-1.35)	1.04 (0.78-1.40)	0.95 (0.69-1.31)	0.92
Multiple sclerosis						
Events	19	9	27	20	19	
Person-years	248804	74234	256308	226181	204545	
HR (95% CI), Model 1	Reference	1.54 (0.70-3.41)	1.30 (0.72-2.36)	1.05 (0.55-1.99)	1.05 (0.54-2.04)	0.76
HR (95% CI), Model 2	Reference	1.59 (0.72-3.53)	1.36 (0.75-2.47)	1.12 (0.59-2.14)	1.15 (0.59-2.25)	0.76
Bronchiectasis						
Events	167	41	169	136	135	
Person-years	248618	74151	255901	226051	204537	
HR (95% CI), Model 1	Reference	0.75 (0.53-1.06)	0.86 (0.69-1.07)	0.75 (0.59-0.94)	0.81 (0.64-1.02)	0.12
HR (95% CI), Model 2	Reference	0.77 (0.55-1.09)	0.88 (0.71-1.09)	0.77 (0.61-0.97)	0.83 (0.65-1.05)	0.21
Asthma						
Events	422	117	431	377	309	
Person-years	213844	63921	221707	196689	178634	
HR (95% CI), Model 1	Reference	0.90 (0.74-1.11)	0.94 (0.82-1.08)	0.91 (0.79-1.05)	0.82 (0.70-0.95)	0.13
HR (95% CI), Model 2	Reference	0.94 (0.77-1.16)	1.01 (0.88-1.15)	0.99 (0.86-1.14)	0.91 (0.78-1.06)	0.68
COPD						
Events	515	144	463	366	266	
Person-years	244685	73136	252621	223649	202622	
HR (95% CI), Model 1	Reference	0.88 (0.73-1.06)	0.81 (0.71-0.92)	0.72 (0.63-0.82)	0.58 (0.50-0.68)	<0.0001
HR (95% CI), Model 2	Reference	0.95 (0.79-1.14)	0.89 (0.79-1.01)	0.81 (0.70-0.93)	0.68 (0.58-0.79)	<0.0001
CKD						
Events	1204	346	1071	839	598	
Person-years	244213	73025	252477	223545	202566	
HR (95% CI), Model 1	Reference	0.91 (0.81-1.02)	0.80 (0.74-0.87)	0.70 (0.64-0.77)	0.56 (0.50-0.62)	<0.0001
HR (95% CI), Model 2	Reference	0.96 (0.85-1.08)	0.87 (0.80-0.95)	0.79 (0.72-0.86)	0.65 (0.59-0.72)	<0.0001
Chronic liver disease						
Events	82	17	59	52	26	
Person-years	247994	74119	255725	226095	204402	

HR (95% CI), Model 1	Reference	0.68 (0.40-1.15)	0.68 (0.49-0.96)	0.68 (0.48-0.98)	0.38 (0.24-0.60)	0.0010
HR (95% CI), Model 2	Reference	0.75 (0.44-1.26)	0.79 (0.56-1.11)	0.82 (0.57-1.18)	0.50 (0.31-0.79)	0.0562
Irritable bowel syndrome						
Events	284	89	295	249	183	
Person-years	242015	72311	249361	220311	199906	
HR (95% CI), Model 1	Reference	0.98 (0.77-1.25)	0.90 (0.76-1.06)	0.81 (0.68-0.96)	0.62 (0.52-0.76)	0.0000
HR (95% CI), Model 2	Reference	1.02 (0.80-1.29)	0.95 (0.81-1.12)	0.87 (0.73-1.04)	0.69 (0.56-0.83)	0.0015
Inflammatory bowel disease						
Events	130	35	105	95	83	
Person-years	246068	73622	254100	225215	203650	
HR (95% CI), Model 1	Reference	0.90 (0.62-1.31)	0.79 (0.61-1.02)	0.81 (0.62-1.06)	0.79 (0.59-1.05)	0.33
HR (95% CI), Model 2	Reference	0.92 (0.64-1.34)	0.81 (0.63-1.05)	0.84 (0.64-1.10)	0.82 (0.62-1.10)	0.53
Treated constipation						
Events	849	239	859	760	627	
Person-years	243961	72743	251231	221973	201053	
HR (95% CI), Model 1	Reference	0.90 (0.78-1.04)	0.92 (0.84-1.01)	0.91 (0.82-1.00)	0.83 (0.74-0.92)	0.0148
HR (95% CI), Model 2	Reference	0.92 (0.79-1.06)	0.94 (0.86-1.04)	0.93 (0.84-1.03)	0.86 (0.77-0.95)	0.0891
Dyspepsia						
Events	2636	714	2480	2132	1853	
Person-years	209121	62969	219655	194950	178836	
HR (95% CI), Model 1	Reference	0.86 (0.80-0.94)	0.84 (0.80-0.89)	0.80 (0.75-0.85)	0.75 (0.70-0.80)	<0.0001
HR (95% CI), Model 2	Reference	0.89 (0.82-0.96)	0.88 (0.83-0.93)	0.84 (0.79-0.89)	0.80 (0.75-0.85)	<0.0001
Diverticular disease						
Events	2199	605	2021	1786	1391	
Person-years	231224	69432	240249	212475	194516	
HR (95% CI), Model 1	Reference	0.88 (0.81-0.97)	0.84 (0.79-0.90)	0.84 (0.78-0.89)	0.71 (0.66-0.76)	<0.0001
HR (95% CI), Model 2	Reference	0.91 (0.83-1.00)	0.88 (0.83-0.94)	0.89 (0.83-0.95)	0.77 (0.72-0.83)	<0.0001
Pernicious anaemia						
Events	47	9	35	29	16	
Person-years	248922	74302	256538	226537	204908	
HR (95% CI), Model 1	Reference	0.60 (0.30-1.24)	0.66 (0.42-1.02)	0.59 (0.37-0.95)	0.35 (0.20-0.63)	0.0086
HR (95% CI), Model 2	Reference	0.64 (0.31-1.30)	0.70 (0.45-1.10)	0.65 (0.40-1.04)	0.39 (0.22-0.71)	0.0355
Fracture						
Events	171	51	194	162	181	
Person-years	248026	73917	255461	225381	203741	
HR (95% CI), Model 1	Reference	0.92 (0.67-1.25)	0.97 (0.78-1.19)	0.87 (0.70-1.09)	1.06 (0.85-1.31)	0.49
HR (95% CI), Model 2	Reference	0.90 (0.66-1.23)	0.94 (0.76-1.15)	0.84 (0.67-1.05)	0.99 (0.80-1.24)	0.50
Osteoporosis						
Events	1509	462	1583	1328	1233	
Person-years	222926	66528	228501	202324	183125	
HR (95% CI), Model 1	Reference	0.96 (0.86-1.06)	0.93 (0.86-1.00)	0.85 (0.79-0.92)	0.86 (0.80-0.93)	0.0002
HR (95% CI), Model 2	Reference	1.01 (0.91-1.12)	1.00 (0.93-1.08)	0.94 (0.87-1.02)	0.99 (0.92-1.07)	0.45
Meniere’s disease						
Events	36	2	24	21	18	

Person-years	249030	74360	256399	226381	204737	
HR (95% CI), Model 1	Reference	0.18 (0.04-0.74)	0.60 (0.36-1.01)	0.57 (0.33-1.00)	0.53 (0.29-0.95)	0.0318
HR (95% CI), Model 2	Reference	0.18 (0.04-0.75)	0.62 (0.37-1.05)	0.60 (0.34-1.05)	0.56 (0.31-1.02)	0.0538
Eczema						
Events	291	106	272	249	214	
Person-years	237980	71170	246212	217008	196961	
HR (95% CI), Model 1	Reference	1.18 (0.95-1.48)	0.87 (0.73-1.03)	0.90 (0.75-1.07)	0.85 (0.71-1.02)	0.0308
HR (95% CI), Model 2	Reference	1.21 (0.97-1.52)	0.90 (0.76-1.06)	0.93 (0.78-1.11)	0.88 (0.73-1.06)	0.0613
Glaucoma						
Events	363	109	386	348	337	
Person-years	245749	73456	252973	223176	201796	
HR (95% CI), Model 1	Reference	0.95 (0.77-1.18)	0.97 (0.84-1.12)	0.99 (0.85-1.15)	1.08 (0.93-1.26)	0.63
HR (95% CI), Model 2	Reference	0.95 (0.76-1.17)	0.96 (0.83-1.11)	0.97 (0.84-1.13)	1.06 (0.90-1.23)	0.72
Cataract						
Events	1784	530	1911	1682	1579	
Person-years	236098	70558	243030	214167	193045	
HR (95% CI), Model 1	Reference	0.90 (0.82-0.99)	0.91 (0.85-0.97)	0.87 (0.81-0.93)	0.90 (0.84-0.97)	0.0016
HR (95% CI), Model 2	Reference	0.92 (0.83-1.01)	0.92 (0.87-0.99)	0.90 (0.84-0.96)	0.94 (0.87-1.01)	0.0256
AMD						
Events	302	78	319	307	272	
Person-years	248279	74131	255527	225519	203959	
HR (95% CI), Model 1	Reference	0.79 (0.61-1.01)	0.90 (0.77-1.05)	0.95 (0.80-1.11)	0.92 (0.78-1.09)	0.38
HR (95% CI), Model 2	Reference	0.80 (0.62-1.02)	0.91 (0.78-1.07)	0.97 (0.82-1.14)	0.95 (0.80-1.13)	0.45
Thyroid disorders						
Events	488	143	528	494	454	
Person-years	235155	69953	240316	210798	189731	
HR (95% CI), Model 1	Reference	0.90 (0.75-1.09)	0.91 (0.81-1.03)	0.90 (0.80-1.03)	0.87 (0.76-0.99)	0.30
HR (95% CI), Model 2	Reference	0.93 (0.77-1.13)	0.97 (0.85-1.09)	0.98 (0.86-1.11)	0.97 (0.85-1.11)	0.96
Prostate disorders¶						
Events	1077	289	851	657	436	
Person-years	136006	35752	109616	80652	59907	
HR (95% CI), Model 1	Reference	0.97 (0.85-1.10)	0.90 (0.82-0.99)	0.94 (0.85-1.04)	0.83 (0.74-0.93)	0.0204
HR (95% CI), Model 2	Reference	0.97 (0.85-1.11)	0.90 (0.83-0.99)	0.94 (0.85-1.04)	0.84 (0.75-0.94)	0.0317

AMD, age related macular degeneration; CI, confidence interval; CKD, chronic kidney disease; COPD, chronic obstructive pulmonary disease; HR, hazard ratio.

*Cox proportional hazard regression models were used to examine associations of the Healthful Plant-based Diet Index (quintiles) with the incidence of individual chronic diseases. The statistical tests were two-sided. Model 1 was adjusted for age and sex; Model 2 was adjusted for Model 1 plus ethnicity, education, income, smoking, alcohol consumption, sleep, physical activity, and GRS for longevity; Model 3 was adjusted for Model 2 and total energy intake.

†Cardiovascular disease includes coronary heart disease, heart failure, atrial fibrillation, other cardiac disease, stroke, and peripheral vascular disease.

‡All cancers encompass any type of cancer except for non-melanoma skin cancer.

[§]These analyses were conducted among women only.

[¶]These analyses were conducted among men only.

Table S5. The association between the Alternate Mediterranean Diet Index and the incidence of individual chronic diseases among individuals by excluding those developed the corresponding disease in the first 4 years of follow-up

	Alternate Mediterranean Diet Index					P-value
	Quintile 1	Quintile 2	Quintile 3	Quintile 4	Quintile 5	for trend*
Cardiovascular disease†						
Events	1038	967	1064	886	1137	
Person-years	172159	168209	196005	177898	224800	
HR (95% CI), Model 1	Reference	0.93 (0.85-1.01)	0.86 (0.79-0.94)	0.80 (0.73-0.87)	0.80 (0.73-0.87)	<0.0001
HR (95% CI), Model 2	Reference	0.95 (0.87-1.04)	0.90 (0.82-0.98)	0.85 (0.78-0.93)	0.89 (0.81-0.97)	0.0040
Coronary heart disease						
Events	608	566	617	541	649	
Person-years	180630	175408	204041	184491	233206	
HR (95% CI), Model 1	Reference	0.95 (0.85-1.07)	0.88 (0.79-0.99)	0.87 (0.78-0.98)	0.83 (0.75-0.93)	0.0146
HR (95% CI), Model 2	Reference	0.98 (0.88-1.10)	0.93 (0.83-1.04)	0.95 (0.85-1.07)	0.94 (0.84-1.06)	0.71
Heart failure						
Events	226	222	225	196	196	
Person-years	187405	181128	209765	189474	238850	
HR (95% CI), Model 1	Reference	0.98 (0.81-1.18)	0.83 (0.69-1.00)	0.80 (0.66-0.98)	0.63 (0.52-0.76)	<0.0001
HR (95% CI), Model 2	Reference	1.03 (0.86-1.24)	0.90 (0.75-1.09)	0.91 (0.75-1.10)	0.76 (0.62-0.92)	0.0246
Atrial fibrillation						
Events	82	89	95	71	95	
Person-years	184546	178235	206767	186684	235412	
HR (95% CI), Model 1	Reference	1.09 (0.80-1.47)	0.98 (0.73-1.32)	0.82 (0.60-1.13)	0.85 (0.63-1.15)	0.35
HR (95% CI), Model 2	Reference	1.12 (0.83-1.51)	1.02 (0.76-1.38)	0.88 (0.64-1.21)	0.96 (0.70-1.30)	0.64
Other cardiac disease						
Events	564	508	550	437	568	
Person-years	184780	178562	207280	187608	236238	
HR (95% CI), Model 1	Reference	0.91 (0.80-1.02)	0.83 (0.73-0.93)	0.73 (0.64-0.82)	0.74 (0.66-0.83)	<0.0001
HR (95% CI), Model 2	Reference	0.94 (0.83-1.06)	0.88 (0.78-0.99)	0.79 (0.70-0.90)	0.84 (0.74-0.95)	0.0027
Stroke						
Events	35	34	40	38	40	
Person-years	187431	181125	209690	189455	238788	
HR (95% CI), Model 1	Reference	0.97 (0.61-1.56)	0.96 (0.61-1.52)	1.01 (0.64-1.60)	0.83 (0.52-1.31)	0.91
HR (95% CI), Model 2	Reference	1.00 (0.62-1.60)	1.00 (0.64-1.58)	1.07 (0.67-1.71)	0.90 (0.56-1.43)	0.96
Peripheral vascular disease						
Events	127	126	119	109	124	
Person-years	187819	181513	210159	189977	238878	
HR (95% CI), Model 1	Reference	1.00 (0.78-1.28)	0.80 (0.62-1.03)	0.81 (0.63-1.05)	0.72 (0.56-0.93)	0.0358
HR (95% CI), Model 2	Reference	1.03 (0.81-1.32)	0.84 (0.65-1.08)	0.87 (0.67-1.12)	0.79 (0.61-1.02)	0.19
Hypertension						
Events	213	172	222	174	201	
Person-years	134014	131276	154122	142527	182756	
HR (95% CI), Model 1	Reference	0.81 (0.66-0.99)	0.87 (0.72-1.05)	0.74 (0.60-0.90)	0.65 (0.54-0.80)	0.0004

HR (95% CI), Model 2	Reference	0.84 (0.69-1.03)	0.93 (0.77-1.13)	0.83 (0.68-1.01)	0.78 (0.64-0.95)	0.1040
Diabetes						
Events	352	353	329	257	293	
Person-years	178581	172977	201799	182874	231872	
HR (95% CI), Model 1	Reference	1.03 (0.89-1.19)	0.82 (0.70-0.95)	0.71 (0.60-0.84)	0.64 (0.55-0.75)	<0.0001
HR (95% CI), Model 2	Reference	1.16 (1.00-1.34)	0.95 (0.82-1.11)	0.91 (0.77-1.07)	0.92 (0.78-1.08)	0.0141
All cancers‡						
Events	769	682	749	640	801	
Person-years	169590	164393	191675	172960	218258	
HR (95% CI), Model 1	Reference	0.88 (0.80-0.98)	0.81 (0.73-0.89)	0.76 (0.68-0.84)	0.73 (0.66-0.81)	<0.0001
HR (95% CI), Model 2	Reference	0.90 (0.82-1.00)	0.84 (0.76-0.93)	0.80 (0.72-0.89)	0.80 (0.72-0.89)	<0.0001
Non-melanoma skin cancer						
Events	411	400	465	407	575	
Person-years	183558	177064	204947	184753	232468	
HR (95% CI), Model 1	Reference	0.98 (0.85-1.12)	0.96 (0.84-1.10)	0.94 (0.82-1.08)	1.04 (0.91-1.18)	0.60
HR (95% CI), Model 2	Reference	0.97 (0.84-1.11)	0.95 (0.83-1.09)	0.92 (0.80-1.06)	1.01 (0.89-1.16)	0.62
Melanoma						
Events	59	47	57	54	78	
Person-years	187523	180428	208958	188333	237208	
HR (95% CI), Model 1	Reference	0.81 (0.55-1.19)	0.83 (0.58-1.19)	0.87 (0.60-1.25)	0.97 (0.69-1.37)	0.71
HR (95% CI), Model 2	Reference	0.81 (0.55-1.19)	0.83 (0.58-1.20)	0.87 (0.60-1.27)	1.00 (0.70-1.41)	0.65
Lung cancer						
Events	103	70	62	61	71	
Person-years	188302	181819	210835	190345	239554	
HR (95% CI), Model 1	Reference	0.66 (0.49-0.90)	0.49 (0.35-0.67)	0.51 (0.37-0.71)	0.45 (0.33-0.62)	<0.0001
HR (95% CI), Model 2	Reference	0.70 (0.52-0.94)	0.60 (0.45-0.81)	0.63 (0.46-0.85)	0.59 (0.44-0.80)	0.0012
Stomach cancer						
Events	18	15	17	14	14	
Person-years	189076	182360	211225	190679	239955	
HR (95% CI), Model 1	Reference	0.86 (0.43-1.71)	0.85 (0.43-1.64)	0.79 (0.39-1.59)	0.63 (0.31-1.28)	0.80
HR (95% CI), Model 2	Reference	0.90 (0.45-1.78)	0.90 (0.46-1.75)	0.87 (0.43-1.76)	0.74 (0.36-1.51)	0.95
Oesophageal cancer						
Events	32	23	17	12	20	
Person-years	188945	182282	211241	190612	240007	
HR (95% CI), Model 1	Reference	0.73 (0.43-1.25)	0.46 (0.26-0.84)	0.37 (0.19-0.72)	0.49 (0.28-0.86)	0.0095
HR (95% CI), Model 2	Reference	0.81 (0.47-1.39)	0.54 (0.30-0.98)	0.45 (0.23-0.88)	0.66 (0.37-1.18)	0.11
Colon cancer						
Events	97	90	87	94	104	
Person-years	187498	180979	209795	189289	238507	
HR (95% CI), Model 1	Reference	0.93 (0.70-1.24)	0.76 (0.57-1.01)	0.90 (0.68-1.20)	0.77 (0.58-1.02)	0.25
HR (95% CI), Model 2	Reference	0.96 (0.72-1.28)	0.79 (0.59-1.06)	0.96 (0.72-1.28)	0.85 (0.64-1.13)	0.48
Ovarian cancer§						
Events	18	20	31	26	34	
Person-years	95360	99785	122763	118502	158965	

HR (95% CI), Model 1	Reference	1.02 (0.54-1.93)	1.25 (0.70-2.24)	1.07 (0.59-1.96)	1.01 (0.57-1.81)	0.91
HR (95% CI), Model 2	Reference	1.04 (0.55-1.96)	1.28 (0.71-2.29)	1.10 (0.60-2.01)	1.06 (0.59-1.90)	0.91
Breast cancer[§]						
Events	188	177	210	196	273	
Person-years	89471	93621	115553	111400	148644	
HR (95% CI), Model 1	Reference	0.89 (0.72-1.09)	0.84 (0.69-1.03)	0.81 (0.66-0.99)	0.83 (0.69-1.00)	0.25
HR (95% CI), Model 2	Reference	0.90 (0.73-1.11)	0.87 (0.71-1.06)	0.85 (0.69-1.04)	0.89 (0.73-1.07)	0.56
Prostate cancer[¶]						
Events	252	261	263	243	289	
Person-years	101940	88654	95344	77966	87247	
HR (95% CI), Model 1	Reference	1.13 (0.95-1.35)	1.03 (0.86-1.22)	1.14 (0.96-1.36)	1.17 (0.99-1.39)	0.27
HR (95% CI), Model 2	Reference	1.10 (0.92-1.31)	0.99 (0.83-1.17)	1.09 (0.91-1.30)	1.10 (0.92-1.30)	0.57
Other cancers						
Events	683	593	654	556	692	
Person-years	172713	167932	195156	176335	222347	
HR (95% CI), Model 1	Reference	0.86 (0.77-0.96)	0.80 (0.72-0.89)	0.75 (0.67-0.84)	0.72 (0.65-0.80)	<0.0001
HR (95% CI), Model 2	Reference	0.88 (0.79-0.98)	0.83 (0.74-0.92)	0.79 (0.70-0.88)	0.78 (0.70-0.87)	<0.0001
Depression						
Events	76	45	50	46	65	
Person-years	175905	171266	199067	180023	227831	
HR (95% CI), Model 1	Reference	0.59 (0.41-0.85)	0.56 (0.39-0.79)	0.55 (0.38-0.80)	0.60 (0.43-0.84)	0.0020
HR (95% CI), Model 2	Reference	0.62 (0.43-0.90)	0.60 (0.42-0.86)	0.62 (0.43-0.90)	0.70 (0.50-0.99)	0.0224
Anxiety						
Events	470	370	419	340	461	
Person-years	182393	176651	204605	185294	233418	
HR (95% CI), Model 1	Reference	0.78 (0.68-0.89)	0.73 (0.64-0.84)	0.64 (0.55-0.73)	0.66 (0.58-0.75)	<0.0001
HR (95% CI), Model 2	Reference	0.80 (0.70-0.92)	0.77 (0.67-0.88)	0.68 (0.59-0.79)	0.72 (0.63-0.83)	<0.0001
Schizophrenia						
Events	14	12	13	12	16	
Person-years	188635	181918	210652	190109	239529	
HR (95% CI), Model 1	Reference	0.86 (0.40-1.86)	0.79 (0.37-1.68)	0.79 (0.37-1.73)	0.82 (0.39-1.69)	0.97
HR (95% CI), Model 2	Reference	0.99 (0.46-2.14)	0.97 (0.45-2.08)	1.03 (0.47-2.26)	1.18 (0.56-2.50)	0.98
Alcohol use disorder						
Events	140	110	104	87	60	
Person-years	187186	181061	210047	189813	239511	
HR (95% CI), Model 1	Reference	0.82 (0.64-1.06)	0.68 (0.53-0.88)	0.65 (0.49-0.85)	0.36 (0.26-0.49)	<0.0001
HR (95% CI), Model 2	Reference	0.87 (0.67-1.11)	0.74 (0.57-0.96)	0.73 (0.55-0.96)	0.42 (0.31-0.58)	<0.0001
Psychoactive substance abuse						
Events	28	20	20	12	15	
Person-years	188746	182087	211092	190593	239995	
HR (95% CI), Model 1	Reference	0.77 (0.43-1.36)	0.68 (0.38-1.21)	0.47 (0.24-0.93)	0.48 (0.26-0.91)	0.11
HR (95% CI), Model 2	Reference	0.86 (0.48-1.53)	0.80 (0.45-1.42)	0.58 (0.29-1.16)	0.65 (0.34-1.24)	0.55
Epilepsy						
Events	51	37	55	45	46	

Person-years	187177	181015	209362	189386	238503	
HR (95% CI), Model 1	Reference	0.73 (0.48-1.12)	0.93 (0.63-1.36)	0.83 (0.56-1.25)	0.66 (0.44-1.00)	0.27
HR (95% CI), Model 2	Reference	0.75 (0.49-1.14)	0.95 (0.64-1.39)	0.86 (0.57-1.30)	0.70 (0.47-1.06)	0.39
Migraine						
Events	85	78	81	72	117	
Person-years	182484	176208	203706	183899	230946	
HR (95% CI), Model 1	Reference	0.92 (0.68-1.25)	0.81 (0.60-1.10)	0.78 (0.57-1.07)	0.99 (0.75-1.32)	0.36
HR (95% CI), Model 2	Reference	0.92 (0.68-1.25)	0.81 (0.60-1.10)	0.78 (0.57-1.08)	0.99 (0.74-1.33)	0.36
Dementia						
Events	120	114	114	95	138	
Person-years	188811	182153	211014	190475	239745	
HR (95% CI), Model 1	Reference	0.92 (0.72-1.20)	0.76 (0.59-0.99)	0.69 (0.53-0.91)	0.77 (0.60-0.99)	0.0421
HR (95% CI), Model 2	Reference	0.94 (0.73-1.21)	0.78 (0.60-1.01)	0.71 (0.54-0.94)	0.80 (0.62-1.03)	0.0894
Parkinson’s disease						
Events	56	60	60	61	54	
Person-years	188772	182162	210718	190296	239670	
HR (95% CI), Model 1	Reference	1.08 (0.75-1.56)	0.92 (0.64-1.32)	1.04 (0.72-1.50)	0.73 (0.50-1.06)	0.24
HR (95% CI), Model 2	Reference	1.07 (0.74-1.54)	0.90 (0.62-1.29)	1.01 (0.70-1.47)	0.69 (0.47-1.02)	0.18
Multiple sclerosis						
Events	11	10	14	8	8	
Person-years	188443	181911	210444	189995	239190	
HR (95% CI), Model 1	Reference	0.91 (0.39-2.15)	1.08 (0.49-2.39)	0.67 (0.27-1.67)	0.52 (0.21-1.31)	0.48
HR (95% CI), Model 2	Reference	0.98 (0.42-2.32)	1.21 (0.54-2.68)	0.77 (0.31-1.95)	0.63 (0.25-1.62)	0.66
Bronchiectasis						
Events	104	100	90	72	95	
Person-years	188236	181582	210256	189821	238927	
HR (95% CI), Model 1	Reference	0.93 (0.71-1.22)	0.68 (0.51-0.90)	0.58 (0.43-0.78)	0.57 (0.43-0.76)	<0.0001
HR (95% CI), Model 2	Reference	0.95 (0.72-1.25)	0.70 (0.53-0.93)	0.59 (0.44-0.81)	0.58 (0.44-0.77)	0.0001
Asthma						
Events	208	168	203	171	225	
Person-years	159905	155822	181341	164090	209685	
HR (95% CI), Model 1	Reference	0.80 (0.65-0.98)	0.82 (0.67-0.99)	0.75 (0.61-0.91)	0.75 (0.62-0.91)	0.0235
HR (95% CI), Model 2	Reference	0.83 (0.68-1.02)	0.87 (0.71-1.05)	0.81 (0.66-1.00)	0.86 (0.70-1.04)	0.29
COPD						
Events	318	254	241	198	213	
Person-years	184308	178898	207612	187836	236885	
HR (95% CI), Model 1	Reference	0.79 (0.67-0.93)	0.62 (0.53-0.74)	0.56 (0.47-0.67)	0.46 (0.39-0.55)	<0.0001
HR (95% CI), Model 2	Reference	0.87 (0.74-1.03)	0.73 (0.62-0.87)	0.69 (0.57-0.82)	0.63 (0.53-0.75)	<0.0001
CKD						
Events	697	575	614	504	556	
Person-years	183773	178398	207206	187489	236354	
HR (95% CI), Model 1	Reference	0.81 (0.73-0.91)	0.72 (0.65-0.81)	0.65 (0.58-0.73)	0.55 (0.49-0.62)	<0.0001
HR (95% CI), Model 2	Reference	0.85 (0.76-0.95)	0.78 (0.70-0.88)	0.73 (0.65-0.82)	0.67 (0.60-0.75)	<0.0001
Chronic liver disease						
Events	47	37	30	26	23	

Person-years	187806	181515	210323	189492	239034	
HR (95% CI), Model 1	Reference	0.83 (0.54-1.28)	0.59 (0.37-0.93)	0.58 (0.36-0.94)	0.42 (0.25-0.69)	0.0057
HR (95% CI), Model 2	Reference	0.92 (0.60-1.42)	0.69 (0.43-1.09)	0.73 (0.45-1.19)	0.59 (0.35-0.99)	0.23
Irritable bowel syndrome						
Events	189	117	137	151	163	
Person-years	183072	176781	205149	185013	233124	
HR (95% CI), Model 1	Reference	0.61 (0.48-0.76)	0.59 (0.47-0.74)	0.69 (0.56-0.86)	0.57 (0.46-0.70)	<0.0001
HR (95% CI), Model 2	Reference	0.63 (0.50-0.79)	0.63 (0.50-0.78)	0.76 (0.61-0.94)	0.64 (0.52-0.80)	<0.0001
Inflammatory bowel disease						
Events	61	50	62	49	62	
Person-years	186297	180382	209089	188701	237821	
HR (95% CI), Model 1	Reference	0.85 (0.58-1.23)	0.91 (0.64-1.29)	0.80 (0.55-1.16)	0.80 (0.56-1.15)	0.72
HR (95% CI), Model 2	Reference	0.87 (0.60-1.26)	0.93 (0.65-1.34)	0.83 (0.57-1.22)	0.85 (0.59-1.22)	0.86
Treated constipation						
Events	471	388	453	405	521	
Person-years	183361	177739	206003	186872	234591	
HR (95% CI), Model 1	Reference	0.82 (0.71-0.93)	0.80 (0.70-0.91)	0.78 (0.68-0.89)	0.78 (0.68-0.88)	0.0004
HR (95% CI), Model 2	Reference	0.83 (0.72-0.95)	0.82 (0.72-0.93)	0.80 (0.70-0.92)	0.81 (0.71-0.93)	0.0049
Dyspepsia						
Events	1078	1079	1161	1051	1253	
Person-years	154060	153103	178578	162536	208234	
HR (95% CI), Model 1	Reference	0.98 (0.90-1.06)	0.88 (0.81-0.96)	0.86 (0.79-0.94)	0.78 (0.72-0.85)	<0.0001
HR (95% CI), Model 2	Reference	1.00 (0.92-1.09)	0.92 (0.84-1.00)	0.92 (0.84-1.00)	0.86 (0.79-0.94)	0.0016
Diverticular disease						
Events	1005	911	1075	851	1056	
Person-years	173504	169017	195341	178010	225295	
HR (95% CI), Model 1	Reference	0.90 (0.83-0.99)	0.91 (0.83-0.99)	0.78 (0.71-0.85)	0.75 (0.69-0.82)	<0.0001
HR (95% CI), Model 2	Reference	0.93 (0.85-1.02)	0.95 (0.87-1.04)	0.84 (0.76-0.92)	0.84 (0.77-0.92)	<0.0001
Pernicious anaemia						
Events	25	16	19	12	13	
Person-years	188561	181882	210821	190279	239559	
HR (95% CI), Model 1	Reference	0.63 (0.33-1.17)	0.61 (0.34-1.12)	0.41 (0.21-0.83)	0.34 (0.17-0.66)	0.0161
HR (95% CI), Model 2	Reference	0.66 (0.35-1.24)	0.67 (0.37-1.23)	0.47 (0.23-0.94)	0.40 (0.20-0.80)	0.0791
Fracture						
Events	98	85	101	99	134	
Person-years	187688	180941	209831	189282	238241	
HR (95% CI), Model 1	Reference	0.85 (0.63-1.13)	0.82 (0.62-1.09)	0.87 (0.65-1.15)	0.89 (0.68-1.16)	0.71
HR (95% CI), Model 2	Reference	0.85 (0.63-1.13)	0.82 (0.62-1.09)	0.86 (0.65-1.14)	0.85 (0.65-1.12)	0.68
Osteoporosis						
Events	730	657	780	717	848	
Person-years	166188	162037	187111	169016	214038	
HR (95% CI), Model 1	Reference	0.88 (0.79-0.97)	0.86 (0.78-0.96)	0.86 (0.77-0.95)	0.77 (0.69-0.85)	<0.0001
HR (95% CI), Model 2	Reference	0.91 (0.82-1.01)	0.93 (0.84-1.02)	0.95 (0.85-1.05)	0.91 (0.82-1.00)	0.31
Meniere's disease						

Events	10	7	9	15	16	
Person-years	188717	182046	210508	190216	239331	
HR (95% CI), Model 1	Reference	0.70 (0.27-1.85)	0.77 (0.31-1.89)	1.39 (0.62-3.12)	1.16 (0.52-2.58)	0.50
HR (95% CI), Model 2	Reference	0.74 (0.28-1.94)	0.83 (0.33-2.05)	1.57 (0.70-3.54)	1.40 (0.62-3.18)	0.35
Eczema						
Events	188	156	156	149	173	
Person-years	180128	174071	202110	182381	229964	
HR (95% CI), Model 1	Reference	0.84 (0.68-1.04)	0.71 (0.58-0.88)	0.75 (0.61-0.94)	0.69 (0.56-0.85)	0.0033
HR (95% CI), Model 2	Reference	0.85 (0.69-1.06)	0.73 (0.59-0.91)	0.78 (0.63-0.97)	0.73 (0.59-0.90)	0.0216
Glaucoma						
Events	193	211	245	211	277	
Person-years	186132	179134	207729	187498	235725	
HR (95% CI), Model 1	Reference	1.09 (0.90-1.33)	1.05 (0.87-1.27)	0.99 (0.82-1.21)	1.01 (0.84-1.21)	0.84
HR (95% CI), Model 2	Reference	1.08 (0.89-1.31)	1.04 (0.86-1.26)	0.97 (0.80-1.19)	0.97 (0.80-1.18)	0.77
Cataract						
Events	943	913	1032	1041	1247	
Person-years	177737	171795	198609	178928	224622	
HR (95% CI), Model 1	Reference	0.93 (0.85-1.02)	0.86 (0.79-0.94)	0.93 (0.85-1.02)	0.84 (0.77-0.92)	0.0006
HR (95% CI), Model 2	Reference	0.94 (0.86-1.03)	0.88 (0.80-0.96)	0.96 (0.88-1.05)	0.88 (0.80-0.96)	0.0097
AMD						
Events	153	172	184	188	226	
Person-years	187980	181016	209921	189319	238320	
HR (95% CI), Model 1	Reference	1.09 (0.88-1.36)	0.95 (0.77-1.18)	1.04 (0.84-1.29)	0.94 (0.76-1.15)	0.56
HR (95% CI), Model 2	Reference	1.12 (0.90-1.39)	0.98 (0.79-1.22)	1.08 (0.87-1.34)	0.99 (0.80-1.22)	0.64
Thyroid disorders						
Events	285	244	290	264	311	
Person-years	176173	170574	196824	177440	223365	
HR (95% CI), Model 1	Reference	0.84 (0.71-1.00)	0.84 (0.71-0.99)	0.82 (0.69-0.97)	0.74 (0.62-0.87)	0.0078
HR (95% CI), Model 2	Reference	0.86 (0.73-1.02)	0.87 (0.74-1.03)	0.88 (0.74-1.04)	0.83 (0.70-0.98)	0.24
Prostate disorders¶						
Events	464	406	455	384	436	
Person-years	94401	82702	88940	72529	80884	
HR (95% CI), Model 1	Reference	0.94 (0.82-1.07)	0.95 (0.83-1.08)	0.96 (0.84-1.10)	0.95 (0.83-1.08)	0.89
HR (95% CI), Model 2	Reference	0.93 (0.82-1.07)	0.94 (0.83-1.07)	0.96 (0.83-1.10)	0.94 (0.82-1.07)	0.85

AMD, age related macular degeneration; CI, confidence interval; CKD, chronic kidney disease; COPD, chronic obstructive pulmonary disease; HR, hazard ratio.

*Cox proportional hazard regression models were used to examine associations of the Alternate Mediterranean Diet Index (quintiles) with the incidence of individual chronic diseases. The statistical tests were two-sided. Model 1 was adjusted for age and sex; Model 2 was adjusted for Model 1 plus ethnicity, education, income, smoking, alcohol consumption, sleep, physical activity, and GRS for longevity; Model 3 was adjusted for Model 2 and total energy intake.

†Cardiovascular disease includes coronary heart disease, heart failure, atrial fibrillation, other cardiac disease, stroke, and peripheral vascular disease.

[‡]All cancers encompass any type of cancer except for non-melanoma skin cancer.

[§]These analyses were conducted among women only.

[¶]These analyses were conducted among men only.

Table S6. The association between the Anti-Empirical Dietary Inflammatory Index and the incidence of individual chronic diseases among individuals by excluding those developed the corresponding disease in the first 4 years of follow-up

	Anti-Empirical Dietary Inflammatory Index					P-value
	Quintile 1	Quintile 2	Quintile 3	Quintile 4	Quintile 5	for trend*
Cardiovascular disease[†]						
Events	1024	1010	1036	979	1043	
Person-years	185095	188136	188194	188672	188974	
HR (95% CI), Model 1	Reference	0.90 (0.82-0.98)	0.88 (0.80-0.96)	0.79 (0.72-0.86)	0.81 (0.75-0.89)	<0.0001
HR (95% CI), Model 2	Reference	0.94 (0.86-1.03)	0.93 (0.85-1.01)	0.85 (0.78-0.93)	0.88 (0.81-0.97)	0.0073
Coronary heart disease						
Events	631	614	587	557	592	
Person-years	193260	195525	195977	196440	196574	
HR (95% CI), Model 1	Reference	0.89 (0.80-1.00)	0.81 (0.72-0.90)	0.73 (0.65-0.82)	0.75 (0.67-0.84)	<0.0001
HR (95% CI), Model 2	Reference	0.94 (0.84-1.06)	0.87 (0.78-0.97)	0.80 (0.72-0.90)	0.83 (0.74-0.93)	0.0010
Heart failure						
Events	224	207	226	202	206	
Person-years	199622	201283	201630	201784	202303	
HR (95% CI), Model 1	Reference	0.83 (0.69-1.00)	0.85 (0.70-1.02)	0.71 (0.59-0.86)	0.70 (0.58-0.84)	0.0012
HR (95% CI), Model 2	Reference	0.92 (0.76-1.11)	0.96 (0.80-1.16)	0.85 (0.70-1.03)	0.85 (0.70-1.03)	0.33
Atrial fibrillation						
Events	78	82	83	100	89	
Person-years	196644	198594	198686	198547	199174	
HR (95% CI), Model 1	Reference	0.96 (0.70-1.30)	0.90 (0.66-1.23)	1.04 (0.77-1.39)	0.88 (0.65-1.19)	0.80
HR (95% CI), Model 2	Reference	1.02 (0.74-1.39)	0.98 (0.71-1.33)	1.14 (0.84-1.54)	0.98 (0.71-1.33)	0.82
Other cardiac disease						
Events	502	522	568	520	515	
Person-years	196876	199049	199061	199223	200259	
HR (95% CI), Model 1	Reference	0.94 (0.83-1.07)	0.98 (0.86-1.10)	0.85 (0.75-0.96)	0.81 (0.72-0.92)	0.0019
HR (95% CI), Model 2	Reference	1.00 (0.89-1.13)	1.05 (0.93-1.18)	0.94 (0.83-1.06)	0.90 (0.79-1.02)	0.12
Stroke						
Events	42	40	25	42	38	
Person-years	199670	201377	201466	201838	202138	
HR (95% CI), Model 1	Reference	0.87 (0.56-1.34)	0.51 (0.31-0.84)	0.82 (0.53-1.26)	0.72 (0.46-1.11)	0.10
HR (95% CI), Model 2	Reference	0.92 (0.59-1.42)	0.55 (0.33-0.91)	0.91 (0.59-1.40)	0.81 (0.51-1.26)	0.18
Peripheral vascular disease						
Events	136	106	110	117	136	
Person-years	199867	201764	201883	202220	202613	
HR (95% CI), Model 1	Reference	0.72 (0.56-0.93)	0.72 (0.56-0.92)	0.73 (0.57-0.94)	0.83 (0.66-1.06)	0.0353
HR (95% CI), Model 2	Reference	0.74 (0.57-0.95)	0.74 (0.57-0.95)	0.76 (0.59-0.98)	0.85 (0.67-1.09)	0.0710
Hypertension						
Events	178	192	213	187	212	
Person-years	142957	148616	149572	151616	151934	
HR (95% CI), Model 1	Reference	0.95 (0.78-1.17)	1.00 (0.82-1.23)	0.83 (0.67-1.02)	0.92 (0.75-1.12)	0.32

HR (95% CI), Model 2	Reference	1.05 (0.85-1.29)	1.13 (0.92-1.38)	0.96 (0.78-1.18)	1.09 (0.88-1.33)	0.50
Diabetes						
Events	448	324	300	278	234	
Person-years	188118	192754	194306	195956	196970	
HR (95% CI), Model 1	Reference	0.68 (0.59-0.78)	0.60 (0.52-0.70)	0.54 (0.46-0.63)	0.44 (0.38-0.52)	<0.0001
HR (95% CI), Model 2	Reference	0.83 (0.72-0.96)	0.77 (0.66-0.89)	0.74 (0.64-0.87)	0.63 (0.54-0.75)	<0.0001
All cancers‡						
Events	669	702	754	760	756	
Person-years	182632	183413	183586	183204	184041	
HR (95% CI), Model 1	Reference	0.98 (0.88-1.09)	1.01 (0.91-1.12)	0.98 (0.88-1.09)	0.95 (0.86-1.05)	0.81
HR (95% CI), Model 2	Reference	1.00 (0.90-1.12)	1.04 (0.94-1.15)	1.02 (0.92-1.13)	0.98 (0.88-1.09)	0.85
Non-melanoma skin cancer						
Events	381	397	459	508	513	
Person-years	195718	196737	197106	196313	196918	
HR (95% CI), Model 1	Reference	0.95 (0.83-1.10)	1.05 (0.91-1.20)	1.11 (0.97-1.27)	1.09 (0.95-1.24)	0.15
HR (95% CI), Model 2	Reference	0.94 (0.81-1.08)	1.02 (0.89-1.17)	1.08 (0.95-1.24)	1.06 (0.93-1.22)	0.24
Melanoma						
Events	44	74	61	65	51	
Person-years	199346	200539	200620	200924	201022	
HR (95% CI), Model 1	Reference	1.58 (1.09-2.30)	1.26 (0.86-1.86)	1.30 (0.89-1.92)	1.01 (0.67-1.51)	0.0656
HR (95% CI), Model 2	Reference	1.57 (1.08-2.29)	1.26 (0.85-1.86)	1.30 (0.88-1.92)	1.01 (0.67-1.53)	0.0781
Lung cancer						
Events	75	55	76	76	85	
Person-years	200592	202252	202407	202596	203009	
HR (95% CI), Model 1	Reference	0.67 (0.47-0.95)	0.88 (0.64-1.21)	0.84 (0.61-1.16)	0.91 (0.67-1.25)	0.23
HR (95% CI), Model 2	Reference	0.70 (0.50-0.97)	0.92 (0.68-1.24)	0.88 (0.65-1.19)	0.85 (0.63-1.15)	0.29
Stomach cancer						
Events	19	17	13	18	11	
Person-years	201104	202655	202910	203055	203569	
HR (95% CI), Model 1	Reference	0.85 (0.44-1.64)	0.62 (0.30-1.25)	0.82 (0.43-1.57)	0.48 (0.23-1.02)	0.34
HR (95% CI), Model 2	Reference	0.92 (0.92-0.92)	0.69 (0.34-1.40)	0.93 (0.93-0.93)	0.56 (0.26-1.19)	0.52
Oesophageal cancer						
Events	17	22	21	21	23	
Person-years	201041	202654	202856	203112	203424	
HR (95% CI), Model 1	Reference	1.21 (0.64-2.28)	1.10 (0.58-2.08)	1.05 (0.55-1.99)	1.11 (0.59-2.08)	0.98
HR (95% CI), Model 2	Reference	1.38 (0.73-2.60)	1.29 (0.68-2.46)	1.28 (0.67-2.46)	1.37 (0.72-2.61)	0.88
Colon cancer						
Events	90	84	88	99	111	
Person-years	199437	201443	201473	201679	202035	
HR (95% CI), Model 1	Reference	0.86 (0.64-1.16)	0.86 (0.64-1.16)	0.93 (0.70-1.24)	1.02 (0.77-1.34)	0.68
HR (95% CI), Model 2	Reference	0.89 (0.66-1.20)	0.90 (0.67-1.21)	0.98 (0.73-1.31)	1.06 (0.80-1.42)	0.71
Ovarian cancer§						
Events	25	32	23	27	22	
Person-years	119305	121599	119579	118722	116172	

HR (95% CI), Model 1	Reference	1.21 (0.72-2.04)	0.86 (0.49-1.52)	1.00 (0.58-1.72)	0.81 (0.46-1.44)	0.63
HR (95% CI), Model 2	Reference	1.23 (0.73-2.09)	0.89 (0.51-1.58)	1.05 (0.61-1.83)	0.88 (0.49-1.58)	0.72
Breast cancer[§]						
Events	208	219	223	195	199	
Person-years	112826	114007	111983	111109	108764	
HR (95% CI), Model 1	Reference	1.03 (0.85-1.25)	1.06 (0.88-1.28)	0.93 (0.76-1.13)	0.96 (0.79-1.17)	0.66
HR (95% CI), Model 2	Reference	1.05 (0.86-1.27)	1.08 (0.89-1.31)	0.94 (0.77-1.15)	0.98 (0.80-1.19)	0.67
Prostate cancer[¶]						
Events	208	239	288	272	302	
Person-years	90723	87685	89613	90181	92956	
HR (95% CI), Model 1	Reference	1.12 (0.93-1.34)	1.25 (1.05-1.50)	1.13 (0.95-1.36)	1.19 (1.00-1.42)	0.15
HR (95% CI), Model 2	Reference	1.08 (0.89-1.30)	1.19 (1.00-1.43)	1.07 (0.89-1.28)	1.12 (0.94-1.34)	0.39
Other cancers						
Events	593	602	668	649	666	
Person-years	185794	186784	187115	186968	187822	
HR (95% CI), Model 1	Reference	0.94 (0.84-1.06)	1.01 (0.90-1.13)	0.94 (0.84-1.06)	0.94 (0.84-1.06)	0.58
HR (95% CI), Model 2	Reference	0.97 (0.87-1.09)	1.04 (0.93-1.17)	0.99 (0.88-1.11)	0.99 (0.88-1.10)	0.76
Depression						
Events	71	56	46	61	48	
Person-years	187489	190072	191842	191876	192814	
HR (95% CI), Model 1	Reference	0.79 (0.56-1.13)	0.65 (0.45-0.94)	0.87 (0.62-1.23)	0.68 (0.47-0.99)	0.14
HR (95% CI), Model 2	Reference	0.85 (0.60-1.21)	0.70 (0.48-1.02)	0.96 (0.68-1.36)	0.76 (0.52-1.11)	0.30
Anxiety						
Events	493	407	390	416	354	
Person-years	193579	196345	196944	197205	198289	
HR (95% CI), Model 1	Reference	0.80 (0.70-0.91)	0.76 (0.66-0.87)	0.80 (0.70-0.91)	0.68 (0.59-0.78)	<0.0001
HR (95% CI), Model 2	Reference	0.83 (0.73-0.95)	0.79 (0.69-0.91)	0.85 (0.74-0.97)	0.72 (0.62-0.83)	0.0003
Schizophrenia						
Events	15	16	13	12	11	
Person-years	200491	202079	202480	202723	203072	
HR (95% CI), Model 1	Reference	1.04 (0.51-2.10)	0.82 (0.39-1.73)	0.74 (0.35-1.59)	0.67 (0.31-1.46)	0.76
HR (95% CI), Model 2	Reference	1.15 (0.56-2.33)	0.92 (0.44-1.95)	0.86 (0.40-1.86)	0.78 (0.35-1.73)	0.89
Alcohol use disorder						
Events	66	84	99	109	143	
Person-years	200061	201580	201815	202004	202159	
HR (95% CI), Model 1	Reference	1.26 (0.91-1.74)	1.45 (1.06-1.97)	1.57 (1.15-2.13)	2.01 (1.50-2.69)	<0.0001
HR (95% CI), Model 2	Reference	1.30 (0.94-1.79)	1.47 (1.08-2.01)	1.60 (1.17-2.18)	2.00 (1.48-2.69)	<0.0001
Psychoactive substance abuse						
Events	13	14	20	24	24	
Person-years	200938	202416	202692	203017	203449	
HR (95% CI), Model 1	Reference	1.09 (0.51-2.31)	1.54 (0.77-3.11)	1.85 (0.94-3.65)	1.83 (0.93-3.60)	0.24
HR (95% CI), Model 2	Reference	1.18 (0.55-2.52)	1.71 (0.85-3.46)	2.06 (1.04-4.10)	2.00 (1.00-3.99)	0.16
Epilepsy						
Events	51	40	43	49	51	

Person-years	199148	201036	201426	201623	202209	
HR (95% CI), Model 1	Reference	0.74 (0.49-1.13)	0.78 (0.52-1.16)	0.86 (0.58-1.27)	0.88 (0.59-1.29)	0.65
HR (95% CI), Model 2	Reference	0.75 (0.49-1.13)	0.78 (0.52-1.18)	0.86 (0.58-1.28)	0.86 (0.58-1.28)	0.68
Migraine						
Events	97	80	81	101	74	
Person-years	191744	194678	196297	196734	197792	
HR (95% CI), Model 1	Reference	0.80 (0.60-1.08)	0.81 (0.60-1.09)	1.01 (0.76-1.33)	0.74 (0.54-1.00)	0.14
HR (95% CI), Model 2	Reference	0.81 (0.60-1.09)	0.80 (0.60-1.08)	0.99 (0.75-1.32)	0.71 (0.52-0.97)	0.11
Dementia						
Events	109	113	114	121	124	
Person-years	200929	202444	202671	202893	203261	
HR (95% CI), Model 1	Reference	0.90 (0.69-1.17)	0.85 (0.65-1.10)	0.84 (0.65-1.09)	0.83 (0.64-1.07)	0.61
HR (95% CI), Model 2	Reference	0.91 (0.70-1.19)	0.86 (0.66-1.12)	0.86 (0.66-1.11)	0.85 (0.65-1.11)	0.72
Parkinson's disease						
Events	47	62	59	58	65	
Person-years	200655	202307	202511	202970	203175	
HR (95% CI), Model 1	Reference	1.15 (0.79-1.68)	1.02 (0.69-1.50)	0.94 (0.64-1.39)	1.02 (0.70-1.49)	0.88
HR (95% CI), Model 2	Reference	1.14 (0.78-1.66)	1.00 (0.68-1.48)	0.92 (0.63-1.36)	1.01 (0.69-1.48)	0.86
Multiple sclerosis						
Events	14	6	9	13	9	
Person-years	200453	201770	202460	202417	202884	
HR (95% CI), Model 1	Reference	0.42 (0.16-1.10)	0.64 (0.27-1.47)	0.92 (0.43-1.97)	0.64 (0.28-1.50)	0.39
HR (95% CI), Model 2	Reference	0.44 (0.17-1.15)	0.66 (0.28-1.54)	0.96 (0.44-2.08)	0.66 (0.28-1.55)	0.45
Bronchiectasis						
Events	84	86	84	109	98	
Person-years	200145	201836	202024	202150	202666	
HR (95% CI), Model 1	Reference	0.93 (0.69-1.25)	0.87 (0.64-1.17)	1.07 (0.81-1.42)	0.94 (0.70-1.26)	0.64
HR (95% CI), Model 2	Reference	0.92 (0.68-1.24)	0.85 (0.63-1.15)	1.04 (0.78-1.39)	0.90 (0.67-1.22)	0.63
Asthma						
Events	194	197	208	211	165	
Person-years	170654	173239	174336	176092	176521	
HR (95% CI), Model 1	Reference	0.98 (0.80-1.20)	1.02 (0.84-1.24)	1.01 (0.83-1.23)	0.78 (0.63-0.96)	0.0696
HR (95% CI), Model 2	Reference	1.06 (0.86-1.29)	1.11 (0.91-1.35)	1.13 (0.92-1.37)	0.88 (0.71-1.09)	0.13
COPD						
Events	260	251	245	217	251	
Person-years	197028	199055	199521	200061	199876	
HR (95% CI), Model 1	Reference	0.87 (0.73-1.04)	0.81 (0.68-0.96)	0.68 (0.57-0.81)	0.77 (0.64-0.91)	0.0005
HR (95% CI), Model 2	Reference	0.92 (0.78-1.10)	0.85 (0.71-1.01)	0.72 (0.60-0.86)	0.76 (0.64-0.91)	0.0017
CKD						
Events	735	583	566	550	512	
Person-years	196067	198330	199203	199435	200185	
HR (95% CI), Model 1	Reference	0.71 (0.63-0.79)	0.65 (0.58-0.72)	0.60 (0.53-0.67)	0.53 (0.48-0.60)	<0.0001
HR (95% CI), Model 2	Reference	0.78 (0.70-0.87)	0.74 (0.66-0.82)	0.70 (0.63-0.78)	0.64 (0.57-0.72)	<0.0001
Chronic liver disease						

Events	47	34	30	33	19	
Person-years	199884	201665	201852	202180	202589	
HR (95% CI), Model 1	Reference	0.69 (0.44-1.08)	0.60 (0.38-0.95)	0.65 (0.42-1.02)	0.37 (0.22-0.63)	0.0062
HR (95% CI), Model 2	Reference	0.79 (0.51-1.23)	0.71 (0.45-1.13)	0.80 (0.51-1.27)	0.47 (0.27-0.80)	0.0977
Irritable bowel syndrome						
Events	171	161	161	140	124	
Person-years	193458	196115	197073	197915	198578	
HR (95% CI), Model 1	Reference	0.91 (0.73-1.13)	0.90 (0.72-1.12)	0.77 (0.62-0.97)	0.68 (0.54-0.86)	0.0127
HR (95% CI), Model 2	Reference	0.95 (0.76-1.18)	0.94 (0.76-1.17)	0.82 (0.65-1.03)	0.73 (0.58-0.93)	0.0672
Inflammatory bowel disease						
Events	63	60	54	53	54	
Person-years	198232	200187	200756	201327	201786	
HR (95% CI), Model 1	Reference	0.93 (0.66-1.33)	0.83 (0.58-1.20)	0.81 (0.56-1.17)	0.82 (0.57-1.18)	0.73
HR (95% CI), Model 2	Reference	0.96 (0.67-1.37)	0.85 (0.59-1.23)	0.83 (0.57-1.21)	0.83 (0.57-1.21)	0.80
Treated constipation						
Events	463	441	440	437	457	
Person-years	196088	197178	198064	198073	199164	
HR (95% CI), Model 1	Reference	0.89 (0.78-1.01)	0.85 (0.75-0.97)	0.82 (0.72-0.93)	0.83 (0.73-0.95)	0.0193
HR (95% CI), Model 2	Reference	0.91 (0.80-1.04)	0.87 (0.77-1.00)	0.85 (0.74-0.97)	0.86 (0.76-0.99)	0.11
Dyspepsia						
Events	1192	1213	1096	1076	1045	
Person-years	165286	169926	171957	174183	175161	
HR (95% CI), Model 1	Reference	0.95 (0.87-1.02)	0.82 (0.76-0.89)	0.78 (0.72-0.85)	0.74 (0.68-0.80)	<0.0001
HR (95% CI), Model 2	Reference	0.98 (0.91-1.07)	0.86 (0.79-0.94)	0.83 (0.76-0.90)	0.80 (0.73-0.87)	<0.0001
Diverticular disease						
Events	990	1012	961	973	962	
Person-years	185776	187755	188925	188946	189765	
HR (95% CI), Model 1	Reference	0.97 (0.89-1.06)	0.89 (0.82-0.97)	0.88 (0.81-0.96)	0.85 (0.78-0.93)	0.0013
HR (95% CI), Model 2	Reference	1.00 (0.92-1.10)	0.92 (0.84-1.01)	0.92 (0.84-1.01)	0.89 (0.81-0.97)	0.0271
Pernicious anaemia						
Events	20	16	15	16	18	
Person-years	200205	202226	202470	202883	203317	
HR (95% CI), Model 1	Reference	0.77 (0.40-1.48)	0.70 (0.36-1.37)	0.73 (0.38-1.42)	0.81 (0.43-1.54)	0.85
HR (95% CI), Model 2	Reference	0.83 (0.43-1.61)	0.78 (0.40-1.53)	0.84 (0.43-1.64)	0.95 (0.49-1.82)	0.95
Fracture						
Events	89	95	102	103	128	
Person-years	199625	201255	201432	201793	201878	
HR (95% CI), Model 1	Reference	0.97 (0.72-1.29)	1.00 (0.75-1.32)	0.96 (0.72-1.27)	1.17 (0.89-1.54)	0.52
HR (95% CI), Model 2	Reference	0.93 (0.70-1.25)	0.95 (0.71-1.26)	0.89 (0.67-1.19)	1.07 (0.81-1.41)	0.69
Osteoporosis						
Events	704	762	779	735	752	
Person-years	177362	180142	180158	179743	180983	
HR (95% CI), Model 1	Reference	0.99 (0.89-1.10)	0.98 (0.88-1.08)	0.89 (0.80-0.99)	0.89 (0.80-0.98)	0.0367
HR (95% CI), Model 2	Reference	1.07 (0.96-1.19)	1.08 (0.98-1.20)	1.01 (0.91-1.12)	1.03 (0.93-1.15)	0.49

Meniere's disease						
Events	10	13	12	12	10	
Person-years	200488	202153	202485	202628	203064	
HR (95% CI), Model 1	Reference	1.27 (0.56-2.90)	1.15 (0.50-2.68)	1.14 (0.49-2.65)	0.94 (0.39-2.26)	0.96
HR (95% CI), Model 2	Reference	1.37 (0.60-3.15)	1.28 (0.55-3.00)	1.31 (0.56-3.07)	1.10 (0.45-2.71)	0.94
Eczema						
Events	148	188	156	164	166	
Person-years	191816	193890	193515	194513	194920	
HR (95% CI), Model 1	Reference	1.21 (0.97-1.50)	0.98 (0.78-1.23)	1.00 (0.80-1.25)	1.00 (0.80-1.25)	0.25
HR (95% CI), Model 2	Reference	1.25 (1.00-1.55)	1.01 (0.81-1.27)	1.04 (0.83-1.30)	1.02 (0.81-1.28)	0.19
Glaucoma						
Events	208	228	233	232	236	
Person-years	198218	199402	199462	199535	199601	
HR (95% CI), Model 1	Reference	1.00 (0.83-1.20)	0.97 (0.80-1.17)	0.92 (0.76-1.11)	0.91 (0.75-1.10)	0.78
HR (95% CI), Model 2	Reference	0.98 (0.82-1.19)	0.95 (0.79-1.15)	0.90 (0.75-1.09)	0.89 (0.74-1.08)	0.69
Cataract						
Events	922	1017	1065	1080	1092	
Person-years	189855	190769	190522	190222	190322	
HR (95% CI), Model 1	Reference	0.98 (0.89-1.07)	0.97 (0.89-1.06)	0.93 (0.85-1.01)	0.91 (0.83-0.99)	0.19
HR (95% CI), Model 2	Reference	1.00 (0.92-1.10)	1.01 (0.92-1.10)	0.97 (0.89-1.06)	0.96 (0.88-1.05)	0.77
AMD						
Events	155	158	211	199	200	
Person-years	199846	201530	201424	201764	201993	
HR (95% CI), Model 1	Reference	0.89 (0.72-1.12)	1.13 (0.92-1.39)	1.00 (0.81-1.24)	0.98 (0.80-1.21)	0.26
HR (95% CI), Model 2	Reference	0.92 (0.73-1.14)	1.16 (0.94-1.44)	1.04 (0.84-1.28)	1.02 (0.82-1.26)	0.24
Thyroid disorders						
Events	306	284	310	270	224	
Person-years	185825	188090	189211	190050	191200	
HR (95% CI), Model 1	Reference	0.88 (0.74-1.03)	0.94 (0.80-1.10)	0.80 (0.68-0.94)	0.66 (0.56-0.79)	<0.0001
HR (95% CI), Model 2	Reference	0.92 (0.79-1.09)	1.01 (0.86-1.18)	0.87 (0.74-1.03)	0.73 (0.61-0.87)	0.0023
Prostate disorders[¶]						
Events	381	415	466	458	425	
Person-years	84652	81600	83060	83505	86648	
HR (95% CI), Model 1	Reference	1.05 (0.91-1.20)	1.10 (0.96-1.26)	1.03 (0.90-1.18)	0.89 (0.78-1.03)	0.0345
HR (95% CI), Model 2	Reference	1.05 (0.91-1.20)	1.10 (0.96-1.26)	1.03 (0.89-1.18)	0.89 (0.77-1.03)	0.0339

AMD, age related macular degeneration; CI, confidence interval; CKD, chronic kidney disease; COPD, chronic obstructive pulmonary disease; HR, hazard ratio.

*Cox proportional hazard regression models were used to examine associations of the Anti-Empirical Dietary Inflammatory Index (quintiles) with the incidence of individual chronic diseases. The statistical tests were two-sided. Model 1 was adjusted for age and sex; Model 2 was adjusted for Model 1 plus ethnicity, education, income, smoking, alcohol consumption, sleep, physical activity, and GRS for longevity; Model 3 was adjusted for Model 2 and total energy intake.

†Cardiovascular disease includes coronary heart disease, heart failure, atrial fibrillation, other cardiac disease, stroke, and peripheral vascular disease.

[‡]All cancers encompass any type of cancer except for non-melanoma skin cancer.

[§]These analyses were conducted among women only.

[¶]These analyses were conducted among men only.

Table S7. The association between the Alternate Healthy Eating Index-2010 and the incidence of individual chronic diseases among individuals by excluding those developed the corresponding disease in the first 4 years of follow-up

	Alternate Healthy Eating Index-2010					P-value
	Quintile 1	Quintile 2	Quintile 3	Quintile 4	Quintile 5	for trend*
Cardiovascular disease†						
Events	1067	1053	986	992	994	
Person-years	184030	187117	187759	189807	190358	
HR (95% CI), Model 1	Reference	0.94 (0.87-1.03)	0.86 (0.79-0.94)	0.86 (0.79-0.94)	0.86 (0.79-0.94)	0.0006
HR (95% CI), Model 2	Reference	0.98 (0.90-1.07)	0.90 (0.83-0.98)	0.91 (0.84-1.00)	0.92 (0.84-1.00)	0.0718
Coronary heart disease						
Events	607	628	578	553	615	
Person-years	192715	195174	195392	196801	197694	
HR (95% CI), Model 1	Reference	1.01 (0.91-1.13)	0.92 (0.82-1.03)	0.89 (0.79-1.00)	0.99 (0.89-1.11)	0.0919
HR (95% CI), Model 2	Reference	1.06 (0.94-1.18)	0.98 (0.87-1.09)	0.95 (0.85-1.07)	1.08 (0.96-1.21)	0.15
Heart failure						
Events	228	217	210	206	204	
Person-years	199282	201250	201256	202043	202791	
HR (95% CI), Model 1	Reference	0.90 (0.75-1.09)	0.85 (0.70-1.02)	0.83 (0.69-1.00)	0.81 (0.67-0.98)	0.19
HR (95% CI), Model 2	Reference	0.96 (0.80-1.16)	0.93 (0.77-1.12)	0.92 (0.76-1.11)	0.92 (0.76-1.11)	0.88
Atrial fibrillation						
Events	93	99	79	86	75	
Person-years	195953	198077	198482	199307	199825	
HR (95% CI), Model 1	Reference	1.02 (0.77-1.36)	0.79 (0.59-1.07)	0.87 (0.64-1.16)	0.75 (0.55-1.02)	0.18
HR (95% CI), Model 2	Reference	1.06 (0.80-1.41)	0.84 (0.62-1.14)	0.93 (0.69-1.25)	0.83 (0.61-1.13)	0.41
Other cardiac disease						
Events	591	530	503	503	500	
Person-years	196532	198439	198870	200165	200462	
HR (95% CI), Model 1	Reference	0.86 (0.76-0.96)	0.79 (0.70-0.89)	0.79 (0.70-0.89)	0.78 (0.69-0.88)	<0.0001
HR (95% CI), Model 2	Reference	0.89 (0.79-1.01)	0.84 (0.74-0.94)	0.84 (0.75-0.95)	0.84 (0.74-0.95)	0.0145
Stroke						
Events	41	37	41	33	35	
Person-years	199322	200926	201249	202109	202883	
HR (95% CI), Model 1	Reference	0.86 (0.55-1.34)	0.92 (0.60-1.42)	0.74 (0.46-1.17)	0.77 (0.49-1.21)	0.67
HR (95% CI), Model 2	Reference	0.88 (0.57-1.38)	0.95 (0.61-1.47)	0.75 (0.47-1.19)	0.78 (0.49-1.23)	0.70
Peripheral vascular disease						
Events	131	130	110	117	117	
Person-years	199678	201398	201847	202471	202953	
HR (95% CI), Model 1	Reference	0.95 (0.75-1.21)	0.79 (0.61-1.01)	0.83 (0.65-1.07)	0.82 (0.64-1.06)	0.27
HR (95% CI), Model 2	Reference	0.99 (0.77-1.26)	0.81 (0.63-1.05)	0.87 (0.67-1.12)	0.85 (0.66-1.10)	0.41
Hypertension						
Events	247	187	207	164	177	
Person-years	144375	148003	149437	150808	152070	
HR (95% CI), Model 1	Reference	0.72 (0.59-0.87)	0.77 (0.64-0.93)	0.61 (0.50-0.74)	0.65 (0.53-0.79)	<0.0001

HR (95% CI), Model 2	Reference	0.76 (0.63-0.92)	0.83 (0.68-0.99)	0.67 (0.55-0.82)	0.72 (0.59-0.88)	0.0007
Diabetes						
Events	377	332	303	299	273	
Person-years	191604	193722	194231	193903	194644	
HR (95% CI), Model 1	Reference	0.87 (0.75-1.01)	0.79 (0.67-0.91)	0.78 (0.67-0.91)	0.72 (0.61-0.84)	0.0003
HR (95% CI), Model 2	Reference	0.97 (0.83-1.12)	0.92 (0.79-1.07)	0.94 (0.81-1.10)	0.87 (0.75-1.03)	0.55
All cancers[‡]						
Events	750	748	767	702	674	
Person-years	179521	183975	183533	184465	185383	
HR (95% CI), Model 1	Reference	0.93 (0.84-1.03)	0.93 (0.84-1.03)	0.84 (0.76-0.93)	0.79 (0.71-0.88)	<0.0001
HR (95% CI), Model 2	Reference	0.96 (0.87-1.07)	0.98 (0.88-1.08)	0.89 (0.81-0.99)	0.85 (0.77-0.95)	0.0163
Non-melanoma skin cancer						
Events	463	440	438	482	435	
Person-years	194806	196459	196368	197226	197932	
HR (95% CI), Model 1	Reference	0.91 (0.80-1.03)	0.88 (0.77-1.01)	0.97 (0.85-1.10)	0.86 (0.76-0.98)	0.14
HR (95% CI), Model 2	Reference	0.89 (0.78-1.02)	0.86 (0.75-0.98)	0.94 (0.83-1.08)	0.83 (0.73-0.95)	0.0482
Melanoma						
Events	60	50	62	57	66	
Person-years	198738	200487	200372	201096	201758	
HR (95% CI), Model 1	Reference	0.80 (0.55-1.16)	0.97 (0.68-1.39)	0.89 (0.62-1.28)	1.01 (0.71-1.44)	0.71
HR (95% CI), Model 2	Reference	0.79 (0.54-1.16)	0.97 (0.68-1.39)	0.89 (0.62-1.29)	1.03 (0.72-1.48)	0.57
Lung cancer						
Events	100	72	64	73	58	
Person-years	200122	202121	202174	202928	203509	
HR (95% CI), Model 1	Reference	0.66 (0.49-0.90)	0.56 (0.41-0.77)	0.63 (0.46-0.85)	0.48 (0.35-0.67)	0.0001
HR (95% CI), Model 2	Reference	0.76 (0.57-1.01)	0.64 (0.48-0.87)	0.77 (0.57-1.03)	0.60 (0.44-0.82)	0.0100
Stomach cancer						
Events	20	18	14	12	14	
Person-years	200943	202502	202633	203270	203947	
HR (95% CI), Model 1	Reference	0.90 (0.48-1.71)	0.70 (0.35-1.39)	0.61 (0.30-1.25)	0.72 (0.36-1.43)	0.64
HR (95% CI), Model 2	Reference	0.94 (0.50-1.78)	0.75 (0.38-1.50)	0.65 (0.32-1.35)	0.79 (0.39-1.59)	0.78
Oesophageal cancer						
Events	29	23	17	15	20	
Person-years	200759	202520	202569	203310	203929	
HR (95% CI), Model 1	Reference	0.78 (0.45-1.35)	0.57 (0.31-1.04)	0.51 (0.27-0.95)	0.68 (0.38-1.20)	0.20
HR (95% CI), Model 2	Reference	0.87 (0.50-1.51)	0.65 (0.36-1.20)	0.60 (0.32-1.13)	0.83 (0.46-1.49)	0.48
Colon cancer						
Events	87	113	113	84	75	
Person-years	199214	201247	201087	201793	202726	
HR (95% CI), Model 1	Reference	1.24 (0.93-1.64)	1.21 (0.91-1.60)	0.89 (0.66-1.20)	0.78 (0.57-1.06)	0.0062
HR (95% CI), Model 2	Reference	1.28 (0.96-1.69)	1.27 (0.96-1.68)	0.94 (0.69-1.27)	0.84 (0.61-1.15)	0.0118
Ovarian cancer[§]						
Events	17	30	26	22	34	
Person-years	100570	114204	119920	126547	134134	

HR (95% CI), Model 1	Reference	1.48 (0.81-2.68)	1.19 (0.64-2.19)	0.93 (0.49-1.76)	1.33 (0.74-2.38)	0.47
HR (95% CI), Model 2	Reference	1.48 (0.82-2.69)	1.20 (0.65-2.21)	0.94 (0.50-1.77)	1.33 (0.74-2.40)	0.46
Breast cancer[§]						
Events	202	208	210	212	212	
Person-years	93943	107506	112724	119139	125377	
HR (95% CI), Model 1	Reference	0.88 (0.73-1.07)	0.84 (0.69-1.02)	0.80 (0.66-0.97)	0.75 (0.62-0.91)	0.0432
HR (95% CI), Model 2	Reference	0.90 (0.74-1.09)	0.86 (0.71-1.05)	0.83 (0.68-1.00)	0.78 (0.64-0.95)	0.14
Prostate cancer[¶]						
Events	286	269	288	250	216	
Person-years	107855	94358	88986	83440	76517	
HR (95% CI), Model 1	Reference	1.01 (0.85-1.19)	1.11 (0.94-1.31)	1.01 (0.86-1.20)	0.94 (0.79-1.12)	0.46
HR (95% CI), Model 2	Reference	0.98 (0.83-1.16)	1.07 (0.91-1.27)	0.99 (0.83-1.17)	0.92 (0.77-1.10)	0.53
Other cancers						
Events	666	656	656	620	580	
Person-years	183347	187135	187233	188072	188695	
HR (95% CI), Model 1	Reference	0.92 (0.83-1.03)	0.90 (0.81-1.01)	0.84 (0.75-0.94)	0.77 (0.69-0.87)	0.0001
HR (95% CI), Model 2	Reference	0.96 (0.86-1.07)	0.94 (0.85-1.05)	0.89 (0.80-1.00)	0.83 (0.74-0.93)	0.0156
Depression						
Events	64	61	39	44	74	
Person-years	188001	190590	191344	192208	191948	
HR (95% CI), Model 1	Reference	0.92 (0.65-1.31)	0.58 (0.39-0.87)	0.64 (0.44-0.95)	1.06 (0.76-1.49)	0.0052
HR (95% CI), Model 2	Reference	0.97 (0.68-1.38)	0.62 (0.41-0.92)	0.69 (0.47-1.02)	1.14 (0.81-1.61)	0.0082
Anxiety						
Events	432	385	393	394	456	
Person-years	194276	196477	196756	197524	197329	
HR (95% CI), Model 1	Reference	0.83 (0.73-0.96)	0.83 (0.72-0.95)	0.81 (0.70-0.93)	0.91 (0.79-1.04)	0.0120
HR (95% CI), Model 2	Reference	0.86 (0.75-0.99)	0.87 (0.75-0.99)	0.85 (0.74-0.98)	0.95 (0.83-1.08)	0.0812
Schizophrenia						
Events	19	11	11	13	13	
Person-years	200432	202027	202088	202842	203456	
HR (95% CI), Model 1	Reference	0.56 (0.26-1.17)	0.54 (0.26-1.15)	0.63 (0.31-1.28)	0.62 (0.30-1.26)	0.41
HR (95% CI), Model 2	Reference	0.62 (0.30-1.31)	0.63 (0.30-1.33)	0.76 (0.37-1.55)	0.76 (0.37-1.57)	0.70
Alcohol use disorder						
Events	189	107	81	80	44	
Person-years	198679	201285	201731	202517	203406	
HR (95% CI), Model 1	Reference	0.57 (0.45-0.73)	0.43 (0.33-0.56)	0.43 (0.33-0.57)	0.24 (0.17-0.34)	<0.0001
HR (95% CI), Model 2	Reference	0.61 (0.48-0.77)	0.47 (0.36-0.61)	0.49 (0.37-0.64)	0.28 (0.20-0.39)	<0.0001
Psychoactive substance abuse						
Events	35	23	10	17	10	
Person-years	200480	202404	202541	203288	203799	
HR (95% CI), Model 1	Reference	0.68 (0.40-1.16)	0.30 (0.15-0.61)	0.53 (0.29-0.94)	0.32 (0.16-0.65)	0.0013
HR (95% CI), Model 2	Reference	0.77 (0.45-1.30)	0.34 (0.17-0.70)	0.63 (0.35-1.13)	0.38 (0.19-0.78)	0.0110
Epilepsy						
Events	52	49	45	43	45	

Person-years	199250	201119	200919	201864	202291	
HR (95% CI), Model 1	Reference	0.91 (0.62-1.35)	0.82 (0.55-1.23)	0.78 (0.52-1.17)	0.81 (0.54-1.21)	0.74
HR (95% CI), Model 2	Reference	0.93 (0.63-1.38)	0.85 (0.57-1.28)	0.81 (0.54-1.22)	0.86 (0.57-1.29)	0.87
Migraine						
Events	94	71	92	88	88	
Person-years	194800	195976	195470	195418	195581	
HR (95% CI), Model 1	Reference	0.72 (0.53-0.98)	0.92 (0.69-1.23)	0.86 (0.64-1.16)	0.84 (0.63-1.13)	0.31
HR (95% CI), Model 2	Reference	0.72 (0.53-0.99)	0.93 (0.70-1.24)	0.88 (0.65-1.18)	0.85 (0.63-1.14)	0.32
Dementia						
Events	131	108	121	98	123	
Person-years	200507	202427	202453	203181	203630	
HR (95% CI), Model 1	Reference	0.75 (0.58-0.97)	0.80 (0.62-1.03)	0.63 (0.49-0.82)	0.77 (0.60-0.98)	0.0153
HR (95% CI), Model 2	Reference	0.76 (0.59-0.98)	0.81 (0.63-1.04)	0.64 (0.49-0.83)	0.76 (0.59-0.98)	0.0193
Parkinson’s disease						
Events	61	65	54	53	58	
Person-years	200551	202285	202217	202911	203653	
HR (95% CI), Model 1	Reference	1.02 (0.72-1.44)	0.82 (0.57-1.19)	0.81 (0.56-1.17)	0.88 (0.61-1.27)	0.62
HR (95% CI), Model 2	Reference	1.01 (0.71-1.43)	0.80 (0.56-1.16)	0.79 (0.54-1.14)	0.85 (0.59-1.22)	0.52
Multiple sclerosis						
Events	9	19	7	8	8	
Person-years	200083	201842	202043	202784	203232	
HR (95% CI), Model 1	Reference	2.01 (0.91-4.44)	0.73 (0.27-1.96)	0.81 (0.31-2.11)	0.79 (0.30-2.07)	0.0485
HR (95% CI), Model 2	Reference	2.14 (2.14-2.14)	0.78 (0.29-2.12)	0.90 (0.34-2.34)	0.88 (0.34-2.33)	0.36
Bronchiectasis						
Events	119	98	81	74	89	
Person-years	199894	201726	201747	202362	203093	
HR (95% CI), Model 1	Reference	0.74 (0.57-0.97)	0.58 (0.44-0.77)	0.51 (0.38-0.69)	0.59 (0.44-0.77)	<0.0001
HR (95% CI), Model 2	Reference	0.76 (0.58-1.00)	0.60 (0.45-0.80)	0.53 (0.40-0.71)	0.60 (0.45-0.79)	0.0001
Asthma						
Events	216	189	186	193	191	
Person-years	170774	173532	174805	175191	176540	
HR (95% CI), Model 1	Reference	0.83 (0.68-1.01)	0.80 (0.65-0.97)	0.81 (0.67-0.99)	0.78 (0.64-0.95)	0.09214
HR (95% CI), Model 2	Reference	0.87 (0.71-1.06)	0.84 (0.69-1.03)	0.86 (0.71-1.05)	0.84 (0.68-1.02)	0.37073
COPD						
Events	328	253	243	196	204	
Person-years	196500	198635	199167	200278	200960	
HR (95% CI), Model 1	Reference	0.72 (0.61-0.85)	0.67 (0.57-0.79)	0.53 (0.45-0.64)	0.54 (0.45-0.65)	<0.0001
HR (95% CI), Model 2	Reference	0.82 (0.69-0.97)	0.79 (0.67-0.93)	0.65 (0.55-0.78)	0.68 (0.57-0.82)	<0.0001
CKD						
Events	644	635	571	561	535	
Person-years	196401	197905	198645	200018	200251	
HR (95% CI), Model 1	Reference	0.93 (0.84-1.04)	0.81 (0.72-0.90)	0.78 (0.70-0.87)	0.73 (0.65-0.82)	<0.0001
HR (95% CI), Model 2	Reference	0.99 (0.88-1.10)	0.88 (0.78-0.98)	0.86 (0.76-0.96)	0.81 (0.72-0.91)	0.0009
Chronic liver disease						
Events	42	37	33	24	27	

Person-years	199713	201441	201717	202300	203000	
HR (95% CI), Model 1	Reference	0.89 (0.57-1.39)	0.80 (0.51-1.27)	0.59 (0.36-0.98)	0.68 (0.42-1.10)	0.26
HR (95% CI), Model 2	Reference	0.98 (0.63-1.52)	0.93 (0.59-1.47)	0.70 (0.42-1.16)	0.84 (0.51-1.37)	0.67
Irritable bowel syndrome						
Events	180	145	121	152	159	
Person-years	195187	196312	197029	197184	197427	
HR (95% CI), Model 1	Reference	0.75 (0.60-0.93)	0.61 (0.48-0.76)	0.74 (0.59-0.92)	0.75 (0.60-0.92)	0.0006
HR (95% CI), Model 2	Reference	0.78 (0.62-0.97)	0.63 (0.50-0.80)	0.78 (0.63-0.97)	0.79 (0.64-0.98)	0.0037
Inflammatory bowel disease						
Events	64	56	54	57	53	
Person-years	198107	200072	200645	201326	202140	
HR (95% CI), Model 1	Reference	0.87 (0.61-1.24)	0.83 (0.58-1.20)	0.88 (0.61-1.26)	0.82 (0.56-1.18)	0.83
HR (95% CI), Model 2	Reference	0.89 (0.62-1.27)	0.85 (0.59-1.23)	0.90 (0.62-1.29)	0.81 (0.56-1.18)	0.86
Treated constipation						
Events	472	471	413	403	479	
Person-years	195147	197847	197820	198707	199045	
HR (95% CI), Model 1	Reference	0.94 (0.83-1.07)	0.80 (0.70-0.91)	0.77 (0.67-0.88)	0.90 (0.79-1.02)	0.0004
HR (95% CI), Model 2	Reference	0.96 (0.84-1.09)	0.82 (0.72-0.94)	0.79 (0.69-0.91)	0.92 (0.81-1.05)	0.0020
Dyspepsia						
Events	1181	1108	1104	1100	1129	
Person-years	166511	170552	172920	172989	173540	
HR (95% CI), Model 1	Reference	0.88 (0.81-0.96)	0.85 (0.78-0.92)	0.84 (0.77-0.91)	0.84 (0.78-0.91)	<0.0001
HR (95% CI), Model 2	Reference	0.91 (0.84-0.99)	0.89 (0.81-0.96)	0.88 (0.81-0.96)	0.89 (0.82-0.97)	0.0137
Diverticular disease						
Events	1073	1017	991	922	895	
Person-years	184762	187365	188552	189632	190856	
HR (95% CI), Model 1	Reference	0.90 (0.83-0.99)	0.86 (0.79-0.94)	0.79 (0.72-0.86)	0.75 (0.68-0.82)	<0.0001
HR (95% CI), Model 2	Reference	0.94 (0.86-1.03)	0.91 (0.83-0.99)	0.85 (0.78-0.93)	0.82 (0.75-0.90)	<0.0001
Pernicious anaemia						
Events	26	15	16	12	16	
Person-years	200375	202167	202258	202926	203375	
HR (95% CI), Model 1	Reference	0.54 (0.28-1.01)	0.55 (0.30-1.03)	0.40 (0.20-0.80)	0.51 (0.27-0.96)	0.0575
HR (95% CI), Model 2	Reference	0.56 (0.29-1.05)	0.58 (0.31-1.08)	0.43 (0.21-0.85)	0.54 (0.29-1.03)	0.10
Fracture						
Events	96	98	103	107	113	
Person-years	199295	201251	201245	201919	202274	
HR (95% CI), Model 1	Reference	0.93 (0.70-1.23)	0.93 (0.70-1.23)	0.94 (0.71-1.24)	0.95 (0.72-1.25)	0.98
HR (95% CI), Model 2	Reference	0.93 (0.70-1.23)	0.92 (0.70-1.22)	0.93 (0.71-1.23)	0.93 (0.71-1.23)	0.98
Osteoporosis						
Events	801	773	739	690	729	
Person-years	177636	179859	179836	179958	181100	
HR (95% CI), Model 1	Reference	0.89 (0.81-0.98)	0.82 (0.74-0.90)	0.75 (0.67-0.83)	0.76 (0.69-0.84)	<0.0001
HR (95% CI), Model 2	Reference	0.93 (0.84-1.02)	0.87 (0.79-0.96)	0.80 (0.72-0.89)	0.83 (0.75-0.92)	0.0002
Meniere’s disease						

Events	12	11	5	13	16	
Person-years	200332	202146	202196	202730	203415	
HR (95% CI), Model 1	Reference	0.87 (0.38-1.98)	0.39 (0.14-1.10)	0.99 (0.45-2.18)	1.18 (0.55-2.51)	0.30
HR (95% CI), Model 2	Reference	0.90 (0.40-2.04)	0.41 (0.14-1.17)	1.05 (0.47-2.32)	1.28 (0.60-2.76)	0.28
Eczema						
Events	198	167	159	133	165	
Person-years	191378	193657	193824	194851	194944	
HR (95% CI), Model 1	Reference	0.81 (0.66-1.00)	0.76 (0.62-0.94)	0.63 (0.51-0.79)	0.77 (0.63-0.95)	0.0014
HR (95% CI), Model 2	Reference	0.83 (0.67-1.02)	0.78 (0.64-0.97)	0.65 (0.52-0.81)	0.80 (0.64-0.98)	0.0052
Glaucoma						
Events	201	224	210	242	260	
Person-years	197761	199216	199302	199712	200227	
HR (95% CI), Model 1	Reference	1.05 (0.87-1.27)	0.95 (0.78-1.15)	1.08 (0.89-1.30)	1.13 (0.94-1.36)	0.37
HR (95% CI), Model 2	Reference	1.05 (0.87-1.27)	0.94 (0.78-1.15)	1.07 (0.89-1.30)	1.12 (0.92-1.35)	0.44
Cataract						
Events	964	1027	1026	1046	1113	
Person-years	189353	190821	190535	190702	190279	
HR (95% CI), Model 1	Reference	0.96 (0.88-1.04)	0.91 (0.83-0.99)	0.90 (0.82-0.98)	0.92 (0.84-1.00)	0.11
HR (95% CI), Model 2	Reference	0.98 (0.89-1.07)	0.93 (0.85-1.01)	0.92 (0.84-1.01)	0.93 (0.85-1.02)	0.26
AMD						
Events	164	178	196	186	199	
Person-years	199649	201311	201411	201853	202333	
HR (95% CI), Model 1	Reference	0.98 (0.79-1.21)	1.02 (0.82-1.25)	0.93 (0.76-1.15)	0.95 (0.77-1.17)	0.93
HR (95% CI), Model 2	Reference	1.00 (0.81-1.24)	1.05 (0.85-1.29)	0.97 (0.78-1.20)	0.98 (0.79-1.21)	0.94
Thyroid disorders						
Events	264	244	291	307	288	
Person-years	188652	189729	189023	188761	188212	
HR (95% CI), Model 1	Reference	0.86 (0.72-1.02)	1.00 (0.85-1.18)	1.03 (0.88-1.22)	0.94 (0.80-1.12)	0.23
HR (95% CI), Model 2	Reference	0.88 (0.74-1.05)	1.04 (0.88-1.24)	1.09 (0.92-1.29)	1.01 (0.85-1.19)	0.18
Prostate disorders¶						
Events	477	459	429	421	359	
Person-years	100393	87890	83287	77248	70648	
HR (95% CI), Model 1	Reference	1.03 (0.90-1.17)	0.97 (0.85-1.11)	1.02 (0.90-1.17)	0.94 (0.82-1.08)	0.72
HR (95% CI), Model 2	Reference	1.03 (0.91-1.17)	0.97 (0.85-1.11)	1.03 (0.90-1.17)	0.95 (0.82-1.09)	0.72

AMD, age related macular degeneration; CI, confidence interval; CKD, chronic kidney disease; COPD, chronic obstructive pulmonary disease; HR, hazard ratio.

*Cox proportional hazard regression models were used to examine associations of the Alternate Healthy Eating Index-2010 (quintiles) with the incidence of individual chronic diseases. The statistical tests were two-sided. Model 1 was adjusted for age and sex; Model 2 was adjusted for Model 1 plus ethnicity, education, income, smoking, alcohol consumption, sleep, physical activity, and GRS for longevity; Model 3 was adjusted for Model 2 and total energy intake.

†Cardiovascular disease includes coronary heart disease, heart failure, atrial fibrillation, other cardiac disease, stroke, and peripheral vascular disease.

[‡]All cancers encompass any type of cancer except for non-melanoma skin cancer.

[§]These analyses were conducted among women only.

[¶]These analyses were conducted among men only.

Table S8. The association between the Healthful Plant-based Diet Index and the incidence of individual chronic diseases among individuals by excluding those developed the corresponding disease in the first 4 years of follow-up

	Healthful Plant-based Diet Index					
	Quintile 1	Quintile 2	Quintile 3	Quintile 4	Quintile 5	P-value
Cardiovascular disease†						
Events	1365	362	1290	1124	951	
Person-years	228447	68654	238025	211185	192760	
HR (95% CI), Model 1	Reference	0.86 (0.77-0.97)	0.89 (0.82-0.96)	0.90 (0.83-0.97)	0.86 (0.79-0.94)	0.0036
HR (95% CI), Model 2	Reference	0.89 (0.79-0.99)	0.93 (0.86-1.00)	0.95 (0.87-1.03)	0.93 (0.85-1.01)	0.19
Coronary heart disease						
Events	825	225	757	648	526	
Person-years	239071	71712	247586	219857	199549	
HR (95% CI), Model 1	Reference	0.90 (0.78-1.04)	0.89 (0.81-0.99)	0.90 (0.81-1.00)	0.85 (0.76-0.95)	0.0466
HR (95% CI), Model 2	Reference	0.93 (0.80-1.08)	0.94 (0.85-1.04)	0.96 (0.86-1.07)	0.93 (0.83-1.04)	0.66
Heart failure						
Events	306	86	270	217	186	
Person-years	247756	73857	255186	225479	204344	
HR (95% CI), Model 1	Reference	0.92 (0.72-1.16)	0.84 (0.71-0.99)	0.79 (0.66-0.94)	0.79 (0.65-0.95)	0.0452
HR (95% CI), Model 2	Reference	0.97 (0.76-1.23)	0.92 (0.78-1.09)	0.88 (0.74-1.05)	0.92 (0.76-1.12)	0.71
Atrial fibrillation						
Events	124	29	111	96	72	
Person-years	243406	72769	251583	222168	201718	
HR (95% CI), Model 1	Reference	0.78 (0.52-1.17)	0.88 (0.68-1.14)	0.92 (0.70-1.20)	0.82 (0.61-1.11)	0.63
HR (95% CI), Model 2	Reference	0.80 (0.53-1.20)	0.93 (0.72-1.20)	0.98 (0.74-1.28)	0.90 (0.66-1.22)	0.83
Other cardiac disease						
Events	686	212	655	586	488	
Person-years	244109	72922	252115	223121	202199	
HR (95% CI), Model 1	Reference	1.00 (0.86-1.17)	0.90 (0.81-1.00)	0.93 (0.83-1.04)	0.88 (0.78-0.99)	0.16
HR (95% CI), Model 2	Reference	1.03 (0.88-1.20)	0.95 (0.85-1.06)	0.99 (0.88-1.11)	0.96 (0.85-1.09)	0.78
Stroke						
Events	46	13	46	54	28	
Person-years	247592	74001	255493	225328	204075	
HR (95% CI), Model 1	Reference	0.92 (0.49-1.70)	0.94 (0.62-1.41)	1.27 (0.85-1.90)	0.75 (0.46-1.22)	0.23
HR (95% CI), Model 2	Reference	0.94 (0.51-1.75)	0.98 (0.65-1.48)	1.34 (0.89-2.01)	0.81 (0.50-1.33)	0.24
Peripheral vascular disease						
Events	162	42	151	137	113	
Person-years	248182	74083	255826	225884	204371	
HR (95% CI), Model 1	Reference	0.83 (0.59-1.17)	0.86 (0.69-1.08)	0.88 (0.70-1.11)	0.81 (0.63-1.04)	0.51
HR (95% CI), Model 2	Reference	0.85 (0.61-1.19)	0.88 (0.71-1.11)	0.91 (0.72-1.16)	0.85 (0.66-1.09)	0.71
Hypertension						
Events	263	81	264	202	172	
Person-years	176187	53613	187406	169208	158280	
HR (95% CI), Model 1	Reference	0.97 (0.76-1.25)	0.89 (0.75-1.06)	0.75 (0.63-0.91)	0.69 (0.57-0.84)	0.0015

HR (95% CI), Model 2	Reference	1.01 (0.79-1.30)	0.96 (0.81-1.14)	0.83 (0.68-1.00)	0.79 (0.64-0.97)	0.0778
Diabetes						
Events	514	139	414	279	238	
Person-years	236120	71131	245563	217514	197777	
HR (95% CI), Model 1	Reference	0.89 (0.74-1.07)	0.77 (0.68-0.88)	0.60 (0.51-0.69)	0.57 (0.49-0.67)	<0.0001
HR (95% CI), Model 2	Reference	0.98 (0.81-1.18)	0.91 (0.79-1.03)	0.73 (0.63-0.85)	0.77 (0.65-0.90)	0.0003
All cancers[‡]						
Events	953	279	899	815	695	
Person-years	224686	67321	232247	205322	187301	
HR (95% CI), Model 1	Reference	0.94 (0.83-1.08)	0.87 (0.79-0.95)	0.89 (0.81-0.98)	0.84 (0.76-0.93)	0.0064
HR (95% CI), Model 2	Reference	0.97 (0.85-1.11)	0.90 (0.82-0.99)	0.93 (0.84-1.02)	0.90 (0.81-1.00)	0.16
Non-melanoma skin cancer						
Events	553	183	569	496	457	
Person-years	242019	72361	248886	220084	199441	
HR (95% CI), Model 1	Reference	1.07 (0.91-1.27)	0.97 (0.86-1.09)	0.97 (0.86-1.10)	1.02 (0.90-1.16)	0.74
HR (95% CI), Model 2	Reference	1.06 (0.90-1.26)	0.95 (0.85-1.07)	0.95 (0.84-1.08)	0.99 (0.87-1.13)	0.66
Melanoma						
Events	71	21	77	65	61	
Person-years	247278	73667	254369	224156	202980	
HR (95% CI), Model 1	Reference	0.96 (0.59-1.57)	1.01 (0.73-1.40)	0.96 (0.68-1.36)	1.01 (0.70-1.44)	1.00
HR (95% CI), Model 2	Reference	0.96 (0.59-1.56)	1.01 (0.73-1.39)	0.96 (0.68-1.35)	1.01 (0.71-1.43)	1.00
Lung cancer						
Events	95	40	81	80	71	
Person-years	248965	74184	256380	226423	204903	
HR (95% CI), Model 1	Reference	1.33 (0.92-1.93)	0.76 (0.56-1.03)	0.84 (0.62-1.13)	0.82 (0.60-1.13)	0.0371
HR (95% CI), Model 2	Reference	1.41 (0.99-2.00)	0.88 (0.67-1.17)	0.91 (0.68-1.22)	0.95 (0.70-1.29)	0.12
Stomach cancer						
Events	21	7	26	12	12	
Person-years	249592	74475	257049	226875	205304	
HR (95% CI), Model 1	Reference	1.16 (0.49-2.72)	1.30 (0.73-2.33)	0.74 (0.36-1.51)	0.90 (0.43-1.87)	0.54
HR (95% CI), Model 2	Reference	1.22 (0.52-2.86)	1.40 (0.78-2.51)	0.81 (0.39-1.67)	1.02 (0.48-2.13)	0.56
Oesophageal cancer						
Events	32	7	20	23	22	
Person-years	249482	74463	256929	226906	205306	
HR (95% CI), Model 1	Reference	0.74 (0.33-1.69)	0.64 (0.36-1.12)	0.88 (0.51-1.53)	1.01 (0.57-1.79)	0.52
HR (95% CI), Model 2	Reference	0.81 (0.36-1.84)	0.73 (0.42-1.29)	1.05 (0.61-1.83)	1.30 (0.73-2.31)	0.44
Colon cancer						
Events	134	29	119	112	78	
Person-years	247505	73849	254964	225447	204301	
HR (95% CI), Model 1	Reference	0.70 (0.47-1.05)	0.83 (0.65-1.07)	0.90 (0.69-1.16)	0.71 (0.53-0.94)	0.13
HR (95% CI), Model 2	Reference	0.73 (0.48-1.08)	0.87 (0.67-1.11)	0.94 (0.73-1.22)	0.76 (0.57-1.02)	0.26
Ovarian cancer[§]						
Events	29	11	30	35	24	
Person-years	113238	38717	148118	147574	147728	

HR (95% CI), Model 1	Reference	1.07 (0.54-2.15)	0.75 (0.45-1.25)	0.86 (0.52-1.42)	0.59 (0.34-1.03)	0.32
HR (95% CI), Model 2	Reference	1.09 (0.54-2.19)	0.77 (0.46-1.29)	0.89 (0.54-1.48)	0.62 (0.36-1.09)	0.42
Breast cancer[§]						
Events	201	82	245	269	247	
Person-years	106347	36576	138852	138149	138765	
HR (95% CI), Model 1	Reference	1.18 (0.91-1.53)	0.93 (0.77-1.12)	1.03 (0.85-1.24)	0.95 (0.78-1.14)	0.35
HR (95% CI), Model 2	Reference	1.20 (0.93-1.55)	0.95 (0.79-1.15)	1.07 (0.89-1.29)	1.00 (0.82-1.21)	0.39
Prostate cancer[¶]						
Events	397	90	368	280	174	
Person-years	145266	38311	117478	86364	63739	
HR (95% CI), Model 1	Reference	0.82 (0.66-1.04)	1.08 (0.94-1.25)	1.12 (0.96-1.31)	0.94 (0.79-1.13)	0.0577
HR (95% CI), Model 2	Reference	0.81 (0.65-1.02)	1.05 (0.91-1.21)	1.08 (0.93-1.27)	0.90 (0.75-1.08)	0.0717
Other cancers						
Events	848	252	779	694	605	
Person-years	229113	68801	236711	209469	190390	
HR (95% CI), Model 1	Reference	0.95 (0.83-1.10)	0.85 (0.77-0.93)	0.85 (0.77-0.94)	0.82 (0.74-0.92)	0.0011
HR (95% CI), Model 2	Reference	0.98 (0.85-1.13)	0.88 (0.79-0.97)	0.89 (0.80-0.98)	0.88 (0.79-0.98)	0.0413
Depression						
Events	78	23	65	72	44	
Person-years	234343	70078	241799	213814	194057	
HR (95% CI), Model 1	Reference	0.99 (0.62-1.57)	0.80 (0.58-1.12)	1.00 (0.72-1.39)	0.67 (0.46-0.98)	0.18
HR (95% CI), Model 2	Reference	1.04 (0.65-1.66)	0.86 (0.62-1.20)	1.09 (0.78-1.52)	0.75 (0.51-1.11)	0.31
Anxiety						
Events	498	167	530	474	391	
Person-years	241634	72266	249037	220114	199310	
HR (95% CI), Model 1	Reference	1.07 (0.90-1.28)	0.95 (0.84-1.08)	0.92 (0.81-1.05)	0.82 (0.71-0.94)	0.0168
HR (95% CI), Model 2	Reference	1.10 (0.92-1.31)	0.99 (0.88-1.13)	0.98 (0.86-1.11)	0.87 (0.76-1.00)	0.13
Schizophrenia						
Events	13	6	20	16	12	
Person-years	249060	74298	256347	226343	204797	
HR (95% CI), Model 1	Reference	1.56 (0.59-4.12)	1.52 (0.75-3.08)	1.41 (0.67-2.97)	1.21 (0.54-2.72)	0.79
HR (95% CI), Model 2	Reference	1.71 (0.65-4.52)	1.75 (0.86-3.56)	1.70 (0.80-3.61)	1.56 (0.68-3.55)	0.58
Alcohol use disorder						
Events	158	41	130	102	70	
Person-years	247727	73967	255388	225960	204576	
HR (95% CI), Model 1	Reference	0.91 (0.65-1.29)	0.88 (0.69-1.11)	0.83 (0.65-1.08)	0.68 (0.51-0.92)	0.15
HR (95% CI), Model 2	Reference	0.93 (0.66-1.32)	0.91 (0.72-1.15)	0.88 (0.68-1.14)	0.74 (0.55-0.99)	0.39
Psychoactive substance abuse						
Events	21	12	25	20	17	
Person-years	249334	74433	256748	226830	205168	
HR (95% CI), Model 1	Reference	2.04 (1.00-4.15)	1.30 (0.72-2.33)	1.26 (0.68-2.36)	1.28 (0.66-2.48)	0.42
HR (95% CI), Model 2	Reference	2.17 (1.07-4.43)	1.41 (0.78-2.53)	1.42 (0.76-2.66)	1.49 (0.76-2.92)	0.32
Epilepsy						
Events	64	17	61	51	41	

Person-years	247554	73839	254850	225383	203816	
HR (95% CI), Model 1	Reference	0.87 (0.51-1.49)	0.91 (0.64-1.29)	0.86 (0.59-1.26)	0.78 (0.52-1.17)	0.83
HR (95% CI), Model 2	Reference	0.88 (0.52-1.51)	0.92 (0.64-1.31)	0.88 (0.60-1.28)	0.80 (0.53-1.21)	0.88
Migraine						
Events	100	31	111	104	87	
Person-years	241595	72047	248046	218242	197314	
HR (95% CI), Model 1	Reference	0.99 (0.66-1.49)	1.00 (0.76-1.31)	1.02 (0.77-1.35)	0.90 (0.67-1.21)	0.94
HR (95% CI), Model 2	Reference	0.99 (0.66-1.49)	1.00 (0.76-1.32)	1.01 (0.76-1.34)	0.89 (0.65-1.20)	0.91
Dementia						
Events	142	40	158	135	106	
Person-years	249337	74350	256714	226680	205118	
HR (95% CI), Model 1	Reference	0.88 (0.62-1.25)	0.98 (0.78-1.24)	0.95 (0.74-1.20)	0.84 (0.65-1.09)	0.69
HR (95% CI), Model 2	Reference	0.89 (0.63-1.26)	1.00 (0.80-1.26)	0.97 (0.76-1.23)	0.86 (0.66-1.12)	0.74
Parkinson's disease						
Events	73	19	71	71	57	
Person-years	249108	74391	256460	226605	205053	
HR (95% CI), Model 1	Reference	0.82 (0.49-1.35)	0.87 (0.63-1.21)	0.99 (0.71-1.38)	0.89 (0.62-1.28)	0.85
HR (95% CI), Model 2	Reference	0.81 (0.49-1.34)	0.86 (0.62-1.20)	0.97 (0.69-1.36)	0.88 (0.61-1.27)	0.85
Multiple sclerosis						
Events	10	3	15	10	13	
Person-years	248786	74217	256287	226159	204535	
HR (95% CI), Model 1	Reference	0.96 (0.26-3.50)	1.34 (0.60-3.02)	0.97 (0.40-2.37)	1.33 (0.56-3.13)	0.88
HR (95% CI), Model 2	Reference	1.04 (0.28-3.78)	1.46 (0.65-3.28)	1.10 (0.45-2.70)	1.57 (0.66-3.75)	0.79
Bronchiectasis						
Events	126	28	118	95	94	
Person-years	248526	74124	255790	225951	204430	
HR (95% CI), Model 1	Reference	0.68 (0.45-1.03)	0.79 (0.62-1.02)	0.69 (0.53-0.91)	0.74 (0.56-0.98)	0.0576
HR (95% CI), Model 2	Reference	0.69 (0.46-1.04)	0.80 (0.62-1.03)	0.70 (0.53-0.91)	0.73 (0.55-0.97)	0.0704
Asthma						
Events	251	64	253	222	185	
Person-years	212702	63635	220655	195877	177973	
HR (95% CI), Model 1	Reference	0.84 (0.64-1.10)	0.94 (0.79-1.12)	0.91 (0.76-1.10)	0.83 (0.68-1.02)	0.41
HR (95% CI), Model 2	Reference	0.87 (0.66-1.14)	0.99 (0.83-1.19)	0.98 (0.81-1.18)	0.91 (0.75-1.12)	0.77
COPD						
Events	348	104	329	254	189	
Person-years	244311	73051	252322	223401	202455	
HR (95% CI), Model 1	Reference	0.94 (0.75-1.17)	0.84 (0.72-0.98)	0.72 (0.61-0.86)	0.60 (0.50-0.72)	<0.0001
HR (95% CI), Model 2	Reference	1.01 (0.81-1.25)	0.93 (0.80-1.08)	0.81 (0.69-0.96)	0.69 (0.57-0.83)	0.0009
CKD						
Events	857	255	774	619	441	
Person-years	243430	72791	251746	223026	202227	
HR (95% CI), Model 1	Reference	0.94 (0.82-1.08)	0.81 (0.74-0.90)	0.73 (0.66-0.81)	0.58 (0.52-0.65)	<0.0001
HR (95% CI), Model 2	Reference	1.00 (0.87-1.15)	0.89 (0.81-0.98)	0.82 (0.73-0.91)	0.68 (0.60-0.77)	<0.0001
Chronic liver disease						
Events	58	11	37	36	21	

Person-years	247935	74108	255688	226053	204387	
HR (95% CI), Model 1	Reference	0.62 (0.33-1.18)	0.60 (0.40-0.92)	0.67 (0.43-1.02)	0.43 (0.26-0.72)	0.0138
HR (95% CI), Model 2	Reference	0.69 (0.36-1.31)	0.71 (0.47-1.08)	0.82 (0.53-1.25)	0.58 (0.34-0.97)	0.24
Irritable bowel syndrome						
Events	196	59	198	172	132	
Person-years	241817	72249	249162	220119	199791	
HR (95% CI), Model 1	Reference	0.94 (0.70-1.26)	0.87 (0.72-1.07)	0.81 (0.66-1.00)	0.65 (0.52-0.82)	0.0057
HR (95% CI), Model 2	Reference	0.97 (0.73-1.30)	0.92 (0.75-1.13)	0.86 (0.70-1.07)	0.71 (0.56-0.89)	0.0536
Inflammatory bowel disease						
Events	79	19	62	69	55	
Person-years	245953	73587	254014	225149	203587	
HR (95% CI), Model 1	Reference	0.80 (0.49-1.33)	0.76 (0.54-1.07)	0.96 (0.69-1.34)	0.85 (0.59-1.22)	0.52
HR (95% CI), Model 2	Reference	0.81 (0.49-1.34)	0.77 (0.55-1.08)	0.98 (0.70-1.36)	0.87 (0.60-1.25)	0.56
Treated constipation						
Events	577	154	566	515	426	
Person-years	243364	72546	250588	221443	200625	
HR (95% CI), Model 1	Reference	0.86 (0.72-1.03)	0.90 (0.80-1.02)	0.92 (0.82-1.04)	0.85 (0.74-0.97)	0.12
HR (95% CI), Model 2	Reference	0.88 (0.73-1.05)	0.93 (0.82-1.04)	0.95 (0.84-1.07)	0.88 (0.77-1.00)	0.32
Dyspepsia						
Events	1508	389	1389	1252	1084	
Person-years	206692	62288	217243	193083	177206	
HR (95% CI), Model 1	Reference	0.82 (0.73-0.92)	0.83 (0.77-0.89)	0.82 (0.76-0.89)	0.77 (0.71-0.83)	<0.0001
HR (95% CI), Model 2	Reference	0.85 (0.76-0.95)	0.86 (0.80-0.93)	0.87 (0.80-0.94)	0.82 (0.76-0.90)	<0.0001
Diverticular disease						
Events	1349	347	1223	1121	858	
Person-years	229380	68855	238521	211049	193362	
HR (95% CI), Model 1	Reference	0.83 (0.74-0.93)	0.83 (0.77-0.90)	0.86 (0.79-0.93)	0.71 (0.65-0.78)	<0.0001
HR (95% CI), Model 2	Reference	0.85 (0.76-0.96)	0.87 (0.80-0.94)	0.91 (0.84-0.99)	0.77 (0.70-0.84)	<0.0001
Pernicious anaemia						
Events	30	6	22	16	11	
Person-years	248894	74291	256507	226510	204899	
HR (95% CI), Model 1	Reference	0.63 (0.26-1.52)	0.65 (0.37-1.13)	0.51 (0.28-0.96)	0.38 (0.19-0.78)	0.0693
HR (95% CI), Model 2	Reference	0.67 (0.28-1.61)	0.70 (0.40-1.22)	0.57 (0.30-1.06)	0.43 (0.21-0.89)	0.17
Fracture						
Events	118	29	138	109	123	
Person-years	247913	73874	255331	225258	203608	
HR (95% CI), Model 1	Reference	0.75 (0.50-1.13)	0.99 (0.77-1.27)	0.85 (0.65-1.11)	1.03 (0.80-1.35)	0.35
HR (95% CI), Model 2	Reference	0.74 (0.50-1.12)	0.97 (0.76-1.24)	0.82 (0.63-1.07)	0.98 (0.75-1.28)	0.36
Osteoporosis						
Events	893	293	966	829	751	
Person-years	221662	66170	227212	201279	182067	
HR (95% CI), Model 1	Reference	1.02 (0.90-1.17)	0.95 (0.87-1.04)	0.88 (0.80-0.97)	0.87 (0.79-0.96)	0.0164
HR (95% CI), Model 2	Reference	1.08 (0.94-1.23)	1.03 (0.93-1.12)	0.98 (0.89-1.08)	1.00 (0.90-1.11)	0.66
Meniere’s disease						

Events	21	2	12	10	12	
Person-years	249002	74360	256376	226361	204719	
HR (95% CI), Model 1	Reference	0.31 (0.07-1.33)	0.53 (0.26-1.08)	0.49 (0.23-1.06)	0.64 (0.30-1.35)	0.20
HR (95% CI), Model 2	Reference	0.32 (0.08-1.38)	0.57 (0.28-1.17)	0.53 (0.25-1.16)	0.73 (0.34-1.55)	0.30
Eczema						
Events	205	77	199	182	159	
Person-years	237793	71105	246038	216889	196828	
HR (95% CI), Model 1	Reference	1.22 (0.94-1.59)	0.90 (0.74-1.10)	0.93 (0.76-1.14)	0.90 (0.72-1.11)	0.18
HR (95% CI), Model 2	Reference	1.25 (0.96-1.62)	0.93 (0.76-1.13)	0.95 (0.78-1.17)	0.91 (0.73-1.13)	0.20
Glaucoma						
Events	262	77	295	265	238	
Person-years	245499	73382	252759	223000	201579	
HR (95% CI), Model 1	Reference	0.93 (0.72-1.20)	1.02 (0.87-1.21)	1.04 (0.87-1.24)	1.05 (0.88-1.26)	0.91
HR (95% CI), Model 2	Reference	0.93 (0.72-1.19)	1.02 (0.86-1.20)	1.03 (0.86-1.22)	1.03 (0.86-1.24)	0.94
Cataract						
Events	1230	361	1327	1159	1099	
Person-years	234845	70176	241680	213006	191983	
HR (95% CI), Model 1	Reference	0.89 (0.79-1.00)	0.90 (0.84-0.98)	0.86 (0.79-0.93)	0.90 (0.82-0.98)	0.0061
HR (95% CI), Model 2	Reference	0.90 (0.80-1.02)	0.92 (0.85-1.00)	0.88 (0.81-0.96)	0.93 (0.85-1.01)	0.0535
AMD						
Events	207	53	227	243	193	
Person-years	248057	74065	255301	225367	203766	
HR (95% CI), Model 1	Reference	0.78 (0.57-1.05)	0.92 (0.76-1.12)	1.08 (0.89-1.31)	0.94 (0.77-1.15)	0.18
HR (95% CI), Model 2	Reference	0.79 (0.58-1.07)	0.94 (0.78-1.14)	1.11 (0.91-1.34)	0.97 (0.79-1.19)	0.18
Thyroid disorders						
Events	331	94	340	334	295	
Person-years	234820	69849	239908	210431	189368	
HR (95% CI), Model 1	Reference	0.88 (0.70-1.10)	0.87 (0.75-1.02)	0.91 (0.78-1.06)	0.84 (0.71-0.99)	0.26
HR (95% CI), Model 2	Reference	0.91 (0.72-1.14)	0.92 (0.79-1.08)	0.98 (0.84-1.15)	0.94 (0.79-1.11)	0.80
Prostate disorders¶						
Events	700	180	549	411	305	
Person-years	135226	35524	108991	80111	59613	
HR (95% CI), Model 1	Reference	0.93 (0.79-1.09)	0.90 (0.80-1.00)	0.91 (0.80-1.02)	0.90 (0.78-1.03)	0.30
HR (95% CI), Model 2	Reference	0.93 (0.79-1.09)	0.89 (0.80-1.00)	0.90 (0.80-1.02)	0.89 (0.78-1.03)	0.28

AMD, age related macular degeneration; CI, confidence interval; CKD, chronic kidney disease; COPD, chronic obstructive pulmonary disease; HR, hazard ratio.

*Cox proportional hazard regression models were used to examine associations of the Healthful Plant-based Diet Index (quintiles) with the incidence of individual chronic diseases. The statistical tests were two-sided. Model 1 was adjusted for age and sex; Model 2 was adjusted for Model 1 plus ethnicity, education, income, smoking, alcohol consumption, sleep, physical activity, and GRS for longevity; Model 3 was adjusted for Model 2 and total energy intake.

†Cardiovascular disease includes coronary heart disease, heart failure, atrial fibrillation, other cardiac disease, stroke, and peripheral vascular disease.

[‡]All cancers encompass any type of cancer except for non-melanoma skin cancer.

[§]These analyses were conducted among women only.

[¶]These analyses were conducted among men only.

Table S9. The association between the Alternate Mediterranean Diet Index and the incidence of individual chronic diseases among individuals with 3 or more dietary assessments

	Alternate Mediterranean Diet Index					P-value
	Quintile 1	Quintile 2	Quintile 3	Quintile 4	Quintile 5	for trend*
Cardiovascular disease†						
Events	1073	988	1103	927	1263	
Person-years	96300.78	99286.92	122706.12	117017.19	161903.5	
HR (95% CI), Model 1	Reference	0.87(0.80-0.95)	0.78(0.72-0.85)	0.69(0.63-0.75)	0.68(0.63-0.74)	<0.0001
HR (95% CI), Model 2	Reference	0.90(0.83-0.99)	0.82(0.76-0.90)	0.74(0.68-0.81)	0.77(0.71-0.84)	<0.0001
Coronary heart disease						
Events	600	521	587	500	642	
Person-years	98210.29	101020.41	124600.63	118577.13	164491.35	
HR (95% CI), Model 1	Reference	0.84(0.74-0.94)	0.77(0.68-0.86)	0.70(0.62-0.79)	0.66(0.59-0.74)	<0.0001
HR (95% CI), Model 2	Reference	0.87(0.77-0.97)	0.81(0.73-0.91)	0.77(0.68-0.86)	0.76(0.68-0.85)	<0.0001
Heart failure						
Events	204	172	170	163	175	
Person-years	99647.49	102308.77	126056.92	119819.96	166125.53	
HR (95% CI), Model 1	Reference	0.80(0.65-0.98)	0.64(0.52-0.78)	0.64(0.52-0.79)	0.50(0.41-0.61)	<0.0001
HR (95% CI), Model 2	Reference	0.85(0.70-1.05)	0.70(0.57-0.86)	0.74(0.60-0.91)	0.62(0.50-0.77)	<0.0001
Atrial fibrillation						
Events	213	214	219	192	257	
Person-years	98963.74	101557.07	125226.41	119058.92	164963.44	
HR (95% CI), Model 1	Reference	0.96(0.80-1.16)	0.80(0.66-0.97)	0.75(0.61-0.91)	0.73(0.61-0.88)	0.0010
HR (95% CI), Model 2	Reference	1.00(0.83-1.21)	0.84(0.70-1.02)	0.81(0.66-0.99)	0.83(0.69-1.01)	0.0691
Other cardiac disease						
Events	452	420	427	356	520	
Person-years	99173.11	101789.8	125392.84	119423.41	165484.4	
HR (95% CI), Model 1	Reference	0.89(0.78-1.02)	0.73(0.64-0.83)	0.63(0.55-0.73)	0.67(0.59-0.76)	<0.0001
HR (95% CI), Model 2	Reference	0.92(0.80-1.05)	0.77(0.68-0.88)	0.69(0.60-0.79)	0.76(0.67-0.87)	<0.0001
Stroke						
Events	96	83	95	81	87	
Person-years	99491.66	102140.04	125910.84	119714.21	165988.42	
HR (95% CI), Model 1	Reference	0.83(0.62-1.11)	0.77(0.58-1.02)	0.69(0.51-0.92)	0.53(0.40-0.71)	0.0007
HR (95% CI), Model 2	Reference	0.85(0.63-1.14)	0.79(0.60-1.06)	0.72(0.53-0.97)	0.57(0.42-0.77)	0.0055
Peripheral vascular disease						
Events	105	104	112	80	122	
Person-years	99558.21	102199.82	125987.12	119757.91	165932.93	
HR (95% CI), Model 1	Reference	0.95(0.72-1.25)	0.82(0.63-1.07)	0.61(0.46-0.82)	0.67(0.52-0.88)	0.0017
HR (95% CI), Model 2	Reference	1.01(0.77-1.32)	0.89(0.68-1.16)	0.67(0.50-0.91)	0.76(0.58-1.00)	0.0276
Hypertension						
Events	506	386	485	405	551	
Person-years	75081.5	77072.52	96948.12	94197.29	132371.73	
HR (95% CI), Model 1	Reference	0.73(0.64-0.84)	0.72(0.63-0.81)	0.62(0.54-0.70)	0.59(0.52-0.67)	<0.0001
HR (95% CI), Model 2	Reference	0.77(0.67-0.88)	0.78(0.69-0.88)	0.69(0.61-0.79)	0.72(0.64-0.82)	<0.0001

Diabetes						
Events	313	313	300	246	302	
Person-years	95715.44	98378.37	122204.43	116312.4	162278.07	
HR (95% CI), Model 1	Reference	0.97(0.83-1.13)	0.75(0.64-0.88)	0.65(0.55-0.77)	0.57(0.49-0.67)	<0.0001
HR (95% CI), Model 2	Reference	1.11(0.95-1.30)	0.89(0.76-1.05)	0.84(0.71-1.00)	0.86(0.73-1.02)	0.0041
All cancers‡						
Events	724	714	764	667	922	
Person-years	93092.95	95613.82	118362.56	111809.26	155639.36	
HR (95% CI), Model 1	Reference	0.94(0.85-1.04)	0.79(0.72-0.88)	0.72(0.65-0.80)	0.70(0.64-0.78)	<0.0001
HR (95% CI), Model 2	Reference	0.97(0.87-1.07)	0.83(0.75-0.92)	0.76(0.69-0.85)	0.77(0.70-0.85)	<0.0001
Non-melanoma skin cancer						
Events	366	365	448	435	605	
Person-years	98505.79	101036.38	124417.01	118208.71	163817.43	
HR (95% CI), Model 1	Reference	0.96(0.83-1.11)	0.95(0.83-1.09)	0.97(0.84-1.12)	0.97(0.85-1.11)	0.96
HR (95% CI), Model 2	Reference	0.94(0.81-1.08)	0.93(0.81-1.07)	0.94(0.81-1.08)	0.92(0.80-1.05)	0.80
Melanoma						
Events	46	54	62	51	82	
Person-years	99149.78	101464.39	125067.25	118519.64	164768.18	
HR (95% CI), Model 1	Reference	1.14(0.77-1.68)	1.04(0.71-1.53)	0.90(0.60-1.35)	1.03(0.72-1.49)	0.83
HR (95% CI), Model 2	Reference	1.13(0.76-1.67)	1.03(0.71-1.52)	0.90(0.60-1.34)	1.03(0.71-1.49)	0.84
Lung cancer						
Events	101	82	58	53	81	
Person-years	99751.03	102423.85	126225.27	119912.35	166287.64	
HR (95% CI), Model 1	Reference	0.77(0.57-1.03)	0.43(0.31-0.59)	0.40(0.29-0.56)	0.43(0.32-0.58)	<0.0001
HR (95% CI), Model 2	Reference	0.93(0.70-1.23)	0.62(0.46-0.84)	0.59(0.43-0.81)	0.71(0.53-0.95)	0.0014
Stomach cancer						
Events	19	21	24	14	20	
Person-years	99763.35	102414.06	126225.4	119939.01	166254.37	
HR (95% CI), Model 1	Reference	1.09(0.58-2.02)	1.04(0.57-1.90)	0.66(0.33-1.32)	0.71(0.38-1.34)	0.42
HR (95% CI), Model 2	Reference	1.12(0.60-2.08)	1.08(0.59-1.98)	0.70(0.35-1.41)	0.79(0.41-1.50)	0.58
Oesophageal cancer						
Events	30	24	22	19	25	
Person-years	99763.53	102433.51	126209.43	119965.29	166323.06	
HR (95% CI), Model 1	Reference	0.77(0.45-1.32)	0.59(0.34-1.02)	0.54(0.30-0.97)	0.53(0.31-0.91)	0.11
HR (95% CI), Model 2	Reference	0.83(0.49-1.43)	0.65(0.37-1.13)	0.63(0.35-1.13)	0.68(0.39-1.18)	0.44
Colon cancer						
Events	98	95	91	92	127	
Person-years	99223.05	101997.74	125831.18	119479.93	165743.08	
HR (95% CI), Model 1	Reference	0.92(0.70-1.22)	0.71(0.53-0.94)	0.75(0.56-1.00)	0.74(0.57-0.97)	0.0661
HR (95% CI), Model 2	Reference	0.96(0.72-1.27)	0.75(0.56-1.00)	0.81(0.61-1.08)	0.84(0.64-1.10)	0.25
Ovarian cancer§						
Events	17	29	31	26	34	
Person-years	50190.78	55278.12	73544.72	74154.14	111168.04	
HR (95% CI), Model 1	Reference	1.51(0.83-2.75)	1.18(0.65-2.14)	0.96(0.52-1.77)	0.80(0.45-1.44)	0.14

HR (95% CI), Model 2	Reference	1.54(0.84-2.80)	1.23(0.68-2.22)	1.01(0.55-1.87)	0.87(0.48-1.59)	0.23
Breast cancer[§]						
Events	178	179	229	235	349	
Person-years	47813	52776.42	70343.12	71061.95	105799.12	
HR (95% CI), Model 1	Reference	0.91(0.74-1.11)	0.86(0.71-1.05)	0.87(0.71-1.05)	0.85(0.71-1.02)	0.48
HR (95% CI), Model 2	Reference	0.92(0.75-1.14)	0.89(0.73-1.08)	0.90(0.74-1.10)	0.90(0.75-1.08)	0.79
Prostate cancer[¶]						
Events	233	239	278	233	291	
Person-years	54724.58	51444.9	58005.55	50454.99	60537.67	
HR (95% CI), Model 1	Reference	1.11(0.96-1.27)	1.07(0.93-1.22)	1.13(0.99-1.30)	1.06(0.92-1.22)	0.46
HR (95% CI), Model 2	Reference	1.08(0.95-1.24)	1.04(0.91-1.19)	1.10(0.95-1.26)	1.01(0.87-1.16)	0.58
Other cancers						
Events	643	593	656	581	769	
Person-years	94223.91	96942.99	119927.29	113515.15	157569.41	
HR (95% CI), Model 1	Reference	0.88(0.78-0.98)	0.77(0.69-0.86)	0.71(0.63-0.79)	0.66(0.60-0.74)	<0.0001
HR (95% CI), Model 2	Reference	0.90(0.80-1.00)	0.80(0.72-0.89)	0.75(0.67-0.84)	0.73(0.65-0.81)	<0.0001
Depression						
Events	114	102	101	99	125	
Person-years	93898.39	97048.67	119694.95	113987.36	159128.18	
HR (95% CI), Model 1	Reference	0.85(0.65-1.11)	0.67(0.51-0.87)	0.68(0.52-0.89)	0.60(0.46-0.77)	0.0007
HR (95% CI), Model 2	Reference	0.90(0.68-1.17)	0.73(0.56-0.96)	0.77(0.58-1.01)	0.72(0.55-0.94)	0.0746
Anxiety						
Events	347	293	369	302	416	
Person-years	97668.66	100434.74	123643.17	117806.57	163398.8	
HR (95% CI), Model 1	Reference	0.79(0.68-0.92)	0.78(0.67-0.90)	0.65(0.56-0.76)	0.61(0.53-0.71)	<0.0001
HR (95% CI), Model 2	Reference	0.81(0.69-0.95)	0.82(0.71-0.95)	0.70(0.60-0.82)	0.69(0.59-0.80)	<0.0001
Schizophrenia						
Events	13	13	13	16	16	
Person-years	99517.35	102158.23	125825.92	119533.52	165980.91	
HR (95% CI), Model 1	Reference	0.97(0.45-2.08)	0.78(0.36-1.69)	1.00(0.48-2.10)	0.71(0.34-1.50)	0.83
HR (95% CI), Model 2	Reference	1.10(0.51-2.38)	0.95(0.44-2.07)	1.31(0.62-2.75)	1.07(0.50-2.28)	0.93
Alcohol use disorder						
Events	127	91	90	78	66	
Person-years	99281.12	101993.52	125717.99	119607.2	166115.01	
HR (95% CI), Model 1	Reference	0.71(0.54-0.93)	0.58(0.44-0.76)	0.54(0.41-0.72)	0.34(0.25-0.46)	<0.0001
HR (95% CI), Model 2	Reference	0.74(0.56-0.97)	0.63(0.48-0.83)	0.61(0.46-0.81)	0.39(0.29-0.54)	<0.0001
Psychoactive substance abuse						
Events	22	15	20	9	16	
Person-years	99695.47	102270.28	126103.07	119868.65	166229.16	
HR (95% CI), Model 1	Reference	0.67(0.35-1.29)	0.74(0.40-1.35)	0.35(0.16-0.77)	0.46(0.24-0.88)	0.0502
HR (95% CI), Model 2	Reference	0.75(0.39-1.44)	0.87(0.47-1.60)	0.44(0.20-0.96)	0.61(0.31-1.20)	0.26
Epilepsy						
Events	44	35	41	45	39	
Person-years	98877.75	101740.59	125249.25	119299.79	165358.93	

HR (95% CI), Model 1	Reference	0.76(0.49-1.19)	0.72(0.47-1.10)	0.83(0.54-1.26)	0.51(0.33-0.79)	0.05
HR (95% CI), Model 2	Reference	0.77(0.50-1.21)	0.74(0.48-1.13)	0.85(0.56-1.30)	0.54(0.35-0.84)	0.09
Migraine						
Events	65	69	77	68	115	
Person-years	96696.67	99206.06	121967.35	116101.2	160443.34	
HR (95% CI), Model 1	Reference	1.02(0.73-1.43)	0.91(0.65-1.26)	0.83(0.59-1.17)	1.00(0.73-1.36)	0.70
HR (95% CI), Model 2	Reference	1.03(0.73-1.44)	0.92(0.66-1.28)	0.84(0.60-1.19)	1.02(0.74-1.39)	0.70
Dementia						
Events	83	64	71	74	109	
Person-years	99778.11	102378.99	126216.86	119949.62	166323.82	
HR (95% CI), Model 1	Reference	0.72(0.52-1.00)	0.64(0.46-0.87)	0.68(0.50-0.93)	0.71(0.53-0.95)	0.0463
HR (95% CI), Model 2	Reference	0.74(0.53-1.02)	0.66(0.48-0.91)	0.71(0.52-0.98)	0.76(0.56-1.02)	0.10
Parkinson’s disease						
Events	49	38	55	52	40	
Person-years	99718.17	102336.31	126061.26	119799.22	166181.31	
HR (95% CI), Model 1	Reference	0.74(0.49-1.13)	0.87(0.59-1.28)	0.86(0.58-1.28)	0.48(0.32-0.74)	0.0106
HR (95% CI), Model 2	Reference	0.73(0.48-1.12)	0.86(0.58-1.27)	0.85(0.57-1.27)	0.47(0.31-0.73)	0.0092
Multiple sclerosis						
Events	11	11	14	12	13	
Person-years	99424.48	102122.46	125687.96	119555.53	165640.47	
HR (95% CI), Model 1	Reference	0.97(0.42-2.24)	1.00(0.45-2.21)	0.90(0.39-2.04)	0.70(0.31-1.58)	0.89
HR (95% CI), Model 2	Reference	1.00(0.43-2.31)	1.04(0.47-2.30)	0.94(0.41-2.16)	0.75(0.33-1.72)	0.93
Bronchiectasis						
Events	77	74	67	61	101	
Person-years	99535.01	102131.94	125801.76	119678.26	165829.73	
HR (95% CI), Model 1	Reference	0.90(0.66-1.24)	0.64(0.46-0.89)	0.59(0.42-0.83)	0.68(0.50-0.92)	0.0053
HR (95% CI), Model 2	Reference	0.92(0.67-1.27)	0.66(0.47-0.92)	0.61(0.43-0.86)	0.70(0.51-0.95)	0.0121
Asthma						
Events	193	174	208	198	240	
Person-years	87364.53	90170.57	111022.95	105587.51	148335.85	
HR (95% CI), Model 1	Reference	0.85(0.69-1.04)	0.81(0.66-0.98)	0.79(0.65-0.97)	0.66(0.55-0.81)	0.0013
HR (95% CI), Model 2	Reference	0.88(0.72-1.08)	0.86(0.71-1.05)	0.87(0.71-1.07)	0.77(0.63-0.94)	0.16
COPD						
Events	278	198	222	164	211	
Person-years	98341.24	101178.77	124948.83	118833.19	164926.39	
HR (95% CI), Model 1	Reference	0.67(0.56-0.81)	0.60(0.50-0.71)	0.46(0.38-0.55)	0.42(0.35-0.50)	<0.0001
HR (95% CI), Model 2	Reference	0.76(0.63-0.91)	0.71(0.59-0.85)	0.57(0.47-0.69)	0.58(0.48-0.69)	<0.0001
CKD						
Events	582	472	513	420	504	
Person-years	99172.59	101834.18	125695.77	119503.82	165623.47	
HR (95% CI), Model 1	Reference	0.76(0.67-0.86)	0.66(0.59-0.74)	0.56(0.49-0.64)	0.48(0.43-0.54)	<0.0001
HR (95% CI), Model 2	Reference	0.80(0.71-0.91)	0.73(0.65-0.82)	0.64(0.56-0.73)	0.59(0.52-0.67)	<0.0001
Chronic liver disease						
Events	29	23	28	24	19	
Person-years	99131.74	101991.46	125753.24	119229	165599.47	

HR (95% CI), Model 1	Reference	0.77(0.45-1.33)	0.76(0.45-1.29)	0.70(0.40-1.20)	0.40(0.22-0.72)	0.0460
HR (95% CI), Model 2	Reference	0.78(0.48-1.27)	0.65(0.40-1.06)	0.77(0.47-1.24)	0.38(0.22-0.67)	0.0176
Irritable bowel syndrome						
Events	139	107	126	126	160	
Person-years	97002.76	99727.63	123112.56	116826.31	162419.99	
HR (95% CI), Model 1	Reference	0.72(0.56-0.92)	0.66(0.51-0.83)	0.67(0.52-0.85)	0.58(0.46-0.73)	<0.0001
HR (95% CI), Model 2	Reference	0.74(0.58-0.96)	0.71(0.56-0.91)	0.75(0.58-0.95)	0.68(0.54-0.86)	0.0151
Inflammatory bowel disease						
Events	48	43	60	50	67	
Person-years	98385.44	101396.36	125081.2	118827.3	165047.56	
HR (95% CI), Model 1	Reference	0.86(0.57-1.30)	0.98(0.67-1.43)	0.85(0.57-1.27)	0.82(0.56-1.19)	0.79
HR (95% CI), Model 2	Reference	0.90(0.60-1.36)	1.04(0.71-1.52)	0.93(0.62-1.40)	0.93(0.64-1.37)	0.95
Treated constipation						
Events	404	349	431	347	510	
Person-years	98609.87	101220.14	124878.66	118896.88	164715.8	
HR (95% CI), Model 1	Reference	0.82(0.71-0.95)	0.80(0.70-0.92)	0.67(0.58-0.77)	0.69(0.61-0.79)	<0.0001
HR (95% CI), Model 2	Reference	0.83(0.72-0.96)	0.82(0.71-0.94)	0.69(0.59-0.79)	0.72(0.63-0.83)	<0.0001
Dyspepsia						
Events	1081	1032	1219	1121	1478	
Person-years	87029.08	91126.99	112654.21	108041.5	151994.09	
HR (95% CI), Model 1	Reference	0.89(0.82-0.97)	0.83(0.77-0.91)	0.79(0.72-0.86)	0.72(0.67-0.78)	<0.0001
HR (95% CI), Model 2	Reference	0.92(0.84-1.00)	0.87(0.80-0.95)	0.84(0.77-0.92)	0.80(0.74-0.87)	<0.0001
Diverticular disease						
Events	947	879	1074	868	1172	
Person-years	95503.29	98771.67	121566.34	116249.27	161603.57	
HR (95% CI), Model 1	Reference	0.88(0.80-0.96)	0.86(0.79-0.94)	0.71(0.65-0.78)	0.68(0.62-0.74)	<0.0001
HR (95% CI), Model 2	Reference	0.91(0.83-1.00)	0.91(0.83-0.99)	0.78(0.71-0.85)	0.78(0.71-0.85)	<0.0001
Pernicious anaemia						
Events	29	18	11	15	18	
Person-years	99479.26	102101.3	125942.5	119658.51	165938.52	
HR (95% CI), Model 1	Reference	0.60(0.33-1.07)	0.29(0.14-0.58)	0.41(0.22-0.77)	0.35(0.19-0.64)	0.0007
HR (95% CI), Model 2	Reference	0.51(0.24-1.09)	0.47(0.22-0.97)	0.36(0.16-0.82)	0.38(0.18-0.79)	0.0365
Fracture						
Events	67	70	86	87	144	
Person-years	99248.67	101785.71	125656.9	119401.31	165523.36	
HR (95% CI), Model 1	Reference	0.98(0.70-1.37)	0.94(0.68-1.29)	0.97(0.70-1.33)	1.11(0.83-1.49)	0.75
HR (95% CI), Model 2	Reference	0.98(0.70-1.38)	0.93(0.67-1.28)	0.95(0.69-1.31)	1.05(0.78-1.42)	0.90
Osteoporosis						
Events	648	649	772	697	923	
Person-years	90754.38	94129.7	115872.55	110061.94	152974.71	
HR (95% CI), Model 1	Reference	0.93(0.83-1.04)	0.86(0.78-0.96)	0.80(0.72-0.89)	0.74(0.66-0.81)	<0.0001
HR (95% CI), Model 2	Reference	0.96(0.86-1.07)	0.93(0.84-1.03)	0.89(0.80-0.99)	0.87(0.79-0.97)	0.0870
Meniere's disease						
Events	9	10	11	12	16	

Person-years	99562.36	102176.18	125693.17	119688.29	165742.56	
HR (95% CI), Model 1	Reference	1.07(0.43-2.63)	0.95(0.39-2.29)	1.08(0.45-2.57)	1.03(0.45-2.35)	1.00
HR (95% CI), Model 2	Reference	1.08(0.44-2.67)	0.98(0.41-2.38)	1.16(0.48-2.77)	1.17(0.50-2.70)	0.99
Eczema						
Events	144	119	154	123	165	
Person-years	95431.22	98018.44	121412.05	115022.22	159742.91	
HR (95% CI), Model 1	Reference	0.80(0.62-1.02)	0.83(0.66-1.04)	0.70(0.55-0.89)	0.67(0.53-0.84)	0.0060
HR (95% CI), Model 2	Reference	0.81(0.63-1.03)	0.85(0.67-1.07)	0.72(0.56-0.92)	0.70(0.55-0.88)	0.0247
Glaucoma						
Events	162	161	190	172	263	
Person-years	98572.12	101025.9	124735.81	118475.58	164382.96	
HR (95% CI), Model 1	Reference	0.95(0.77-1.18)	0.89(0.72-1.10)	0.85(0.68-1.05)	0.93(0.76-1.13)	0.62
HR (95% CI), Model 2	Reference	0.94(0.76-1.17)	0.88(0.71-1.09)	0.83(0.67-1.03)	0.89(0.72-1.09)	0.51
Cataract						
Events	792	730	886	924	1242	
Person-years	96588.76	99368.36	122414.29	116253.16	160969.39	
HR (95% CI), Model 1	Reference	0.86(0.78-0.95)	0.81(0.73-0.89)	0.87(0.79-0.96)	0.81(0.74-0.89)	<0.0001
HR (95% CI), Model 2	Reference	0.87(0.79-0.96)	0.83(0.75-0.91)	0.89(0.81-0.98)	0.85(0.77-0.93)	0.0012
AMD						
Events	127	137	142	144	217	
Person-years	99603.82	102149.88	125953.08	119630.99	165847.47	
HR (95% CI), Model 1	Reference	1.01(0.79-1.29)	0.81(0.64-1.03)	0.83(0.66-1.06)	0.87(0.69-1.08)	0.21
HR (95% CI), Model 2	Reference	1.02(0.80-1.30)	0.83(0.65-1.05)	0.85(0.67-1.08)	0.89(0.71-1.11)	0.31
Thyroid disorders						
Events	234	201	261	253	350	
Person-years	93657.97	96650.01	118734.56	113031.07	156270.13	
HR (95% CI), Model 1	Reference	0.80(0.66-0.96)	0.81(0.68-0.96)	0.80(0.67-0.95)	0.76(0.64-0.90)	0.0220
HR (95% CI), Model 2	Reference	0.81(0.67-0.98)	0.84(0.71-1.01)	0.85(0.71-1.02)	0.85(0.72-1.01)	0.22
Prostate disorders¶						
Events	443	385	424	357	437	
Person-years	51594.84	48389.38	54474.95	47551.91	57027.33	
HR (95% CI), Model 1	Reference	0.90(0.81-1.00)	0.87(0.78-0.96)	0.89(0.80-0.99)	0.85(0.77-0.95)	0.0253
HR (95% CI), Model 2	Reference	0.90(0.81-1.00)	0.87(0.79-0.97)	0.89(0.80-1.00)	0.86(0.77-0.96)	0.0463

AMD, age related macular degeneration; CI, confidence interval; CKD, chronic kidney disease; COPD, chronic obstructive pulmonary disease; HR, hazard ratio.

*Cox proportional hazard regression models were used to examine associations of the Alternate Mediterranean Diet Index (quintiles) with the incidence of individual chronic diseases. The statistical tests were two-sided. Model 1 was adjusted for age and sex; Model 2 was adjusted for Model 1 plus ethnicity, education, income, smoking, alcohol consumption, sleep, physical activity, and GRS for longevity; Model 3 was adjusted for Model 2 and total energy intake.

†Cardiovascular disease includes coronary heart disease, heart failure, atrial fibrillation, other cardiac disease, stroke, and peripheral vascular disease.

‡All cancers encompass any type of cancer except for non-melanoma skin cancer.

[§]These analyses were conducted among women only.

[¶]These analyses were conducted among men only.

Table S10. The association between the Anti-Empirical Dietary Inflammatory Index and the incidence of individual chronic diseases among individuals with three or more dietary assessments

	Anti-Empirical Dietary Inflammatory Index					P-value
	Quintile 1	Quintile 2	Quintile 3	Quintile 4	Quintile 5	for trend*
Cardiovascular disease†						
Events	1102	1061	1069	1012	1110	
Person-years	115128.51	116527.68	116788.33	116721.3	117150.09	
HR (95% CI), Model 1	Reference	0.89(0.81-0.96)	0.85(0.78-0.92)	0.77(0.71-0.84)	0.81(0.74-0.88)	<0.0001
HR (95% CI), Model 2	Reference	0.94(0.86-1.02)	0.91(0.83-0.99)	0.85(0.78-0.92)	0.89(0.82-0.97)	0.0037
Coronary heart disease						
Events	629	566	557	521	577	
Person-years	119471.59	120657.02	120956.85	120917.94	121375.43	
HR (95% CI), Model 1	Reference	0.83(0.74-0.93)	0.77(0.69-0.87)	0.70(0.62-0.78)	0.74(0.66-0.83)	<0.0001
HR (95% CI), Model 2	Reference	0.89(0.80-1.00)	0.85(0.76-0.95)	0.78(0.70-0.88)	0.84(0.75-0.95)	0.0012
Heart failure						
Events	193	178	181	167	165	
Person-years	122856.76	123881.26	123895.27	123722.49	124573.91	
HR (95% CI), Model 1	Reference	0.84(0.68-1.03)	0.80(0.65-0.98)	0.71(0.57-0.87)	0.66(0.54-0.81)	0.0010
HR (95% CI), Model 2	Reference	0.93(0.76-1.14)	0.91(0.74-1.12)	0.84(0.68-1.03)	0.80(0.65-0.99)	0.27
Atrial fibrillation						
Events	218	205	209	237	226	
Person-years	121289.45	122433.8	122289.12	122005.14	122944.27	
HR (95% CI), Model 1	Reference	0.86(0.71-1.04)	0.82(0.68-0.99)	0.89(0.74-1.07)	0.80(0.66-0.97)	0.16
HR (95% CI), Model 2	Reference	0.92(0.76-1.12)	0.90(0.74-1.09)	1.00(0.83-1.21)	0.91(0.75-1.10)	0.65
Other cardiac disease						
Events	432	428	462	430	423	
Person-years	121318.9	122518.31	122546.05	122504.63	123270.19	
HR (95% CI), Model 1	Reference	0.90(0.79-1.03)	0.93(0.81-1.06)	0.83(0.72-0.94)	0.78(0.68-0.89)	0.0021
HR (95% CI), Model 2	Reference	0.96(0.84-1.10)	0.99(0.87-1.14)	0.92(0.80-1.05)	0.87(0.76-0.99)	0.20
Stroke						
Events	83	94	84	74	107	
Person-years	122855.39	123758.14	123817.59	123829.76	124315.78	
HR (95% CI), Model 1	Reference	1.04(0.77-1.39)	0.88(0.65-1.19)	0.74(0.54-1.02)	1.03(0.77-1.37)	0.16
HR (95% CI), Model 2	Reference	1.07(0.80-1.44)	0.91(0.67-1.23)	0.78(0.56-1.07)	1.07(0.80-1.44)	0.19
Peripheral vascular disease						
Events	116	89	107	106	105	
Person-years	122899.8	124056.8	123946.83	123922.22	124597.79	
HR (95% CI), Model 1	Reference	0.72(0.54-0.94)	0.83(0.64-1.09)	0.80(0.62-1.04)	0.77(0.59-1.00)	0.16
HR (95% CI), Model 2	Reference	0.73(0.55-0.96)	0.85(0.65-1.10)	0.82(0.63-1.07)	0.77(0.59-1.02)	0.21
Hypertension						
Events	490	475	449	446	473	
Person-years	88618.5	92026.95	92921.67	94041.56	94681.98	
HR (95% CI), Model 1	Reference	0.85(0.75-0.97)	0.76(0.67-0.86)	0.71(0.63-0.81)	0.73(0.64-0.82)	<0.0001
HR (95% CI), Model 2	Reference	0.94(0.83-1.06)	0.85(0.75-0.97)	0.82(0.72-0.93)	0.86(0.75-0.98)	0.0193

Diabetes						
Events	444	303	261	248	218	
Person-years	116163.47	118817.26	119779.16	120232.02	121585.98	
HR (95% CI), Model 1	Reference	0.64(0.55-0.74)	0.53(0.45-0.62)	0.49(0.42-0.57)	0.41(0.35-0.49)	<0.0001
HR (95% CI), Model 2	Reference	0.79(0.68-0.91)	0.67(0.58-0.79)	0.67(0.57-0.79)	0.60(0.50-0.71)	<0.0001
All cancers‡						
Events	697	727	798	787	782	
Person-years	112661.08	113330.21	112959.08	112961.82	113567.11	
HR (95% CI), Model 1	Reference	0.97(0.87-1.07)	1.03(0.93-1.14)	0.98(0.88-1.08)	0.94(0.85-1.04)	0.48
HR (95% CI), Model 2	Reference	1.00(0.90-1.11)	1.06(0.96-1.18)	1.02(0.92-1.13)	0.98(0.88-1.09)	0.51
Non-melanoma skin cancer						
Events	393	407	437	495	487	
Person-years	120545.92	121161.93	121338.36	120699.1	121417.59	
HR (95% CI), Model 1	Reference	0.96(0.83-1.10)	0.98(0.85-1.12)	1.07(0.94-1.22)	1.01(0.88-1.15)	0.49
HR (95% CI), Model 2	Reference	0.94(0.82-1.08)	0.96(0.84-1.10)	1.04(0.91-1.19)	0.99(0.87-1.14)	0.58
Melanoma						
Events	43	66	67	59	60	
Person-years	122504.73	123170.91	123125.62	123082.01	123704.47	
HR (95% CI), Model 1	Reference	1.44(0.98-2.12)	1.42(0.97-2.09)	1.22(0.82-1.81)	1.21(0.82-1.79)	0.33
HR (95% CI), Model 2	Reference	1.42(0.97-2.09)	1.40(0.95-2.05)	1.19(0.80-1.77)	1.19(0.80-1.78)	0.37
Lung cancer						
Events	70	55	80	78	92	
Person-years	123346.23	124322.32	124276.18	124194.06	124819.32	
HR (95% CI), Model 1	Reference	0.71(0.50-1.01)	0.99(0.72-1.37)	0.93(0.67-1.28)	1.06(0.77-1.44)	0.20
HR (95% CI), Model 2	Reference	0.78(0.56-1.08)	1.06(0.78-1.44)	1.02(0.75-1.39)	1.02(0.76-1.38)	0.37
Stomach cancer						
Events	16	24	18	25	15	
Person-years	123592.44	124500.87	124495.62	124345.23	125198.66	
HR (95% CI), Model 1	Reference	1.41(0.75-2.65)	1.00(0.51-1.97)	1.35(0.72-2.54)	0.77(0.38-1.56)	0.32
HR (95% CI), Model 2	Reference	1.47(0.78-2.77)	1.07(0.54-2.11)	1.43(0.76-2.71)	0.81(0.40-1.67)	0.31
Oesophageal cancer						
Events	20	28	27	18	27	
Person-years	123550.66	124507.72	124466.31	124479.06	125129.56	
HR (95% CI), Model 1	Reference	1.27(0.72-2.26)	1.15(0.65-2.06)	0.74(0.39-1.39)	1.04(0.58-1.87)	0.47
HR (95% CI), Model 2	Reference	1.42(0.80-2.53)	1.32(0.74-2.36)	0.88(0.46-1.69)	1.27(0.70-2.29)	0.49
Colon cancer						
Events	96	93	93	99	122	
Person-years	122680.71	123835.37	123641.28	123626.38	124252	
HR (95% CI), Model 1	Reference	0.89(0.67-1.18)	0.85(0.64-1.13)	0.87(0.65-1.15)	1.03(0.78-1.34)	0.55
HR (95% CI), Model 2	Reference	0.93(0.70-1.24)	0.90(0.67-1.20)	0.94(0.70-1.25)	1.10(0.84-1.45)	0.60
Ovarian cancer§						
Events	33	36	20	22	26	
Person-years	72745.17	74726.61	73872.14	73732.71	72424.54	
HR (95% CI), Model 1	Reference	1.04(0.65-1.67)	0.57(0.33-1.00)	0.62(0.36-1.06)	0.73(0.43-1.21)	0.0898

HR (95% CI), Model 2	Reference	1.09(0.68-1.76)	0.62(0.35-1.08)	0.68(0.40-1.18)	0.83(0.49-1.42)	0.20
Breast cancer[§]						
Events	228	247	233	244	218	
Person-years	68991.75	70254.35	69304.38	69288.89	67884.71	
HR (95% CI), Model 1	Reference	1.05(0.88-1.26)	1.00(0.83-1.20)	1.04(0.87-1.24)	0.94(0.78-1.13)	0.78
HR (95% CI), Model 2	Reference	1.07(0.89-1.28)	1.01(0.84-1.21)	1.05(0.87-1.26)	0.95(0.78-1.15)	0.75
Prostate cancer[¶]						
Events	212	242	292	252	277	
Person-years	56302.25	53852.5	54671.58	54735.1	55884.5	
HR (95% CI), Model 1	Reference	1.10(0.95-1.27)	1.21(1.05-1.39)	1.08(0.94-1.25)	1.11(0.96-1.28)	0.12
HR (95% CI), Model 2	Reference	1.07(0.93-1.24)	1.17(1.02-1.35)	1.04(0.90-1.20)	1.07(0.93-1.23)	0.24
Other cancers						
Events	598	625	692	659	668	
Person-years	114496.86	115335.04	115147.03	115102.83	115684.21	
HR (95% CI), Model 1	Reference	0.97(0.87-1.09)	1.04(0.93-1.16)	0.96(0.86-1.07)	0.94(0.84-1.05)	0.37
HR (95% CI), Model 2	Reference	1.01(0.90-1.13)	1.08(0.97-1.21)	1.01(0.90-1.13)	0.99(0.88-1.10)	0.46
Depression						
Events	137	116	94	104	90	
Person-years	115763.98	117221.03	118064.99	118098.64	119056.35	
HR (95% CI), Model 1	Reference	0.83(0.65-1.06)	0.67(0.51-0.87)	0.74(0.57-0.95)	0.64(0.49-0.83)	0.0051
HR (95% CI), Model 2	Reference	0.90(0.70-1.15)	0.73(0.56-0.96)	0.83(0.64-1.08)	0.73(0.55-0.96)	0.0963
Anxiety						
Events	423	332	327	338	307	
Person-years	119268.61	121157.36	121051.78	121293.79	122149.22	
HR (95% CI), Model 1	Reference	0.76(0.65-0.87)	0.74(0.64-0.86)	0.75(0.65-0.87)	0.68(0.59-0.79)	<0.0001
HR (95% CI), Model 2	Reference	0.79(0.69-0.92)	0.78(0.68-0.91)	0.81(0.70-0.93)	0.73(0.63-0.85)	0.0004
Schizophrenia						
Events	12	22	12	8	17	
Person-years	123219.6	124154.22	124236.13	124227.23	124860.14	
HR (95% CI), Model 1	Reference	1.83(0.91-3.70)	1.00(0.45-2.22)	0.66(0.27-1.62)	1.39(0.66-2.93)	0.0965
HR (95% CI), Model 2	Reference	2.10(1.03-4.26)	1.18(0.53-2.65)	0.81(0.33-2.01)	1.77(0.82-3.78)	0.0747
Alcohol use disorder						
Events	54	79	86	98	135	
Person-years	122945.01	123876.21	123931.87	123854.43	124334.31	
HR (95% CI), Model 1	Reference	1.45(1.03-2.05)	1.55(1.10-2.17)	1.74(1.25-2.43)	2.34(1.70-3.21)	<0.0001
HR (95% CI), Model 2	Reference	1.50(1.06-2.12)	1.59(1.13-2.25)	1.79(1.28-2.51)	2.36(1.71-3.26)	0.0001
Psychoactive substance abuse						
Events	7	17	21	20	17	
Person-years	123528.84	124403.45	124410.59	124355.04	125121.68	
HR (95% CI), Model 1	Reference	2.47(1.02-5.97)	3.03(1.29-7.15)	2.88(1.22-6.84)	2.41(1.00-5.84)	0.13
HR (95% CI), Model 2	Reference	2.72(1.12-6.58)	3.34(1.41-7.90)	3.24(1.36-7.72)	2.61(1.07-6.40)	0.0787
Epilepsy						
Events	51	37	42	35	39	
Person-years	122311.65	123627.82	123645.82	123638.29	124408.41	

HR (95% CI), Model 1	Reference	0.69(0.45-1.05)	0.76(0.51-1.15)	0.62(0.40-0.95)	0.67(0.44-1.02)	0.19
HR (95% CI), Model 2	Reference	0.69(0.45-1.06)	0.77(0.51-1.16)	0.62(0.40-0.95)	0.66(0.43-1.02)	0.19
Migraine						
Events	87	63	81	98	65	
Person-years	117939.2	119735.57	120522.78	120692.53	121813.71	
HR (95% CI), Model 1	Reference	0.70(0.51-0.97)	0.90(0.66-1.22)	1.09(0.81-1.45)	0.72(0.52-1.00)	0.0205
HR (95% CI), Model 2	Reference	0.71(0.51-0.98)	0.90(0.66-1.22)	1.09(0.81-1.47)	0.72(0.51-1.00)	0.0205
Dementia						
Events	69	85	76	80	91	
Person-years	123511.02	124408.68	124409.34	124358.35	125029.24	
HR (95% CI), Model 1	Reference	1.08(0.79-1.49)	0.91(0.65-1.25)	0.89(0.64-1.23)	0.96(0.70-1.32)	0.73
HR (95% CI), Model 2	Reference	1.11(0.81-1.52)	0.93(0.67-1.29)	0.93(0.67-1.28)	1.00(0.73-1.38)	0.78
Parkinson’s disease						
Events	41	51	41	44	57	
Person-years	123325.52	124321.57	124346.02	124355.8	124949.48	
HR (95% CI), Model 1	Reference	1.10(0.73-1.66)	0.83(0.54-1.28)	0.85(0.55-1.30)	1.04(0.70-1.56)	0.57
HR (95% CI), Model 2	Reference	1.11(0.74-1.68)	0.84(0.54-1.30)	0.85(0.56-1.32)	1.07(0.71-1.62)	0.54
Multiple sclerosis						
Events	18	11	9	15	8	
Person-years	123117.6	123968.8	124233.09	123976.03	124775.55	
HR (95% CI), Model 1	Reference	0.61(0.29-1.29)	0.50(0.23-1.12)	0.85(0.43-1.69)	0.46(0.20-1.06)	0.24
HR (95% CI), Model 2	Reference	0.60(0.28-1.27)	0.48(0.21-1.08)	0.80(0.40-1.61)	0.42(0.18-0.98)	0.20
Bronchiectasis						
Events	67	73	67	98	75	
Person-years	123003.58	124029.34	124045.52	123853.14	124668.52	
HR (95% CI), Model 1	Reference	0.98(0.70-1.37)	0.86(0.61-1.21)	1.20(0.88-1.64)	0.88(0.64-1.23)	0.20
HR (95% CI), Model 2	Reference	0.98(0.71-1.37)	0.86(0.61-1.21)	1.20(0.87-1.64)	0.88(0.63-1.23)	0.20
Asthma						
Events	214	199	199	208	193	
Person-years	105642.45	107566.69	107463.9	108519.59	109323.57	
HR (95% CI), Model 1	Reference	0.90(0.74-1.09)	0.88(0.73-1.07)	0.90(0.74-1.09)	0.82(0.68-1.00)	0.40
HR (95% CI), Model 2	Reference	0.98(0.81-1.19)	0.99(0.81-1.20)	1.03(0.85-1.26)	0.96(0.78-1.17)	0.96
COPD						
Events	230	216	217	198	212	
Person-years	121265.42	122628.54	122477.75	122757.38	122906.28	
HR (95% CI), Model 1	Reference	0.85(0.71-1.03)	0.82(0.68-0.98)	0.71(0.59-0.86)	0.73(0.61-0.89)	0.0032
HR (95% CI), Model 2	Reference	0.89(0.74-1.08)	0.85(0.70-1.02)	0.74(0.61-0.90)	0.72(0.59-0.87)	0.0045
CKD						
Events	624	503	482	462	420	
Person-years	120730.04	122256.57	122571.78	122567.17	123458.8	
HR (95% CI), Model 1	Reference	0.72(0.64-0.82)	0.65(0.58-0.74)	0.60(0.53-0.68)	0.52(0.46-0.59)	<0.0001
HR (95% CI), Model 2	Reference	0.81(0.72-0.91)	0.75(0.67-0.85)	0.72(0.64-0.81)	0.64(0.56-0.72)	<0.0001
Chronic liver disease						
Events	35	36	25	30	19	
Person-years	130398.9	130501.9	130421.65	130516.88	130629.09	

HR (95% CI), Model 1	Reference	0.94(0.57-1.57)	0.61(0.35-1.08)	0.72(0.42-1.24)	0.56(0.31-0.99)	0.18
HR (95% CI), Model 2	Reference	1.06(1.06-1.06)	0.71(0.71-0.71)	0.88(0.88-0.88)	0.69(0.69-0.69)	0.46
Irritable bowel syndrome						
Events	148	158	127	135	90	
Person-years	119061.83	120477.57	121173.71	121468.84	122296.7	
HR (95% CI), Model 1	Reference	1.03(0.82-1.29)	0.82(0.65-1.04)	0.86(0.68-1.09)	0.57(0.44-0.74)	0.0001
HR (95% CI), Model 2	Reference	1.10(0.88-1.38)	0.88(0.70-1.12)	0.95(0.75-1.20)	0.64(0.49-0.83)	0.0013
Inflammatory bowel disease						
Events	54	60	53	54	47	
Person-years	121776.25	122990.32	123221.75	123410.23	124144.08	
HR (95% CI), Model 1	Reference	1.10(0.76-1.59)	0.96(0.65-1.40)	0.96(0.66-1.41)	0.82(0.56-1.22)	0.70
HR (95% CI), Model 2	Reference	1.14(0.79-1.66)	1.00(0.68-1.46)	1.02(0.69-1.49)	0.87(0.58-1.30)	0.75
Treated constipation						
Events	430	447	391	395	378	
Person-years	120807.6	121642.91	121862.5	121665.37	122825.96	
HR (95% CI), Model 1	Reference	0.97(0.85-1.11)	0.82(0.71-0.94)	0.80(0.70-0.92)	0.74(0.65-0.85)	<0.0001
HR (95% CI), Model 2	Reference	1.00(0.87-1.14)	0.85(0.74-0.98)	0.85(0.74-0.97)	0.79(0.68-0.91)	0.0013
Dyspepsia						
Events	1289	1231	1110	1200	1101	
Person-years	102909.16	105929.22	106981.92	108023.39	108974.63	
HR (95% CI), Model 1	Reference	0.89(0.82-0.96)	0.78(0.72-0.84)	0.81(0.75-0.88)	0.73(0.67-0.79)	<0.0001
HR (95% CI), Model 2	Reference	0.94(0.87-1.01)	0.83(0.76-0.90)	0.88(0.81-0.96)	0.80(0.74-0.87)	<0.0001
Diverticular disease						
Events	1006	1033	939	993	969	
Person-years	114628.46	115907	116852.79	116617.76	117337.14	
HR (95% CI), Model 1	Reference	0.97(0.89-1.05)	0.84(0.77-0.92)	0.87(0.80-0.95)	0.82(0.75-0.90)	<0.0001
HR (95% CI), Model 2	Reference	1.01(0.93-1.11)	0.89(0.81-0.97)	0.93(0.85-1.02)	0.89(0.81-0.97)	0.0042
Pernicious anaemia						
Events	28	22	18	17	16	
Person-years	130441.16	130529.98	130615.45	130704.39	130771.66	
HR (95% CI), Model 1	Reference	0.76(0.42-1.36)	0.60(0.32-1.12)	0.55(0.29-1.05)	0.54(0.29-1.04)	0.25
HR (95% CI), Model 2	Reference	0.82(0.46-1.49)	0.66(0.35-1.25)	0.64(0.33-1.22)	0.64(0.33-1.24)	0.74
Fracture						
Events	78	80	86	91	119	
Person-years	122769.63	123695.37	123655.69	123608.43	124191.82	
HR (95% CI), Model 1	Reference	0.94(0.69-1.28)	0.97(0.72-1.32)	0.99(0.73-1.33)	1.25(0.94-1.67)	0.22
HR (95% CI), Model 2	Reference	0.90(0.66-1.23)	0.92(0.67-1.25)	0.91(0.67-1.23)	1.14(0.85-1.52)	0.40
Osteoporosis						
Events	745	763	713	759	709	
Person-years	109715.22	111882.31	110972.74	111117.14	112041.38	
HR (95% CI), Model 1	Reference	0.93(0.84-1.03)	0.85(0.76-0.94)	0.87(0.79-0.96)	0.78(0.71-0.87)	<0.0001
HR (95% CI), Model 2	Reference	1.01(0.91-1.12)	0.95(0.85-1.05)	1.00(0.90-1.11)	0.93(0.83-1.03)	0.36
Meniere's disease						
Events	7	13	17	15	6	

Person-years	123206.28	124181.49	124262.47	124093.6	124867.58	
HR (95% CI), Model 1	Reference	1.78(0.71-4.47)	2.28(0.94-5.51)	1.98(0.80-4.87)	0.77(0.26-2.31)	0.11
HR (95% CI), Model 2	Reference	1.91(0.76-4.80)	2.49(1.02-6.04)	2.21(0.89-5.50)	0.89(0.29-2.69)	0.0988
Eczema						
Events	124	165	147	135	134	
Person-years	117817.45	119354.68	118689.52	118921.69	120072.25	
HR (95% CI), Model 1	Reference	1.26(1.00-1.59)	1.10(0.87-1.40)	0.99(0.77-1.26)	0.95(0.75-1.22)	0.11
HR (95% CI), Model 2	Reference	1.31(1.04-1.66)	1.15(0.91-1.47)	1.04(0.81-1.34)	1.00(0.78-1.28)	0.0866
Glaucoma						
Events	159	189	208	197	195	
Person-years	121896.94	122486.03	122438.28	122380.16	122684.3	
HR (95% CI), Model 1	Reference	1.09(0.88-1.34)	1.15(0.93-1.41)	1.04(0.84-1.28)	0.99(0.80-1.22)	0.59
HR (95% CI), Model 2	Reference	1.07(0.86-1.32)	1.11(0.90-1.37)	1.00(0.81-1.24)	0.95(0.77-1.18)	0.57
Cataract						
Events	813	915	916	958	972	
Person-years	117254.67	117642.09	117379.98	117180.4	117484.54	
HR (95% CI), Model 1	Reference	1.01(0.91-1.11)	0.96(0.87-1.06)	0.95(0.86-1.04)	0.92(0.84-1.01)	0.32
HR (95% CI), Model 2	Reference	1.03(0.93-1.13)	0.99(0.90-1.09)	0.98(0.89-1.08)	0.96(0.87-1.06)	0.72
AMD						
Events	140	138	159	163	167	
Person-years	122797.42	123990.51	123743.03	123684.47	124331.7	
HR (95% CI), Model 1	Reference	0.88(0.69-1.11)	0.97(0.77-1.21)	0.93(0.74-1.17)	0.92(0.73-1.15)	0.84
HR (95% CI), Model 2	Reference	0.89(0.70-1.12)	0.99(0.78-1.24)	0.95(0.75-1.19)	0.94(0.75-1.19)	0.88
Thyroid disorders						
Events	297	266	273	266	197	
Person-years	114449.89	115764.84	116474.35	116358.86	117780.89	
HR (95% CI), Model 1	Reference	0.84(0.71-0.99)	0.84(0.71-0.99)	0.80(0.68-0.95)	0.58(0.49-0.70)	<0.0001
HR (95% CI), Model 2	Reference	0.88(0.75-1.04)	0.89(0.75-1.05)	0.86(0.73-1.02)	0.63(0.52-0.76)	<0.0001
Prostate disorders[¶]						
Events	372	403	440	427	404	
Person-years	52527.54	50225.1	50562.48	50722.09	52067.05	
HR (95% CI), Model 1	Reference	1.03(0.92-1.15)	1.06(0.95-1.18)	0.99(0.88-1.10)	0.88(0.79-0.99)	0.0105
HR (95% CI), Model 2	Reference	1.04(0.93-1.16)	1.07(0.96-1.19)	0.99(0.89-1.11)	0.89(0.79-1.00)	0.0135

AMD, age related macular degeneration; CI, confidence interval; CKD, chronic kidney disease; COPD, chronic obstructive pulmonary disease; HR, hazard ratio.

*Cox proportional hazard regression models were used to examine associations of the Anti-Empirical Dietary Inflammatory Index (quintiles) with the incidence of individual chronic diseases. The statistical tests were two-sided. Model 1 was adjusted for age and sex; Model 2 was adjusted for Model 1 plus ethnicity, education, income, smoking, alcohol consumption, sleep, physical activity, and GRS for longevity; Model 3 was adjusted for Model 2 and total energy intake.

[†]Cardiovascular disease includes coronary heart disease, heart failure, atrial fibrillation, other cardiac disease, stroke, and peripheral vascular disease.

[‡]All cancers encompass any type of cancer except for non-melanoma skin cancer.

[§]These analyses were conducted among women only.

[¶]These analyses were conducted among men only.

Table S11. The association between the Alternate Healthy Eating Index-2010 and the incidence of individual chronic diseases among individuals with three or more dietary assessments

	Alternate Healthy Eating Index-2010					P-value
	Quintile 1	Quintile 2	Quintile 3	Quintile 4	Quintile 5	for trend*
Cardiovascular disease†						
Events	1207	1104	1058	1001	984	
Person-years	114172.23	115821.02	116801.25	117589.28	117932.14	
HR (95% CI), Model 1	Reference	0.89(0.82-0.96)	0.82(0.76-0.90)	0.77(0.71-0.84)	0.77(0.70-0.83)	<0.0001
HR (95% CI), Model 2	Reference	0.92(0.85-1.00)	0.87(0.80-0.95)	0.83(0.76-0.90)	0.83(0.76-0.90)	<0.0001
Coronary heart disease						
Events	660	576	583	512	519	
Person-years	118977.76	120377.87	120740.36	121418.65	121864.19	
HR (95% CI), Model 1	Reference	0.86(0.77-0.96)	0.86(0.77-0.96)	0.76(0.67-0.85)	0.78(0.69-0.87)	<0.0001
HR (95% CI), Model 2	Reference	0.90(0.81-1.01)	0.92(0.82-1.03)	0.82(0.73-0.93)	0.86(0.76-0.97)	0.0164
Heart failure						
Events	214	177	162	169	162	
Person-years	122447.21	123639.18	123919.35	124350.12	124573.83	
HR (95% CI), Model 1	Reference	0.80(0.65-0.97)	0.71(0.58-0.88)	0.74(0.60-0.90)	0.71(0.58-0.87)	0.0030
HR (95% CI), Model 2	Reference	0.85(0.70-1.04)	0.79(0.64-0.97)	0.83(0.68-1.02)	0.82(0.67-1.01)	0.17
Atrial fibrillation						
Events	230	251	209	207	198	
Person-years	120729.05	121921.26	122481.28	122759.31	123070.88	
HR (95% CI), Model 1	Reference	1.06(0.89-1.27)	0.87(0.72-1.05)	0.85(0.71-1.03)	0.83(0.68-1.00)	0.0294
HR (95% CI), Model 2	Reference	1.11(0.92-1.32)	0.93(0.77-1.12)	0.92(0.76-1.11)	0.91(0.75-1.11)	0.19
Other cardiac disease						
Events	503	468	399	390	415	
Person-years	120978.44	122011.85	122660.79	123224.85	123282.15	
HR (95% CI), Model 1	Reference	0.90(0.79-1.02)	0.74(0.65-0.85)	0.72(0.63-0.82)	0.77(0.68-0.88)	<0.0001
HR (95% CI), Model 2	Reference	0.94(0.83-1.07)	0.79(0.69-0.90)	0.78(0.68-0.89)	0.84(0.73-0.95)	0.0004
Stroke						
Events	94	100	109	73	66	
Person-years	122501.1	123325.03	123803.03	124349.03	124598.46	
HR (95% CI), Model 1	Reference	1.03(0.78-1.36)	1.10(0.83-1.45)	0.73(0.53-0.99)	0.66(0.48-0.90)	0.0024
HR (95% CI), Model 2	Reference	1.07(0.80-1.42)	1.15(0.87-1.51)	0.76(0.56-1.04)	0.70(0.51-0.96)	0.0051
Peripheral vascular disease						
Events	117	113	87	115	91	
Person-years	122613.89	123640.77	124118.74	124436.17	124613.87	
HR (95% CI), Model 1	Reference	0.93(0.72-1.21)	0.70(0.53-0.93)	0.92(0.71-1.19)	0.73(0.55-0.96)	0.0411
HR (95% CI), Model 2	Reference	0.98(0.76-1.27)	0.75(0.56-0.99)	1.00(0.77-1.30)	0.78(0.59-1.03)	0.0873
Hypertension						
Events	554	502	466	400	411	
Person-years	89054	91784.79	92783.82	94012.94	94655.1	
HR (95% CI), Model 1	Reference	0.85(0.76-0.96)	0.77(0.68-0.87)	0.65(0.57-0.74)	0.66(0.58-0.75)	<0.0001
HR (95% CI), Model 2	Reference	0.91(0.81-1.03)	0.83(0.74-0.94)	0.73(0.64-0.83)	0.75(0.66-0.86)	<0.0001

Diabetes						
Events	372	274	285	290	253	
Person-years	117802.55	119341.01	119825.34	119714.14	119894.84	
HR (95% CI), Model 1	Reference	0.73(0.62-0.85)	0.75(0.64-0.87)	0.76(0.65-0.89)	0.67(0.57-0.79)	<0.0001
HR (95% CI), Model 2	Reference	0.82(0.70-0.96)	0.89(0.76-1.04)	0.95(0.81-1.11)	0.86(0.73-1.01)	0.10
All cancers[‡]						
Events	859	746	752	737	697	
Person-years	110557.7	113284.15	113401.81	114094.05	114141.59	
HR (95% CI), Model 1	Reference	0.81(0.73-0.89)	0.80(0.72-0.88)	0.77(0.69-0.85)	0.72(0.65-0.79)	<0.0001
HR (95% CI), Model 2	Reference	0.84(0.76-0.93)	0.84(0.76-0.93)	0.82(0.74-0.90)	0.77(0.70-0.86)	<0.0001
Non-melanoma skin cancer						
Events	450	438	455	452	424	
Person-years	119769.13	120898.38	121139.61	121554.7	121801.08	
HR (95% CI), Model 1	Reference	0.93(0.82-1.07)	0.95(0.84-1.08)	0.93(0.82-1.07)	0.88(0.77-1.00)	0.42
HR (95% CI), Model 2	Reference	0.91(0.79-1.03)	0.91(0.80-1.04)	0.89(0.78-1.02)	0.82(0.71-0.94)	0.0768
Melanoma						
Events	66	53	52	60	64	
Person-years	121837.6	123075.72	123217.31	123659.46	123797.65	
HR (95% CI), Model 1	Reference	0.77(0.54-1.11)	0.74(0.52-1.07)	0.85(0.60-1.21)	0.90(0.63-1.27)	0.50
HR (95% CI), Model 2	Reference	0.76(0.53-1.10)	0.74(0.51-1.07)	0.85(0.60-1.21)	0.91(0.64-1.29)	0.46
Lung cancer						
Events	107	62	72	71	63	
Person-years	122847.82	124120.25	124270.69	124728.71	124990.65	
HR (95% CI), Model 1	Reference	0.54(0.39-0.73)	0.61(0.45-0.82)	0.58(0.43-0.79)	0.50(0.37-0.69)	<0.0001
HR (95% CI), Model 2	Reference	0.65(0.48-0.88)	0.76(0.57-1.01)	0.77(0.58-1.03)	0.74(0.54-0.99)	0.0500
Stomach cancer						
Events	21	19	23	19	16	
Person-years	123249.8	124316.3	124512.93	124904.01	125149.78	
HR (95% CI), Model 1	Reference	0.92(0.49-1.71)	1.12(0.62-2.03)	0.94(0.50-1.75)	0.82(0.43-1.59)	0.91
HR (95% CI), Model 2	Reference	0.95(0.51-1.78)	1.19(0.66-2.16)	1.01(0.54-1.89)	0.90(0.46-1.75)	0.93
Oesophageal cancer						
Events	27	22	29	17	25	
Person-years	123212.39	124290.35	124487.41	124974.94	125168.22	
HR (95% CI), Model 1	Reference	0.81(0.46-1.42)	1.06(0.63-1.79)	0.62(0.34-1.15)	0.95(0.55-1.64)	0.45
HR (95% CI), Model 2	Reference	0.89(0.50-1.56)	1.20(0.71-2.03)	0.72(0.39-1.33)	1.14(0.65-1.99)	0.48
Colon cancer						
Events	107	106	113	96	81	
Person-years	122139.08	123588.69	123666	124229.31	124412.66	
HR (95% CI), Model 1	Reference	0.94(0.72-1.24)	0.99(0.76-1.29)	0.82(0.62-1.09)	0.69(0.52-0.92)	0.0695
HR (95% CI), Model 2	Reference	0.98(0.75-1.28)	1.04(0.80-1.36)	0.88(0.67-1.17)	0.75(0.56-1.01)	0.20
Ovarian cancer[§]						
Events	24	29	28	23	33	
Person-years	62052.67	71004.97	73998.89	77532.83	82911.81	
HR (95% CI), Model 1	Reference	1.00(0.58-1.72)	0.91(0.53-1.57)	0.70(0.39-1.23)	0.91(0.53-1.54)	0.71

HR (95% CI), Model 2	Reference	1.02(0.59-1.75)	0.93(0.54-1.60)	0.71(0.40-1.26)	0.92(0.54-1.57)	0.73
Breast cancer§						
Events	225	224	235	221	265	
Person-years	58157.3	66945.56	69725.92	73041.8	77853.51	
HR (95% CI), Model 1	Reference	0.85(0.71-1.02)	0.85(0.71-1.02)	0.75(0.63-0.91)	0.84(0.70-1.00)	0.0604
HR (95% CI), Model 2	Reference	0.86(0.72-1.04)	0.87(0.72-1.04)	0.78(0.65-0.94)	0.87(0.73-1.05)	0.14
Prostate cancer¶						
Events	294	274	296	226	185	
Person-years	65906.84	57076.46	54441.04	51575.95	46445.63	
HR (95% CI), Model 1	Reference	1.07(0.93-1.22)	1.16(1.02-1.32)	0.99(0.86-1.14)	0.93(0.81-1.07)	0.0234
HR (95% CI), Model 2	Reference	1.05(0.92-1.19)	1.13(0.99-1.28)	0.97(0.84-1.11)	0.90(0.78-1.04)	0.0266
Other cancers						
Events	741	666	623	624	588	
Person-years	112890.84	115141.69	115529.45	116008.5	116195.48	
HR (95% CI), Model 1	Reference	0.85(0.76-0.94)	0.77(0.69-0.86)	0.76(0.68-0.84)	0.71(0.63-0.79)	<0.0001
HR (95% CI), Model 2	Reference	0.88(0.79-0.97)	0.81(0.73-0.90)	0.81(0.72-0.90)	0.76(0.68-0.85)	<0.0001
Depression						
Events	119	119	100	85	118	
Person-years	115906.5	117226.38	117989.48	118624.08	118458.55	
HR (95% CI), Model 1	Reference	0.96(0.74-1.24)	0.79(0.61-1.04)	0.66(0.50-0.88)	0.91(0.70-1.17)	0.0313
HR (95% CI), Model 2	Reference	1.02(0.79-1.31)	0.85(0.65-1.11)	0.72(0.54-0.96)	0.97(0.75-1.27)	0.0925
Anxiety						
Events	361	358	329	305	374	
Person-years	119634.23	120776.94	121283.32	121850.01	121376.24	
HR (95% CI), Model 1	Reference	0.93(0.80-1.07)	0.83(0.71-0.96)	0.75(0.64-0.87)	0.89(0.77-1.03)	0.0027
HR (95% CI), Model 2	Reference	0.96(0.83-1.12)	0.87(0.75-1.02)	0.80(0.68-0.93)	0.94(0.81-1.09)	0.0314
Schizophrenia						
Events	21	11	13	14	12	
Person-years	122975.77	123997.83	124163.45	124685.35	124874.91	
HR (95% CI), Model 1	Reference	0.51(0.25-1.06)	0.60(0.30-1.20)	0.64(0.32-1.26)	0.54(0.26-1.10)	0.3065
HR (95% CI), Model 2	Reference	0.57(0.28-1.19)	0.70(0.35-1.40)	0.77(0.39-1.53)	0.68(0.33-1.40)	0.6163
Alcohol use disorder						
Events	179	98	73	64	38	
Person-years	121987.21	123606.06	124016.34	124485.51	124846.7	
HR (95% CI), Model 1	Reference	0.56(0.44-0.71)	0.42(0.32-0.55)	0.37(0.28-0.49)	0.22(0.16-0.32)	<0.0001
HR (95% CI), Model 2	Reference	0.59(0.46-0.76)	0.45(0.34-0.59)	0.41(0.31-0.55)	0.25(0.18-0.36)	<0.0001
Psychoactive substance abuse						
Events	36	14	11	10	11	
Person-years	123079.21	124261.43	124442.27	124965.61	125071.09	
HR (95% CI), Model 1	Reference	0.39(0.21-0.73)	0.31(0.16-0.61)	0.28(0.14-0.56)	0.31(0.16-0.61)	<0.0001
HR (95% CI), Model 2	Reference	0.44(0.44-0.44)	0.35(0.35-0.35)	0.33(0.33-0.33)	0.37(0.37-0.37)	0.0009
Epilepsy						
Events	51	40	38	36	39	
Person-years	122310.54	123400.99	123561.03	124107.83	124251.6	

HR (95% CI), Model 1	Reference	0.76(0.50-1.15)	0.71(0.47-1.09)	0.67(0.44-1.03)	0.72(0.47-1.10)	0.34
HR (95% CI), Model 2	Reference	0.78(0.51-1.18)	0.74(0.48-1.13)	0.70(0.45-1.08)	0.77(0.50-1.17)	0.49
Migraine						
Events	86	79	72	76	81	
Person-years	119773.68	120347	120429.61	120167.54	119985.96	
HR (95% CI), Model 1	Reference	0.88(0.65-1.19)	0.79(0.58-1.09)	0.83(0.61-1.13)	0.87(0.64-1.19)	0.66
HR (95% CI), Model 2	Reference	0.89(0.66-1.21)	0.81(0.59-1.11)	0.85(0.62-1.16)	0.88(0.65-1.20)	0.75
Dementia						
Events	97	66	81	67	90	
Person-years	123060.31	124261.39	124481.22	124890.68	125023.03	
HR (95% CI), Model 1	Reference	0.63(0.46-0.85)	0.74(0.55-1.00)	0.59(0.43-0.81)	0.78(0.58-1.04)	0.0071
HR (95% CI), Model 2	Reference	0.64(0.47-0.88)	0.76(0.56-1.02)	0.61(0.44-0.83)	0.80(0.59-1.07)	0.0138
Parkinson’s disease						
Events	52	52	45	43	42	
Person-years	123053.98	124181.68	124290.66	124746.2	125025.86	
HR (95% CI), Model 1	Reference	0.96(0.65-1.41)	0.81(0.54-1.21)	0.77(0.51-1.15)	0.75(0.50-1.14)	0.53
HR (95% CI), Model 2	Reference	0.96(0.65-1.41)	0.80(0.53-1.19)	0.76(0.50-1.14)	0.73(0.48-1.11)	0.46
Multiple sclerosis						
Events	12	21	9	9	10	
Person-years	122761.7	123895.35	124130.31	124587.6	124696.11	
HR (95% CI), Model 1	Reference	1.71(0.84-3.48)	0.73(0.31-1.74)	0.73(0.31-1.73)	0.80(0.34-1.87)	0.0891
HR (95% CI), Model 2	Reference	1.74(0.85-3.54)	0.76(0.32-1.82)	0.76(0.32-1.83)	0.86(0.37-2.02)	0.11
Bronchiectasis						
Events	87	86	71	68	68	
Person-years	122675.13	123805.3	124006.32	124429.58	124683.77	
HR (95% CI), Model 1	Reference	0.90(0.67-1.22)	0.72(0.52-0.98)	0.66(0.48-0.91)	0.64(0.46-0.88)	0.0181
HR (95% CI), Model 2	Reference	0.92(0.68-1.24)	0.73(0.53-1.00)	0.67(0.49-0.93)	0.63(0.46-0.88)	0.0207
Asthma						
Events	245	188	208	189	183	
Person-years	105675.17	107237.64	108580.71	108276.43	108746.26	
HR (95% CI), Model 1	Reference	0.73(0.60-0.88)	0.78(0.65-0.94)	0.70(0.58-0.85)	0.66(0.55-0.81)	0.0002
HR (95% CI), Model 2	Reference	0.76(0.63-0.92)	0.83(0.69-1.00)	0.75(0.62-0.91)	0.71(0.58-0.86)	0.0042
COPD						
Events	279	234	220	176	164	
Person-years	120761.86	121962.57	122584.65	123269.18	123457.11	
HR (95% CI), Model 1	Reference	0.79(0.67-0.94)	0.72(0.61-0.86)	0.56(0.47-0.68)	0.52(0.43-0.63)	<0.0001
HR (95% CI), Model 2	Reference	0.91(0.76-1.08)	0.87(0.73-1.04)	0.72(0.59-0.87)	0.67(0.55-0.81)	0.0002
CKD						
Events	572	554	483	447	435	
Person-years	120896.75	121828.24	122386.1	123199.58	123273.68	
HR (95% CI), Model 1	Reference	0.93(0.82-1.04)	0.78(0.69-0.88)	0.71(0.63-0.80)	0.69(0.61-0.78)	<0.0001
HR (95% CI), Model 2	Reference	0.99(0.88-1.11)	0.86(0.76-0.97)	0.79(0.70-0.90)	0.78(0.69-0.89)	<0.0001
Chronic liver disease						
Events	40	37	24	21	23	
Person-years	130049.31	130383.3	130602.39	130604.71	130828.71	

HR (95% CI), Model 1	Reference	0.90(0.55-1.46)	0.60(0.35-1.04)	0.49(0.27-0.88)	0.58(0.33-1.01)	0.0593
HR (95% CI), Model 2	Reference	0.99(0.99-0.99)	0.70(0.70-0.70)	0.59(0.59-0.59)	0.74(0.74-0.74)	0.32
Irritable bowel syndrome						
Events	152	140	117	118	131	
Person-years	119852.87	120649.95	121399.44	121184.1	121392.3	
HR (95% CI), Model 1	Reference	0.86(0.68-1.08)	0.68(0.54-0.87)	0.68(0.53-0.87)	0.72(0.57-0.91)	0.0042
HR (95% CI), Model 2	Reference	0.89(0.71-1.12)	0.73(0.57-0.93)	0.73(0.57-0.93)	0.78(0.62-0.99)	0.0419
Inflammatory bowel disease						
Events	65	51	57	58	37	
Person-years	121514.22	122883.61	123196.88	123828.12	124119.8	
HR (95% CI), Model 1	Reference	0.77(0.53-1.11)	0.85(0.60-1.22)	0.86(0.60-1.22)	0.54(0.36-0.81)	0.0545
HR (95% CI), Model 2	Reference	0.80(0.55-1.15)	0.89(0.62-1.28)	0.91(0.63-1.30)	0.57(0.38-0.86)	0.0934
Treated constipation						
Events	453	420	380	368	420	
Person-years	120044	121665.48	121998.45	122487.01	122609.39	
HR (95% CI), Model 1	Reference	0.88(0.77-1.00)	0.77(0.67-0.88)	0.73(0.64-0.84)	0.82(0.72-0.94)	0.0001
HR (95% CI), Model 2	Reference	0.89(0.78-1.02)	0.78(0.68-0.90)	0.75(0.65-0.86)	0.83(0.73-0.95)	0.0004
Dyspepsia						
Events	1293	1234	1122	1141	1141	
Person-years	103891.58	105545.58	107785.16	107632.4	107963.59	
HR (95% CI), Model 1	Reference	0.91(0.84-0.98)	0.79(0.73-0.86)	0.80(0.74-0.87)	0.79(0.73-0.85)	<0.0001
HR (95% CI), Model 2	Reference	0.94(0.87-1.02)	0.83(0.77-0.90)	0.85(0.78-0.92)	0.83(0.76-0.90)	<0.0001
Diverticular disease						
Events	1150	1013	980	930	867	
Person-years	113817.7	115624.69	116557.02	117370.9	117972.85	
HR (95% CI), Model 1	Reference	0.84(0.77-0.91)	0.79(0.72-0.86)	0.73(0.67-0.80)	0.67(0.62-0.74)	<0.0001
HR (95% CI), Model 2	Reference	0.87(0.80-0.95)	0.84(0.77-0.92)	0.80(0.73-0.87)	0.74(0.68-0.81)	<0.0001
Pernicious anaemia						
Events	30	19	18	20	14	
Person-years	130170.77	130575.03	130727.94	130771.55	130817.36	
HR (95% CI), Model 1	Reference	0.57(0.31-1.05)	0.56(0.30-1.04)	0.62(0.34-1.13)	0.48(0.25-0.92)	0.15
HR (95% CI), Model 2	Reference	0.59(0.59-0.59)	0.59(0.59-0.59)	0.66(0.66-0.66)	0.50(0.26-0.97)	0.23
Fracture						
Events	88	83	81	89	113	
Person-years	122263.34	123589.75	123820.06	124082.23	124165.55	
HR (95% CI), Model 1	Reference	0.86(0.64-1.16)	0.81(0.60-1.09)	0.86(0.64-1.16)	1.05(0.79-1.39)	0.31
HR (95% CI), Model 2	Reference	0.86(0.63-1.16)	0.80(0.59-1.08)	0.84(0.63-1.14)	1.01(0.76-1.35)	0.37
Osteoporosis						
Events	793	773	745	693	685	
Person-years	109659.15	111101.47	111331.01	111731.95	111905.21	
HR (95% CI), Model 1	Reference	0.90(0.81-0.99)	0.84(0.76-0.92)	0.76(0.68-0.84)	0.73(0.65-0.80)	<0.0001
HR (95% CI), Model 2	Reference	0.94(0.85-1.04)	0.90(0.81-0.99)	0.82(0.74-0.91)	0.80(0.72-0.89)	<0.0001
Meniere's disease						
Events	17	7	8	14	12	

Person-years	122853.28	124079.95	124281.59	124604.61	124791.98	
HR (95% CI), Model 1	Reference	0.40(0.16-0.95)	0.44(0.19-1.03)	0.77(0.38-1.57)	0.65(0.31-1.37)	0.18
HR (95% CI), Model 2	Reference	0.40(0.17-0.97)	0.46(0.20-1.08)	0.81(0.39-1.65)	0.70(0.33-1.48)	0.21
Eczema						
Events	184	137	129	119	136	
Person-years	117338.86	118904.71	119044.5	119877.19	119690.33	
HR (95% CI), Model 1	Reference	0.72(0.57-0.89)	0.67(0.53-0.84)	0.61(0.48-0.77)	0.69(0.55-0.87)	0.0001
HR (95% CI), Model 2	Reference	0.73(0.58-0.91)	0.69(0.55-0.86)	0.63(0.50-0.79)	0.70(0.56-0.88)	0.0006
Glaucoma						
Events	175	186	176	202	209	
Person-years	121333.49	122262.69	122539.5	122839.2	122910.83	
HR (95% CI), Model 1	Reference	1.01(0.82-1.24)	0.93(0.76-1.15)	1.06(0.86-1.30)	1.09(0.89-1.33)	0.64
HR (95% CI), Model 2	Reference	1.01(0.82-1.25)	0.93(0.75-1.15)	1.05(0.85-1.29)	1.06(0.86-1.30)	0.74
Cataract						
Events	874	895	884	968	953	
Person-years	116414.08	117627.14	117787.98	117621.73	117490.75	
HR (95% CI), Model 1	Reference	0.93(0.84-1.02)	0.88(0.80-0.96)	0.94(0.86-1.03)	0.89(0.81-0.98)	0.0637
HR (95% CI), Model 2	Reference	0.95(0.86-1.04)	0.90(0.82-0.99)	0.97(0.88-1.06)	0.92(0.83-1.01)	0.19
AMD						
Events	149	139	156	155	168	
Person-years	122572.32	123639.54	123898.84	124253.68	124182.76	
HR (95% CI), Model 1	Reference	0.85(0.67-1.07)	0.91(0.73-1.14)	0.87(0.69-1.09)	0.90(0.72-1.13)	0.66
HR (95% CI), Model 2	Reference	0.85(0.67-1.07)	0.92(0.73-1.16)	0.88(0.70-1.11)	0.90(0.72-1.13)	0.71
Thyroid disorders						
Events	253	234	247	296	269	
Person-years	115784.57	116506.69	116315.06	116419.53	115802.98	
HR (95% CI), Model 1	Reference	0.85(0.71-1.02)	0.88(0.74-1.05)	1.02(0.86-1.21)	0.90(0.76-1.07)	0.14
HR (95% CI), Model 2	Reference	0.87(0.73-1.05)	0.92(0.77-1.10)	1.08(0.91-1.28)	0.97(0.82-1.16)	0.14
Prostate disorders¶						
Events	512	428	394	386	326	
Person-years	61269.76	53006.62	51203.03	47789.05	42835.8	
HR (95% CI), Model 1	Reference	0.97(0.87-1.07)	0.86(0.78-0.96)	0.95(0.86-1.06)	0.86(0.77-0.96)	0.0175
HR (95% CI), Model 2	Reference	0.97(0.88-1.08)	0.87(0.78-0.97)	0.96(0.87-1.07)	0.87(0.78-0.98)	0.0271

AMD, age related macular degeneration; CI, confidence interval; CKD, chronic kidney disease; COPD, chronic obstructive pulmonary disease; HR, hazard ratio.

*Cox proportional hazard regression models were used to examine associations of the Alternate Healthy Eating Index-2010 (quintiles) with the incidence of individual chronic diseases. The statistical tests were two-sided. Model 1 was adjusted for age and sex; Model 2 was adjusted for Model 1 plus ethnicity, education, income, smoking, alcohol consumption, sleep, physical activity, and GRS for longevity; Model 3 was adjusted for Model 2 and total energy intake.

†Cardiovascular disease includes coronary heart disease, heart failure, atrial fibrillation, other cardiac disease, stroke, and peripheral vascular disease.

‡All cancers encompass any type of cancer except for non-melanoma skin cancer.

[§]These analyses were conducted among women only.

[¶]These analyses were conducted among men only.

Table S12. The association between the Healthful Plant-based Diet Index and the incidence of individual chronic diseases among individuals with three or more dietary assessments

	Healthful Plant-based Diet Index					P-value
	Quintile 1	Quintile 2	Quintile 3	Quintile 4	Quintile 5	for trend*
Cardiovascular disease[†]						
Events	1385	826	916	1238	989	
Person-years	128785.15	88270.18	102205.13	136520.04	126535.4	
HR (95% CI), Model 1	Reference	0.87(0.80-0.95)	0.83(0.76-0.90)	0.87(0.81-0.94)	0.79(0.72-0.85)	<0.0001
HR (95% CI), Model 2	Reference	0.90(0.83-0.98)	0.88(0.81-0.96)	0.93(0.86-1.01)	0.87(0.80-0.95)	0.0068
Coronary heart disease						
Events	773	455	492	637	493	
Person-years	134147.62	91604.65	105793.33	141355.62	130477.59	
HR (95% CI), Model 1	Reference	0.88(0.78-0.99)	0.83(0.74-0.93)	0.84(0.76-0.94)	0.75(0.67-0.85)	<0.0001
HR (95% CI), Model 2	Reference	0.92(0.82-1.03)	0.89(0.79-1.00)	0.92(0.83-1.03)	0.85(0.76-0.96)	0.10
Heart failure						
Events	235	146	154	198	151	
Person-years	138443.54	94206.07	108580.69	144492.7	133206.69	
HR (95% CI), Model 1	Reference	0.92(0.74-1.13)	0.84(0.68-1.03)	0.85(0.70-1.03)	0.75(0.60-0.92)	0.0935
HR (95% CI), Model 2	Reference	0.98(0.80-1.21)	0.94(0.77-1.16)	0.97(0.80-1.18)	0.91(0.74-1.14)	0.94
Atrial fibrillation						
Events	293	152	190	273	187	
Person-years	136302.47	93064.07	107236.41	142674.8	131684.03	
HR (95% CI), Model 1	Reference	0.78(0.64-0.95)	0.86(0.71-1.03)	0.99(0.83-1.17)	0.80(0.66-0.97)	0.0197
HR (95% CI), Model 2	Reference	0.81(0.67-0.99)	0.92(0.77-1.11)	1.07(0.91-1.27)	0.91(0.75-1.10)	0.0574
Other cardiac disease						
Events	551	358	357	493	416	
Person-years	136497.57	93129.15	107403.33	143134.29	131993.74	
HR (95% CI), Model 1	Reference	0.94(0.82-1.07)	0.81(0.71-0.92)	0.86(0.76-0.98)	0.82(0.72-0.94)	0.0066
HR (95% CI), Model 2	Reference	0.98(0.86-1.12)	0.86(0.75-0.99)	0.93(0.82-1.05)	0.91(0.79-1.04)	0.21
Stroke						
Events	113	67	74	115	73	
Person-years	138322.69	94234.1	108579.33	144409.09	133031.45	
HR (95% CI), Model 1	Reference	0.85(0.63-1.16)	0.81(0.61-1.10)	0.97(0.75-1.27)	0.70(0.51-0.95)	0.12
HR (95% CI), Model 2	Reference	0.87(0.64-1.18)	0.84(0.63-1.14)	1.01(0.77-1.33)	0.74(0.54-1.01)	0.21
Peripheral vascular disease						
Events	131	74	95	121	102	
Person-years	138530.35	94388.62	108671.57	144693.85	133139.04	
HR (95% CI), Model 1	Reference	0.81(0.61-1.07)	0.89(0.68-1.16)	0.85(0.66-1.10)	0.79(0.60-1.04)	0.45
HR (95% CI), Model 2	Reference	0.82(0.62-1.10)	0.92(0.71-1.21)	0.90(0.70-1.16)	0.85(0.64-1.11)	0.68
Hypertension						
Events	624	344	437	521	407	
Person-years	98617.7	69215.58	80609.47	109498.95	104348.95	
HR (95% CI), Model 1	Reference	0.76(0.67-0.87)	0.83(0.73-0.94)	0.74(0.65-0.83)	0.61(0.54-0.70)	<0.0001
HR (95% CI), Model 2	Reference	0.81(0.71-0.92)	0.91(0.80-1.03)	0.82(0.73-0.93)	0.73(0.64-0.83)	<0.0001

Diabetes						
Events	463	252	268	266	225	
Person-years	131981.99	90771.49	104759.22	139909.14	129156.05	
HR (95% CI), Model 1	Reference	0.80(0.68-0.93)	0.74(0.63-0.86)	0.56(0.48-0.65)	0.53(0.45-0.62)	<0.0001
HR (95% CI), Model 2	Reference	0.90(0.77-1.05)	0.88(0.76-1.03)	0.71(0.61-0.83)	0.75(0.63-0.89)	0.0002
All cancers‡						
Events	950	586	678	843	734	
Person-years	125723.21	85677.56	99605.81	132053.09	122419.62	
HR (95% CI), Model 1	Reference	0.87(0.79-0.97)	0.85(0.77-0.94)	0.80(0.73-0.88)	0.76(0.69-0.84)	<0.0001
HR (95% CI), Model 2	Reference	0.89(0.81-0.99)	0.89(0.80-0.98)	0.84(0.76-0.92)	0.81(0.74-0.90)	0.0008
Non-melanoma skin cancer						
Events	523	337	404	504	451	
Person-years	135296.34	91993.69	106222.06	141380.52	130270.28	
HR (95% CI), Model 1	Reference	0.93(0.81-1.07)	0.96(0.84-1.09)	0.92(0.81-1.04)	0.92(0.81-1.05)	0.65
HR (95% CI), Model 2	Reference	0.91(0.79-1.04)	0.92(0.81-1.05)	0.88(0.77-1.00)	0.86(0.75-0.98)	0.18
Melanoma						
Events	69	45	64	51	66	
Person-years	138010.38	93722.62	108139.58	143502.6	132212.57	
HR (95% CI), Model 1	Reference	0.92(0.63-1.34)	1.11(0.79-1.57)	0.66(0.46-0.96)	0.92(0.65-1.32)	0.0830
HR (95% CI), Model 2	Reference	0.91(0.63-1.33)	1.10(0.78-1.56)	0.66(0.45-0.95)	0.91(0.64-1.31)	0.0843
Lung cancer						
Events	87	63	63	89	73	
Person-years	138978.39	94491.05	108979.71	144964.76	133544.21	
HR (95% CI), Model 1	Reference	0.99(0.71-1.37)	0.83(0.60-1.15)	0.86(0.64-1.17)	0.76(0.55-1.05)	0.44
HR (95% CI), Model 2	Reference	1.15(0.84-1.56)	1.01(0.74-1.38)	1.04(0.77-1.38)	0.99(0.72-1.35)	0.90
Stomach cancer						
Events	26	15	21	20	16	
Person-years	139222.55	94788.15	109148.2	145225.01	133748.91	
HR (95% CI), Model 1	Reference	0.89(0.47-1.69)	1.12(0.62-2.00)	0.87(0.48-1.57)	0.83(0.44-1.59)	0.90
HR (95% CI), Model 2	Reference	0.92(0.49-1.74)	1.17(0.65-2.09)	0.92(0.50-1.67)	0.89(0.46-1.72)	0.92
Oesophageal cancer						
Events	32	18	19	26	25	
Person-years	139206.95	94743.5	109171.84	145273.34	133737.67	
HR (95% CI), Model 1	Reference	0.84(0.47-1.51)	0.78(0.44-1.38)	0.86(0.50-1.45)	0.97(0.56-1.67)	0.91
HR (95% CI), Model 2	Reference	0.91(0.51-1.63)	0.89(0.50-1.58)	1.00(0.59-1.71)	1.22(0.70-2.12)	0.86
Colon cancer						
Events	144	75	95	119	70	
Person-years	138061.95	94024.57	108387.16	144432.16	133129.89	
HR (95% CI), Model 1	Reference	0.75(0.57-0.99)	0.82(0.63-1.07)	0.79(0.61-1.01)	0.52(0.39-0.70)	0.0007
HR (95% CI), Model 2	Reference	0.77(0.58-1.02)	0.86(0.66-1.12)	0.84(0.65-1.08)	0.57(0.42-0.77)	0.0063
Ovarian cancer§						
Events	31	26	25	28	27	
Person-years	61307.32	51043.12	63234.19	94803.73	97112.81	
HR (95% CI), Model 1	Reference	0.98(0.58-1.66)	0.76(0.45-1.29)	0.56(0.34-0.95)	0.54(0.32-0.91)	0.0541

HR (95% CI), Model 2	Reference	1.03(0.61-1.73)	0.80(0.47-1.37)	0.61(0.36-1.03)	0.60(0.35-1.03)	0.0640
Breast cancer§						
Events	198	170	198	303	301	
Person-years	57669.29	48122.28	59523.44	89018.57	91390.52	
HR (95% CI), Model 1	Reference	1.03(0.84-1.26)	0.97(0.79-1.18)	0.99(0.83-1.19)	0.97(0.81-1.17)	0.98
HR (95% CI), Model 2	Reference	1.04(0.85-1.28)	0.99(0.81-1.21)	1.03(0.86-1.24)	1.02(0.84-1.23)	0.99
Prostate cancer¶						
Events	385	197	246	275	172	
Person-years	82897.03	47057.73	49948.06	55066.75	40476.35	
HR (95% CI), Model 1	Reference	0.95(0.80-1.13)	1.07(0.95-1.19)	1.12(0.99-1.27)	0.96(0.83-1.11)	0.15
HR (95% CI), Model 2	Reference	0.94(0.79-1.12)	1.04(0.93-1.17)	1.09(0.97-1.24)	0.93(0.80-1.07)	0.18
Other cancers						
Events	829	500	563	718	632	
Person-years	128059.99	87481.43	101213.18	134616.38	124394.98	
HR (95% CI), Model 1	Reference	0.85(0.76-0.95)	0.81(0.73-0.91)	0.78(0.70-0.86)	0.75(0.67-0.83)	<0.0001
HR (95% CI), Model 2	Reference	0.87(0.78-0.97)	0.85(0.76-0.95)	0.82(0.74-0.91)	0.81(0.72-0.90)	0.0005
Depression						
Events	118	90	96	135	102	
Person-years	131278.78	89463.73	102868.6	137643.34	126950.54	
HR (95% CI), Model 1	Reference	1.07(0.81-1.40)	0.97(0.74-1.27)	0.98(0.76-1.27)	0.78(0.59-1.03)	0.24
HR (95% CI), Model 2	Reference	1.14(0.87-1.51)	1.06(0.81-1.40)	1.10(0.85-1.43)	0.91(0.68-1.20)	0.49
Anxiety						
Events	394	289	295	386	363	
Person-years	135465.21	91969.56	106182.29	141185.34	130118.35	
HR (95% CI), Model 1	Reference	1.01(0.87-1.18)	0.88(0.75-1.02)	0.83(0.72-0.96)	0.82(0.71-0.95)	0.0109
HR (95% CI), Model 2	Reference	1.06(0.91-1.23)	0.93(0.80-1.08)	0.89(0.77-1.03)	0.90(0.78-1.05)	0.19
Schizophrenia						
Events	10	16	14	21	10	
Person-years	138933.49	94513.59	108932.33	144894.64	133423.28	
HR (95% CI), Model 1	Reference	2.41(1.09-5.33)	1.85(0.82-4.21)	2.12(0.98-4.57)	1.12(0.45-2.76)	0.11
HR (95% CI), Model 2	Reference	2.70(1.22-5.98)	2.25(0.99-5.12)	2.74(1.26-5.95)	1.58(0.63-3.94)	0.0691
Alcohol use disorder						
Events	125	85	90	93	59	
Person-years	138243.17	94107.82	108521.08	144719.92	133349.83	
HR (95% CI), Model 1	Reference	1.08(0.82-1.42)	1.02(0.78-1.35)	0.85(0.65-1.12)	0.64(0.46-0.88)	0.0200
HR (95% CI), Model 2	Reference	1.11(0.84-1.47)	1.08(0.82-1.42)	0.90(0.68-1.19)	0.69(0.50-0.96)	0.0536
Psychoactive substance abuse						
Events	19	17	14	17	15	
Person-years	139187.73	94740.04	109016.94	145219.15	133655.74	
HR (95% CI), Model 1	Reference	1.43(0.74-2.76)	1.06(0.53-2.13)	1.03(0.53-2.01)	1.06(0.52-2.15)	0.84
HR (95% CI), Model 2	Reference	1.54(0.80-2.98)	1.19(0.59-2.39)	1.16(0.59-2.28)	1.26(0.62-2.59)	0.79
Epilepsy						
Events	56	35	30	50	33	
Person-years	138066.19	93915.4	108403.53	144409.04	132837.84	

HR (95% CI), Model 1	Reference	0.91(0.60-1.39)	0.68(0.43-1.06)	0.86(0.58-1.27)	0.63(0.40-0.98)	0.21
HR (95% CI), Model 2	Reference	0.92(0.60-1.41)	0.69(0.44-1.09)	0.88(0.60-1.31)	0.66(0.42-1.04)	0.31
Migraine						
Events	91	54	54	108	87	
Person-years	135058.88	91655.66	105501.26	139793.04	128694.94	
HR (95% CI), Model 1	Reference	0.79(0.57-1.11)	0.66(0.47-0.93)	0.94(0.70-1.25)	0.77(0.56-1.04)	0.0968
HR (95% CI), Model 2	Reference	0.80(0.57-1.12)	0.66(0.47-0.94)	0.94(0.71-1.26)	0.77(0.56-1.05)	0.10
Dementia						
Events	89	67	66	102	77	
Person-years	139138.08	94707.35	109078.71	145132.81	133659.67	
HR (95% CI), Model 1	Reference	1.04(0.76-1.44)	0.87(0.63-1.20)	1.02(0.76-1.36)	0.86(0.63-1.18)	0.65
HR (95% CI), Model 2	Reference	1.07(0.78-1.47)	0.90(0.65-1.24)	1.05(0.78-1.41)	0.90(0.65-1.24)	0.73
Parkinson’s disease						
Events	54	33	43	62	42	
Person-years	138956.63	94658.56	108995.17	145115.12	133572.91	
HR (95% CI), Model 1	Reference	0.87(0.56-1.34)	0.97(0.65-1.45)	1.08(0.74-1.56)	0.82(0.54-1.25)	0.70
HR (95% CI), Model 2	Reference	0.87(0.56-1.34)	0.97(0.64-1.45)	1.07(0.74-1.56)	0.83(0.54-1.27)	0.72
Multiple sclerosis						
Events	10	9	16	14	12	
Person-years	138746.2	94524.47	108794.74	144787.17	133218.5	
HR (95% CI), Model 1	Reference	1.25(0.51-3.09)	1.89(0.85-4.19)	1.19(0.52-2.72)	1.05(0.44-2.51)	0.47
HR (95% CI), Model 2	Reference	1.27(0.51-3.15)	1.95(0.87-4.35)	1.23(0.53-2.84)	1.12(0.46-2.70)	0.47
Bronchiectasis						
Events	89	56	67	82	86	
Person-years	138627.29	94364.39	108702.67	144714.63	133191.12	
HR (95% CI), Model 1	Reference	0.85(0.61-1.19)	0.85(0.62-1.18)	0.76(0.56-1.04)	0.86(0.63-1.18)	0.55
HR (95% CI), Model 2	Reference	0.86(0.62-1.21)	0.86(0.62-1.19)	0.77(0.57-1.05)	0.87(0.63-1.19)	0.61
Asthma						
Events	246	156	185	232	194	
Person-years	119517.05	81642.17	94365.63	126437.26	116554.1	
HR (95% CI), Model 1	Reference	0.91(0.74-1.11)	0.92(0.76-1.12)	0.85(0.71-1.03)	0.77(0.63-0.94)	0.12
HR (95% CI), Model 2	Reference	0.96(0.78-1.17)	1.00(0.82-1.21)	0.93(0.78-1.13)	0.88(0.72-1.07)	0.70
COPD						
Events	311	164	186	239	173	
Person-years	136357.04	93064.85	107425.14	143250.35	131938	
HR (95% CI), Model 1	Reference	0.74(0.61-0.89)	0.71(0.59-0.86)	0.69(0.58-0.82)	0.55(0.45-0.66)	<0.0001
HR (95% CI), Model 2	Reference	0.79(0.66-0.96)	0.79(0.66-0.95)	0.78(0.66-0.93)	0.65(0.53-0.79)	0.0005
CKD						
Events	713	409	455	550	364	
Person-years	136165.57	93080.77	107232.35	143065.74	132039.93	
HR (95% CI), Model 1	Reference	0.81(0.71-0.91)	0.77(0.68-0.87)	0.70(0.63-0.79)	0.51(0.45-0.59)	<0.0001
HR (95% CI), Model 2	Reference	0.86(0.76-0.98)	0.86(0.76-0.97)	0.80(0.72-0.90)	0.62(0.54-0.71)	<0.0001
Chronic liver disease						
Events	50	27	16	33	19	
Person-years	146818.4	99860.94	114712.49	152134.06	138942.54	

HR (95% CI), Model 1	Reference	0.72(0.43-1.21)	0.34(0.18-0.64)	0.61(0.37-0.99)	0.38(0.21-0.69)	0.0020
HR (95% CI), Model 2	Reference	0.80(0.80-0.80)	0.41(0.41-0.41)	0.76(0.76-0.76)	0.53(0.53-0.53)	0.0550
Irritable bowel syndrome						
Events	157	114	122	156	109	
Person-years	135011.67	92038.92	106138.51	141079.61	130209.94	
HR (95% CI), Model 1	Reference	0.98(0.77-1.24)	0.88(0.69-1.12)	0.80(0.64-1.00)	0.58(0.45-0.74)	0.0002
HR (95% CI), Model 2	Reference	1.03(0.81-1.32)	0.95(0.75-1.21)	0.89(0.70-1.12)	0.66(0.51-0.86)	0.0096
Inflammatory bowel disease						
Events	74	40	49	58	47	
Person-years	137159.85	93555.88	107934.92	144181.22	132710.77	
HR (95% CI), Model 1	Reference	0.81(0.55-1.19)	0.87(0.60-1.25)	0.78(0.55-1.11)	0.70(0.48-1.03)	0.43
HR (95% CI), Model 2	Reference	0.83(0.56-1.22)	0.91(0.63-1.31)	0.83(0.58-1.18)	0.76(0.52-1.12)	0.68
Treated constipation						
Events	491	294	370	485	401	
Person-years	136194.75	92530.63	106843.28	142140.37	131095.31	
HR (95% CI), Model 1	Reference	0.84(0.72-0.97)	0.89(0.78-1.03)	0.87(0.76-0.99)	0.77(0.68-0.89)	0.0064
HR (95% CI), Model 2	Reference	0.86(0.74-0.99)	0.92(0.80-1.05)	0.90(0.79-1.02)	0.81(0.70-0.93)	0.0453
Dyspepsia						
Events	1473	913	1021	1325	1199	
Person-years	116892.22	80716.8	93441.44	124885.49	116882.37	
HR (95% CI), Model 1	Reference	0.86(0.80-0.94)	0.82(0.76-0.89)	0.79(0.73-0.85)	0.75(0.70-0.82)	<0.0001
HR (95% CI), Model 2	Reference	0.90(0.83-0.98)	0.86(0.80-0.94)	0.84(0.78-0.91)	0.82(0.76-0.89)	<0.0001
Diverticular disease						
Events	1259	791	862	1139	889	
Person-years	128391.52	88236.93	101974.08	135980.26	126760.37	
HR (95% CI), Model 1	Reference	0.88(0.81-0.97)	0.82(0.75-0.90)	0.81(0.75-0.88)	0.68(0.62-0.75)	<0.0001
HR (95% CI), Model 2	Reference	0.92(0.84-1.01)	0.87(0.80-0.95)	0.88(0.81-0.95)	0.76(0.69-0.83)	<0.0001
Pernicious anaemia						
Events	29	19	10	22	11	
Person-years	138770.94	94583.9	108929.1	145015.87	133411.1	
HR (95% CI), Model 1	Reference	0.87(0.49-1.56)	0.38(0.19-0.79)	0.60(0.34-1.06)	0.31(0.15-0.63)	0.0045
HR (95% CI), Model 2	Reference	0.97(0.57-1.67)	0.39(0.19-0.78)	0.68(0.40-1.16)	0.36(0.18-0.71)	0.0052
Fracture						
Events	90	68	72	100	124	
Person-years	138348.76	94128.89	108513.44	144298.19	132631.64	
HR (95% CI), Model 1	Reference	1.02(0.74-1.40)	0.91(0.66-1.24)	0.92(0.69-1.23)	1.22(0.92-1.63)	0.19
HR (95% CI), Model 2	Reference	0.99(0.72-1.36)	0.87(0.64-1.19)	0.88(0.65-1.17)	1.14(0.85-1.51)	0.29
Osteoporosis						
Events	832	557	680	841	779	
Person-years	124397.79	84539.79	97338.39	129916.3	119536.53	
HR (95% CI), Model 1	Reference	0.91(0.82-1.02)	0.94(0.85-1.04)	0.85(0.77-0.94)	0.84(0.76-0.93)	0.0045
HR (95% CI), Model 2	Reference	0.98(0.88-1.09)	1.03(0.93-1.14)	0.96(0.87-1.06)	0.99(0.90-1.10)	0.67
Meniere’s disease						
Events	21	6	7	13	11	

Person-years	138877.11	94579.62	108964.87	144894.77	133295.04	
HR (95% CI), Model 1	Reference	0.40(0.16-1.00)	0.40(0.17-0.94)	0.54(0.27-1.11)	0.49(0.23-1.06)	0.11
HR (95% CI), Model 2	Reference	0.42(0.17-1.03)	0.42(0.18-1.00)	0.58(0.28-1.20)	0.55(0.25-1.19)	0.17
Eczema						
Events	167	117	111	161	149	
Person-years	132633.06	90495.66	104578.09	138986.5	128162.27	
HR (95% CI), Model 1	Reference	1.00(0.79-1.27)	0.81(0.64-1.03)	0.88(0.71-1.10)	0.89(0.70-1.11)	0.40
HR (95% CI), Model 2	Reference	1.03(0.81-1.31)	0.83(0.65-1.06)	0.91(0.72-1.13)	0.91(0.72-1.15)	0.45
Glaucoma						
Events	199	135	167	224	223	
Person-years	136910.34	93280.94	107563.72	142864.17	131266.54	
HR (95% CI), Model 1	Reference	0.96(0.77-1.19)	1.01(0.82-1.25)	1.03(0.85-1.25)	1.14(0.93-1.39)	0.56
HR (95% CI), Model 2	Reference	0.95(0.76-1.18)	1.00(0.81-1.23)	1.01(0.83-1.23)	1.11(0.91-1.36)	0.68
Cataract						
Events	973	689	815	1079	1018	
Person-years	131533.49	89401.91	103325.36	137003.42	125677.51	
HR (95% CI), Model 1	Reference	0.95(0.86-1.05)	0.94(0.85-1.03)	0.91(0.83-0.99)	0.93(0.85-1.02)	0.33
HR (95% CI), Model 2	Reference	0.96(0.87-1.06)	0.96(0.87-1.05)	0.94(0.86-1.03)	0.97(0.89-1.07)	0.72
AMD						
Events	171	105	129	189	173	
Person-years	138495.86	94247.49	108523.73	144389.36	132890.7	
HR (95% CI), Model 1	Reference	0.83(0.65-1.06)	0.86(0.69-1.09)	0.93(0.75-1.15)	0.93(0.75-1.17)	0.58
HR (95% CI), Model 2	Reference	0.84(0.65-1.07)	0.87(0.69-1.10)	0.94(0.76-1.16)	0.94(0.75-1.18)	0.62
Thyroid disorders						
Events	273	203	219	304	300	
Person-years	131360.02	88601.49	102117.43	134993.16	123756.73	
HR (95% CI), Model 1	Reference	0.99(0.82-1.18)	0.88(0.74-1.06)	0.87(0.74-1.03)	0.88(0.75-1.05)	0.36
HR (95% CI), Model 2	Reference	1.02(0.85-1.23)	0.93(0.78-1.12)	0.94(0.79-1.11)	0.99(0.83-1.17)	0.82
Prostate disorders¶						
Events	644	350	356	422	274	
Person-years	77135.4	43741.15	46345.54	51001.07	37881.09	
HR (95% CI), Model 1	Reference	0.97(0.85-1.10)	0.90(0.82-0.99)	0.94(0.85-1.04)	0.83(0.74-0.93)	0.0204
HR (95% CI), Model 2	Reference	0.97(0.85-1.11)	0.90(0.83-0.99)	0.94(0.85-1.04)	0.84(0.75-0.94)	0.0317

AMD, age related macular degeneration; CI, confidence interval; CKD, chronic kidney disease; COPD, chronic obstructive pulmonary disease; HR, hazard ratio.

*Cox proportional hazard regression models were used to examine associations of the Healthful Plant-based Diet Index (quintiles) with the incidence of individual chronic diseases. The statistical tests were two-sided. Model 1 was adjusted for age and sex; Model 2 was adjusted for Model 1 plus ethnicity, education, income, smoking, alcohol consumption, sleep, physical activity, and GRS for longevity; Model 3 was adjusted for Model 2 and total energy intake.

†Cardiovascular disease includes coronary heart disease, heart failure, atrial fibrillation, other cardiac disease, stroke, and peripheral vascular disease.

‡All cancers encompass any type of cancer except for non-melanoma skin cancer.

[§]These analyses were conducted among women only.

[¶]These analyses were conducted among men only.

Table S13. Field codes for diseases of interest

Long term condition grouping	Conditions included as reported by participants	Field Code
1. Hypertension	Hypertension	1065
	Essential Hypertension	1072
2. Depression	Essential Depression	1072
	Postnatal Depression	1531
3. Asthma	Asthma	1111
4. Atrial fibrillation	Atrial Fibrillation	1471
5. Coronary heart disease	Heart attack/Myocardial Infarction	1075
	Angina	1074
6. Dyspepsia	Gastro-oesophageal reflux (GORD)/gastric reflux	1138
	Oesophagitis /Barrett's oesophagus	1139
	Gastric stomach ulcers	1142
	Gastric erosions/gastritis	1143
	Duodenal ulcer	1457
	Dyspepsia/indigestion	1510
	Hiatus hernia	1474
	Helicobacter pylori	1442
7. Diabetes	Diabetic nephropathy	1607
	Diabetic neuropathy/ulcers	1468
	Diabetes	1220
	Type 1 diabetes	1222
	Type 2 diabetes	1223
	Diabetic eye disease	1276
8. Thyroid disorders	Thyroid problem (not cancer)	1224
	Hyperthyroidism/thyrotoxicosis	1225
	Hypothyroidism/myxoedema	1226
	Grave’s disease	1522
	Thyroid goitre	1610
	Thyroiditis	1428
9. Chronic Obstructive Pulmonary Disease (COPD)	COPD/chronic obstructive airways disease	1112
	Emphysema/chronic bronchitis	1113
	Emphysema	1472
10. Anxiety	Anxiety/panic attacks	1287
	Nervous breakdown	1288
	Post-traumatic stress disorder	1469
	Obsessive compulsive disorder	1615
	Stress	1614
	Insomnia	1616
	Psychological/psychiatric problem	1243
11. Irritable bowel syndrome	Irritable bowel syndrome	1154
12. Alcohol use disorder	Alcohol dependency	1408
	Alcoholic liver disease/alcoholic cirrhosis	1604

13. Other psychoactive substance abuse	Opioid dependency	1409
	Other substance abuse/dependency	1410
14. Treated constipation	Constipation	1599
15. Stroke/Transient Ischaemic Attack (TIA)	Stroke	1081
	TIA	1082
	Subarachnoid haemorrhage	1083
	Brain haemorrhage	1086
	Ischaemic stroke	1583
16. Chronic kidney disease	Polycystic kidney	1427
	Diabetic nephropathy	1607
	Renal/kidney failure	1192
	Renal failure requiring dialysis	1193
	Renal failure not requiring dialysis	1194
	Kidney nephropathy	1519
	Immunoglobulin A (IgA) nephropathy	1520
17. Diverticular disease	Diverticular disease	1458
	Diverticulitis	1458
18. Peripheral vascular disease	Peripheral vascular disease	1067
	Leg claudication/intermittent claudication	1087
19. Heart failure	Cardiomyopathy	1079
	Hypertrophic cardiomyopathy	1588
	Heart failure/pulmonary oedema	1076
20. Prostate disorders	Prostate problem (not cancer)	1207
	Enlarged prostate	1396
	Benign prostatic hypertrophy	1516
21. Epilepsy	Epilepsy	1264
22. Dementia	Dementia, Alzheimer's disease, Cognitive impairment	1263
23. Schizophrenia/bipolar disorder	Schizophrenia	1289
	mania/bipolar disorder/manic depression	1291
24. Psoriasis/eczema	Eczema/dermatitis	1452
	Psoriasis	1453
25. Inflammatory Bowel Disease	Inflammatory Bowel Disease	1461
	Crohn's disease	1462
	Ulcerative colitis	1463
26. Migraine	Migraine	1265
27. Bronchiectasis	Bronchiectasis	1114
28. Parkinson's disease	Parkinson's disease	1262
29. Multiple Sclerosis	Multiple Sclerosis	1261
30. Osteoporosis	Osteoporosis	1465
31. Chronic liver disease	Oesophageal varices	1141
	Non infective hepatitis	1157
	Liver failure/cirrhosis	1158
	Primary biliary cirrhosis	1506
32. Meniere's disease	Meniere's disease	1421

33. Pernicious Anaemia	Pernicious Anaemia	1331
34. Other heart/cardiac problem	Heart/cardiac problem	1066
35. Fracture	Fracture pelvis	1647
	Fracture neck of femur / hip	1648
	Fracture patella / knee	1650
36. Glaucoma	Glaucoma	1277
37. Cataract	Cataract	1278
38. AMD	AMD	1528
39. Lung Cancer	Lung Cancer	1001
40. Skin Cancer	Non-melanoma Cancer	1060
41. Melanoma	Melanoma	1059
42. Stomach Cancer	Stomach cancer	1018
43. Oesophageal cancer	Lung Cancer	1017
44. Colon cancer	Colon cancer	1022
45. Prostate cancer	Prostate cancer	1044
46. Ovarian cancer	Ovarian cancer	1039
47. Breast cancer	Breast cancer	1002
48. other cancers	Cancer of lip/mouth/pharynx/oral cavity	1004
	Salivary gland cancer	1005
	larynx/throat cancer	1006
	nasal cavity cancer	1007
	ear cancer	1008
	sinus cancer	1009
	lip cancer	1010
	tongue cancer	1011
	gum cancer	1012
	parotid gland cancer	1015
	other salivary gland cancer	1016
	small intestine/small bowel cancer	1019
	large bowel cancer/colorectal cancer	1020
	anal cancer	1021
	liver/hepatocellular cancer	1024
	gallbladder/bile duct cancer	1025
	pancreas cancer	1026
	small cell lung cancer	1027
	non-small cell lung cancer	1028
	peripheral nerve/autonomic nerve cancer	1029
	eye and/or adnexal cancer	1030
	meningeal cancer / malignant meningioma	1031
	brain cancer / primary malignant brain tumour	1032
	spinal cord or cranial nerve cancer	1033
	kidney/renal cell cancer	1034
	bladder cancer	1035

other cancer of urinary tract	1036
female genital tract cancer	1037
male genital tract cancer	1038
cervical cancer	1041
vaginal cancer	1042
vulval cancer	1043
testicular cancer	1045
penis cancer	1046
lymphoma	1047
leukaemia	1048
multiple myeloma	1050
myelofibrosis or myelodysplasia	1051
hodgkins lymphoma / hodgkins disease	1052
non-hodgkins lymphoma	1053
chronic lymphocytic	1055
chronic myeloid	1056
other haematological malignancy	1058
basal cell carcinoma	1061
squamous cell carcinoma	1062
primary bone cancer	1063
mesothelioma	1064
thyroid cancer	1065
parathyroid cancer	1066
adrenal cancer	1067
sarcoma/fibrosarcoma	1068
malignant lymph node, unspecified	1070
metastatic cancer (unknown primary)	1071
cin/pre-cancer cells cervix	1072
rodent ulcer	1073
acute myeloid leukaemia	1074
retinoblastoma	1075
kaposis sarcoma	1076
mouth cancer	1077
tonsil cancer	1078
oropharynx / oropharyngeal cancer	1079
trachea cancer	1080
thymus cancer / malignant thymoma	1081
heart / mediastinum cancer	1082
respiratory / intrathoracic cancer	1084
bone metastases / bony secondaries	1085
appendix cancer	1086
fallopian tube cancer	1087
malignant insulinoma	1088

Table S14. ICD codes for diseases of interest

Long term condition grouping	Conditions included as reported by participants	ICD10 code	ICD9 code
1. Hypertension	Essential (primary) hypertension	I10	401
	Hypertensive heart disease	I11	402
	Hypertensive heart disease with (congestive) heart failure	I110	403
	Hypertensive heart disease without (congestive) heart failure	I119	404
	Hypertensive renal disease	I12	405
	Hypertensive renal disease with renal failure	I120	
	Hypertensive renal disease without renal failure	I129	
	Hypertensive heart and renal disease	I13	
	Hypertensive heart and renal disease with (congestive) heart failure	I130	
	Hypertensive heart and renal disease with renal failure	I131	
	Hypertensive heart and renal disease with both (congestive) heart failure and renal failure	I132	
	Hypertensive heart and renal disease, unspecified	I139	
	Secondary hypertension	I15	
	Renovascular hypertension	I150	
	Hypertension secondary to other renal disorders	I151	
	Hypertension secondary to endocrine disorders	I152	
	Other secondary hypertension	I158	
	Secondary hypertension, unspecified	I159	
2. Depression	Depressive episode	F32	2962
	Recurrent depressive disorder	F33	2963
	Dysthymia	F341	3004
	Other recurrent mood [affective] disorders	F381	311
	Postschizophrenic depression	F204	
3. Asthma	Asthma	J45	493
	Predominantly allergic asthma	J450	
	Nonallergic asthma	J451	
	Mixed asthma	J458	
	Asthma, unspecified	J459	
4. Atrial Fibrillation	Atrial fibrillation and flutter	I48	427
	Paroxysmal atrial fibrillation	I480	
	Persistent atrial fibrillation	I481	
	Chronic atrial fibrillation	I482	
	Atrial fibrillation and atrial flutter, unspecified	I489	
5. Coronary heart disease	Angina pectoris	I20	413
	Acute myocardial infarction	I21	410
	Subsequent myocardial infarction	I22	411
	Certain current complications following acute myocardial infarction	I23	412
	Other acute ischaemic heart diseases	I24	414
	Chronic ischaemic heart disease	I25	
6. Dyspepsia	Gastro-oesophageal reflux disease	K21	53081
	Gastro-oesophageal reflux disease with oesophagitis	K210	5368

	Gastro-oesophageal reflux disease without oesophagitis	K219	
	Oesophagitis	K20	
	Barrett’s oesophagus	K227	
	Other specified diseases of oesophagus	K228	
	Disease of oesophagus, unspecified	K229	
	Disorders of oesophagus in diseases classified elsewhere	K23	
	Gastric ulcer	K25	
	Gastritis and duodenitis	K29	
	Duodenal ulcer	K26	
	Dyspepsia	K30	
	Congenital hiatus hernia	Q401	
	Helicobacter pylori [H.pylori] as the cause of diseases classified to other chapters	B980	
7. Diabetes	Diabetic polyneuropathy	G632	250
	Diabetic mononeuropathy	G590	
	Diabetic retinopathy	H360	
	Diabetic cataract	H280	
	Insulin-dependent diabetes mellitus	E10	
	Non-insulin-dependent diabetes mellitus	E11	
	Malnutrition-related diabetes mellitus	E12	
	Other specified diabetes mellitus	E13	
	Unspecified diabetes mellitus	E14	
	Congenital iodine-deficiency syndrome	E00	240
8. Thyroid disorders	Thyrotoxicosis [hyperthyroidism]	E05	241
	Other hypothyroidism	E03	242
	Other non-toxic goitre	E04	243
	Iodine-deficiency-related thyroid disorders and allied conditions	E01	244
	Thyroiditis	E06	245
	Other disorders of thyroid	E07	246
	Subclinical iodine-deficiency hypothyroidism	E02	
9. Chronic obstructive pulmonary disease (COPD)	Simple and mucopurulent chronic bronchitis	J41	491
	Unspecified chronic bronchitis	J42	
	Emphysema	J43	492
	Other chronic obstructive pulmonary disease	J44	494
10. Anxiety	Phobic anxiety disorders	F40	3000
	Other anxiety disorders	F41	3002
	Reaction to severe stress, and adjustment disorders	F43	3009
	Posttraumatic stress disorder	F431	
	Obsessive-compulsive disorder	F42	3003
	Stress, not elsewhere classified	Z733	308
	Disorders of initiating and maintaining sleep [insomnias]	G470	7805
	Mental disorder, not otherwise specified	F99	
11. Irritable bowel syndrome	Irritable bowel syndrome	K58	5641
	Dependence syndrome	**F102**	**3039**

12. Alcohol use disorder	Alcoholic liver disease	K70	291
	Harmful use	F101	303
13. Other psychoactive substance abuse	Harmful use	F111	304
	Acute intoxication	F100	305
	Dependence syndrome	F112	
	Unspecified mental and behavioural disorder	F119	
14. Treated constipation	Constipation	K590	5640
15. Stroke/Transient Ischaemic Attack (TIA)	Stroke, not specified as haemorrhage or infarction	I64	438
	Occlusion and stenosis of precerebral arteries, not resulting in cerebral infarction	I65	435
	Subarachnoid haemorrhage	I60	430
	Intracerebral haemorrhage	I61	431
	Other nontraumatic intracranial haemorrhage	I62	432
	Occlusion and stenosis of cerebral arteries, not resulting in cerebral infarction	I66	433
	Cerebral infarction	I63	434
	Acute but ill-defined cerebrovascular disease		436
	Other and ill-defined cerebrovascular disease		437
16. Chronic kidney disease	Polycystic kidney, infantile type	Q611	75315
	Polycystic kidney, adult type	Q612	
	Polycystic kidney, unspecified	Q613	
	Acute renal failure	N17	584
	Chronic renal failure	N18	585
	Unspecified renal failure	N19	586
	Renal complications	E112	587
	Other	N028	588
17. Diverticular disease	Diverticular disease of intestine	K57	562
18. Peripheral vascular disease	Other aneurysm	I72	440
	Other peripheral vascular diseases	I73	443
	Arterial embolism and thrombosis		444
19. Heart failure	Cardiomyopathy	I42	425
	Heart failure	I50	428
20. Prostate disorders	Hyperplasia of prostate	N40	600
	Inflammatory diseases of prostate	N41	601
	Other disorders of prostate	N42	602
	Disorders of prostate in diseases classified elsewhere	N510	
21. Epilepsy	Epilepsy	G40	345
22. Dementia	Creutzfeldt-Jakob disease	A810	290
	Dementia in Alzheimer's disease	F00	
	Vascular dementia	F01	
	Unspecified dementia	F03	
	Delirium superimposed on dementia	F051	
	Amnesic syndrome	F106	
	Alzheimer's disease	G30	
	Other degenerative diseases of nervous system, not elsewhere classified	G31	

	Progressive vascular leukoencephalopathy	I673	
23. Schizophrenia/bipolar disorder	Schizophrenia	F20	295
	Schizotypal disorder	F21	
	Manic episode	F30	296
	Bipolar affective disorder	F31	
24. Psoriasis/eczema	Atopic dermatitis	L20	696
	Seborrhoeic dermatitis	L21	692
	Diaper [napkin] dermatitis	L22	
	Allergic contact dermatitis	L23	
	Irritant contact dermatitis	L24	
	Unspecified contact dermatitis	L25	
	Exfoliative dermatitis	L26	
	Dermatitis due to substances taken internally	L27	
	Other dermatitis	L30	
	Psoriasis	L40	
	Parapsoriasis	L41	
25. Inflammatory Bowel Disease	Crohn's disease [regional enteritis]	K50	
	Ulcerative colitis	K51	
26. Migraine	Migraine	G43	346
27. Bronchiectasis	Bronchiectasis	J47	494
28. Parkinson’s disease	Parkinson's disease	G20	332
	Secondary Parkinsonism	G21	3321
	Parkinsonism in diseases classified elsewhere	G22	333
	Other degenerative diseases of basal ganglia	G23	
	Extrapyramidal and movement disorder, unspecified	G259	
	Extrapyramidal and movement disorders in diseases classified elsewhere	G26	
	Multisystem degeneration	G903	
29. Multiple Sclerosis	Multiple sclerosis	G35	340
30. Chronic liver disease	Oesophageal varices	I85	571
	Toxoplasma hepatitis	B581	
	Alcoholic hepatitis	K701	
	Toxic liver disease with acute hepatitis	K712	
	Toxic liver disease with chronic persistent hepatitis	K713	
	Toxic liver disease with chronic lobular hepatitis	K714	
	Toxic liver disease with chronic active hepatitis	K715	
	Toxic liver disease with hepatitis, not elsewhere classified	K716	
	Fibrosis and cirrhosis of liver	K74	
	Primary biliary cirrhosis	K743	
31. Osteoporosis	Polyarthrosis	M15	7330
	Primary generalised (osteo)arthrosis	M150	
	Primary generalized (osteo)arthrosis, Multiple sites	M1500	
	Heberden's nodes (with arthropathy)	M151	
	Coxarthrosis [arthrosis of hip]	M16	
	Gonarthrosis [arthrosis of knee]	M17	

32. Meniere's disease	Meniere's disease	H810	3860
33. Pernicious Anaemia	Vitamin B12 deficiency anaemia	D51	2810
34. Heart/cardiac problem	Cardiac arrest	I46	393
	Other cardiac arrhythmias	I49	394
	Complications and ill-defined descriptions of heart disease	I51	395
	Other heart disorders in diseases classified elsewhere	I52	396
	Other cerebrovascular diseases	I67	397
	Cerebrovascular disorders in diseases classified elsewhere	I68	398
	Sequelae of cerebrovascular disease	I69	399
	Atherosclerosis	I70	400
	Acute pericarditis		420
	Acute and subacute endocarditis		421
	Acute myocarditis		422
	Other diseases of pericardium		423
	Other diseases of endocardium		424
	Conduction disorders		426
	Cardiac dysrhythmias		427
	Ill-defined descriptions and complications of heart disease		429
35. Fracture	Multiple fractures of lumbar spine and pelvis	S327	808
	Multiple fractures of lumbar spine and pelvis (closed)	S3270	
	Fracture of femur	S72	820
	Fracture of patella	S820	8210
	Fracture of patella (closed)	S8200	824
36. Glaucoma	Glaucoma	H40	365
37. Cataract	Senile cataract	H25	366
	Other cataract	H26	
	Cataract and other disorders of lens in diseases classified elsewhere	H28	
38. AMD	Degeneration of macula and posterior pole	H353	3625
39. Lung Cancer	Malignant neoplasm of bronchus and lung	C34	162
40. non-melanoma skin cancer	Other malignant neoplasms of skin	C44	173
	Mesothelioma	C45	
41. Melanoma	Malignant melanoma of skin	C43	172
42. Stomach Cancer	Malignant neoplasm of stomach	C16	151
43. Oesophageal cancer	Malignant neoplasm of oesophagus	C15	150
44. Colon cancer	Malignant neoplasm of colon	C18	153
45. prostate cancer	Malignant neoplasm of prostate	C61	185
46. ovarian cancer	Malignant neoplasm of ovary	C56	183
47. Breast cancer	Malignant neoplasm of breast	C50	174
48. other cancers	Malignant neoplasm of lip	C00	140
	Malignant neoplasm of base of tongue	C01	141
	Malignant neoplasm of other and unspecified parts of tongue	C02	142
	Malignant neoplasm of gum	C03	143

Malignant neoplasm of floor of mouth	C04	144
Malignant neoplasm of palate	C05	145
Malignant neoplasm of other and unspecified parts of mouth	C06	146
Malignant neoplasm of parotid gland	C07	147
Malignant neoplasm of other and unspecified major salivary glands	C08	148
Malignant neoplasm of tonsil	C09	149
Malignant neoplasm of oropharynx	C10	152
Malignant neoplasm of nasopharynx	C11	155
Malignant neoplasm of pyriform sinus	C12	156
Malignant neoplasm of hypopharynx	C13	157
Malignant neoplasm of other and ill-defined sites in the lip, oral cavity and pharynx	C14	158
Malignant neoplasm of small intestine	C17	159
Malignant neoplasm of rectosigmoid junction	C19	160
Malignant neoplasm of anus and anal canal	C21	161
Malignant neoplasm of liver and intrahepatic bile ducts	C22	163
Malignant neoplasm of gallbladder	C23	164
Malignant neoplasm of other and unspecified parts of biliary tract	C24	165
Malignant neoplasm of pancreas	C25	166
Malignant neoplasm of other and ill-defined digestive organs	C26	167
Malignant neoplasm of nasal cavity and middle ear	C30	171
Malignant neoplasm of accessory sinuses	C31	175
Malignant neoplasm of larynx	C32	176
Malignant neoplasm of trachea	C33	177
Malignant neoplasm of thymus	C37	180
Malignant neoplasm of heart, mediastinum and pleura	C38	181
Malignant neoplasm of other and ill-defined sites in the respiratory system and intrathoracic organs	C39	184
Malignant neoplasm of bone and articular cartilage of limbs	C40	186
Malignant neoplasm of bone and articular cartilage of other and unspecified sites	C41	187
hematopoietic and reticuloendothelial systems (ICD-O-3 specific)	C42	188
Kaposi's sarcoma	C46	189
Malignant neoplasm of peripheral nerves and autonomic nervous system	C47	190
Malignant neoplasm of retroperitoneum and peritoneum	C48	191
Malignant neoplasm of other connective and soft tissue	C49	192
Malignant neoplasm of vulva	C51	193
Malignant neoplasm of vagina	C52	194
Malignant neoplasm of cervix uteri	C53	195
Malignant neoplasm of corpus uteri	C54	196
Malignant neoplasm of other and unspecified female genital organs	C57	197
Malignant neoplasm of placenta	C58	198
Malignant neoplasm of penis	C60	200
Malignant neoplasm of testis	C62	201
Malignant neoplasm of other and unspecified male genital organs	C63	202
Malignant neoplasm of kidney, except renal pelvis	C64	203

	Malignant neoplasm of renal pelvis	C65	204
	Malignant neoplasm of ureter	C66	205
	Malignant neoplasm of bladder	C67	206
	Malignant neoplasm of other and unspecified urinary organs	C68	207
	Malignant neoplasm of eye and adnexa	C69	208
	Malignant neoplasm of meninges	C70	
	Malignant neoplasm of brain	C71	
	Malignant neoplasm of spinal cord, cranial nerves and other parts of central nervous system	C72	
	Malignant neoplasm of thyroid gland	C73	
	Malignant neoplasm of adrenal gland	C74	
	Malignant neoplasm of other endocrine glands and related structures	C75	
	Malignant neoplasm of other and ill-defined sites	C76	
	Secondary and unspecified malignant neoplasm of lymph nodes	C77	
	Secondary malignant neoplasm of respiratory and digestive organs	C78	
	Secondary malignant neoplasm of other sites	C79	
	Malignant neoplasm without specification of site	C80	
	Hodgkin's disease	C81	
	Follicular [nodular] non-Hodgkin's lymphoma	C82	
	Diffuse non-Hodgkin's lymphoma	C83	
	Peripheral and cutaneous T-cell lymphomas	C84	
	Other and unspecified types of non-Hodgkin's lymphoma	C85	
	Other specified types of T/NK-cell lymphoma	C86	
	Malignant immunoproliferative diseases	C88	
	Multiple myeloma and malignant plasma cell neoplasms	C90	
	Lymphoid leukaemia	C91	
	Myeloid leukaemia	C92	
	Monocytic leukaemia	C93	
	Other leukaemias of specified cell type	C94	
	Leukaemia of unspecified cell type	C95	
	Other and unspecified malignant neoplasms of lymphoid, haematopoietic and related tissue	C96	
	Malignant neoplasms of independent (primary) multiple sites	C97	

Table S15. Scoring for the Alternate Mediterranean Diet Index

Food groups	Food items	Scoring	
		Criteria for 0 point	Criteria for 1 point
Whole grains	Porridge, muesli, oat crunch, bran cereal, cereal bar, non-white bread (flour types, brown, wholemeal, other type), seeded or other bread, crispbread, whole-wheat cereal, other cereal, whole meal pasta, brown rice, couscous, other cooked grains	<2 servings/day	≥2 servings/day
Fruits	Mixed fruit, apple, banana, berries, cherries, grapefruit, grapes, mango, melon, orange, orange-like small fruits, peach/nectarine, pear, pineapple, plum, other fruits, stewed/cooked fruit, prunes, other dried fruit	<2 servings/day	≥2 servings/day
Vegetables	Mixed vegetables, vegetable pieces, coleslaw, side salad, beetroot, broccoli, butternut squash, cabbage/kale, carrots, cauliflower, celery, courgette, cucumber, garlic, leeks, lettuce, mushrooms, onion, parsnip, sweet peppers, spinach, sprouts, sweetcorn, sweet potato, fresh tomatoes, cooked or tinned tomatoes, turnip/swede, watercress, other vegetable	<2 servings/day	≥2 servings/day
Nuts	Salted peanuts, unsalted peanuts, salted nuts, unsalted nuts, seeds	<0.17 servings/day	≥0.17 servings/day
Legumes	Baked beans, other beans or lentils, broad beans, green beans, peas, soy or vegetable milk, vegetarian sausages/burgers, tofu, quorn, other vegetarian alternative	0 serving/day	>0 serving/day
Fish	Tinned tuna, oily fish, breaded fish, battered fish, white fish, prawns, lobster/crab, shellfish, other fish	0 serving/day	>0 serving/day
Red meat	Sausage, beef, pork, lamb, crumbed or deep-fried poultry, poultry, bacon, ham, liver, other meat	≥0.5 servings/day	<0.5 servings/day
Alcohol	Red wine, white wine, rose wine, fortified wine, beer, spirit, others	<5 or >15 g/day	5-15 g/day
Ratio of MUFA to SFA		<1.12 servings/day	≥1.12 servings/day

Table S16. Scoring for the Empirical Dietary inflammatory index

Food groups	Food items	Scoring			
		Minimum score	Maximum score	Criteria for minimum score (0)	Criteria for maximum score (10)
Processed meant	Sausage, bacon, ham	0	1.38	0 serving/day	≥1 serving/day
Red meat	Beef, pork, lamb				
Women		0	1.33	0 serving/day	≥0.5 serving/day
Men		0	1.33	0 serving/day	≥0.75 serving/day
Organ meat	Liver	0	0.4	0 serving/day	>0 serving/day
Fish	Tinned tuna, breaded fish, battered fish, white fish, prawns, lobster/crab, shellfish, other fish	0	0.35	0 serving/day	≥0.5 serving/day
Dark yellow vegetables	Carrots, sweet potato, butternut squash, beetroot, parsnip, turnip/swede				
Women		-0.78	0	>0.75 serving/day	0 serving/day
Men		-0.78	0	≥0.5 serving/day	0 serving/day
Green leafy vegetables	Cabbage/kale, lettuce, spinach, watercress				
Women		-0.78	0	>0.6 serving/day	0 serving/day
Men		-0.78	0	≥0.5 serving/day	0 serving/day
Other vegetables	Other vegetable				
Women		0	0.4	0 serving/day	≥3.5 serving/day
Men		0	0.4	0 serving/day	≥3.1 serving/day
Refined grains	Sweetened cereal, plain cereal, white bread, naan bread, garlic bread, white pasta, white rice, pancake, scotch pancake, croissant, scone, savoury or cheesy biscuits, other savoury snack, snackpot				
Women		0	1.62	0 serving/day	≥4 serving/day
Men		0	1.62	0 serving/day	≥5 serving/day
High-energy beverages	Fizzy drink, fruit smoothie, dairy smoothie				
Women		0	1.5	0 serving/day	≥0.25 serving/day
Men		0	1.5	0 serving/day	≥0.33 serving/day
Low-energy beverages	Low-energy beverages				
Women		0	1.1	0 serving/day	≥0.25 serving/day

Men		0	1.1	0 serving/day	>0 serving/day
Tomato	Tinned tomato, fresh tomato				
Women		0	0.92	0 serving/day	≥1 serving/day
Men		0	0.92	0 serving/day	≥0.56 serving/day
Beer	Beer				
Women		-1.06	0	>0 serving/day	0 serving/day
Men		-1.06	0	≥1 serving/day	0 serving/day
Wine	Red wine, white wine, rose wine, fortified wine, spirit, others				
Women		-2.11	0	>1.5 serving/day	0 serving/day
Men		-2.11	0	≥2 serving/day	0 serving/day
Tea	Standard tea, rooibos tea, green tea, herbal tea, other tea				
Women		0	0.11	0 serving/day	≥4.5 serving/day
Men		0	0.11	0 serving/day	≥4 serving/day
Coffee	Instant coffee, filtered coffee, cappuccino, latte, espresso, other coffee drinks	-2.83	0	≥3 serving/day	0 serving/day
Fruit juices	Orange juice, grapefruit juice, other fruit/vegetable juice	-0.22	0	≥1 serving/day	0 serving/day
Snacks	Fried potatoes, sweetcorn, crackers/crispbreads with butter/margarine	-0.33	0	≥0.5 serving/day	0 serving/day
Pizza	Pizza	-0.44	0	>0 serving/day	0 serving/day

Table S17. Scoring for the Alternate Healthy Eating Index-2010

Food groups	Scoring	
	Criteria for minimum score (0)	Criteria for maximum score (10)
Whole grains, servings/d		
Women	0	≥5
Men	0	≥6
Fruits, servings/d	0	≥4
Vegetables, servings/d	0	≥5
Nuts and legumes, servings/d	0	≥1
Sugar-sweetened beverages and fruit juice, servings/d	≥1	0
Red meat/processed meat, servings/d	≥1.5	0
Fish, servings/d	0	≥1
PUFA, % of energy	≤2	≥10
Adding salt to foods	Always	Never/rarely
Alcohol, drinks/d		
Women	≥2.5	0.5–1.5
Men	≥3.5	0.5–2.0

Table S18. Scoring for the Healthful Plant-based Diet Index

Food groups	Food items	Scoring
Whole grains	Porridge, muesli, oat crunch, bran cereal, cereal bar, non-white bread (flour types, brown, wholemeal, other type), seeded or other bread, crispbread, whole-wheat cereal, other cereal, whole meal pasta, brown rice, couscous, other cooked grains	Positive
Fruits	Mixed fruit, apple, banana, berries, cherries, grapefruit, grapes, mango ,melon, orange, orange-like small fruits, peach/nectarine, pear, pineapple, plum, other fruits, stewed/cooked fruit, prunes, other dried fruit	Positive
Vegetables	Mixed vegetables, vegetable pieces, coleslaw, side salad, beetroot, broccoli, butternut squash, cabbage/kale, carrots, cauliflower, celery, courgette, cucumber, garlic, leeks, lettuce, mushrooms, onion, parsnip, sweet peppers, spinach, sprouts, sweetcorn, sweet potato, fresh tomatoes, cooked or tinned tomatoes, turnip/swede, watercress, other vegetable	Positive
Nuts	Salted peanuts, unsalted peanuts, salted nuts, unsalted nuts, seeds	Positive
Legumes	Baked beans, other beans or lentils, broad beans, green beans, peas, soy or vegetable milk, vegetarian sausages/burgers, tofu, quorn, other vegetarian alternative	Positive
Tea and coffee	Instant coffee, filtered coffee, cappuccino, latte, espresso, other coffee drinks, standard tea, rooibos tea, green tea, herbal tea, other tea	Positive
Refined grains	Sweetened cereal, plain cereal, white bread, naan bread, garlic bread, white pasta, white rice, pancake, scotch pancake, croissant, scone, savoury or cheesy biscuits, other savoury snack, snackpot	Reverse
Potatoes	Fried potatoes, boiled/baked potatoes, mashed potatoes, crisps (e.g., potato chips)	Reverse
Sugary drinks	Low calorie or diet drinks (e.g. fizzy, squash), carbonated (fizzy) drinks, squash or cordial	Reverse
Fruit juices	Orange juice, grapefruit juice, other fruit/vegetable juice, fruit smoothie	Reverse
Sweets and desserts	Double crust pie, single crust pie/flan, crumble topping, Yorkshire pudding, Danish pastry, fruitcake, cake, doughnuts, sponge pudding, other dessert, chocolate bar, white chocolate, milk chocolate, dark chocolate, chocolate-covered raisin, chocolate sweet, diet sweets, chocolate-covered biscuits, chocolate biscuits, sweet biscuits, other sweets	Reverse
Animal fat	Butter on bread/crackers (spreadable, low fat, normal fat, or unknown type), dairy spread on bread/crackers (very low fat, low fat, normal fat, unknown type)	Reverse
Dairy	Milk, dairy smoothie, flavored milk, yogurt, ice-cream, low fat hard cheese, hard cheese, soft cheese, blue cheese, low fat cheese spread, cheese spread, cottage cheese, feta cheese, mozzarella cheese, goat's cheese, other cheese, cheesecake, milk-based pudding, other milk-based pudding	Reverse
Eggs	Whole eggs, omelettes or scrambled egg, eggs in sandwiches, scotch egg, other egg dishes	Reverse
Fish/seafood	Tinned tuna, oily fish, breaded fish, battered fish, white fish, prawns, lobster/crab, shellfish, other fish	Reverse
Meat	Sausage, beef, pork, lamb, crumbed or deep-fried poultry, poultry, bacon, ham, liver, other meat	Reverse
Miscellaneous anima based foods	Pizza, Indian snacks	Reverse
