# Supplementary material for: Healthy dietary patterns and the risk of individual chronic diseases in community-dwelling adults
Source: Nat Commun. 2023 Oct 23;14:6704. doi: 10.1038/s41467-023-42523-9 (PMC10593819; doi:10.1038/s41467-023-42523-9)
Supplement: Supplementary file 3 — Reporting Summary [file 41467_2023_42523_MOESM3_ESM.pdf]

nature portfolio

Corresponding author(s): Xianwen Shang, Mingguang He, Honghua Yu

Last updated by author(s): Sep 27, 2023

Reporting Summary

Nature Portfolio wishes to improve the reproducibility of the work that we publish. This form provides structure for consistency and transparency in reporting. For further information on Nature Portfolio policies, see our Editorial Policies and the Editorial Policy Checklist.

Statistics

For all statistical analyses, confirm that the following items are present in the figure legend, table legend, main text, or Methods section.

n/a	Confirmed	
☐	☒	The exact sample size (*n*) for each experimental group/condition, given as a discrete number and unit of measurement
☐	☒	A statement on whether measurements were taken from distinct samples or whether the same sample was measured repeatedly
☐	☒	The statistical test(s) used AND whether they are one- or two-sided<br>*Only common tests should be described solely by name; describe more complex techniques in the Methods section.*
☐	☒	A description of all covariates tested
☐	☒	A description of any assumptions or corrections, such as tests of normality and adjustment for multiple comparisons
☐	☒	A full description of the statistical parameters including central tendency (e.g. means) or other basic estimates (e.g. regression coefficient) AND variation (e.g. standard deviation) or associated estimates of uncertainty (e.g. confidence intervals)
☐	☒	For null hypothesis testing, the test statistic (e.g. *F*, *t*, *r*) with confidence intervals, effect sizes, degrees of freedom and *P* value noted<br>*Give P values as exact values whenever suitable.*
☒	☐	For Bayesian analysis, information on the choice of priors and Markov chain Monte Carlo settings
☒	☐	For hierarchical and complex designs, identification of the appropriate level for tests and full reporting of outcomes
☐	☒	Estimates of effect sizes (e.g. Cohen's *d*, Pearson's *r*), indicating how they were calculated

Our web collection on statistics for biologists contains articles on many of the points above.

Software and code

Policy information about availability of computer code

Data collection	Questionnaire and clinical lab tests were used to collect data in the UK Biobank.
Data analysis	All data analyses were performed using SAS 9.4 for Windows (SAS Institute Inc.). The codes used for analyses in this study are available upon request. Access to codes will be granted for requests for academic use within 3 weeks of application (Dr. Xianwen Shang).

For manuscripts utilizing custom algorithms or software that are central to the research but not yet described in published literature, software must be made available to editors and reviewers. We strongly encourage code deposition in a community repository (e.g. GitHub). See the Nature Portfolio guidelines for submitting code & software for further information.

Data

Policy information about availability of data

All manuscripts must include a data availability statement. This statement should provide the following information, where applicable:

- Accession codes, unique identifiers, or web links for publicly available datasets
- A description of any restrictions on data availability
- For clinical datasets or third party data, please ensure that the statement adheres to our policy

Data from the UK Biobank (ukbiobank.ac.uk/) are available to researchers on application to the UK Biobank (https://www.ukbiobank.ac.uk/). The present study was conducted under application number 62443 of the UK Biobank resource.

Research involving human participants, their data, or biological material

Policy information about studies with human participants or human data. See also policy information about sex, gender (identity/presentation), and sexual orientation and race, ethnicity and racism.

Reporting on sex and gender	The UK Biobank collects biological sex of participants, which was determined by self-reported data. We reported the results in the whole population in the manuscript. We have conducted moderation analysis by gender and found that the association between alternate Mediterranean diet score and incident diabetes was stronger in women than in men (Figure S1). No other significant interactions were found after controlling false discovery rate. This means that the associations between dietary patterns and the risk of chronic diseases did not differ between genders.
Reporting on race, ethnicity, or other socially relevant groupings	Ethnicity was treated as a confounder in the analysis. As more than 96% of the participants were white, it is not possible to perform data analysis stratified by ethnicity. This has been discussed as a limitation:<br>Finally, most of the participants in our study were Caucasians thus our findings may not be generalized to other ethnic groups.
Population characteristics	We included 121,513 participants (55.9% females) aged 30-75 (mean ± SD: 59.0 ± 7.9) years at baseline in the final analysis.Individuals with higher dietary scores were more likely to be older, be highly educated, exercise, and less likely to smoke. Individuals with higher AMED, AHEI-2010, or HPDI scores were more likely to be female whereas those with a higher AEDII score were more likely to be male (Table 1).
Recruitment	The participants were recruited from one of the 22 assessment centres throughout the United Kingdom. Details of the study design have been shown elsewhere (PMID: 25826379).
Ethics oversight	The UK Biobank Study's ethical approval has been granted by the National Information Governance Board for Health and Social Care and the NHS North West Multicenter Research Ethics Committee (REC reference: 16/NW/0274)

Note that full information on the approval of the study protocol must also be provided in the manuscript.

Field-specific reporting

Please select the one below that is the best fit for your research. If you are not sure, read the appropriate sections before making your selection.

☒ Life sciences ☐ Behavioural & social sciences ☐ Ecological, evolutionary & environmental sciences

For a reference copy of the document with all sections, see nature.com/documents/nr-reporting-summary-flat.pdf

Life sciences study design

All studies must disclose on these points even when the disclosure is negative.

Sample size	Baseline data on 502505 participants were collected. The number of newly diagnosed cases varied from 94 for multiple sclerosis to 9815 for dyspepsia, which allows us to detect the major findings of our study with a statistical power of >80%.
Data exclusions	Individuals with no data on diet (n=295,101), or with only one dietary assessment (n=83,413) were excluded from the analysis. Individuals with total energy intake in either the highest or lowest percentile were excluded from the analysis (n=2478). We included 121,513 participants (55.9% females) aged 30-75 (mean ± SD: 59.0 ± 7.9) years at baseline in the final analysis.
Replication	A sub-cohort of the UK Biobank completed the assessment on ≥1 of the five occasions (n=207404) between April 2009 and June 2012. Individuals who completed ≥2 dietary assessments were included in the analysis (n=121513). We repeated the analyses among participants with 3 or more dietary assessments (n=69420).
Randomization	Randomization was not directly relevant to this observational study. Covariates were adjusted for in statistical models.
Blinding	Health-related outcomes (inpatients/mortality data) were blindly linked to baseline data. Data provided to the researchers did not contain any of personally identifiable variables.

Reporting for specific materials, systems and methods

We require information from authors about some types of materials, experimental systems and methods used in many studies. Here, indicate whether each material, system or method listed is relevant to your study. If you are not sure if a list item applies to your research, read the appropriate section before selecting a response.

Materials & experimental systems

n/a	Involved in the study
☒	☐ Antibodies
☒	☐ Eukaryotic cell lines
☒	☐ Palaeontology and archaeology
☒	☐ Animals and other organisms
☒	☐ Clinical data
☒	☐ Dual use research of concern
☒	☐ Plants

Methods

n/a	Involved in the study
☒	☐ ChIP-seq
☒	☐ Flow cytometry
☒	☐ MRI-based neuroimaging
